# Chromosome-scale assembly of the *Monopterus* genome

**DOI:** 10.1093/gigascience/giy046

**Published:** 2018-04-24

**Authors:** Xueya Zhao, Majing Luo, Zhigang Li, Pei Zhong, Yibin Cheng, Fengling Lai, Xin Wang, Jiumeng Min, Mingzhou Bai, Yulan Yang, Hanhua Cheng, Rongjia Zhou

**Affiliations:** 1 Hubei Key Laboratory of Cell Homeostasis, Laboratory of Molecular and Developmental Genetics, College of Life Sciences, Wuhan University, Wuhan 430072, P. R. China; 2 BGI Genomics, BGI-Shenzhen, Shenzhen 518083, P. R. China

**Keywords:** *whole-genome sequencing*, *genome assembly*, *chromosomes*, *fish*

## Abstract

**Background:**

The teleost fish *Monopterus albus* is emerging as a new model for biological studies due to its natural sex transition and small genome, in addition to its enormous economic and potential medical value. However, no genomic information for the *Monopterus* is currently available.

**Findings:**

Here, we sequenced and *de novo* assembled the genome of *M. albus* and report the *de novo*chromosome assembly by FISH walking assisted by conserved synteny (Cafs). Using Cafs, 328 scaffolds were assembled into 12 chromosomes, which covered genomic sequences of 555 Mb, accounting for 81.3% of the sequences assembled in scaffolds (∼689 Mb). A total of 18 ,660 genes were mapped on the chromosomes and showed a nonrandom distribution along chromosomes.

**Conclusions:**

We report the first reference genome of the *Monopterus* and provide an efficient Cafs strategy for a *de novo* chromosome-level assembly of the *Monopterus* genome, which provides a valuable resource, not only for further studies in genetics, evolution, and development, particularly sex determination, but also for breed improvement of the species.

## Data Description

### Background

The freshwater fish *Monopterus albus* taxonomically belongs to the teleost family Synbranchidae of the order Synbranchiformes. This fish is distributed mainly in southern and eastern Asia, in northern Australia, and in the southeastern United States [1]. *Monopterus* is an economically important species because of its high nutritional value (e.g., high polyunsaturated fatty acid omega-6 levels) and potential medical value. The most influential Chinese pharmacy monograph, the Bencao Gangmu, a compendium of materia medica written by the pharmacist Shi-Zhen Li during the Ming Dynasty (AD 1368∼AD 1644), recommended *Monopterus* as a natural drug with medicinal virtues.

As an emerging model species in development, genetics, and evolution [2], *Monopterus* has the attractive feature of undergoing a sex transition from female to intersex to male during its life [3]. This discovery may have considerable theoretical significance in sex determination [4]. *Monopterus* has a small genome size (∼800 Mb) and a minimum chromosome number (n = 12) among teleosts, whose chromosome numbers range from 12 to 223 [5]. In addition, all chromosomes of *Monopterus* are telocentric. Given that a third whole-genome duplication occurred in the whole teleost lineage compared to the two genome duplications that occurred in other land vertebrates [6–8], the speciation and sexual differentiation of *Monopterus* may provide new insights into vertebrate evolution. However, the mechanisms of sex determination in the species remain unknown.

Whole-genome sequencing will provide detailed genetic data for studies of genetics, development, and evolution and for the genetic manipulation of *Monopterus*. However, no genetic map is currently available for this species. The whole-genome shotgun approach, with high throughput and low cost, is based on a second-generation sequencing platform that makes the whole-genome *de novo* assembly of a species possible without the need for a physical map. However, the sequence data produced by second-generation sequencing technologies are highly fragmented due to the short lengths of the reads. A number of methods for increasing the contiguity and accuracy of *de novo* assemblies have recently been developed. The read length generated from sequencing can be improved by a third-generation sequencing platform, such as single-molecule real-time sequencing, with raw reads of a mean length of 15 kb [9], and nanopore single-molecular sequencing, with raw reads of approximately 5–50 kb [10]. Some strategies for the assembly of a long scaffold have also been developed, e.g., BAC/fosmid paired end sequencing, the long-read sequencing (LRseq) [11] approach, contiguity-preserving transposase sequencing (*fragScaff*) [12], and various assembly algorithms [13, 14]. Recently, chromatin interactions, such as high-throughput/resolution chromosome conformation capture, have been used to assemble ultra-long scaffolds that can lead to a chromosome-scale assembly; however, a certain amount of error occurs when used for *de novo* assembly [14, 15]. Thus, accurate chromosome-level assembly remains a major challenge.

The most widely used strategy for chromosome-level assembly of the scaffolds generated by second-generation sequencing is based on a high-density genetic map at the chromosome level. Nevertheless, this strategy is feasible only when high-density genetic maps of a species are available. Because there is no genetic map available for *Monopterus*, we have developed an efficient assembly strategy: *de novo*chromosome assembly by FISH walking assisted by conserved synteny (Cafs). Using Cafs technology, which is efficient and cost effective, a precise chromosome-level assembly covering 81.3% of the sequences assembled in scaffolds was produced.

### Whole-genome sequencing

A whole-genome shotgun strategy and second-generation sequencing technology (Illumina HiSeq 2000 platform) were used to sequence two male *Monopterus*. Genomic DNA was extracted from eels from the Wuhan area in the Yangtze River basin. To reduce the risk of nonrandom sequencing, eight paired-end sequencing libraries with insert sizes of 170 bp, 500 bp, 800 bp, 2 kb, 5 kb, 10 kb, 20 kb, and 40 kb were constructed. Of them, the library with 40 kb inserts was from a second individual for assembling of long scaffolds. These libraries generated 101.62 GB of sequence data. To reduce sequencing errors in the assembly, sequence reads were filtered to remove low-quality reads. After filtering, 78.64 GB (97.6X) of sequence data were retained for the assembly, which ensures a high single-base accuracy (Additional files: Fig. S1 and Table S1).

### Estimation of genome size

A k-mer was defined as a sequence of k bases in length. The frequency of k-mers in a collection of short, insert-sized reads could be calculated with a 1 bp sliding window. When an optimal amount of data was present, the k-mer frequency followed a Poisson distribution. The k-mer value was used to estimate the genome size, as follows: genome size = K_num/Peak_depth, where K_num is the total number of k-mers and Peak_depth is the expected value of the k-mer depth [16]. The 17-mer distribution obeyed the theoretical Poisson distribution. The data used for k-mer analysis were derived from the male that was used to construct the genome sequencing libraries. The heterozygosity revealed from the k-mer analysis reflects the inner heterozygosity in an individual. Finally, we observed that the proportion of heterozygosity in the *Monopterus* genome was small, and estimated that the entire genome comprised 806 Mb, with a GC (guanine-cytosine) content of 40.8% (Additional files: Figs. S2, S3, and Table S2).

### *De novo* genome assembly

The *Monopterus* genome was *de novo* assembled with the SOAPdenovo software [16]. SOAPdenovo employs the de Bruijn graph algorithm to simplify assembly and reduce computational complexity. Low-quality reads were filtered out, and potential sequencing errors were removed or corrected with the k-mer frequency methodology. The SOAPdenovo assembly process consisted of three main steps: contig construction, scaffold construction, and gap filling. The sequence data derived from the libraries with insert size of 2 kb, 5 kb, 10 kb, and 20 kb were used to assemble the scaffolds by SOAPdenovo. The sequence data derived from the library with 40 kb insert were used to build scaffolds with SSPACE version 1.1 software [17].

To assess assembly quality and completeness, high quality reads from short-insert-size libraries (75 bp read lengths) were aligned to the assembly with the BWA program [18] (version 0.5.9-r16), with default parameters. Next, SOAPcoverage (version 2.27) was used to calculate sequencing depth. A total of 91.06% reads could be mapped, and they covered 99.69% of the assembly, excluding gaps. To further test for possible contigs that might be mis-joined in scaffolds, we analyzed paired-end information. We found that, if contigs were included only when both ends could be uniquely mapped onto the assembly, 90.66% of paired-ends were in the correct orientation and at the expected distance, according to the utilized short-insert-size libraries (Additional file: Tables S3).

The final assembly comprised 689.5 Mb with contig and scaffold N50 sizes of 22.2 kb and 2.1 Mb, respectively (Table 1). More than 90% of the total sequence was covered by 379 scaffolds; the longest scaffold spanned 11.7 Mb (Table 1). Assembly accuracy was further demonstrated by 91.06% reads mapping (99.69% coverage) to the reference sequences of the genome and the successful mapping of 321 bacterial artificial chromosomes (BACs) sequenced with Sanger sequencing technology.

**Table 1:** Statistics of the assembly of the *Monopterus* genome

	Contigs*		Scaffolds	
	Size (bp)	Number	Size (bp)	Number
N90	4,762	33 ,115	368 ,242	379
N80	8,655	23, 414	775 ,515	254
N70	12, 290	17, 275	1,109 ,624	180
N60	16 ,188	12 ,785	1,519 ,751	128
N50	22 ,239	8,438	2,106 ,322	87
Longest	159 ,913	—-	11, 676, 616	—-
Total size	634 ,655 ,961	—-	689 ,524 ,511	—-
Total number(≥100 bp)	—-	117, 579	—-	62 ,978
Total number (≥2 kb)	—-	44 ,314	—-	2,360

* The contig size was the final size after filling intrascaffold gaps. Contigs with lengths shorter than 100 bp were not included in the statistics.

To evaluate the quality of the assembled genome, we conducted Benchmarking Universal Single-Copy Orthologs analysis [19] using BUSCO v2.0 with vertebrata_odb9 including 2,586 BUSCOs. Using the BUSCO analysis, 96.5% of BUSCOs were completely detected in the assembled genome (2,464: complete and single-copy, 32: complete and duplicated) among 2,586 tested BUSCOs. The number of fragmented and missing BUSCOs was 56 and 34, respectively. Together, the genome of the *Monopterus* assembled is of high quality.

### Repeat elements

Transposable elements were identified in the genome with a combination of homology-based and de novo approaches. The homology-based approach utilized database Repbase [20] (release 19.06), with RepeatMasker (RepeatMasker, Version 4.0.3) and RepeatProteinMask (from the RepeatMasker package) programs with the default parameters [20]. The *de novo*approach used two prediction programs, RepeatModeler [21] (version 1.0.7) and LTR-FINDER [22] (version 1.0.5), to build the *de novo* repeat libraries based on the genome sequences. Next, contaminations and multicopy genes were removed from the libraries. Then, the RepeatMasker was used again to find repeats in these repetitive sequence libraries. Finally, we combined all the results generated by these methods. To improve our comparisons to other teleost fish, we used the same procedure and parameters to analyze the *Danio rerio*, *Oryzias latipes*, *Gasterosteus aculeatus*, *Tetraodon nigroviridis*, and *Takifugu rubripes* genomes.

For the assembled sequence, the repetitive element content of the *Monopterus* genome (28%) was much lower than that of the zebrafish (61%) and about the same as medaka (29%) genomes but higher than that of the threespine stickleback (16%) and pufferfish (8–10%) genomes (Additional file: Fig. S4). In the *Monopterus* genome, the main repetitive transposable elements were the DNA transposons and long interspersed nuclear elements (LINEs). At 8%, the LINEs were the largest category of transposable elements. The percent of LINEs was greater than that found in other teleost fish (2–5%), which might be associated with genome instability [23].

It should be pointed out that only ∼79% of the expected genome size was captured in contigs (634.7 Mb of 806 Mb), and the BUSCO analysis showed that the 634.7 Mb genome assembly was complete. These data suggest that the unassembled genome probably consists of noncoding DNA, possibly containing many repeats. Thus, the repeat abundance would likely be underestimated. Third-generation sequencing will provide a more complete assembly.

### Genes and function annotation

We used both homology-based and *de novo* methods to predict genes in the *Monopterus* genome by scanning the local *Monopterus* genome database, which also included RNA-seq data. For the homology-based prediction, protein sequences from *D. rerio*, *O. latipes*, *G. aculeatus*, *T. nigroviridis*, and *T. rubripes* were downloaded from the Ensemble platform [24] (release 75) and aligned with the *Monopterus* genome with the Tblastn program [25]. Accordingly, homologous genomic sequences were input into the Genewise program [26] to align matching proteins. This procedure allowed us to define gene structures. For *de novo* prediction, both the Fgenesh [27] and Genscan [28] programs were used to predict coding genes with the appropriate parameters. Homology-based and *de novo*- derived gene sets were combined with comprehensive, nonredundant reference gene sets, obtained with the GLEAN platform [29]. Genes were corrected by comparisons with the RNA-seq data; these RNA-seqs were mapped to the *Monopterus* genome with the Tophat program, and the Cufflinks program (version cufflinks-2.2.1) [30] was used to assemble transcripts. After that, we selected 1,000 intact genes, defined as gene set “A,” that were supported by the homology-based prediction, and passing a fifth-order Markov model, to verify the ORFs of RNA transcripts based on the hidden Markov model. In the *Monopterus*,  24,056 protein-coding genes were predicted (Additional file: Table S4-S6). The average gene sizes were similar to those of other teleost fish (Additional files: Fig. S5 and Table S5).

Blastp was used to search for proteins encoded in the *Monopterus* genome by comparing candidate sequences against the SwissProt and TrEMBL databases from the UniProt Knowledgebase (UniProtKB) [31]. The annotated motifs and domains in the available databases (ProDom, PRINTS, Pfam, SMART, PANTHER, and PROSITE) were obtained with the InterProScan program [32] (version 4.7). In gene ontology (GO) [33] analyses, gene functions were obtained from the corresponding InterPro entries. Subsets of the GO terms were obtained according to the program DAVID (version 6.7) [34]. X-associated genes were annotated based on the GO term list in human, and Z-associated genes were annotated based on the GO term list in chicken. All genes were also aligned against the Kyoto Encyclopedia of Genes and Genomes (KEGG) [35] (release 68) protein database. The genes that matched genes in the KEGG database were assumed to be involved in the corresponding signaling pathways. Approximately 80% of the genes could be functionally annotated with homology analysis (Additional file: Fig. S6).

### *De novo* chromosome assembly by Cafs-strategy

**Figure 1:**
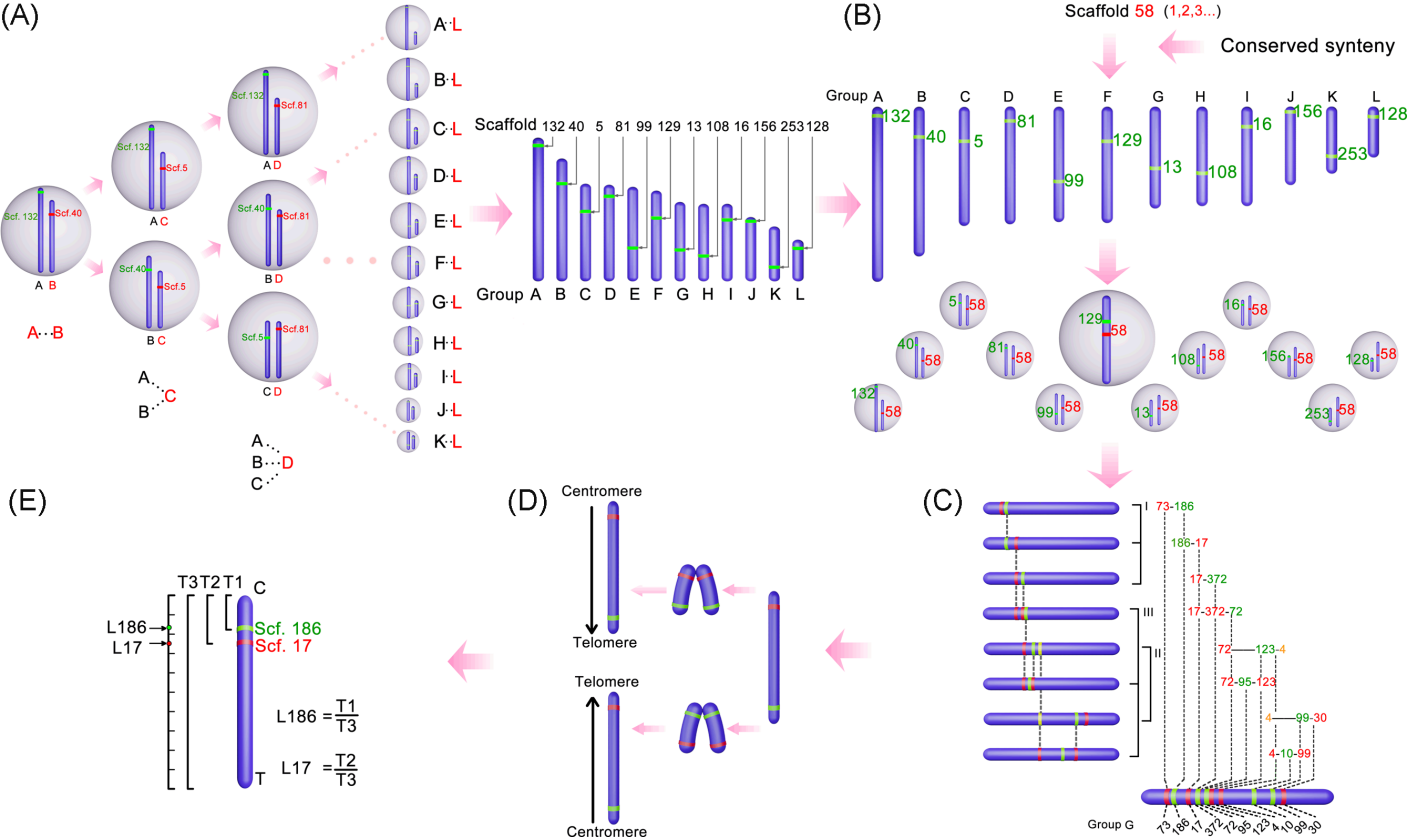
Overview of *de novo* chromosome assembly by FISH walking assisted by conserved synteny. (A) Identification of 12 linkage groups by probe combination mapping. FISH probes are hybridized on pachytene chromosomes. Red and green dots indicate scaffold locations. (B) Synteny-assisted scaffold mapping. Each candidate BAC (scaffold) is cohybridized with 12 landmarks by dual-color FISH, respectively. Synteny-supported/nonsupported scaffolds are determined by FISH. (C) Determination of scaffold order on chromosome by FISH walking. (I) The order of two scaffolds is identified by dual-color FISH if both of them are on one side of the chromosome. (II) If the scaffolds are in the center of the chromosome, three-color FISH is applied to determine their order. (III) The order of some scaffolds (labeled with one color) could be identified by three signals dual-color FISH, when their two neighboring scaffolds (labeled with another color) have been determined. (D) Identification of orientation of linkage groups on metaphase chromosomes. Telomeres and centromeres can be observed on the metaphase chromosomes. (E) Localization of scaffolds is determined by calculating the corresponding distances to the centromere.

To assemble chromosomes with accurate sequences from the scaffolds, we developed an efficient assembly strategy without using any genetic map information, Cafs (Fig. 1), which is based on chromosome fluorescent *in situ* hybridization (FISH) and the shared synteny between distantly related fish species.

We first prepared probes of BACs from sequenced clones and PCR (polymerase chain reaction) fragment pools representing scaffolds for chromosome FISH and performed synteny analysis of these scaffolds by comparing with the fish species medaka, sticklebacks, *Tetraodon*, and *Monopterus*. Second, from the synteny information of the homologous sequences of these scaffolds in the three fish species, probe combination mapping was used to determine 12 linkage groups corresponding to 12 chromosomes, each with a molecular landmark (Fig. 1A). Briefly, group A and B were first discriminated by two unlinked scaffolds labeled with two different colors. If another scaffold was unlinked to the previous two scaffolds, the third scaffold was identified as a marker of group C. Third, based on the predicted syntenic relationship between the related fish species, probes for the candidate scaffolds were cohybridized with the landmarks of the chromosomes, which have been identified. Scaffolds with no predicted location and that were inconsistent with the predicted location were further determined by cohybridization with 12 landmarks using dual-color FISH, respectively (Fig. 1B). For example, scaffold 58 would be grouped into the F group, as it is linked with scaffold 129, which was the landmark of the F group. Fourth, the loose and long pachytene chromosomes were adopted to determine the location and order relationship of the scaffolds through dual- and three-color FISH. An original marker was used as a walking start (e.g., scaffold 73), and the location of the second scaffold (e.g., scaffold 186) relative to the original marker was identified by dual-color FISH. The location of a new scaffold was determined by the known scaffold locations using dual- or three-color FISH (e.g., scaffolds 72, 123, and 4) (Fig. 1C). Finally, because all 12 chromosomes are telocentric, the telomeres of the metaphase chromosome were used as landmarks to determine the directions of the mapped scaffolds on the chromosomes (Fig. 1D). The relative position of all scaffolds on chromosomes was determined by the measurement of the signals to the centromere (Fig. 1E). The distance values were measured by Image-Pro Plus 6.0, and each value was obtained from an average of more than five cells.

Using the Cafs assembly strategy, we conducted large-scale mapping of the scaffolds on each chromosome. Metaphase chromosomes were prepared according to routine protocols from the *Monopterus* kidney tissue [5]. Meiotic pachytene bivalents were prepared from *Monopterus* testis using a previously described method [36]. The FISH was conducted as previously described [37]. The BAC end sequences were aligned to the genome database by Blat (version blat_34) [38]. BACs with two ends aligned to one scaffold and those ends with sequences with homology to scaffolds greater than 90% were used as probes for FISH. Of the ∼747 sequenced clones, 148 BACs could be used as probes for FISH (Additional file: Table S7). The BACs were confirmed by PCR sequencing from the internal regions of the BACs. A total of 148 BACs and 38 pools of PCR fragments (Additional file: Table S8) (8–15 sequences covering a total length of 20–30 kb on a scaffold) representing 186 scaffolds were prepared as probes for chromosome FISH.

Before the hybridization experiment, we performed a genome-wide synteny analysis to compare these fish species. We constructed a reference map using the syntenic relationship among the genomes of medaka, stickleback, and *Tetraodon* to help map the scaffolds on the *Monopterus* chromosomes. The syntenic blocks between *Monopterus* and other fish were aligned by Lastz (Blastz) [39] with parameters of T = 2 and Y = 3400. Furthermore, we used Blat to search for homologous sequences among medaka, sticklebacks, *Tetraodon*, and *Monopterus* in order to fill the gap sequences of blocks in the reference map. If two homologous sequences were linked in all three species, we defined the corresponding scaffolds in *Monopterus* as predicted linked scaffolds.

**Figure 2:**
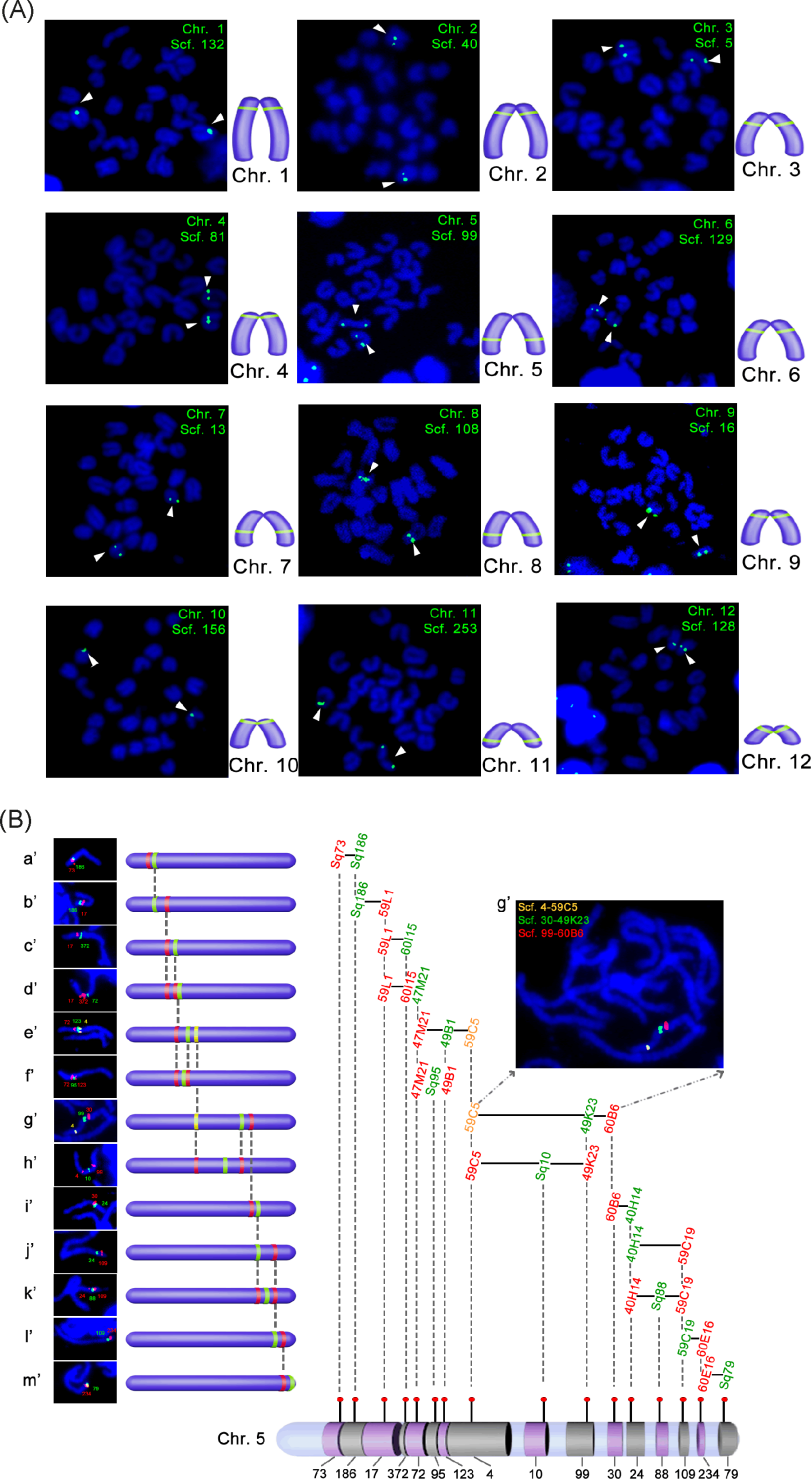
Chromosome assembly by Cafs. (A) FISH images show 12 molecular landmarks corresponding to 12 chromosomes. Green signals indicate the landmarks labeled by digoxigenin and detected with FITC (Fluorescein Isothiocyanate). Each chromosome is determined by a landmark. Chromosomes are stained with 49–6-diamidino-2-phenylindole (blue). (B) Localization of each scaffold on chromosome 5 by FISH walking strategy. FISH images and corresponding scaffold order from (a’) to (m’) are shown in the left panels. A three-color FISH image (g’) in the upper right indicates the relative order of scaffolds 4 (yellow, FITC+Cy3), 30 (green, FITC), and 99 (red, Cy3) on chromosome 5. Probes (red dots) and their locations on scaffolds are used to assemble chromosome 5.

**Figure 3:**
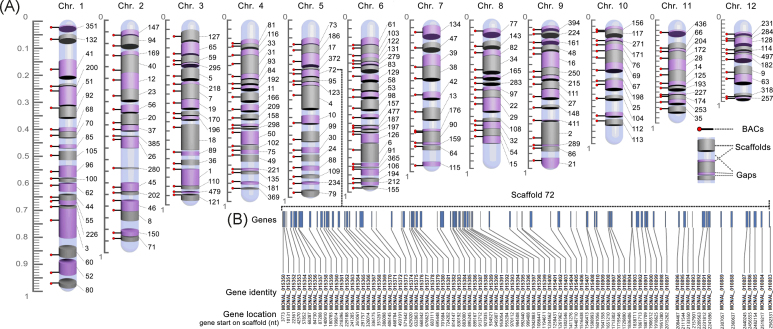
Chromosome-scale assembly of the *Monopterus* genome. (A) Each chromosome is assembled with scaffolds and their order from telomere (down end) to centromere (up end). The gray and purple cylinders represent the anchored scaffolds. The segments in light blue between two neighboring scaffolds indicate gaps. Sticks with a red head anchored on each scaffold indicate the positions of the BACs used as probes. Scale bar, 0–1. (B) Scaffold 72 with 87 genes (blue bars) and their location on chromosome 5 is highlighted.

Under the guidance of the synteny of the homologous sequence of these 186 scaffolds in the other three fish species, 78 probe combinations of cohybridization were performed to identify 12 linkage groups, each with a molecular landmark (Fig. 2A). We then conducted the walking in a range of 11–22 steps per chromosome (Fig. 2B; Additional file: Fig. S7). A total of 186 scaffolds were assembled into 12 pachytene chromosomes through step-by-step combination hybridization of the probes using the above-mentioned 148 BACs and 38 PCR fragment pools (Fig. 3A). We then determined the orientation of each chromosome by dual-color FISH on metaphase chromosomes using the telomere as a morphological landmark (Additional file: Fig. S8). Of these mapped scaffolds, 92% (99/108) were consistent with the shared synteny between the related fish species (medaka, sticklebacks, and *Tetraodon*). From the synteny analysis, an additional 142 scaffolds were predicted and further assembled into 12 chromosomes, respectively. Based on the results of the FISH experiments and collinearity analyses, we can expect approximately 8% placement errors among these 142 scaffolds. Accurate mapping of the 142 scaffolds remains to be confirmed by third-generation sequencing. The current assembly, particularly the locations of the 186 scaffolds, have great implications for comparative genomics and evolution studies. As the genome of *Monopterus* has the least number of chromosomes in the teleosts, the determination of 12 linkage groups of the *Monopterus* genome is crucial for the studies of large-scale chromosome recombination events (e.g., chromosome fusion or fission) in the teleost evolution.

We then integrated 328 scaffolds into the reference genome. These mapped scaffolds consisted of 455 Mb determined by FISH and 100 Mb determined by syntenic analysis, which covers genomic sequences of a total length of 555 Mb, accounting for 81.3% of the sequences assembled in scaffolds (689.5 Mb). Based on the assembly, a total of 18, 660 protein-coding genes were annotated with location information on the chromosomes (Table 2). For example, there are 87 protein-coding genes on scaffold 72, which was located on chromosome 5 (Fig. 3B). These data indicate that a *de novo* chromosome-level assembly of the *Monopterus* genome was produced using the Cafs strategy.

**Table 2:** Assembly statistics for each chromosome

Chromosome	Chromosome size (kb)	Scaffold No.	Gene No.	Gene density (n/10Mb)
1	75 ,908.7	33	2,264	298
2	65,103.9	32	2,133	328
3	51,637.3	21	1,872	363
4	51,162.1	30	1,791	350
5	50,080.0	27	1,517	303
6	48,093.1	27	1,659	345
7	42,410.1	29	1,500	354
8	41,999.7	30	1,456	347
9	41,928.7	23	1,241	296
10	34,690.8	30	1,262	364
11	29,285.5	23	1,086	371
12	22,774.4	23	879	386
Total	555,074.3	328	18, 660	336

### Chromosome-wide gene clustering

**Figure 4:**
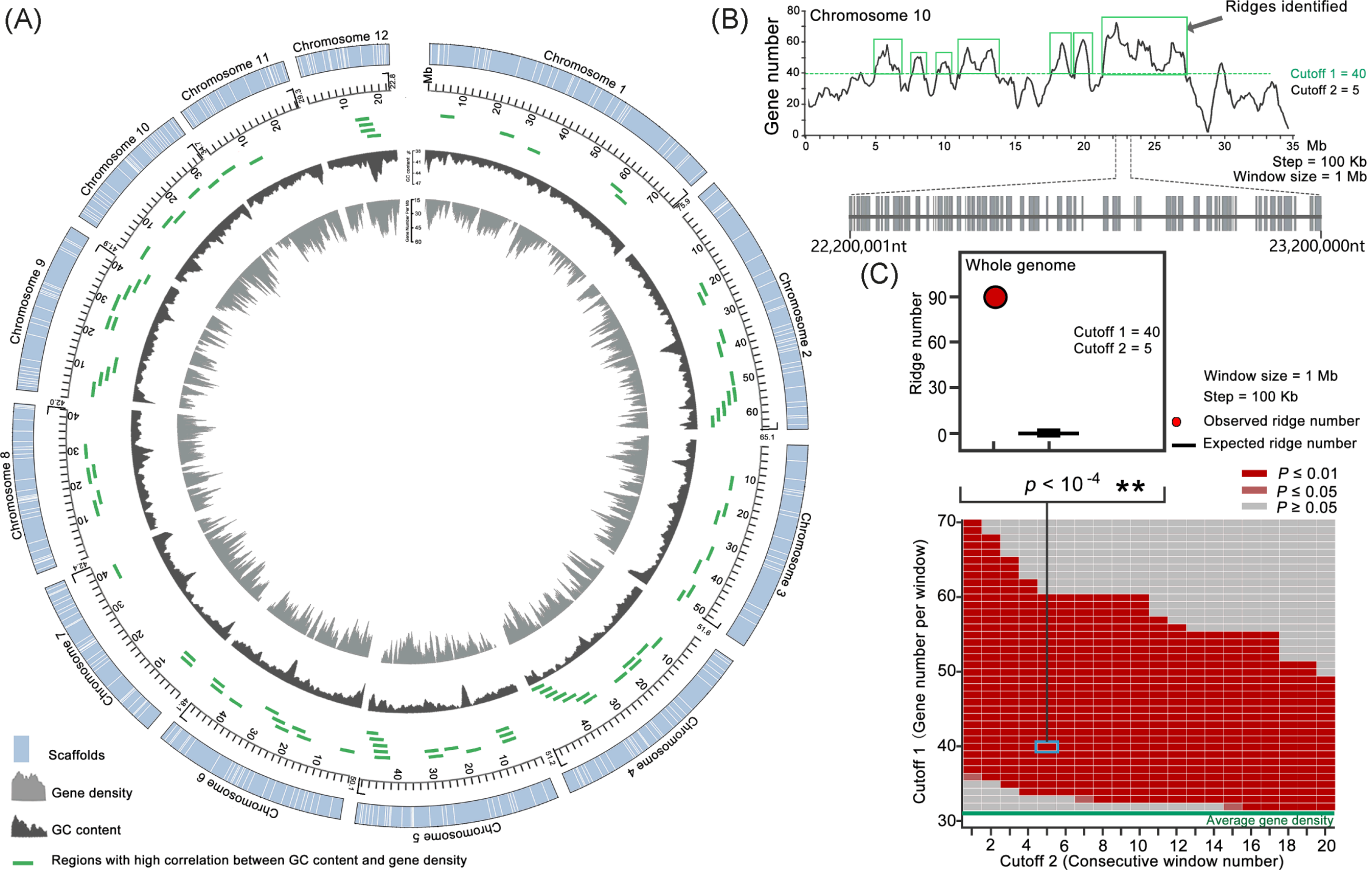
Chromosome-wide gene clustering. (A) Circos shows the assembled chromosomes, GC content, and gene density. The inner scale is 2 Mb. The strips in the outer circle indicate the scaffolds packaged into chromosomes, with each gap (see Fig. 3) replaced with 1 kb of Ns. The inner dark gray ridges show the moving GC percentage, and the inner gray ridges show the moving number of the genes at a window size of 1 Mb. The green sticks indicate the location of the regions with high correlation between GC content with gene density (R > 0.95, *P*-value < 0.01). (B) Distribution of gene clusters (ridges) on chromosome 10. Curves indicate the moving numbers of genes at a window size of 1 Mb (step = 100 kb). The windows with a maximum gene density from nt 22, 200, 001 to 23, 200, 000 on chromosome 10, which contains 71 genes, are shown in the lower panel. Green boxes highlight ridges in which there are at least five consecutive moving windows with a lower limit of 40 genes per window. (C) Statistical tests of numbers of gene density ridges in the genome corresponding to background noise (null model). The heat map in the lower panel shows *P*-values in the significance test of observed ridge numbers against the null model ( 10,000 independent permutations of gene positions). The *x*-axis indicates the cutoff values of numbers of consecutive moving windows, which reflects the extent of the clustering. The *y*-axis indicates the cutoff values of gene numbers within a certain window size (step 100 kb), which reflects the degree of intensity of the clusters. Green lines represent the average gene number in a certain window size. The upper panel highlights a significance test at the condition of two cutoff values, gene density (40/Mb) and consecutive window numbers (5). Red dots represent the number of observed ridges in the genome. Box plots (black) represent distribution of the ridge numbers in  10,000 independent permutations of gene positions in a random fashion.

To further investigate gene clustering along the chromosomes, we calculated the gene density per chromosome. The average gene density in the genome was 33.6 genes per Mb, with the maximum gene density on chromosome 12, which is the shortest chromosome; the minimum gene density was on chromosome 9 (Table 2). Further sliding window analysis showed that there was also biased distribution of the gene density within the chromosome (Fig. 4A). Using a 1-Mb window size and 100-kb step size, the maximum gene density in the genome was detected from nt 22, 200, 001 to nt 23, 200, 000 on chromosome 10, which contains 71 genes, in comparison with an average of 33.6 genes per Mb in the genome (Fig. 4B).

We then tested the statistical significance of pairing correlations between gene density and GC content. As these parameters are not normally distributed, we used the nonparametric Spearman correlation test on the ranks of the paired quantities. Correlation analyses were performed with R software [40]. The R package ggplot2 was used to draw scatter plots and box plots. The distribution pattern of the gene density was consistent with the corresponding GC content along the chromosomes (Fig. 4A; Additional file: Fig. S9A). In order to see whether particular regions contribute to the positive correlation, we used the sliding window analysis to divide the genome into 4-Mb small regions and calculated the correlation coefficient between GC content and gene density in each 4 Mb-region. Using a 3-Mb window size and 100-kb step size, we divided the genomes into 5,196 regions each with a size of 4 Mb. The analysis showed that there is a large proportion (68.21%) of 4-Mb regions with correlations lower than 0.7 (Additional file: Fig. S9B). Some regions with very high correlation coefficients (R >0.95, *P*-value <0.01) are detected in the genome, e.g., four regions on chromosome 12 (Fig. 4A). In this analysis, Circos (version 0.69) [41] was used to plot the assembled chromosomes, GC content, and gene density.

To investigate whether the distribution of the genes along the chromosomes is nonrandom, we computed the probability of the events (ridge numbers under a random permutation of the gene positions greater than or equal to actual numbers of ridges) following a method previously described [42]. A ridge was used to describe a chromosome region with high gene density, which is thus defined as at least W consecutive windows, each containing a gene number higher than H. Thus, the ridge is determined by two parameters: cutoff 1 (C_H_), gene number per window, and cutoff 2 (C_W_), number of consecutive windows. The actual ridge numbers (N) in the genome were calculated under C_H_ and C_W_ by sliding window analysis. We used the following calculation parameters to set up a null model: suppose we have a random permutation of X_1_, X_2_, …, X_i_ in the range of 1 to S; i, gene number on the chromosome; S, length of the chromosome; and X_1_, X_2_, …, X_i_, gene locations on the chromosome. With the same cutoff values under actual conditions (C_H_ and C_W_), we can obtain a ridge number (n) under the null model. We can compute the frequency (f) when n ≥ N by permutation  10,000 times. If f = 0, the *P*-value is <10^−4^, or the *P*-value is =  f/10,000. For all of the cutoff C_H_ and C_W_ combinations, we calculated the *P*-value under different window sizes of 0.2, 0.3, 0.5, 1, 2, and 3 Mb, respectively.

Using a combination of the two cutoffs, the number of ridges of each chromosome can be identified. For example, using cutoffs of 40 genes per Mb and 5 consecutive windows, 7 ridges on chromosome 10 were identified (Fig. 4B), and 90 ridges were identified in the genome (Fig. 4C). The probability of the observed ridges occurring in random permutations of gene positions was very low (*P*-value <10^−4^) (Fig. 4C), confirming nonrandom and clustering distribution of genes along the chromosomes. Probabilities (ridges numbers under a random permutation greater than or equal to ridges numbers in the *Monopterus* genome) for a series of cutoff sets and different window sizes were also calculated. The results showed that there were significant differences in ridge numbers between the *Monopterus* genome and random permutations of gene positions (Fig. 4C; Additional file: Fig. S10). The ridge numbers of high gene density directly reflect the clustering of genes along the chromosomes. These analyses suggest that the ridge pattern on the chromosomes probably represents a higher-order structure in the genome.

### Re-use potential

In summary, here we report the first genome to be sequenced and assembled in the order Synbranchiformes in freshwater fish. Because *Monopterus* is not only an economically important freshwater fish in aquacultural production but also an increasingly known model species for biological studies, the assembled genome will provide valuable information for genetic improvement of economical traits by hunting key genes/QTLs in the species; for understanding molecular mechanisms underlying sex reversal; for genome evolution studies by comparative genomics among *Monopterus*, other fish, and the species in land; and for speciation and ecological conservation researchers by dissecting aerial respiration ability of the species, which is a crucial feature in the origin of the tetrapods from the sea up to the land. We also expect that the genome data will be used by other researchers for functional genomics, e.g., gene knockout using genes provided here through the clustered regularly interspaced short palindromic repeats (CRISPR)/CRISPR-associated protein 9 approach, and also making transgenic fish using the genes and assembly represented in this study. In addition, the assembled genome provides a reference for further complete and accurate assembly/annotation as gaps/assembly errors exist in the version of assembly using second-generation sequencing technology.

## Availability of supporting data

The genome data from this study have been deposited at DDBJ/EMBL/GenBank (accession number AONE00000000), and the raw transcriptome data have been submitted to the National Center for Biotechnology Information Gene Expression Omnibus (accession number GSE43649). Datasets further supporting the manuscript, including BUSCO results, annotations, and perl scripts, are available in the *GigaScience* database, GigaDB [43].

## Additional files

**Supplemental Figure S1:** Sequencing depth distribution of the *Monopterus* genome.

**Supplemental Figure S2:** Genome size estimation using 17-mer.

**Supplemental Figure S3:** The GC distribution of the *Monopterus* genome.

**Supplemental Figure S4:** Divergence distribution of the classified transposable elements.

**Supplemental Figure S5:** Comparisons of predicted coding genes of *Monopterus* with other teleost fish.

**Supplemental Figure S6:** The Gene Ontology of the *Monopterus* genes.

**Supplemental Figure S7:** Localization of each scaffold on chromosomes by FISH-walking strategy.

**Supplemental Figure S8:** Orientation of each linkage group on metaphase chromosomes.

**Supplemental Figure S9:** Correlation coefficient of GC content with gene density.

**Supplemental Figure S10:** Statistical tests of numbers of gene density ridges in the genome corresponding to background noise (null model) in different window sizes (0.2, 0.3, 0.5, 1, 2, 3 Mb).

**Supplemental Table S1:** Statistics of sequencing.

**Supplemental Table S2:** Statistics of genome from 17-mer.

**Supplemental Table S3:** Statistics of mapping.

**Supplemental Table S4:** Statistics of predicted coding genes.

**Supplemental Table S5:** Comparisons of predicted coding genes of *Monopterus* with other teleost fish.

**Supplemental Table S6:** Annotated classification of the *Monopterus* genes.

**Supplemental Table S7:** Alignments of BAC ends to reference genome.

**Supplemental Table S8:** Information of FISH probes synthesized by PCR.

## Abbreviations

BAC: bacterial artificial chromosome; bp: base pair; BUSCO: Benchmarking Universal Single-Copy Orthologs; Cafs: chromosome assembly by FISH walking assisted by conserved synteny; CRISPR: clustered regularly interspaced short palindromic repeats; FISH: fluorescent *in situ* hybridization; GC: guanine-cytosine; GO: gene ontology; KEGG: Kyoto Encyclopedia of Genes and Genomes; LINEs: long interspersed nuclear elements; PCR: polymerase chain reaction.

## Ethics statement

*Monopterus* were obtained from Hubei, China. All animal experiments and methods were performed in accordance with the relevant approved guidelines and regulations, as well as under the approval of the Ethics Committee of Wuhan University.

## Supplementary Material

GIGA-D-17-00210_Original_Submission.pdf

GIGA-D-17-00210_Revision_1.pdf

GIGA-D-17-00210_Revision_2.pdf

GIGA-D-17-00210_Revision_3.pdf

Response_to_Reviewer_Comments_Original_Submission.pdf

Response_to_Reviewer_Comments_Revision_1.pdf

Response_to_Reviewer_Comments_Revision_2.pdf

Reviewer_1_Report_(Original_Submission) -- Matthias Platzer 10/1/2017 Reviewed

Reviewer_2_(Original_Submission)_Attachment-swamp_eel_plots.pdf

Reviewer_2_Report_(Original_Submission) -- Christiaan Henkel 11/20/2017 Reviewed

Reviewer_2_Report_(Revision_1) -- Christiaan Henkel 2/5/2018 Reviewed

Reviewer_2_Report_(Revision_2) -- Christiaan Henkel 3/21/2018 Reviewed

Additional Files
