# Supplementary material for: Chromosome-scale assembly of the Monopterus genome
Source: Gigascience. 2018 Apr 24;7(5):giy046. doi: 10.1093/gigascience/giy046 (PMC5946948; doi:10.1093/gigascience/giy046)
Supplement: GIGA-D-17-00210_Original_Submission.pdf [file giy046_giga-d-17-00210_original_submission.pdf]

GigaScience

Chromosome-scale assembly of the Monopterus genome reveals a co-regulation landscape of interconvertible regions of sex during sex transition

--Manuscript Draft--

Manuscript Number:	GIGA-D-17-00210
Full Title:	Chromosome-scale assembly of the Monopterus genome reveals a co-regulation landscape of interconvertible regions of sex during sex transition
Article Type:	Research
Funding Information:	National Natural Science Foundation of China (31571280) — Professor Rongjia Zhou<br>National Natural Science Foundation of China (31471182) — Prof. Hanhua Cheng
Abstract:	Background: The teleost fish Monopterus albus is emerging as a new model for biological studies due to its natural sex transition and small genome, in addition to its enormous economic and medical value. However, the mechanisms of sex determination in the species remain unknown.<br><br>Results: Here, we sequenced and de novo assembled whole genome of the Monopterus, and report the de novo chromosome assembly by FISH walking assisted by conserved synteny (Cafs) for Monopterus. Using Cafs, 328 scaffolds were assembled into 12 chromosomes, which cover genomic sequences of 555 Mb, accounting for 81.3% of the sequenced genome (~689 Mb). A total of 18,860 genes were mapped on the chromosomes and showed a non-random distribution along chromosomes. Genome-wide analysis together with transcriptomes of the three stages of gonadal development showed large-scale clustering of the co-regulated genes associated with gonad transition. Moreover, two types of genes with opposite expression modes were expressed in a coordinated, alternating, mutually interconvertible manner during gonad transition and tended to be chromosomally clustered separately. Remarkably, we discovered a co-regulation mechanism of the interconvertible regions of sex-associated expression (IRSEs) during sex transition. A distinct feature of the IRSEs is the exponential distribution of their sizes with an enrichment of ~0.5 Mb on chromosomes.<br><br>Conclusions: A precise chromosome-level assembly was produced. Genome-wide analysis together with transcriptomes of gonadal differentiation showed a co-regulation mechanism of the interconvertible regions of sex-associated expression during sex transition. The IRSEs probably represent a new type of combinatorial regulation of gene expression in a higher chromatin structure, suggesting a novel mechanism for sex determination at both the transcriptional and chromatin organization levels.
Corresponding Author:	Rongjia Zhou, Ph.D.<br>Wuhan University<br>Wuhan, Hubei CHINA
Corresponding Author Secondary Information:	
Corresponding Author's Institution:	Wuhan University
Corresponding Author's Secondary Institution:	
First Author:	Xueya Zhao
First Author Secondary Information:	
Order of Authors:	Xueya Zhao
	Majing Luo
	Zhigang Li

Powered by Editorial Manager® and ProduXion Manager® from Aries Systems Corporation

	Pei Zhong
	Yibin Cheng
	Jiumeng Min
	Mingzhou Bai
	Yulan Yang
	Hanhua Cheng
	Rongjia Zhou, Ph.D.
**Order of Authors Secondary Information:**	
**Opposed Reviewers:**	
**Additional Information:**	
**Question**	**Response**
Are you submitting this manuscript to a special series or article collection?	No
**Experimental design and statistics**<br><br>Full details of the experimental design and statistical methods used should be given in the Methods section, as detailed in our Minimum Standards Reporting Checklist. Information essential to interpreting the data presented should be made available in the figure legends.<br><br>Have you included all the information requested in your manuscript?	Yes
**Resources**<br><br>A description of all resources used, including antibodies, cell lines, animals and software tools, with enough information to allow them to be uniquely identified, should be included in the Methods section. Authors are strongly encouraged to cite Research Resource Identifiers (RRIDs) for antibodies, model organisms and tools, where possible.<br><br>Have you included the information requested as detailed in our Minimum Standards Reporting Checklist?	Yes
**Availability of data and materials**<br><br>All datasets and code on which the conclusions of the paper rely must be either included in your submission or deposited in publicly available repositories	Yes

Powered by Editorial Manager® and ProduXion Manager® from Aries Systems Corporation

(where available and ethically appropriate), referencing such data using a unique identifier in the references and in the "Availability of Data and Materials" section of your manuscript.<br><br>Have you have met the above requirement as detailed in our Minimum Standards Reporting Checklist?	

Manuscript Click here to download Manuscript MS.docx

Chromosome-scale assembly of the *Monopterus* genome reveals a co-regulation landscape of interconvertible regions of sex during sex transition

Running title: Chromosome-level assembly and sex transition

Xueya Zhao[1], Majing Luo[1], Zhigang Li[1], Pei Zhong[1], Yibin Cheng[1], Jiumeng Min[2], Mingzhou Bai[2], Yulan Yang[2], Hanhua Cheng[1]*, Rongjia Zhou[1]*

[1]Hubei Key Laboratory of Cell Homeostasis, Laboratory of Molecular and Developmental Genetics, College of Life Sciences, Wuhan University, Wuhan 430072, P. R. China

[2]BGI-Shenzhen, Shenzhen 518083, P. R. China

*Corresponding authors: Professors Rongjia Zhou and Hanhua Cheng, College of Life Sciences, Wuhan University, Wuhan 430072, P. R. China, Fax: 0086-27-68756253, E-mail: rjzhou@whu.edu.cn, hhcheng@whu.edu.cn

Abstract

Background: The teleost fish *Monopterus albus* is emerging as a new model for biological studies due to its natural sex transition and small genome, in addition to its enormous economic and medical value. However, the mechanisms of sex determination in the species remain unknown.

Results: Here, we sequenced and de novo assembled whole genome of the *Monopterus*, and report the *de novo* chromosome assembly by FISH walking assisted by conserved synteny (Cafs) for *Monopterus*. Using Cafs, 328 scaffolds were assembled into 12 chromosomes, which cover genomic sequences of 555 Mb, accounting for 81.3% of the sequenced genome (~689 Mb). A total of 18,860 genes were mapped on the chromosomes and showed a non-random distribution along chromosomes. Genome-wide analysis together with transcriptomes of the three stages of gonadal development showed large-scale clustering of the co-regulated genes associated with gonad transition. Moreover, two types of genes with opposite expression modes were expressed in a coordinated, alternating, mutually interconvertible manner during gonad transition and tended to be chromosomally clustered separately. Remarkably, we discovered a co-regulation mechanism of the interconvertible regions of sex-associated expression (IRSEs) during sex transition. A distinct feature of the IRSEs is the exponential distribution of their sizes with an enrichment of ~0.5 Mb on chromosomes.

Conclusions: A precise chromosome-level assembly was produced. Genome-wide analysis together with transcriptomes of gonadal differentiation showed a co-regulation mechanism of the interconvertible regions of sex-associated expression during sex transition. The IRSEs probably represent a new type of combinatorial regulation of gene expression in a higher chromatin structure, suggesting a novel mechanism for sex determination at both the transcriptional and chromatin organization levels.

Key words: genome assembly, chromosomes, co-regulation, sex determination, fish

Background

The freshwater fish *Monopterus albus* taxonomically belongs to the teleost family Synbranchidae of the order Synbranchiformes. This fish is distributed mainly in southern and eastern Asia, in northern Australia and in the southeastern United States [1]. *Monopterus* is an economically important species for fish production because of its high nutritional value (e.g., high polyunsaturated fatty acid omega-6 levels) and high medical value. The most influential Chinese pharmacy monograph, the Bencao Gangmu, a compendium of materia medica written by the pharmacist Shi-Zhen Li during the Ming Dynasty (AD 1368~AD 1644), recommended *Monopterus* as a natural drug with medicinal virtues to cure several types of diseases, such as facial paralysis, internal haemorrhoid haemorrhage, and other pathogenic conditions described as being influenced by wind and dampness in Traditional Chinese Medicine.

As an emerging model species in development, genetics and evolution [2], *Monopterus* has the attractive feature of undergoing a sex transition from female to intersex to male during its life [3]. This discovery may have considerable theoretical significance in sex determination [4]. *Monopterus* has a small genome size (~800 Mb) and a minimum chromosome number (n = 12) among teleosts, whose chromosome numbers range from 12 to 223 [5]. In addition, all chromosomes of *Monopterus* are telocentric. Given that a third whole-genome duplication occurred in the whole teleost lineage compared to the two genome duplications that occurred in other land vertebrates [6-8], the speciation and sexual differentiation of *Monopterus* may provide new insights into vertebrate evolution. However, the mechanisms of sex determination in the species remain unknown.

Whole-genome sequencing will provide detailed genetic data for studies of genetics, development and evolution and for the genetic manipulation of *Monopterus*. However, no genetic map is currently available for this species. The whole-genome shotgun approach, with high throughput and low cost, is based on a next-generation sequencing (NGS) platform that makes the whole-genome *de novo* assembly of a species possible without the need for a physical map. However, the sequence data produced by NGS are highly fragmented due to the

short lengths of the reads. A number of methods for increasing the contiguity and accuracy of *de novo* assemblies have recently been developed. The read length generated from sequencing can be improved by a third-generation sequencing platform, such as single-molecule real-time (SMRT) sequencing, with raw reads of a mean length of 15 kb [9], and nanopore single-molecular sequencing, with raw reads of approximately 5-50 kb [10, 11]. However, major drawbacks of these techniques include relatively high error rates and high costs of sequencing. Some strategies for the assembly of a long scaffold have also been developed, for example, BAC/fosmid paired end sequencing from large-insert libraries, the long-read sequencing (LRseq) [12] approach, contiguity-preserving transposase sequencing (*fragScaff*)[13], and various assembly algorithms [14, 15]. Recently, chromatin interactions, such as Hi-C, have been used to assemble chromosomes, which can produce ultra-long scaffolds; however, a certain amount of error occurs when used for *de novo* assembly[15, 16]. Thus, accurate chromosome-level assembly remains a major challenge.

The most widely used strategy for chromosome-level assembly of the scaffolds generated by NGS is based on a high-density genetic map on chromosomes. Nevertheless, this strategy is feasible only when complete genetic maps of a species are available. Because there is no genetic map available for *Monopterus*, we have developed an efficient assembly strategy: *de novo* chromosome assembly by FISH walking assisted by conserved synteny (Cafs). Using Cafs technology, which is efficient and cost effective, a precise chromosome-level assembly with 81.3% coverage of the sequenced genome was produced. Using this assembly, we describe a new form of combinatorial regulation of gene expression through interconvertible domains of sex-associated expression (IDSEs) that reasonably explains the sex transition mechanism.

Analysis

Genome sequencing and assembly

The *Monopterus* genome was sequenced with the whole-genome shotgun strategy and the Illumina HiSeq 2000 platform. Eight paired-end libraries with insert sizes up to 40 kb generated a 78.6 GB sequence dataset with a sequencing depth of 97-fold, which provided high single-base accuracy (Additional file: Figure S1 and Table S1). The genome size was estimated from k-mer analysis to be 806 Mb, with a GC content of 40.8% (Additional file: Figures S2-S3 and Table S2). The final assembly comprised 689.5 Mb with contig and scaffold N50 sizes of 22.2 kb and 2.1 Mb, respectively (Table 1). Over 90% of the total sequence was covered by 379 scaffolds; the longest scaffold spanned 11.7 Mb (Table 1). Assembly accuracy was further demonstrated by 99.7% reads mapping to the genome and the successful mapping of 321 bacterial artificial chromosomes (BACs), sequenced with Sanger sequence technology (Additional file: Tables S3).

The repetitive element content (Additional file: Figure S4) of the *Monopterus* genome (28%) was much lower than that of the zebrafish (61%) and about the same as medaka (29%) genomes, but higher than that of the threespine stickleback (16%) and pufferfish (8-10%) genomes. In the *Monopterus* genome, the main repetitive transposable elements were the DNAs and LINEs. At 8%, the LINEs were the largest category of transposable elements. The percent of LINEs was greater than that found in other teleost fish (2-5%), and they acted in concert with Piwi-interacting small RNAs (piRNAs), as guardians for genome defense and germline stability [17].

We used both homology-based and de novo methods to predict genes in the *Monopterus* genome by scanning the database, which also included RNA-seq data. In the *Monopterus*, a total of 24,056 protein-coding genes were predicted (Additional file: Table S4-S5). The gene sizes were similar to those of other teleost fish (Additional file: Figure S5 and Table S6). Approximately 80% of the genes could be functionally annotated with homology analysis (Additional file: Figure S6).

Chromosome-level assembly strategy without a genetic map

To assemble chromosomes with accurate sequences from the scaffolds, we developed an

efficient assembly strategy without using any genetic map information, Cafs (Figure 1), which is based on chromosome fluorescent *in situ* hybridization (FISH) and the shared synteny between closely related fish species.

We first prepared probes of 148 BACs from ~747 sequenced clones (Table S7) and 38 PCR fragment pools, each of which contains 8-15 sequences and covers a total length of 20-30 kb on a scaffold (Table S8) for chromosome FISH. Second, probe combination mapping was used to determine 12 linkage groups, corresponding to 12 chromosomes. Briefly, group A and group B were first discriminated by two unlinked scaffolds labelled with two different colours. If another scaffold was unlinked to the previous two scaffolds, the third scaffold was identified as a marker of group C. Accordingly, 78 efficient combinations of co-hybridization were performed to identify 12 linkage groups, each with a molecular landmark (Figure 1A). Third, based on the predicted syntenic relationship between closely related fish species, probes for the candidate scaffolds were co-hybridized with the landmarks or the sequences, which have been identified. Scaffolds with no predicted location and that were inconsistent with the predicted location were further determined by co-hybridization with 12 landmarks using dual-colour FISH respectively (Figure 1B). For example, scaffold 58 would be grouped into the F group, as it is linked with scaffold 129, which was the landmark of the F group. Fourth, the loose and long pachytene chromosomes were adopted to determine the location and order relationship of the scaffolds through dual- and three-colour FISH. An original marker was used as a walking start (e.g., scaffold 73), and the location of the second scaffold (e.g., scaffold 186) relative to the original marker was identified by dual-colour FISH. The order of another scaffold was determined by the known scaffold locations using dual- or three-colour FISH (e.g., scaffolds 72, 123, and 4) (Figure 1C). Finally, because all 12 chromosomes are telocentric, the telomeres of the metaphase chromosome were used as landmarks to determine the directions of the mapped scaffolds on the chromosomes (Figure 1D). The relative position of each scaffold was determined by the measurement of the signals to the centromere (Figure 1E).

de novo chromosome-level assembly

Using the Cafs assembly strategy, we conducted large-scale mapping of the scaffolds on each chromosome. A scaffold was mapped on each chromosome by FISH, which was used as a landmark (Figure 2A). Using FISH-walking technology, we then conducted the walking in a range of 11-22 steps per chromosome (Figure 2B; Additional file: Figure S7). A total of 186 scaffolds were assembled into 12 pachytene chromosomes through step-by-step combination hybridization of the probes using the above-mentioned 148 BACs and 38 PCR fragment pools (Figure 3A). We then determined the orientation of each chromosome by dual-colour FISH on metaphase chromosomes using the telomere as a morphological landmark (Additional file: Figure S8). Of these mapped scaffolds, 92% (99/108) were consistent with the shared synteny between closely related fish species (medaka, sticklebacks and *Tetraodon*). Therefore, we performed a genome-wide synteny analysis to compare these fish species. From the analysis, 142 of the scaffolds were further assembled into 12 chromosomes respectively. However, 8% (~11) of the scaffolds could not be resolved by the conserved synteny prediction due to possible rearrangements in the *Monopterus* lineage. This issue should be further corrected in future experiments.

We finally integrated 328 scaffolds into 12 chromosomes. These mapped scaffolds consisted of 455 Mb determined by FISH and 100 Mb determined by syntenic analysis, which covers genomic sequences of a total length of 555 Mb, accounting for 81.3% of the sequenced genome of 683 Mb with 97.6X. Based on the assembly, a total of 18,660 protein-coding genes were annotated with location information on the chromosomes (Table 2). For example, there are 87 protein-coding genes on scaffold 72, which was located on chromosome 5 (Figure 3B). These data indicate that a *de novo* chromosome-level assembly of the *Monopterus* genome was produced using an efficient Cafs strategy.

Chromosome-wide association of gene clustering with gene expression

The most attractive feature of *Monopterus* is its natural transition of the gonads from ovary to ovotestis to testis during its life. Haematoxylin and eosin staining was used to identify the

gonads. In a female animal, follicles in different periods of development exist in the ovary. As sex transition occurred, the follicles degenerated, and sperm appeared in seminiferous tubules. Ultimately, the gonad completed its transition from female to male (Figure 4A). To depict the expression profiles of the *Monopterus* genome during gonad transition and to establish a transcriptome atlas, deep transcriptome sequencing was conducted using gonad samples of the three developmental stages (ovary, ovotestis and testis). We analysed the distribution patterns of genes on chromosomes in accordance with their expression profiles. Genome-wide analysis showed a significant positive correlation between the gene density and expression level in the three types of gonads (p-value<2.2e-16) (Figure 4B; Additional file: Figure S9A).

To further investigate gene clustering along the chromosomes, we calculated the gene density per chromosome. The average gene density in the genome was 33.6 genes per Mb, with the maximum gene density on chromosome 12, which is the shortest chromosome, and the minimum gene density on chromosome 9 (Table 2). Further sliding window analysis showed that there was also biased distribution of the gene density within the chromosome (Figure 4B). Using a 1-Mb window size and 100-kb step size, the maximum gene density in the genome was detected from nt 22,200,001 to nt 23,200,000 on chromosome 10, which contains 71 genes, in comparison with an average of 33.6 genes per Mb in the genome (Figure 4C). The distribution pattern of the gene density was consistent with the corresponding GC content along the chromosomes (Figure 4B; Additional file: Figure S9B).

To investigate whether the distribution of the genes along the chromosomes is non-random, we analysed the gene density with a series of window sizes (0.2, 0.3, 0.5, 1, 2, 3 Mb) and a step of 100 kb. We employed ridges to describe the chromosome regions with high gene density. Gene density ridges are characterized by two parameters: gene number per window (cutoff 1) and consecutive window numbers (cutoff 2). Using a combination of the two cutoffs, the distribution pattern of the ridges of each chromosome can be identified. For example, using cutoffs of 40 genes per Mb and 5 consecutive windows, 7 ridges on chromosome 10 were identified (Figure 4C), and 90 ridges were identified in the genome (Figure 4D). The probability of the observed ridges occurring in random permutations of gene

positions was significantly low (p-value < 10^{-4}) (Figure 4D), confirming non-random and clustering distribution of genes along the chromosomes. Probabilities (ridge numbers in random ≥ observations) for a series of cutoff sets and different window sizes were also calculated and showed a highly significant difference in ridges with high gene density along chromosomes in comparison with random permutations of gene positions (Figure 4D; Additional file: Figure S10). The ridge numbers of high gene density directly reflect the clustering of genes along the chromosomes. These analyses suggest that the ridge pattern on the chromosomes probably represents a higher-order structure in the genome.

Identification of types of gene expression during gonad transition

To identify the key gene types during gonad transition, all genes were classified according to their expression trends during gonad transition. Because of the three stages of gonad differentiation, all genes were classified into 9 types, 8 of which were differentially expressed (Figure 5A). While the non-differentially expressed genes (type IX) accounted for the highest proportion (5354/18660 = 28.7%), a majority of the differentially expressed genes were clearly type IV (up-regulated from ovotestis to testis) and type V (down-regulated from ovotestis to testis), accounting for 23.8% and 20.2%, respectively. The gene numbers of each type had no distribution bias among the chromosomes (Additional file: Figure S11). Gene Ontology (GO) analysis showed that the GO terms in the biological process category were over-represented in all types, except for types VII and VIII due to their low gene numbers. Type IV genes were mainly enriched in "translation", "cell projection organization", "determination of bilateral symmetry", and "urogenital system development" (for example, the *cyp51* gene), whereas type V genes were mainly enriched in "protein localization", "RNA processing", and "apoptosis" (for example, the *casp3* gene) (Additional file: Figure S12). Interestingly, the type IV genes were enriched in the "steroid biosynthesis" pathway. For example, the key genes *cyp51* and *dhcr24*, which were clearly up-regulated during gonad transition, were identified in this pathway (Figure 5B). Moreover, the type V genes were enriched in the "progesterone-mediated oocyte maturation" pathway. For example, *mad2l2* and *Rps6ka* were down-regulated in this pathway (Figure 5C).

Clustering patterns of different types of differentially expressed genes in the genome

To evaluate the distribution of gene types along the chromosomes, we further analysed the gene percentage per window (GPW) of each type of genes in the genome. The GPW was calculated as the proportion of a type of genes in the total genes per window and was used to evaluate the distribution of clustering, in which a group of genes was observed as a ridge. Here, the GPW ridges were also defined by two parameters, the GPW (cutoff 1) and consecutive window number (cutoff 2). Using both cutoffs in combination, a series of ridges for each type of genes along the chromosomes could be identified (Figure 6A, 6C; Additional file: Figure S13-S14). When defining a ridge with a GPW cutoff higher than 23% and at least 4 consecutive windows and using a window size of 1 Mb (step = 100 kb), the type IV genes showed the highest degree of clustering, and the type V genes also showed a high degree of clustering in comparison with the other types of genes (Figure 6C). This analysis showed that co-regulated genes of different types tend to be clustered on chromosomes in a large-scale, especially type IV and V genes.

To further characterize the clustering patterns of each type of gene, we analysed the continuous degree of gene clustering. Within a cluster, maximum gene numbers of 8 were detected in types IV and V (Figure 7A). We further tested the significance of the gene clustering when the number of clusters observed in the data was significantly greater than that expected under a null model. We therefore estimated the numbers of clusters corresponding to background noise (Figure 7B). The observed numbers of singleton genes of all co-regulated gene types were significantly lower (p-value = 10^{-4}, p-value < $2=10^{-5}$, p-value < 10^{-5}, p-value = 10^{-4}, p-value < 10^{-5}, p-value < 2.1×10^{-3}, p-value = 4×10^{-3}, p-value = 1.65×10^{-2}, and p-value < 1×10^{-5} for each type of genes) in the real genome than in the permutated genomes. For types IV and V, the numbers of clusters with 8 genes in the real genome were significantly higher than expected under a null model (p-value = 9.5×10^{-3} and 4.8×10^{-2} for IV and V, respectively). These analyses further confirmed the high degree of clustering of type IV and V genes on the chromosomes.

Co-regulation of sex-associated expression interconvertible domains during sex transition.

Because type IV and V genes are the two main groups of differentially expressed genes in the genome, we investigated their distribution patterns on all chromosomes using sliding window analysis. Notably, types IV and V revealed an alternating distribution pattern in the genome (Figure 8A). The GPWs of these two types of genes showed a significant negative correlation (Spearman rank correlation test, p-value<2.2e-16) (Figure 8B). In comparison with random distribution, the absolute values of the correlation coefficient of the GPW between the type IV and V genes were significantly high, in the range from 0.3 Mb to 2 Mb, especially for the window size of 0.5 Mb (p-value=8×10^{-3}) (Figure 8C; Additional file: Figure S15). This data showed that the types IV and V genes occupy different chromosome regions in a mutual exclusive distribution manner.

The distribution pattern can be described in interconvertible regions of sex-associated expression (IRSE). IRSEs explain that one gene type has a higher GPW in a certain region compared to its neighbouring regions on the chromosome, while the other type of genes has a lower GPW in this region compared to its neighbouring regions on the chromosome, which emerge in an alternating, mutually interconvertible manner along the chromosomes (Figure 8D).

To further characterize the co-regulation features of the type IV and V genes in the genome, we analysed the frequency distribution of the IRSEs and the intervals between IRSEs along chromosomes. The distribution follows an exponential function with R^2=0.8284. The interval length ranged from 0.15 to 3.4 Mb with an enrichment of 0.55 Mb in the genome, 90% of which were 0.25-1.5 Mb (Figure 8E). This distribution of interval lengths matched the sliding window analysis of the correlation between the GPW of the type IV and V genes. These two types of genes showed a distinct positioning pattern along the chromosome, suggesting an obvious association between coordinated expression and their loci on chromosome (Figure 9).

Altogether, the clustering and positioning features of genes with coordinated expression suggested a co-regulation mechanism of interconvertible domains of sex-associated expression (IDSE) during sex transition.

Discussion

Chromosome-level assembly after genome sequencing is a massive challenge, and there is an inevitable need for obtaining complete genome information to understand the genome structure, variation and functions. Teleost chromosome assembly is more difficult if no genetic map is available because of the complex genome constitutions caused by the additional whole-genome duplication that occurred in the fish lineage during evolution in comparison with land vertebrate genomes. Chromosome-level assembly based on a genetic map provides a reasonable solution for fish genomes. Of the 38 fish genomes sequenced thus far (Table S9), 15 have been assembled based on genetic maps, with only the *Tetraodon* genome assembled without genetic maps [8], while the other 22 species do not have chromosome assemblies. In the absence of a genetic map, we describe a strategy for *de novo* chromosome assembly by FISH walking assisted by conserved synteny, which is efficient and cost effective. In the chromosome assembly of *Monopterus*, we combined Illumina sequencing, BAC-end sequencing and FISH walking on loose pachytene chromosomes, assisted by conserved synteny, to produce a precise assembly at the chromosome level, with 81.3% coverage of the sequenced genome. Compared to 64.6% coverage in the *Tetraodon* genome, the *Monopterus* genome assembly is of much higher quality at the chromosome level in the absence of genetic map information. In addition, the *Monopterus* genome assembly facilitates the comparison of genomes at different phylogenetic distances to help infer ancient and recent rearrangement events in genome evolution and to improve the understanding of the mechanisms of genome evolution. This assembly will also play an important role in molecular breeding because the organized genome data of *Monopterus* can serve as a reference to select favoured genes and linked molecular markers near the QTLs of economic importance.

Gene positions on chromosomes are related to their functions in some cases. A typical

example of the position effect is the temporal-spatial expression of homeotic genes on the anterior-posterior axis, which is consistent with their position in the clusters on chromosomes [18]. Genome-wide studies showed that eukaryotic genes with coordinated expression are often clustered on chromosomes [19]. However, most gene functions in vertebrates are not dependent on their position in the genome, and many genes have only a minor quantitative position effect on their coordinated expression [20]. Various developmental processes probably have different types of position effects and clustering. The types and underlying mechanisms of the position effects remain largely unknown. In a large-scale gene ridge analysis of the *Monopterus* genome, gene clustering was evident. In particular, the type IV and V genes showed a high clustering pattern, which was related to gonad transition. Thus, gene position effects and clustering probably play a certain role in some developmental processes, such as in gonad differentiation.

In the present study, we found a co-regulation mechanism of IRSEs during sex transition (Figure 9). Two types of genes with opposite expression modes tend to be chromosomally clustered separately and are collaboratively expressed in an alternating, interconvertible manner during sex transition. Co-regulated genes within a cluster potentially contact each other to form an interconvertible domain of sex-associated expression (IDSE) through chromatin folding. In detail, IRSE-o (high expression in the ovary) in a chromosomal segment containing many down-regulated genes (type V genes) showed a relatively high level of expression in the ovary and low expression in the testis during gonad transition. Similarly, IRSE-t (high expression in the testis) in a chromosomal segment containing many up-regulated genes (type IV genes) showed a relatively high level of expression in the testis and low expression in the ovary during gonad transition. According to the mutually exclusive distribution of the type IV and V genes detected by our data, the IRSE-o should occupy a different topological domain from the IRSE-t. Accordingly, the gene expression changes during gonad transition can be described as high-expressed IRSEs in the ovary transitioning to low-expressed IRSEs in the testis, while low-expressed IRSEs in the ovary transitioning to high-expressed IRSEs in the testis. A hypothetical model is proposed in which the IRSE may potentially fold into the IDSE by chromatin folding. Thus, the low-expressed IDSE-t in the ovary will transitioning to

high-expressed IDSE-t in the testis, while the high-expressed IDSE-o in the ovary will become low-expressed IDSE-o in the testis (Figure 9). The IDSEs share two similar features with topologically associating domains (TADs): the “co-regulation” of genes within the IDSEs and the blocking of the “spread” of activity between neighbouring IDSEs [21]. A distinct feature of the IRSEs is the exponential size distribution of the IRSEs, with an enrichment of ~0.5 Mb. In addition, the IDSEs are closely associated with sex determination. Thus, the IDSEs represent a new type of combinatorial regulation of gene expression.

The underlying molecular mechanisms of sex transition are largely unknown thus far, although several genes involved in sexual development, such as *Sox9* and *Dmrt1*, have been identified in *Monopterus* [22, 23]. The IDSEs and chromosomal clustering of reproductive genes probably result from selection in favour of sex transition. Position effects have shaped the evolution of the chromosome organization [24], which could facilitate the formation of the IDSEs during the speciation of *Monopterus*. According to the mutually exclusive distribution and regulation of the type IV and V genes, we further determined two regulatory pathways during gonad transition. The regulatory factors, as key members of the IRSEs, potentially participate in gonad transition. As the transition from ovary to testis must occur during the lifetime of *Monopterus*, the same set of the genome should convert its expression globally to ensure programmed ovary degradation followed by testis reconstruction. Thus, the interconvertible regulation model, IDSEs, could explain the sex transition process to that point, thus suggesting a new mechanism of sex determination at both the transcriptional and higher chromatin organization levels.

Conclusions

Monopterus has the attractive feature of undergoing a sex transition from female to intersex to male during its life. However, the mechanisms of sex determination in the *Monopterus* are poorly understood. We provided an efficient Cafs strategy for a *de novo* chromosome-level assembly of the *Monopterus* genome. Genome-wide analysis together with transcriptomes of the three stages of gonadal development showed a co-regulation mechanism of the

interconvertible regions of sex-associated expression (IRSEs) during sex transition. The IRSEs probably represent a new type of combinatorial regulation of gene expression in a higher chromatin structure. This represents a remarkable mechanism for sex determination at both the transcriptional and chromatin organization levels.

Materials and Methods

Ethics statement

Monopterus were obtained from Hubei, China. All animal experiments and methods were performed in accordance with the relevant approved guidelines and regulations, as well as under the approval of the Ethics Committee of Wuhan University.

DNA library construction and sequencing

A whole-genome shotgun strategy and next-generation sequencing technology (Illumina HiSeq 2000 platform) were used to sequence two male *Monopterus*. Genomic DNA was extracted from eels from the Wuhan area in the Yangtze River basin. To reduce the risk of non-random sequencing, 8 paired-end sequencing libraries with insert sizes of 170 bp, 500 bp, 800 bp, 2 kb, 5 kb, 10 kb, 20 kb, and 40 kb were constructed for the genome. The libraries generated 101.62 GB of sequence data. To reduce sequencing errors in the assembly, sequence reads were filtered to remove low quality reads. After filtering, 78.64 GB (97.6X) of sequence data were retained for the assembly.

Estimation of genome size

A k-mer was defined as a sequence of k bases in length. The frequency of k-mers in a collection of short, insert-sized reads could be calculated with a 1 bp sliding window. When an optimal amount of data was present, the k-mer frequency followed a Poisson distribution. The k-mer value was used to estimate the genome size, as follows: Genome Size = K_num/Peak_depth, where K_num is the total number of k-mers, and Peak_depth is the expected value of the k-mer depth [25]. The 17-mer distribution obeyed the theoretical Poisson distribution. Finally, we found that the proportion of heterozygosity in the *Monopterus* genome was small, and estimated that the entire

genome comprised 806 Mb.

Genome assembly

The *Monopterus* genome was de novo assembled with the SOAPdenovo software [25] (http://soap.genomics.org.cn). SOAPdenovo employs the de Bruijn graph algorithm to simplify assembly and reduce the computational complexity. Low quality reads were filtered out and potential sequencing errors were removed or corrected with the k-mer frequency methodology. The SOAPdenovo assembly process consisted of three main steps: contig construction, scaffold construction, and gap filling.

About 39 GB (48X) of the short-insert-size, clean data was used to build contigs, and all clean sequence reads were used to build scaffolds with SSPACE (version 1.1) software. To assess assembly quality and completeness, high quality reads from short-insert-size libraries (75 bp read lengths) were aligned to the assembly with the BWA program [26] (version 0.5.9-r16), with default parameters. Next, SOAPcoverage (version 2.27) was used to calculate sequencing depth. A total of 91.06 % reads could be mapped, and they covered 99.69 % of the assembly, excluding gaps. To further test for possible contigs that might be mis-joined in scaffolds, we analyzed paired-end information. We found that, if contigs were included only when both ends could be uniquely mapped onto the assembly, more than 90.65 % of paired-ends were in the correct orientation and at the expected distance, according to the utilized short-insert-size libraries.

Repeat annotation

Transposable elements (TEs) were identified in the genome with combination of homology-based and de novo approaches. The homology-based approach utilized database Repbase [27] (release 19.06), with RepeatMasker (version 4.0.3) and RepeatProteinMask (from the RepeatMasker package) programs with the default parameters [27]. The de novo approach used two prediction programs, RepeatModeler [28] (version 1.0.7) and LTR-FINDER [29] (version 1.0.5), to build the de novo repeat libraries based on the genome sequences. Next, contaminations and multi-copy genes were removed from the libraries. Then, the RepeatMasker was used a second time to find repeats in these repetitive sequence libraries. Finally, we combined all the results generated by these methods. To improve our comparisons to other teleost fishes, we employed the same

procedure and parameters to analyze the *Danio rerio*, *Oryzias latipes*, *Gasterosteus aculeatus*, *Tetraodon nigroviridis*, and *Takifugu rubripes*.

Gene annotation

We used both homology-based and de novo methods to predict genes in the *Monopterus* genome by scanning the database, which also included RNA-seq data. For the homology-based prediction, protein sequences from D. rerio, O. latipes, G. aculeatus, T. nigroviridis, and T. rubripes were downloaded from the Ensembl platform[30] (release 75) and aligned with the *Monopterus* genome with the Tblastn program[31]. Accordingly, homologous genomic sequences were input into the Genewise program[32] to align matching proteins. This procedure allowed us to define gene structures. For de novo prediction, both the Fgenesh[33] and Genscan[34] programs were employed to predict coding genes, with the appropriate parameters. Homology-based and de novo derived gene sets were combined with comprehensive, non-redundant reference gene sets, obtained with the GLEAN platform (http://sourceforge.net/projects/glean-gene/). Genes were corrected by comparisons with the RNA-seq data; these RNA-seqs were mapped to the *Monopterus* genome with the Tophat program, and the Cufflinks program (http://cufflinks.cbcb.umd.edu/) was used to assemble transcripts. After that, we selected 1000 intact genes, defined as gene set "A", which were supported by the homology-based prediction, and passing a fifth-order Markov model, to verify the ORFs of RNA transcripts based on the Hidden Markov Model (HMM).

Gene function annotations

Blastp was used to search for proteins encoded in the M. albus genome by comparing candidate sequences against the SwissProt and TrEMBL databases from UniProt Knowledgebase (UniProtKB) [35]. The annotated motifs and domains in the available databases (ProDom, PRINTS, Pfam, SMART, PANTHER, and PROSITE) were obtained with the InterProScan program [36] (version 4.7). In gene ontology (GO) [37] analyses, gene functions were obtained from the corresponding InterPro entries. Subsets of the GO terms were obtained according to the program of DAVID program (version 6.7) [38] X-associated genes were annotated based on human GO term list and Z-associated genes were annotated based on chicken GO term list. All

genes were also aligned against the KEGG [39] (release 68) protein database. The genes that matched genes in the KEGG database were assumed to be involved in the corresponding signaling pathways.

Linkage prediction and synteny analysis

We constructed a reference map using the syntenic relationship among the genomes of medaka, stickleback and *Tetraodon* to help map the scaffolds on the *Monopterus* chromosomes. The syntenic blocks between *Monopterus* and other fishes were aligned by Lastz (Blastz) [40] with parameters of T=2 and Y=3400. Furthermore, we used Blat to search for homologous sequences among medaka, sticklebacks, *Tetraodon* and *Monopterus* in order to fill the gap sequences of blocks in the reference map. If two homologous sequences were linked in all three close species, we defined the corresponding scaffolds in *Monopterus* as predicted linked scaffolds.

BAC end sequencing and aligning to scaffolds

The whole genome BAC library of the *Monopterus* was constructed in our laboratory [41]. BAC DNAs were extracted by a routine protocol and purified using an EZNATMBAC/PAC DNA kit (Omega, USA) according to the manufacturer's methods. BAC end sequencing was performed on an ABI 3730xl DNA Analyzer at Sangon Biotech (Shanghai, China). The primers for the BAC end sequencing were designed from the flanking region of the *Bam*HI cloning site of the pIndigoBAC-5 vector (Epicentre, Madison, USA). The sequences of the primers are

pIB FP 50-GGATGTGCTGCAAGGCGATTAAGTTGG-30,

pIB RP 50-CTCGTATGTTGTGTGGAATTGTGAGC-30.

The BAC end sequences were aligned to the genome database by Blat. BACs with two ends aligned to one scaffold, and those ends with sequences with homology to scaffolds greater than 90% were used as probes for FISH. The BACs were confirmed by PCR sequencing from the internal regions of the BACs.

Chromosome preparation

Metaphase chromosomes were prepared according to routine protocols from the *Monopterus* kidney tissue [5]. Meiotic pachytene bivalents were prepared from *Monopterus* testis using a previously described method [42]. In brief, the testis samples were separated and directly treated with hypotonic solution (0.75 M KCl: 0.45% sodium citrate (1:1, v/v)) for 6-8 h at 26 °C, then being pre-fixed with fixative I (chloroform: absolute ethanol: acetic acid (6:3:1, v/v/v, 37 °C) for 15 min. They were then transferred into ice cold fixative II (absolute ethanol: acetic acid (3:1, v/v) and treated for 15 min at 37 °C. After the germ cells were released from the fixative by tearing, they were fixed three times in fixative II for 15 min at 37 °C. Finally, the cell suspension was dropped onto a cold glass slide and allowed to air-dry.

Fluorescent *in situ* hybridization

For FISH to chromosomes, we used BACs and PCR fragments as probes. BAC DNAs were extracted by routine protocol and purified using an EZNATMBAC/PAC DNA kit (Omega Bio-Tek, Doraville, USA) according to the manufacturer’s methods. To synthesize a probe long enough to obtain a stronger signal; 8-15 sequences with a length of 1.5-2.5 kb each on a scaffold were taken as the template for PCR. The series of PCR fragments were mixed into a PCR pool that covers a total length of 20-30 kb on a scaffold.

The FISH was conducted as previously described [43], with modifications. These BACs and mixed PCR segments were labelled with digoxigenin-11-dUTP (Roche, Lewes, UK) or biotin-16-dUTP (Roche) or both by standard nick translation. A hybridization mixture of 20 µl [100 ng of total labelled probe, 20 µg of salmon sperm DNA (Life Technologies, Carlsbad, USA), 10 µl deionized formamide (Amresco, Solon, USA), and 10% dextran sulfate (Dingguo Changsheng Biotech, Beijing, China), 2 x SSC] was denatured at 75 °C for 5 min and allowed to hybridize overnight to the denatured chromosomes at 37 °C. The digoxigenin-labelled probes were detected and amplified with anti-digoxigenin-fluorescein, Fab fragments from sheep (Roche) and FITC-AffiniPure rabbit anti-sheep IgG (H+L) (Sigma, St. Louis, USA). Biotin-labelled probes were detected and amplified with Cy3 streptavidin (Jackson ImmunoResearch, West Grove, USA) and biotinylated anti-streptavidin (Vector Laboratories, Burlingame, USA) respectively. After the chromosomes were washed and counterstained with DAPI (49-6-diamidino-2-phenylindole)

(Sigma, St. Louis, USA), chromosome images were obtained using a Leica DMLA fluorescence microscope (Leica, Wetzlar, Germany).

FISH walking

The first 12 scaffolds non-linked with each other were used as landmarks corresponding to 12 chromosomes, and the other scaffold locations were then determined by hybridization with the landmark probes. The identified scaffolds could also be used as landmarks. The scaffold located at one end of a chromosome was used as a walking start, and the scaffold order was determined in a stepwise manner by dual- or three-colour FISH. The orientation of a linkage group on a chromosome was determined by two dual-colour FISH labelled probes at either end of the metaphase chromosome. The relative localization of the scaffolds was calculated by the ratio of the distance from the signal to the centromere to the length of the chromosome. The distance values were measured by Image-Pro Plus 6.0, and each value was obtained from an average of more than five cells.

Transcriptome sequencing and expression analysis

The total RNAs were extracted from three tissues (ovary, ovotestis, and testis) of three developmental stages of *Monopterus*. mRNAs were enriched using Oligo(dT) and then broken into 200-500 bp fragments to facilitate cDNA library construction. The short cDNA fragments were purified using a QiaQuick PCR extraction kit (QIAGEN, Hilden, Germany), and the library was sequenced on an Illumina HiSeq2000. To determine the gene expression levels, the RNA-Seq reads from three tissues were mapped to the reference genome by TOPHAT [44] (version 1.3.3), and the reads per kilobase per million reads (RPKM) [45] values were calculated for each gene.

Analysis of differentially expressed genes

We compared the gene expression levels (RPKM) between two stages after removing genes if their RPKM<0.1. We defined genes as differentially expressed if they showed at least a 2-fold change in expression and an FDR (false discovery rate) < 0.05 (*p*-value<0.01) (Mann-Whitney U test) (based on the Poisson model) [46]. Genes were classified as

expression types according to their expression patterns through three developmental stages. Sliding window analysis with a window size of 1 Mb and step of 100 kb was performed to characterize the gene distribution on the chromosomes as a percentage of each type of gene in the total genes within one window.

Statistical analysis

To test whether the distribution of genes on the chromosomes was random or non-random, we computed the probability of ≥ actual numbers of ridges under a random permutation of the gene positions following previous method [47]. A ridge was used to describe a chromosome region with high gene density, which is thus defined as at least W consecutive windows, each containing a gene number higher than H. Thus, the ridge is determined by two parameters: cutoff 1 (C_H), gene number per window, and cutoff 2 (C_W), number of consecutive windows. The actual ridge numbers (N) in the genome were calculated under C_H and C_W by sliding window analysis. We used the following calculation parameters to set up a null model: suppose we have a random permutation of X_1, X_2,..., X_i in the range of 1 to S; i, gene number on the chromosome; S, length of the chromosome; and X_1, X_2,..., X_i, gene locations on the chromosome. With the same cutoff values under actual conditions (C_H and C_W), we can obtain a ridge number (n) under the null model. We can compute the frequency (f) when n ≥ N by permutation 10,000 times. If f = 0, the *p*-value < 10^{-4}, or the *p*-value = f/10000. For all of the cutoff C_H and C_W combinations, we calculated the *p*-value under different window sizes of 0.2, 0.3, 0.5, 1, 2, and 3 Mb respectively.

For assessing the distribution of the co-regulated types of genes, we first counted the GPW of each gene type for all windows along chromosomes. The total gene number within a window is N_T and the gene number of certain types of genes is Ni (i = 1, 2,..., 8); thus, GPW-i = N_i/N_T. The ridges were also defined by two parameters: C_H, gene percentage per window (GPW), and C_W, consecutive window numbers. We set up a null model using the following calculation parameters: the actual gene positions: X_1, X_2,..., X_i, i = gene number on chromosome. Suppose we have a random permutation of Y_1, Y_2,..., Y_j in the range of X_1, X_2,..., X_i, j = gene number of the gene type, and Y_1, Y_2,..., Y_j are defined as the gene locations of this type on the chromosome. The calculation of the *p*-values was the same as above.

To calculate and test the correlation between GPW values for all types of genes, using sliding window analysis with a definite window size and step, we used the non-parametric Spearman correlation test to calculate the correlation coefficient R between observed GPW values. Under the same window size and step size, the correlation coefficient r between GPW-i was also calculated in the null model. One thousand correlation coefficients (r1, r2, …, r1000) were obtained by permutation 1,000 times. We count the number f of occasions where $|r_i|>=|R|$; thus, the *p*-value = f/1000 for all window sizes of 0.2, 0.3, 0.5, 1, 2, and 3 Mb respectively.

Correlation analyses were performed with R software (www.r-project.org). The R package ggplot2 was used to draw scatterplots and boxplots. We tested the statistical significance of pairing correlations among the gene density, GC content, expression levels, per window in the genome and the GPWs of any gene types. As these parameters are not normally distributed, we used the non-parametric Spearman correlation test on the ranks of the paired quantities. Function fitting was performed by the curve fitting tool of Matlab (Vision 7.0).

Circos program

The Circos program (http://circos.ca) was applied to draw the circus maps. Data to generate the chromosome mapping include the parameters of the start and end locations of the scaffolds and the lengths of the chromosomes, which were read in a text. Data to generate the GC content, gene density and expression levels were read in five texts respectively. The GC content was described by the percentages of G and C in a sliding window of 3 Mb (step = 100 kb). The gene densities were described by the gene number in a sliding window of 3 Mb (step = 100 kb). The expression levels were described by the values of $\log_2$ (RPKM+2). The Circos.conf files were called to generate svg files.

Data availability statement

The genome data from this study have been deposited at DDBJ/EMBL/GenBank under accession number AONE00000000, and the raw transcriptome data have been submitted to NCBI Gene Expression Omnibus (GEO; http://www.ncbi.nlm.nih.gov/geo/) under accession

number GSE43649.

Supplemental data

Supplemental data includes fifteen figures and nine tables.

Acknowledgements

This work was supported by the National Natural Science Foundation of China, National Key Technologies R&D Program and Hubei Province Science and Technology Project.

Author contributions

Conceptualization: Rongjia Zhou.

Funding acquisition: Rongjia Zhou, Hanhua Cheng.

Investigation: Xueya Zhao, Majing Luo, Zhigang Li, Yibin Cheng, Jiumeng Min, Mingzhou Bai, Yulan Yang.

Methodology: Xueya Zhao, Majing Luo, Zhigang Li, Pei Zhong.

Supervision: Rongjia Zhou, Hanhua Cheng.

Validation: Xueya Zhao, Rongjia Zhou.

Writing – original draft: Xueya Zhao, Rongjia Zhou.

Writing – review & editing: Xueya Zhao, Rongjia Zhou.

Figure legends

Figure 1 Overview of *de novo* chromosome assembly by FISH walking assisted by conserved synteny. A: Identification of 12 linkage groups by probe combination mapping. FISH probes are hybridized on pachytene chromosomes. Red and green dots indicate scaffold locations. B: Synteny-assisted scaffold mapping. Each candidate BAC (scaffold) is co-hybridized with 12 landmarks by dual-colour FISH respectively. Synteny-supported/non-supported scaffolds are determined by FISH. C: Determination of scaffold order on chromosome by FISH walking. (I) The order of two scaffolds is identified by dual-colour FISH if both of them are on one side of the chromosome. (II) If the scaffolds are in the centre of the chromosome, three-colour FISH

is applied to determine their order. (III) The order of some scaffolds (labelled with one colour) could be identified by three signals dual-colour FISH, when their two neighbouring scaffolds (labelled with another colour) have been determined. D: Identification of orientation of linkage groups on metaphase chromosomes. Telomeres and centromeres can be observed on the metaphase chromosomes. E: Localization of scaffolds is determined by calculating the corresponding distances to the centromere.

Figure 2 Chromosome assembly by Cafs. A: FISH images show 12 molecular landmarks corresponding to 12 chromosomes. Green signals indicate the landmarks labelled by digoxigenin and detected with FITC. Each chromosome is determined by a landmark. Chromosomes are stained by DAPI (blue). B: Localization of each scaffold on chromosome 5 by FISH walking strategy. FISH images and corresponding scaffold order from (a') to (m') are shown in the left panels. A three-colour FISH image (g') in the upper right indicates the relative order of scaffolds 4 (yellow, FITC+Cy3), 30 (green, FITC) and 99 (red, Cy3) on chromosome 5. Probes (red dots) and their locations on scaffolds are used to assemble chromosome 5.

Figure 3 Chromosome-scale assembly of the *Monopterus* genome. A: Each chromosome is assembled with scaffolds and their order from telomere (down end) to centromere (up end). Chromosomes 1 to 12 are determined by their assembled sizes (Mb). The grey and purple cylinders represent the anchored scaffolds. The segments in light blue between two neighbouring scaffolds indicate gaps. Sticks with a red head anchored on each scaffold indicate the positions of the BACs used as probes. Scale bar, 80 Mb. B: Scaffold 72 with 87 genes (blue bars) on chromosome 5 is highlighted.

Figure 4 Genome and expression during gonad transition. A: H&E staining shows the typical features of gonads during sex transition. O, ova; Ode, ova degenerated; T, testis. B: Circos is used to plot the assembled chromosomes, GC content, gene density, and transcriptomes in the ovary, ovotestis and testis. The inner scale is 2 Mb. The strips in the outer circle indicate the scaffolds anchored by FISH. The outer dark grey ridges show the moving GC percentage, and

the inner grey ridges show the moving number of the genes at a window size of 1 Mb. Expression levels [$\log_2$ (RPKM+2)] of genes in ovary, ovotestis and testis are displayed by blue, green, and red lines, respectively. C: Distribution of gene clusters (ridges) on chromosome 10. Curves indicate the moving numbers of genes at a window size of 1 Mb (step = 100 kb). The windows with a maximum gene density from nt 22200001 to 23200000 on chromosome 10, which contains 71 genes, are shown in the lower panel. Green boxes highlight ridges in which there are at least 5 consecutive moving windows with a lower limit of 40 genes per window. D: Statistical tests of numbers of gene density ridges in the genome corresponding to background noise (null model). The heat map in the lower panel shows *p*-values in the significance test of observed ridge numbers against the null model (10,000 independent permutations of gene positions). The *x*-axis indicates the cutoff values of numbers of consecutive moving windows, which reflects the extent of the clustering. The *y*-axis indicates the cutoff values of gene numbers within a certain window size (step 100 kb), which reflects the degree of intensity of the clusters. Green lines represent the average gene number in a certain window size. The upper panel highlights a significance test at the condition of two cutoff values, gene density (40/Mb) and consecutive window numbers (5). Red dots represent the number of observed ridges in the genome. Boxplots (black) represent distribution of the ridge numbers in 10,000 independent permutations of gene positions in a random fashion.

Figure 5 Identification of gene regulation pathways in gonad transition. A: Differentially expressed genes are classified into 8 types in accordance with their expression differences during gonad transition from ovary to ovotestis to testis. Differential expression is identified by fold change of RPKM (≥2). Genes with no expression difference among gonads are shown in type IX. B: Type IV genes are enriched in the steroid biosynthesis pathway with a *p*-value = 0.041 (DAVID, version 6.7). Fisher's exact test (FDR = 0.05) was used to assess the enrichment. Up-regulated genes in the pathway during gonad transition are highlighted in different shades of red (RPKM fold change). Expression patterns of both *cyp51* and *dhcr24* are shown in the right panel. Different shapes indicate the nature of the molecules. C: Type V genes are enriched in the progesterone-mediated oocyte maturation pathway with a *p*-value =

0.04. Down-regulated genes in the pathway during gonad transition are highlighted in different shades of blue (RPKM fold change).

Figure 6 Clustering patterns of different types of differentially expressed genes in the genome. A: Distribution of different types of differentially expressed genes on chromosome 8. The *y*-axis indicates the percentage of each type of genes in the total genes within a 1-Mb sliding window (step 100 kb) along chromosome 8. B: Expression profiles of typical genes *fgf8b, casp3, cyp51a1 and eomes* on chromosome 8 during gonad transition. O, ovary; OT, ovotestis; T, testis. C: Statistical tests of ridge numbers of the gene percentages per window (GPW) of different types of genes in the genome corresponding to background noise (null model). The heat map shows the *p*-values in the significance test of the observed ridge numbers against the null model (10,000 independent permutations of gene positions). The *x*-axis indicates the cutoff values of the numbers of consecutive moving windows, which reflects the extent of the clustering. The *y*-axis indicates the cutoff values of GPW, which reflects the degree of intensity of the clusters. Green boxes indicate a higher clustering degree with a GPW cutoff of higher than 23% and at least 4 consecutive windows.

Figure 7 Frequency distribution of the size (in number of genes) of consecutive gene clusters of co-regulated genes of different types in the genome. A: Pie diagram represents number distribution of different sizes of consecutive gene clusters of co-regulated genes of different types in the genome. Gene cluster sizes (n=1, 2, ...8) are indicated in different colours. B: Significant clustering of type IV and V genes in the genome. Boxplots represent the distribution of cluster numbers for each size of cluster and their statistical tests against background noise (null model) in 10,000 independent permutations of gene positions. Red dots represent the numbers of observed clusters for each cluster size (in number of genes). Boxplots with a red background indicate that the observed number for the size of the cluster is significantly different from the number obtained by a random distribution. The observed numbers of singleton genes of all co-regulated gene types are significantly lower in the real genome than in the permutated genome, and there is a significant clustering of co-regulated genes in the *Monopterus* genome, especially for type IV and V genes.

Figure 8 Distribution features of type IV and V genes on chromosomes. A: Sliding window analysis reflects the distribution patterns of differentially expressed genes of types IV and V in the *Monopterus* genome. The *y*-axis indicates the percentages of the two type of genes in the total genes within a 1-Mb sliding window (step 100 kb) along each chromosome. The *x*-axis indicates the window locations along the chromosome. Red curves represent the type IV genes up-regulated from intersex to male. Blue curves represent the type V genes down-regulated from intersex to male. The distribution shows a pattern of alternating sine curves along all chromosomes. B: Correlation analysis of gene percentage per window (GPW) for type IV (*y*-axis) and V (*x*-axis) genes in the genome, which shows a significant negative correlation (p-value<2.2e-16). C: Frequency distribution of the correlation coefficient of GPW of type IV and V genes in different window sizes in the genome and their statistical tests. Red dots represent the observed correlation coefficient in the genome. Boxplots represent the distribution of the correlation coefficients of the GPWs of type IV and V genes in 1,000 independent permutations of gene positions (null model). The *p*-value is shown in blue font. The distribution of type IV and V genes showed significant mutual exclusion in a range from 0.3 Mb to 2 Mb, especially at 0.5 Mb. D: Distribution model of type IV and V genes on chromosomes. Red and blue curves correspond to the curves shown in Figure 6. Red and blue blocks represent alternating interconvertible regions of sex-associated expression (IRSE-o, high expression in ovary; IRSE-t, high expression in testis). Intervals indicate the distance (in Mb) between two IRSEs. E: Scatterplot showing a frequency distribution of the interval length in the genome. The *x*-axis indicates the interval length. The *y*-axis indicates the interval numbers. The distribution follows an exponential function with R^2=0.8284.

Figure 9 A co-regulation pattern of interconvertible domain of sex-associated expression (IDSE) during sex transition. Chromosome 10 is used as an example to illustrate a process of the IDSEs transition. Red and blue bars indicate type IV and V genes on chromosome 10 respectively. Co-regulated genes potentially contact each other to form the IDSE through chromatin folding. Red and blue blocks indicate up- and down-regulated IDSEs from ovary to testis respectively. Low-expressed IDSE-t (-) in ovary will transitioning to high-expressed

IDSE-t (+) in testis, while high-expressed IDSE-o (+) in ovary will be low-expressed IDSE-o (-) in testis.

References

1. Collins TM, Trexler JC, Nico LG, Rawlings TA. Genetic Diversity in a Morphologically Conservative Invasive Taxon: Multiple Introductions of Swamp Eels to the Southeastern United States. Conserv Biol. 2002; 16:1024-35.
2. Cheng HH, Guo YQ, Yu QX, Zhou RJ. The rice field eel as a model system for vertebrate sexual development. Cytogenet Genome Res. 2003; 101:274-7.
3. Liu CK. Rudimentary hermaphroditism in the symbranchoid eel, Monopterus javanensis. Sinensia. 1944; 15:1-8.
4. Bullough WS. Hermaphroditism in the lower vertebrates. Nature. 1947; 160:9-11.
5. Yu XJ, Zhou T, Li YC, Li K, Zhou M. Chromosomes of Chinese fresh-water fishes. Beijing Science Press. 1989:1-148.
6. Zhou RJ, Cheng HH, Tiersch TR. Differential genome duplication and fish diversity. Rev Fish Biol Fisher. 2002; 11:331-7.
7. Christoffels A, Koh EG, Chia JM, Brenner S, Aparicio S, Venkatesh B. Fugu genome analysis provides evidence for a whole-genome duplication early during the evolution of ray-finned fishes. Mol Biol Evol. 2004; 21:1146-51.
8. Jaillon O, Aury JM, Brunet F, Petit JL, Stange-Thomann N, Mauceli E, et al. Genome duplication in the teleost fish Tetraodon nigroviridis reveals the early vertebrate proto-karyotype. Nature. 2004; 431:946-57.
9. Huddleston J, Ranade S, Malig M, Antonacci F, Chaisson M, Hon L, et al. Reconstructing complex regions of genomes using long-read sequencing technology. Genome Res. 2014; 24:688-96.
10. Goodwin S, Gurtowski J, Ethe-Sayers S, Deshpande P, Schatz MC, McCombie WR. Oxford Nanopore sequencing, hybrid error correction, and de novo assembly of a eukaryotic genome. Genome Res. 2015; 25:1750-6.
11. Chaisson MJ, Huddleston J, Dennis MY, Sudmant PH, Malig M, Hormozdiari F, et al. Resolving the complexity of the human genome using single-molecule sequencing. Nature. 2015; 517:608-11.
12. Voskoboynik A, Neff NF, Sahoo D, Newman AM, Pushkarev D, Koh W, et al. The genome sequence of the colonial chordate, Botryllus schlosseri. Elife. 2013; 2:e00569.
13. Adey A, Kitzman JO, Burton JN, Daza R, Kumar A, Christiansen L, et al. In vitro, long-range sequence information for de novo genome assembly via transposase contiguity. Genome Res. 2014; 24:2041-9.
14. Kim J, Larkin DM, Cai Q, Asan, Zhang Y, Ge RL, et al. Reference-assisted chromosome assembly. Proc Natl Acad Sci U S A. 2013; 110:1785-90.
15. Putnam NH, O'Connell BL, Stites JC, Rice BJ, Blanchette M, Calef R, et al. Chromosome-scale shotgun assembly using an in vitro method for long-range linkage. Genome Res. 2016; 26:345-50.
16. Burton JN, Adey A, Patwardhan RP, Qiu R, Kitzman JO, Shendure J. Chromosome-scale

scaffolding of de novo genome assemblies based on chromatin interactions. Nat Biotechnol. 2013; 31:1119-25.

17. Reuter M, Berninger P, Chuma S, Shah H, Hosokawa M, Funaya C, et al. Miwi catalysis is required for piRNA amplification-independent LINE1 transposon silencing. Nature. 2011; 480:264-7.

18. Graham A, Papalopulu N, Krumlauf R. The murine and Drosophila homeobox gene complexes have common features of organization and expression. Cell. 1989; 57:367-78.

19. Hurst LD, Pal C, Lercher MJ. The evolutionary dynamics of eukaryotic gene order. Nat Rev Genet. 2004; 5:299-310.

20. Semon M, Duret L. Evolutionary origin and maintenance of coexpressed gene clusters in mammals. Mol Biol Evol. 2006; 23:1715-23.

21. Dixon JR, Gorkin DU, Ren B. Chromatin Domains: The Unit of Chromosome Organization. Mol Cell. 2016; 62:668-80.

22. Zhou RJ, Liu L, Guo YQ, Yu HS, Cheng HH, Huang X, et al. Similar gene structure of two Sox9a genes and their expression patterns during gonadal differentiation in a teleost fish, rice field eel (Monopterus albus). Mol Reprod Dev. 2003; 66:211-7.

23. Huang X, Guo YQ, Shui Y, Gao S, Yu HS, Cheng HH, et al. Multiple alternative splicing and differential expression of dmrt1 during gonad transformation of the rice field eel. Biol Reprod. 2005; 73:1017-24.

24. Chen XS, Zhang JZ. The Genomic Landscape of Position Effects on Protein Expression Level and Noise in Yeast. Cell Syst. 2016; 2:347-54.

25. Li RQ, Fan W, Tian G, Zhu HM, He L, Cai J, et al. The sequence and de novo assembly of the giant panda genome. Nature. 2010; 463:311-7.

26. Li H, Durbin R. Fast and accurate short read alignment with Burrows-Wheeler transform. Bioinformatics. 2009; 25:1754-60.

27. Jurka J, Kapitonov VV, Pavlicek A, Klonowski P, Kohany O, Walichiewicz J. Repbase Update, a database of eukaryotic repetitive elements. Cytogenet Genome Res. 2005; 110:462-7.

28. Price AL, Jones NC, Pevzner PA. De novo identification of repeat families in large genomes. Bioinformatics. 2005; 21:i351-i8.

29. Xu Z, Wang H. LTR_FINDER: an efficient tool for the prediction of full-length LTR retrotransposons. Nucleic Acids Res. 2007; 35:W265-8.

30. Flicek P, Ahmed I, Amode MR, Barrell D, Beal K, Brent S, et al. Ensembl 2013. Nucleic Acids Res. 2013; 41:D48-55.

31. Mount DW. Using the Basic Local Alignment Search Tool (BLAST). CSH Protoc. 2007; 2007:pdb top17.

32. Birney E, Clamp M, Durbin R. GeneWise and genomewise. Genome Res. 2004; 14:988-95.

33. Burge C, Karlin S. Prediction of complete gene structures in human genomic DNA. J Mol Biol. 1997; 268:78-94.

34. Salamov AA, Solovyev VV. Ab initio gene finding in Drosophila genomic DNA. Genome Res. 2000; 10:516-22.

35. Bairoch A, Apweiler R. The SWISS-PROT protein sequence database and its supplement TrEMBL in 2000. Nucleic Acids Res. 2000; 28:45-8.

36. Mulder N, Apweiler R. InterPro and InterProScan: tools for protein sequence classification and comparison. Methods Mol Biol. 2007; 396:59-70.

37. Ashburner M, Ball CA, Blake JA, Botstein D, Butler H, Cherry JM, et al. Gene Ontology: tool for the unification of biology. Nat Genet. 2000; 25:25-9.

38. Huang da W, Sherman BT, Lempicki RA. Systematic and integrative analysis of large gene lists using DAVID bioinformatics resources. Nat Protoc. 2009; 4:44-57.

39. Kanehisa M, Goto S. KEGG: kyoto encyclopedia of genes and genomes. Nucleic Acids Res. 2000; 28:27-30.

40. Schwartz S, Kent WJ, Smit A, Zhang Z, Baertsch R, Hardison RC, et al. Human-mouse alignments with BLASTZ. Genome Res. 2003; 13:103-7.

41. Jang SH, Zhou F, Xia LX, Zhao W, Cheng HH, Zhou RJ. Construction of a BAC library and identification of Dmrt1 gene of the rice field eel, Monopterus albus. Biochem Biophys Res Commun. 2006; 348:775-80.

42. Yu QX, Fan LC, Cui JX, Ren XH, Li K, Yu XJ. High resolution G-binding and idiogram on pachytene bivalents of rice field eels. Sci China (B). 1994:1090.

43. Henegariu O, Dunai J, Chen XN, Korenberg JR, Ward DC, Greally JM. A triple color FISH technique for mouse chromosome identification. Mamm Genome. 2001; 12:462-5.

44. Trapnell C, Pachter L, Salzberg SL. TopHat: discovering splice junctions with RNA-Seq. Bioinformatics. 2009; 25:1105-11.

45. Mortazavi A, Williams BA, McCue K, Schaeffer L, Wold B. Mapping and quantifying mammalian transcriptomes by RNA-Seq. Nat Methods. 2008; 5:621-8.

46. Chen S, Yang PC, Jiang F, Wei YY, Ma ZY, Kang L. De novo analysis of transcriptome dynamics in the migratory locust during the development of phase traits. PLoS One. 2010; 5:e15633.

47. Caron H, van Schaik B, van der Mee M, Baas F, Riggins G, van Sluis P, et al. The human transcriptome map: clustering of highly expressed genes in chromosomal domains. Science. 2001; 291:1289-92.

Table 1 Click here to download Table Table 1.docx

Table 1. Assembly of the *Monopterus* genome

	Contig		Scaffold	
	**Size (bp)**	**Number**	**Size (bp)**	**Number**
N90	4,762	33,115	368,242	379
N80	8,655	23,414	775,515	254
N70	12,290	17,275	1,109,624	180
N60	16,188	12,785	1,519,751	128
N50	22,239	8,438	2,106,322	87
Longest	159,913	----	11,676,616	----
Total size	634,655,961	----	689,524,511	----
Total number(>=100bp)	----	117,579	----	62,978
Total number (>=2kb)	----	44,314	----	2,360

*The contig length was the final contig length after filling intra-scaffold gaps. Contigs with lengths shorter than 100bp were not included in the statistics.

 Click here to download Table Table 2.docx

Table 2． Assembly statistics for each chromosome

Chromosome	Chromosome length (Kb)	Scaffolds No.	Gene No.	Gene density (n/10Mb)
**1**	75908.7	33	2264	298
**2**	65103.9	32	2133	328
**3**	51637.3	21	1872	363
**4**	51162.1	30	1791	350
**5**	50080.0	27	1517	303
**6**	48093.1	27	1659	345
**7**	42410.1	29	1500	354
**8**	41999.7	30	1456	347
**9**	41928.7	23	1241	296
**10**	34690.8	30	1262	364
**11**	29285.5	23	1086	371
**12**	22774.4	23	879	386
**Total**	555074.3	328	18660	336

Figure 1

Click here to download Figure Figure 1.tif

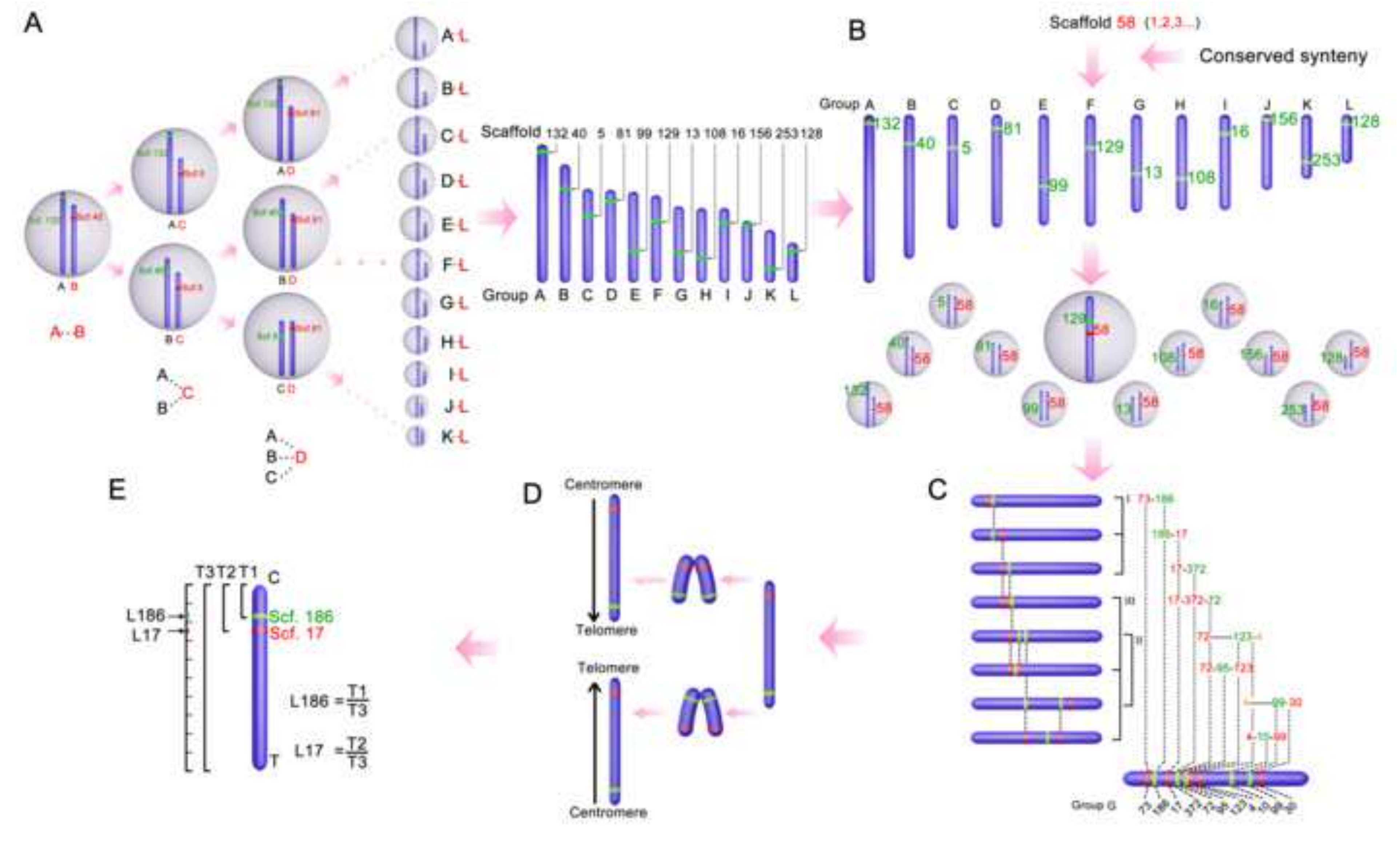

Figure 2

Click here to download Figure Figure 2.tif

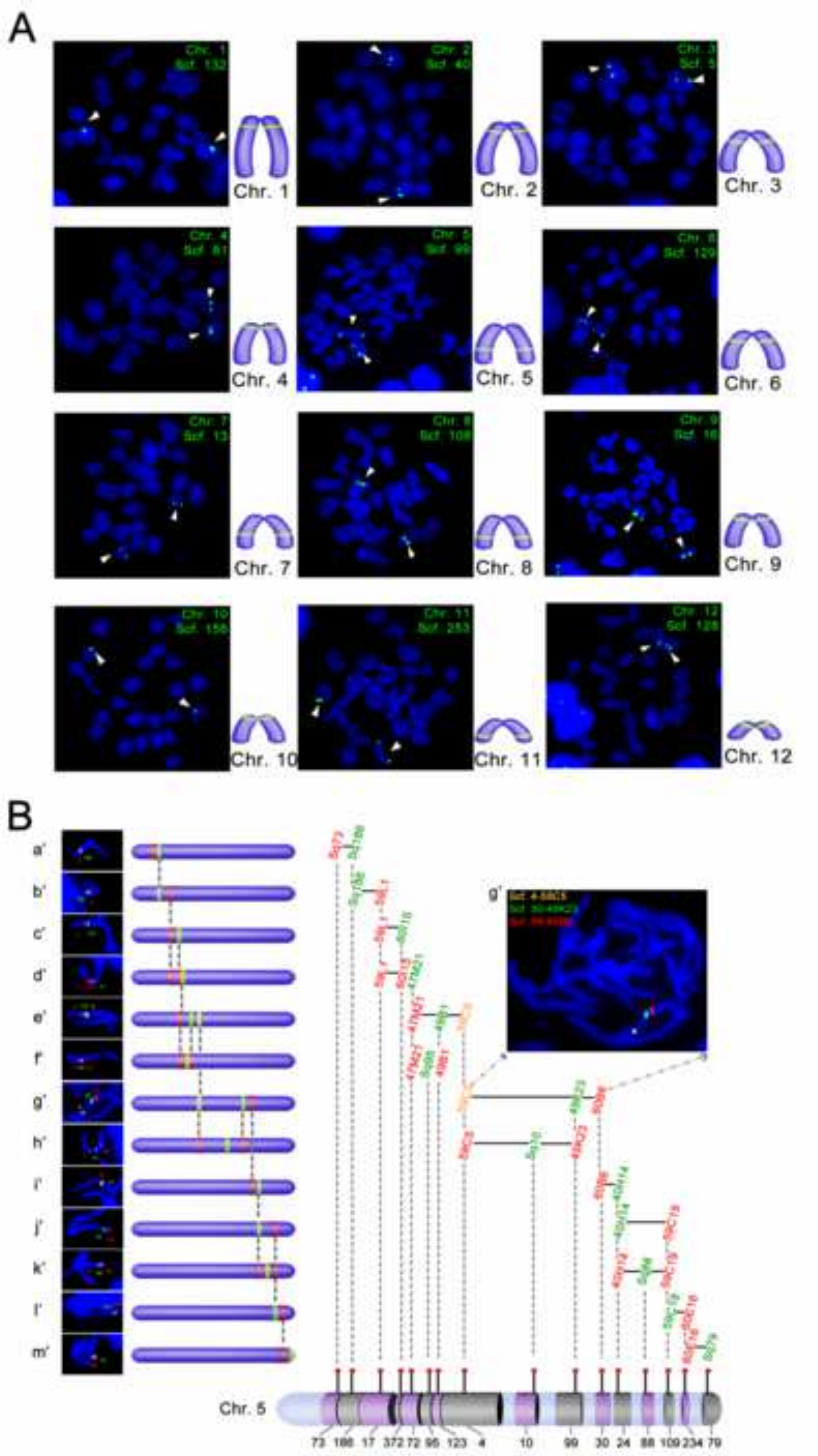

Figure 3

Click here to download Figure Figure 3.tif

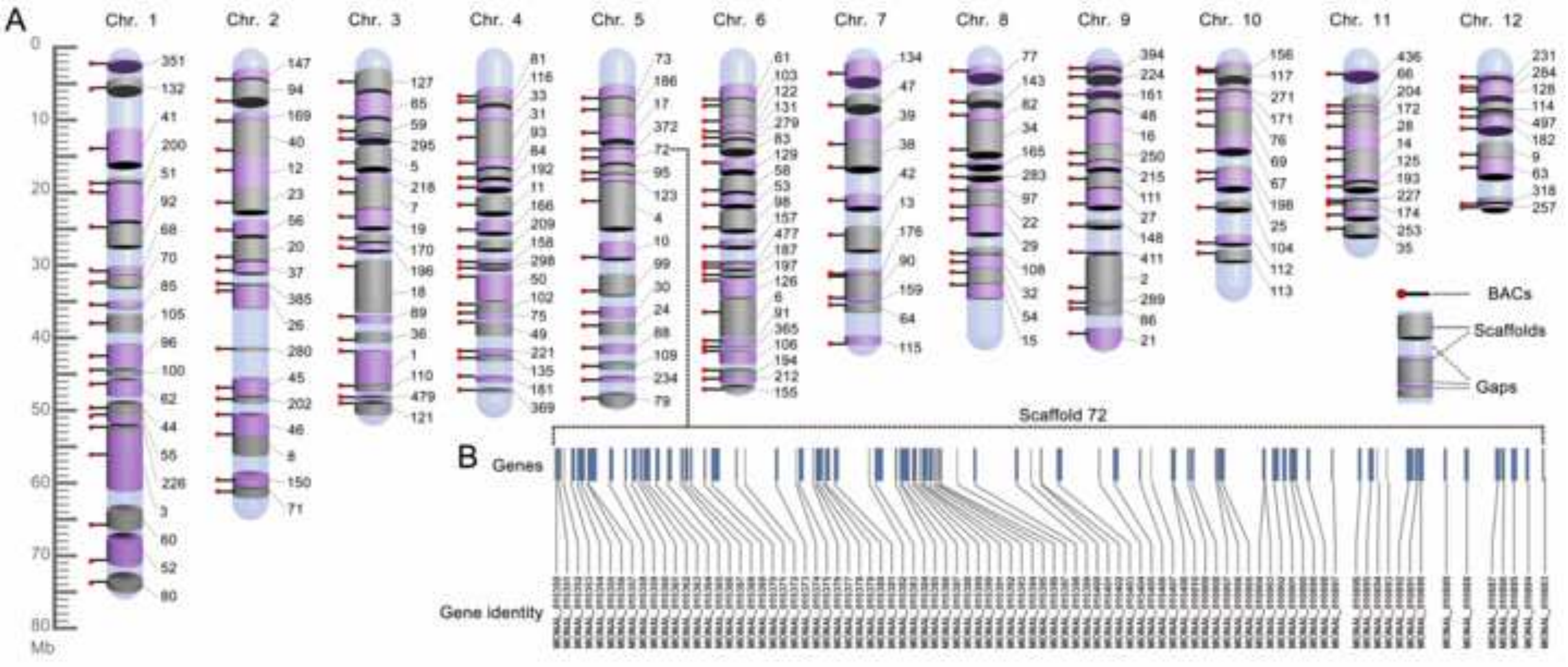

Figure 4

Click here to download Figure Figure 4.tif

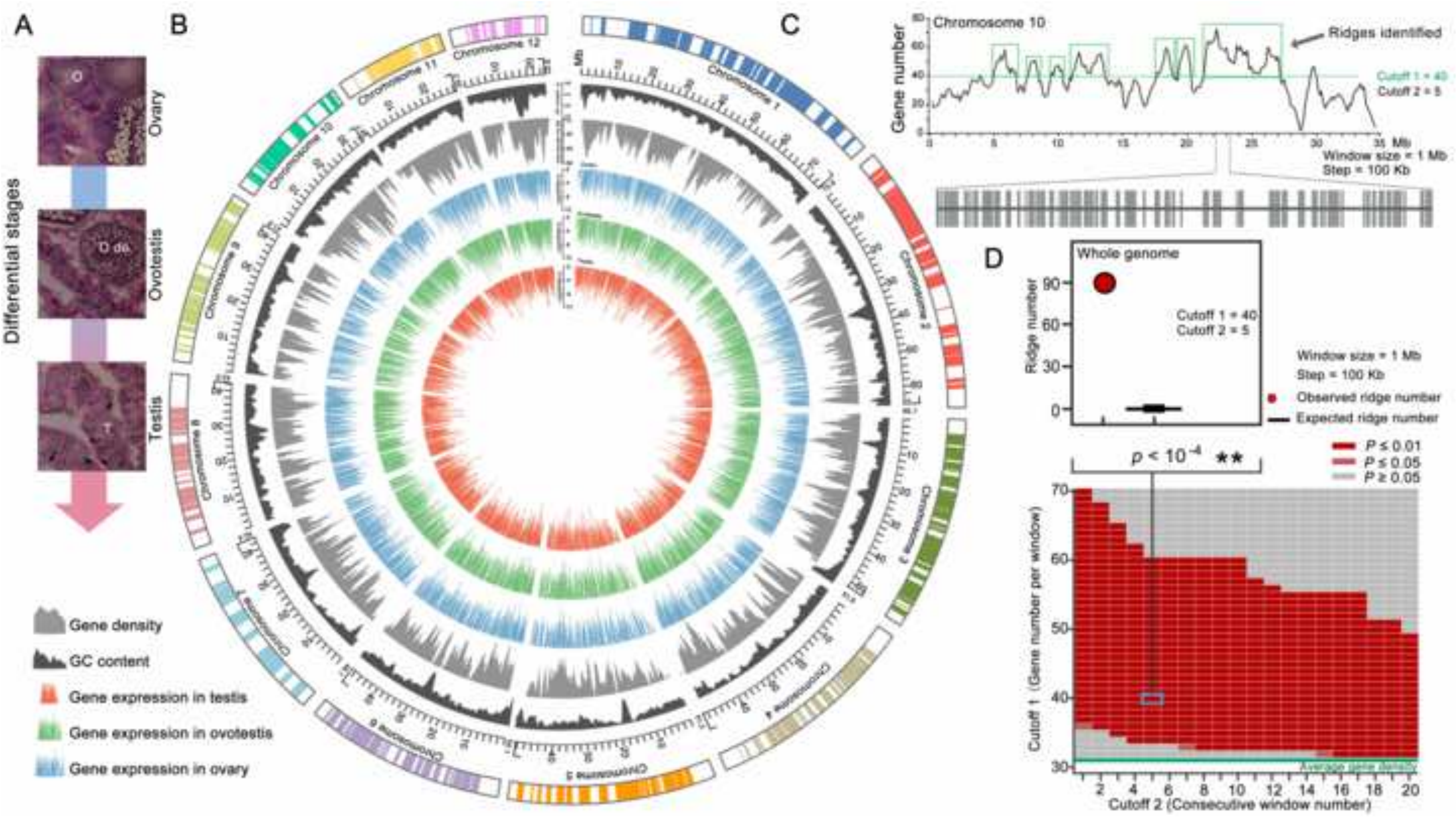

Figure 5 Click here to download Figure Figure 5.tif

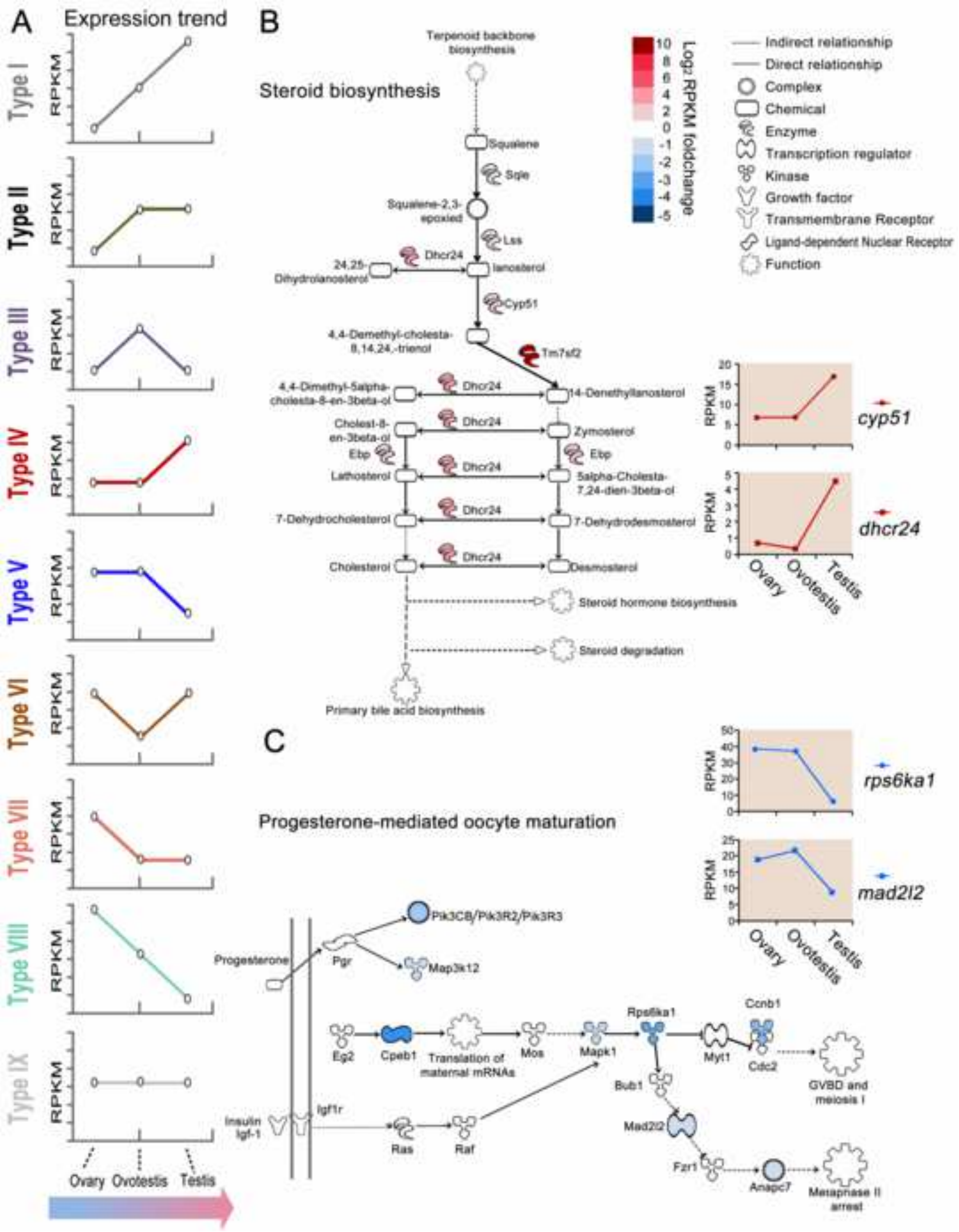

Figure 6

Click here to download Figure Figure 6.tif

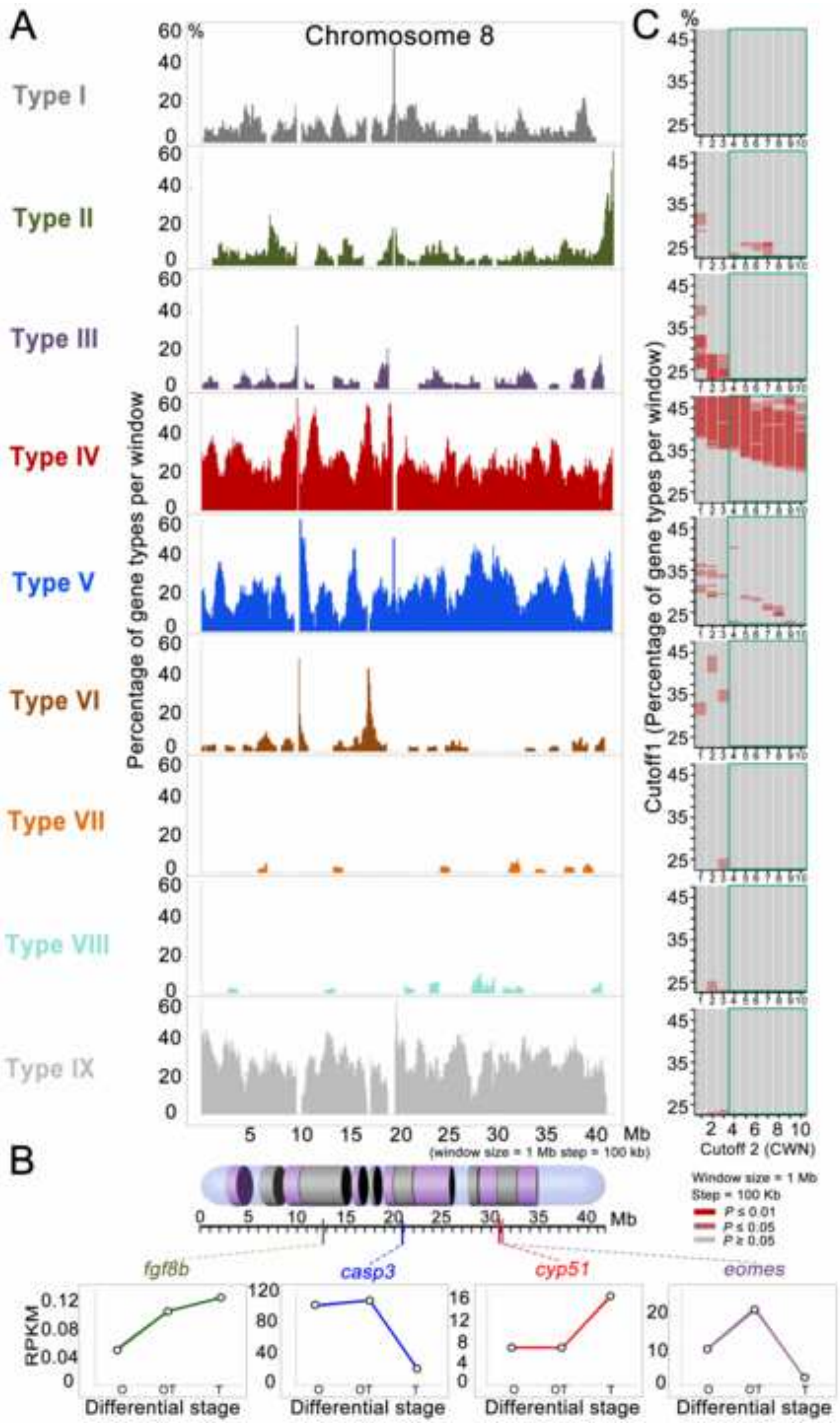

Figure 7 Click here to download Figure Figure 7.tif

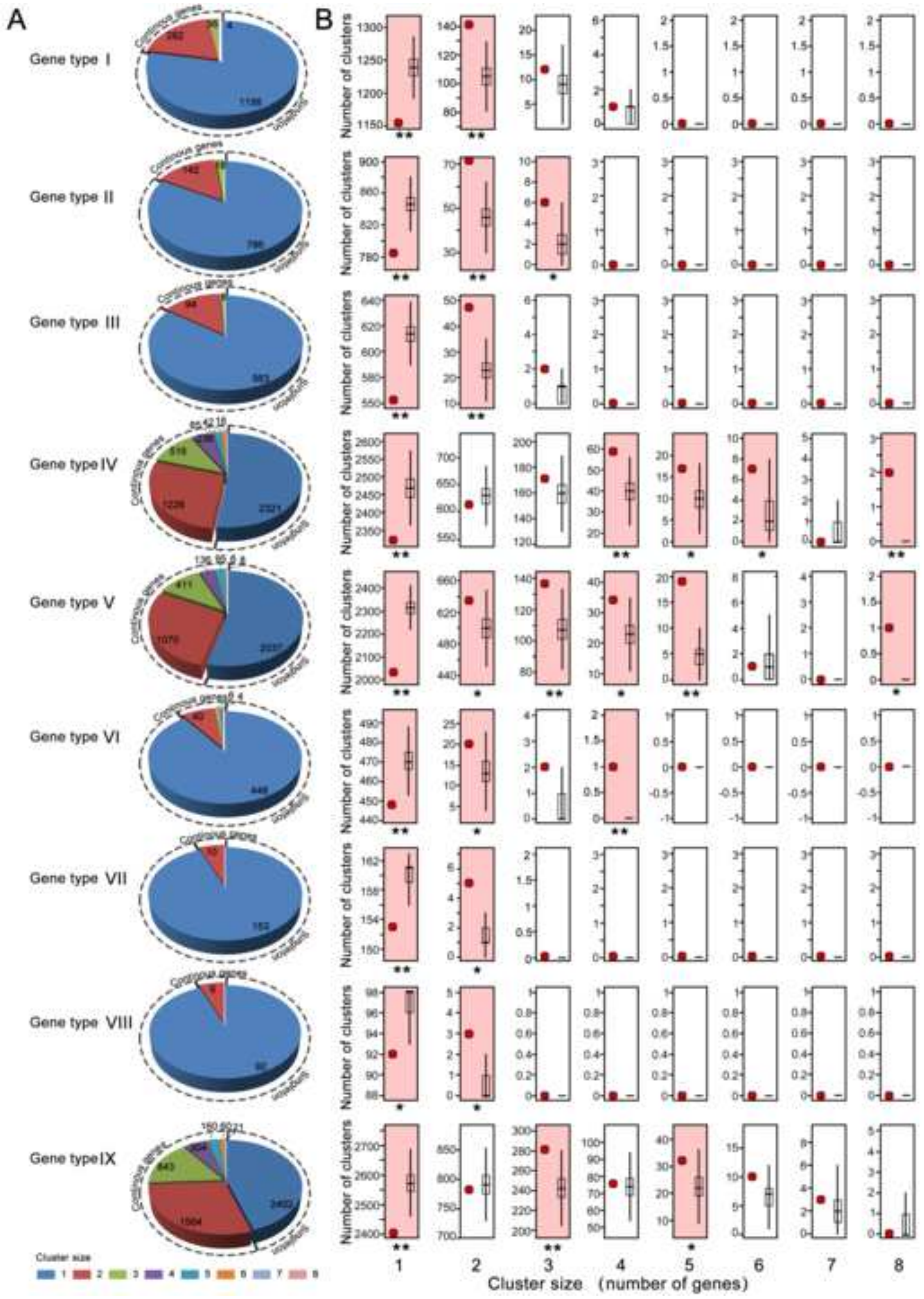

Figure 8 Click here to download Figure Figure 8.tif

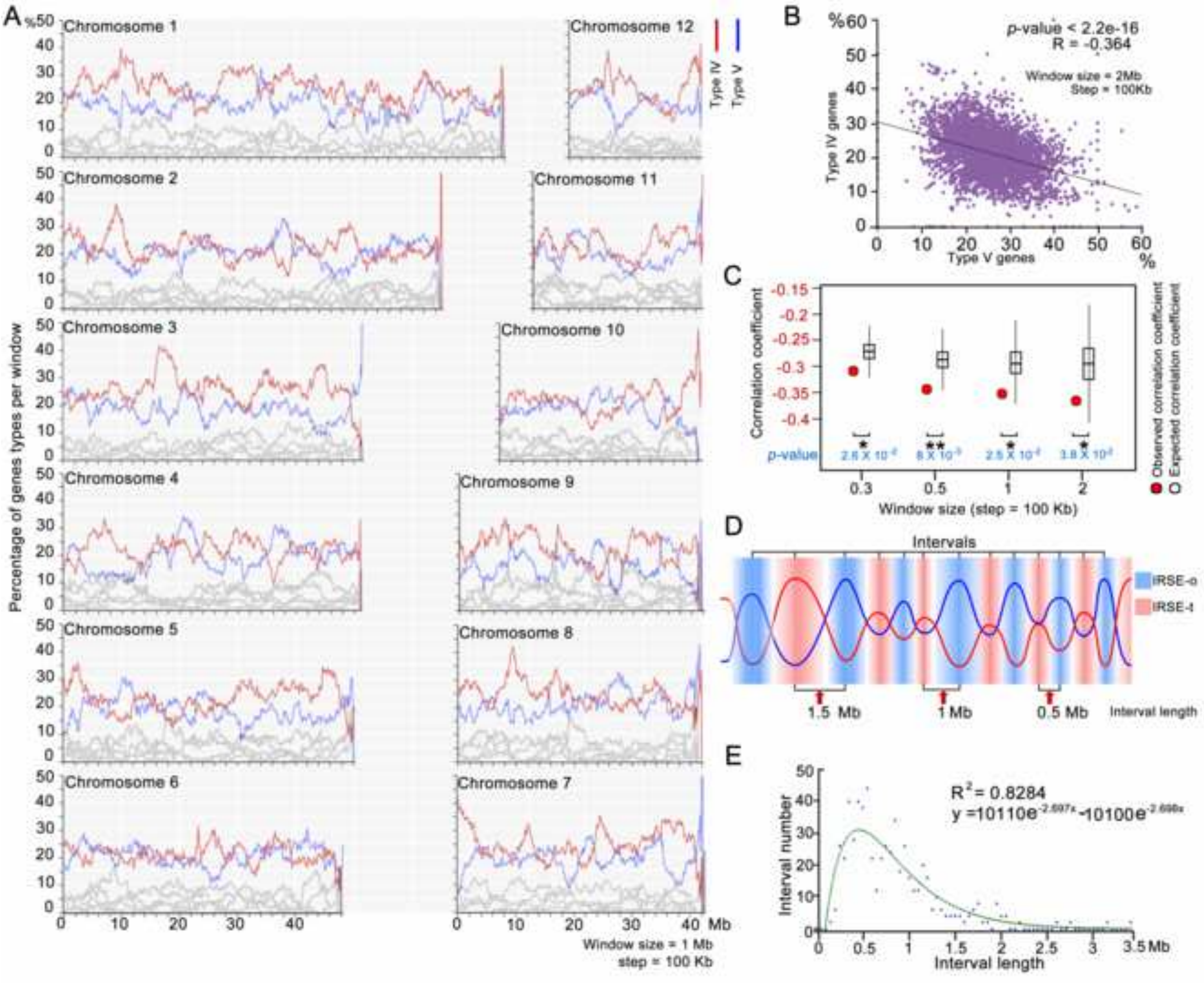

Figure 9

Click here to download Figure Figure 9.tif

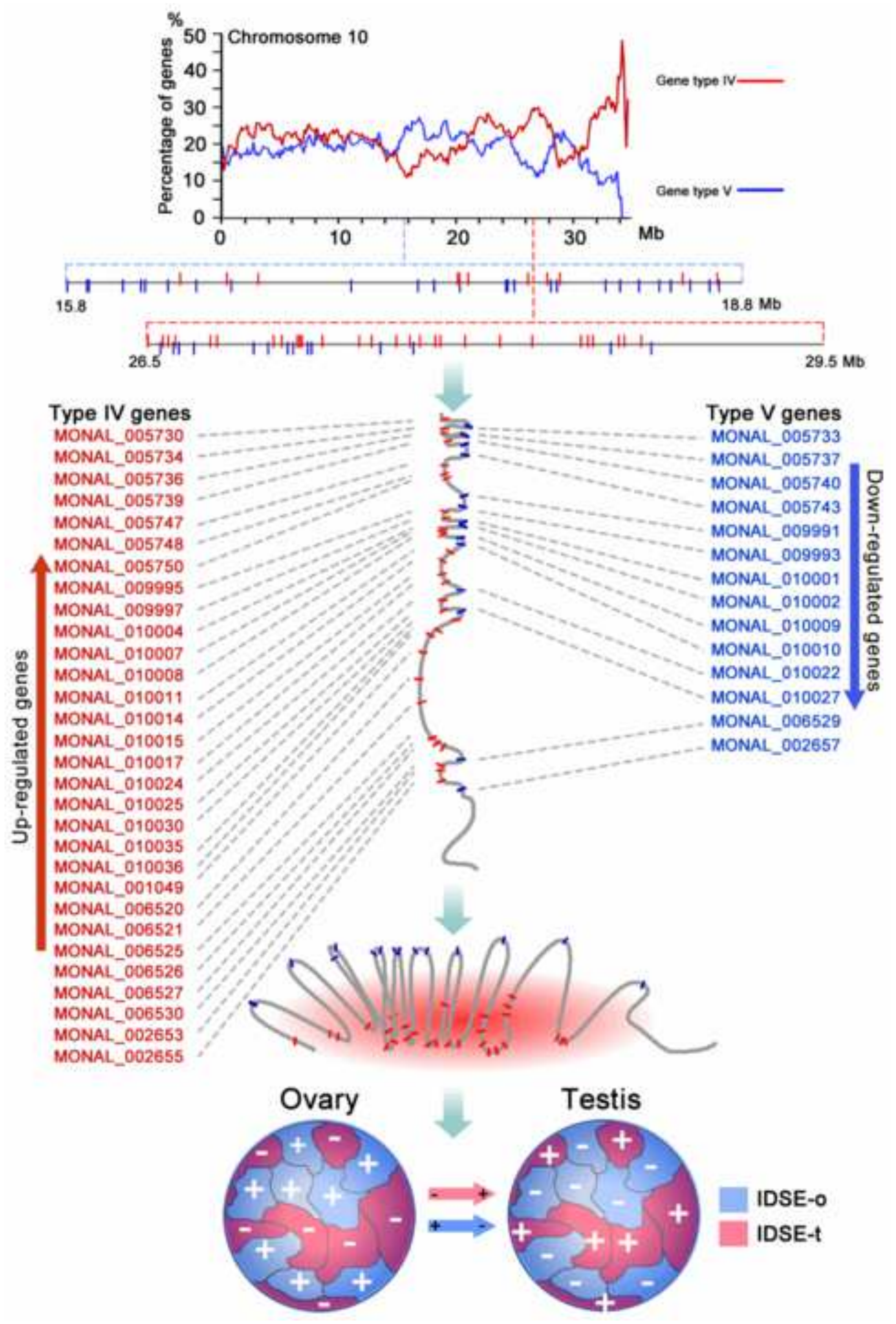

Click here to access/download
Supplementary Material
Additional files.docx

Personal Cover Click here to download Personal Cover cover letter.docx

Wuhan University
Since 1893
Laboratory of Molecular and Developmental Genetics, College of Life
Wuhan 430072, China
Tel: (0086)27-68756253, E-mail: rjzhou@whu.edu.cn

Editors
GigaScience

August 20, 2017

Dear Editor,

I am pleased to submit an manuscript entitled "**Chromosome-scale assembly of the *Monopterus* genome reveals a co-regulation landscape of interconvertible regions of sex during sex transition**" for your consideration for publication in *GigaScience*.

The teleost fish *Monopterus* is emerging as a new model for biological studies as its natural sex transition, small genome and evolution, besides enormous economic and magic medical values. For example, it contains high polyunsaturated fatty acid Omega-6. The most influential pharmacy monograph, the Bencao Gangmu, a compendium of materia medica, by pharmacist Shi-Zhen Li during the Ming Dynasty (AD 1368~AD 1644), recommended *Monopterus* as a natural drug with medicinal virtues to cure several kinds of diseases, such as facial paralysis, internal hemorrhoid hemorrhage, and pathogenic wind and dampness. Thus, it is a huge and new land in biomedical field.

Because of the unknown genome of the species, this work performs a large scale of *de novo* chromosome assembly by FISH walking assisted with conserved synteny (Cafs) after whole genome sequencing, in addition to reporting a new *de novo* chromosome assembly strategy, Cafs.

Using Cafs, a total of 18,860 genes are accurately mapped on chromosomes. Notably, we can find structure features of the genome, e.g. a nonrandom distribution of both genes and clusters along chromosomes based on the assembly, which cannot be found if only genome is sequenced, most often.

Underlying mechanisms of natural sex transition from female to male remain largely unknown. A key finding in this paper, using the assembly, is to unveil sex transition mechanism. We discover a co-regulation mechanism of interconvertible regions of sex-associated expression (IRSE) during sex transition. Two types of genes with opposite expression mode are coordinately expressed at an alternatively interconvertible manner during gonad transition, which tend to be chromosomally clustered separately. We further

character the IRSEs in a genome-wide scale.

The work provides new insights into a new kind of combinatorial regulation of gene expression in a higher chromatin organization, suggesting a new mechanism of sex determination at both transcriptional and chromatin organization levels. This is very an intriguing finding in biology.

The work unveils a coordination mechanism of genome structure and function, and highlights the importance of the fish as a model organism for biomedical research. I have also produced an eye-catching cover art for your consideration.

With these major findings, I am sure this is good topic for your journal. I am pleased to submit it for your consideration for publication in your journal.

The manuscript has not been submitted or is under consideration for publication elsewhere now. All authors agree to submit to your journal.

I look forward to hearing from you. Thank you for your consideration!

With best regards,

Rongjia Zhou, Ph.D.
Luojia Distinguished Professor
Genetics

PS,
May I suggest following potential reviewers:

Professor Meisheng Yi
Sun Yat-sen University
School of Marine Sciences
Zhuhai 519082, P.R.China
E-mail: yimsh@mail.sysu.edu.cn

Professor Songlin Chen
Yellow Sea Fisheries Research Institute
Chinese Academy of Fishery Sciences
Key Laboratory for Sustainable Development of Marine Fisheries
Ministry of Agriculture
Qingdao, China

Email: chensl@ysfri.ac.cn

Investigator Bing Su
Key Laboratory of Cellular and Molecular Evolution,
Kunming Institute of Zoology
Chinese Academy of Sciences
Kunming, China
E-mail: sub@mail.kiz.ac.cn

Professor Tian Dacheng
Nanjing University
College of Life Sciences
Phone:+86-025-83686406
Fax:+86-025-83592705
E-mail: dtian@nju.edu.cn
