# Supplementary material for: Chromosome-scale assembly of the Monopterus genome
Source: Gigascience. 2018 Apr 24;7(5):giy046. doi: 10.1093/gigascience/giy046 (PMC5946948; doi:10.1093/gigascience/giy046)
Supplement: GIGA-D-17-00210_Revision_1.pdf [file giy046_giga-d-17-00210_revision_1.pdf]

GigaScience
Chromosome-scale assembly of the Monopterus genome
--Manuscript Draft--

**Manuscript Number:**	GIGA-D-17-00210R1
**Full Title:**	Chromosome-scale assembly of the Monopterus genome
**Article Type:**	Data Note
**Funding Information:**	National Natural Science Foundation of China (31571280) — Professor Rongjia Zhou<br>National Natural Science Foundation of China (31471182) — Prof. Hanhua Cheng
**Abstract:**	Background: The teleost fish Monopterus albus is emerging as a new model for biological studies due to its natural sex transition and small genome, in addition to its enormous economic and potential medical value. However, no genomic information for the Monopterus is currently available.<br><br>Findings: Here, we sequenced and de novo assembled whole genome of the Monopterus, and report the de novo chromosome assembly by FISH walking assisted by conserved synteny (Cafs) for Monopterus. Using Cafs, 328 scaffolds were assembled into 12 chromosomes, which cover genomic sequences of 555 Mb, accounting for 81.3% of the sequences assembled in scaffolds (~689 Mb). A total of 18,860 genes were mapped on the chromosomes and showed a non-random distribution along chromosomes.<br><br>Conclusions: We report the first reference genome of the Monopterus and provided an efficient Cafs strategy for a de novo chromosome-level assembly of the Monopterus genome，which provides a valuable resource, not only for further studies in genetics, evolution and development, particularly sex determination, but also for breed improvement of the species.
**Corresponding Author:**	Rongjia Zhou, Ph.D.<br>Wuhan University<br>Wuhan, Hubei CHINA
**Corresponding Author Secondary Information:**	
**Corresponding Author's Institution:**	Wuhan University
**Corresponding Author's Secondary Institution:**	
**First Author:**	Xueya Zhao
**First Author Secondary Information:**	
**Order of Authors:**	Xueya Zhao<br>Majing Luo<br>Zhigang Li<br>Pei Zhong<br>Yibin Cheng<br>Fengling Lai<br>Xin Wang<br>Jiumeng Min<br>Mingzhou Bai<br>Yulan Yang

Powered by Editorial Manager® and ProduXion Manager® from Aries Systems Corporation

	Hanhua Cheng
	Rongjia Zhou, Ph.D.
**Order of Authors Secondary Information:**	
**Response to Reviewers:**	Point-to-point responses to reviewers<br><br>To Reviewer 1:<br><br>The manuscript contains valuable and interesting data/results, but the presentation lacks clarity and soundness in many places as well as does not meet scientific standards in a few cases.<br><br>Responses: Many thanks for your critical comments. We have revised the whole manuscript following your suggestions and scientific standards. We have also formatted the version as “Data Note”, not as a "Research Article". Following suggestions of Editor Hans Zauner (omit the second part of the paper on sex transition using RNAseq data, only include first part, genome assembly). As in the part two, the reviewer 2 suggested to repeat RNAseq data, however the biological replications need gonad samples which can only be obtained in reproductive season (June- Sept), this work cannot be performed in several months. Nevertheless, final paper in genome assembly for the Data Note is complete and meets standards of the Gigascience.<br><br>MAJOR COMPULSORY REVISIONS<br>GENERAL<br>The selection of cutoffs 1/2 is not clear and seems to be highly subjective. Essential data (eg. map positions of ridges and IRSEs) is missing. Terminology centrally to the manuscript is not clear (eg. "interconvertible", "interconvertible region", "interconvertible domain" and "interconvertible manner"). lines 47/372/418Clear overstatements: "we discovered a co-regulation mechanism", "found a co-regulation mechanism" and "showed a co-regulation mechanism". 306-310The features (GWPs) in this correlation analysis are mutually (inversely) dependent. Moreover it is not clear whether the given p-value has been corrected for multiple testing. 314-319The paragraph is conceptually/semantically not clear to me. What means "distribution pattern can be described in interconvertible regions", "IRSEs explain" and "emerge in an alternating, mutually interconvertible manner along the chromosome"? Fig8D and the reference therein to Fig6 are unclear. 321-331It is not clear (neither from the Methods) how IRSEs are being defined and mapped. Therefore the presented data are not traceable. What means "suggesting an obvious association" in the Results. The IDSE definition and the concept as well as Fig9 are also not clear hand should have in no way been placed in the Results.<br><br>Responses: The second part has been deleted as stated above.<br><br>MINOR ESSENTIAL COMMENTS<br><br>34/67<br>No data are presented to support "enormous/high" medical value of the fish. The reference to a medieval writing indicates at best a "potential" medical value.<br><br>Responses: Thanks, we have modified them as “potential<br>medical value”. (line 36/69 in the new version)<br><br>44<br>The terminology "types of genes" is misleading.<br>48/113"IRSE" vs. "IDSE"?<br><br>Responses: The second part has been deleted as stated above.<br><br>91<br>"NGS" means not necessarily "short length of the reads". This refers only to 2nd generation technologies.

	Responses: “NGS” has been replaced by “the second generation sequencing technologies”. (line 91 in the new version)<br><br>107<br>What are "complete genetic maps"?<br><br>Responses: "complete genetic maps" has been replaced by“high-density genetic map”. (line 109/110 in the new version)<br><br>110<br>Wording "chromosome-level assembly with 81.3% coverage of the sequenced genome" is not scientifically sound.<br>Responses: The sentence has been re-written as: “chromosome-level assembly covering 81.3% of the sequences assembled in scaffolds”. (line 114 in the new version)<br><br>113<br>"reasonably explains" is not scientifically sound.<br><br>Responses: The section has been deleted.<br><br>122<br>How "78.6 GB sequence dataset with a sequencing depth of 97-fold" was calculated?<br><br>Responses: We have added the sequence data description including the related methods (line 128 in the new version).<br>The genome size was estimated from k-mer analysis (806 Mb). The sequencing depth can be calculated from the total number of sequencing bases (78.6 GB) and the genome size (806 Mb) as 78.6 GB/806 Mb=97.<br><br>128<br>Not "mapping to the genome" but mapping to the reference sequence of the genome.<br><br>Responses: Thanks, we have corrected it as “mapping to the reference sequence of the genome”. (line 163 in the new version)<br><br>135<br>"DNAs"?<br><br>Responses: DNA transposons, TEs are assigned according to their mechanism of transposition, which can be described as either copy and paste (retrotransposons) or cut and paste (DNA transposons). Retrotransposons are commonly grouped into three main orders: LTRs, SINEs and LINEs. DNAs refer to the DNA transposons. We have replaced the “DNAs” with “DNA transposons”. (line 191 in the new version)<br><br>136<br>"acted in concert"?<br><br>Responses: We have corrected it as “which is involved in guardians for genome defense and germline stability via piRNAs”. (line 193 in the new version)<br><br>140<br>"the database"?<br><br>Responses: the local Monopterus genome database, we have modified it. (line 199 in the new version)<br><br>156-176<br>What means "group", "efficient combinations", "landmarks or the sequences", "order of another scaffold", "each scaffold"?<br><br>Responses: We have modified them as:<br>“linkage group” refers to the sequence sets of a chromosome.<br>“efficient combinations” replaced by “probes combinations” (line 241 in the new

version)
“landmarks or the sequences” replaced by “each with a molecular landmark” (line 243 in the new version) “The order of another scaffold” replaced by “The location of a new scaffold” (line 255 in the new version)
“each scaffold” replaced by “all scaffolds on chromosomes”. (line 259 in the new version)

180
Rephrase "A scaffold was mapped on each chromosome by FISH, which was used as a landmark" and describe clearer how the landmark scaffolds were selected.

Responses: We have modified it and added a paragraph to describe the landmark scaffolds.
Line 236-245:"We first prepared probes of BACs from sequenced clones and PCR fragment pools (8-15 sequences and covers a total length of 20-30 kb on a scaffold) representing scaffolds for chromosome FISH, and performed synteny analysis of these scaffolds by comparing with the fish species (medaka, sticklebacks, Tetraodon and Monopterus). Second, from the synteny information of the homologous sequences of these scaffolds in the three fish species，probe combination mapping was used to determine 12 linkage groups, corresponding to 12 chromosomes, each with a molecular landmark (Figure 1A). Briefly, group A and B were first discriminated by two unlinked scaffolds labelled with two different colours. If another scaffold was unlinked to the previous two scaffolds, the third scaffold was identified as a marker of group C. "

183-194
Where do the numbers 186/108142 come from and how they relate to each other? The sentence "142 of the scaffolds were further assembled into 12 chromosomes" is misleading. What means "issue should be further corrected"?

Responses: We have re-written the sentences clearly in the section “De novo chromosome assembly by Cafs-strategy.”
186 refers to the number of the scaffolds mapped by FISH;
These 186 scaffolds can be divided into two categories according to the results of the synteny analysis:
108 scaffolds whose homologous sequences had the consistent synteny in at least two of the three closely related fish species, while the rest 76 scaffolds didn’t show consistent synteny.
For the 108 scaffolds whose location could be predicted by the synteny analysis, 99 of which were confirmed by FISH, while the rest 9 whose FISH results were inconsistent with their prediction, which may be the result of the rearrangement of the genome in the genome evolution. Thus, the accuracy of the synteny prediction is 99/108=92%.
142 refer to the number of the scaffolds mapped by synteny prediction only, without FISH confirmation. FISH work remains to be further performed in the future. (line 285-298 in the new version)

196/199
" We finally integrated 328 scaffolds into 12 chromosomes" is lab jargon. You assembled 328 scaffolds into reference sequences for 12 chromosomes. The same refers to "sequenced genome of 683 Mb with 97.6X". You sequenced a genome and assembled a reference sequence of 683 Mb that is (most likely - please provide the overall length of assembled reads eg. as supplement) 97.6x covered by aligned reads.

Responses: We have re-described the sentences as,“We finally assembled 328 scaffolds into reference sequences for 12 chromosomes”,
“accounting for 81.3% of the sequenced genome of 683 Mb with 97.6X” was replaced by “accounting for 81.3% of the sequences assembled in scaffolds (689.5 Mb)”.
The overall length of assembled reads was shown in table 1.
(line 300/303 in the new version)

203/207
What mean "efficient" and "most attractive"?

Responses: We have deleted the words and modified. (line 207 in the new version)

Powered by Editorial Manager® and ProduXion Manager® from Aries Systems Corporation

	213-217<br>What means "deep transcriptome sequencing"? It must be explained at least in Methods. What means "distribution patterns of genes on chromosomes in accordance with their expression profiles"? The statement "significant positive correlation between the gene density and expression" should be better explained/supported in the main text. The terms "gene density" and "expression" are not introduced Figures show "Gene number". The reference to Fig4B is not helpful. R values should be given together with the p-value in the main text. For any p-values the respective test should be indicated.<br><br>Responses: The second part has been deleted as stated above.<br><br>227<br>The statement "distribution pattern of the gene density was consistent with the corresponding GC content" is not supported by data.<br><br>Responses: In the previous version, FigS9B showed the spearman correlation test for GC content and gene density, and the figure has been changed into Fig.S9 in the new version. (line 324 in the new version)<br><br>234-239<br>Define "consecutive window numbers" unambiguously. Not "distribution pattern of the ridges" but numbers of ridges have been defined. What means "significantly low", "(ridge numbers in random ≥ observations", "highly significant difference in ridges with high gene density"?<br><br>Responses: We have revised them as:<br>“consecutive window numbers” means “the numbers of consecutive moving windows in which each gene numbers have a lower limit of cutoff 1”. We have added the detailed description in brackets.<br>“distribution pattern of the ridges” is replaced by “the numbers of the ridges”.<br>“significantly low” is replaced by “very low”.<br>“ridge numbers in random ≥ observations” is replaced by “ridges numbers under a random permutation ≥ ridges numbers in the Monopterus genome”.<br>“showed a highly significant difference in ridges with high gene density along chromosomes in comparison with random permutations of gene positions” is replaced by “the results showed that there were significant differences in ridges numbers between the Monopterus genome and random permutations of gene positions”. (line 341-345 in the new version)<br><br>245<br>The statement "These analyses suggest that the ridge pattern on the chromosomes probably represents a higher-order structure in the genome" belongs to Discussion not to Results.<br><br>Responses: We have put the sentence in the last section. (line 351 in the new version)<br><br>250/739<br>Clarify the meaning of "key gene types during gonad transition" and "gene regulation pathways in gonad transition".<br>255-259<br>What means "clearly"? Please provide gene numbers/fractions for each type as suppl table. What means "the GO terms in the biological process category were over-represented in all types"?<br>281-283<br>What means "high degree" when comparing types V and II as well as "clustered on chromosomes in a large-scale"?<br>286-287<br>What means "each type of gene" and "continuous degree of gene clustering"?<br>292-296<br>How the p-values were obtained (corrected for multiple testing) and how they relate to "*/**" in Fig7B. 336What means "complete genome information" - a complete and error free as possible reference sequence?<br>337-342

	Please provide a justification/reference for the statement.<br>343<br>What means "the other 22 species"?<br>345<br>Please provide data for "efficient and cost effective".<br>348 The statement "81.3% coverage of the sequenced genome is most likely an overestimation.<br>Responses: The part and discussion sections have been deleted as the data note format.<br>353<br>Provide data for "improve the understanding of the mechanisms of genome". 355-356What means "favoured genes" and "QTLs of economic importance".<br>372<br>Clear overstatement: "found a co-regulation mechanism". 375-377<br>Clarify what means "cluster" in this context and whether in your concept an IDSE is formed by a subset of genes of a "ridge"383What means "IRSE-o should occupy a different topological domain from the IRSE-t".<br><br>394<br>Provide data for "IDSEs are closely associated with sex determination".<br><br>Responses: The second part has been deleted as stated above.<br><br>428<br>(line 411 in the new version)To my understanding no "animal experiments" have been performed.<br><br>Responses: Yes, we have performed animal experiments, kidney tissues were dissected, which has been added in the line 265 in the new version.<br><br>585<br>the statement "three tissues (ovary, ovotestis, and testis)" is not corre ct. How many biological replicates were identified? 596-597<br>Clarify whether "RPKM<0.1" refers to one or both analyzed states. What means "FDR (false discovery rate) < 0.05 (p-value<0.01)"?<br>741-745The statement "Differential expression is identified by fold change of RPKM (≥2)" contradicts those in Methods. What means "p-value = 0.041 (DAVID, version 6.7). Fisher's exact test (FDR = 0.05) was used to assess the enrichment"?<br>752-764<br>The figure as such does not show "Clustering patterns". What are "typical genes" and "intensity of clusters"? "and at least" should read as "in at least"?<br>766-778Phrasing "consecutive gene clusters of co-regulated genes" is misleading and should read as "clusters of consecutive co-regulated genes". Significance is not explained (test, thresholds, multiple test correction). 786What means "alternating sine curves"?<br>793<br>Are the p-values corrected for multiple testing?<br>803-810The legend does not sufficiently explain what is shown in the different panels.<br><br>Responses: The second part has been deleted as stated above.<br><br>Tab 1<br>What "*" refers to? What means "final contig length after filling intra-scaffold gaps"? Does the overall scaffold length include Ns?<br><br>Responses: We have modified the description of Table 1. The final contig size are the size after filling gaps. "contig length" is replaced by "contig size".<br><br>Tab2<br>"Chromosome length" is incorrect/misleading.<br><br>Responses: "Chromosome length" is replaced by "Chromosome size".<br><br>Fig5B

Cyp51 in the left panel is shaded according to log2(FC)=2 (ie. FC=4) but the right panel shows a FC<3.
FigS12
What means "gene numbers in each catalog"?
FigS13The title does not cover type IX.

Responses: The second part has been deleted as stated above.

DISCRETIONARY COMMENTS
39
"Cafs" is not clear here.

Responses: "Cafs" was the abbreviation of “de novo chromosome assembly by FISH walking assisted by conserved synteny”. The detailed strategy of Cafs were in the section “De novo chromosome assembly by Cafs-strategy”. (line 39 in the new version)

87-90
Mixing "genetic" and "physical" maps is confusing for a non-geneticist reader.

Responses: Yes, our statement is not clear and accurate. Both genetic map and physical map can assist chromosome assembly, but they are different. We have modified them by using the word in different sentences. (line 89-92 in the new version)

98
"BAC/fosmid paired end sequencing from large-insert libraries" is a tautology.

Responses: Yes, we have described it as “BAC/fosmid paired end sequencing”. (line 101 in the new version)

106
"map on chromosomes" map of chromosomes?

Responses: We have modified it. (line 109 in the new version)

128
Not "Sanger sequence technology" but "Sanger sequencing technology".

Responses: Thanks, we have modified it. (line 164 in the new version)

146
Replace "Chromosome-level assembly strategy without a genetic map" by "Chromosome assembly by FISH walking assisted by conserved synteny - strategy outline", outline the strategy in this section and provide the details for the actual project in the next section.

Responses: Thanks, we have modified it, and provide the details for the actual project in the section following the Data Note format. (line 230 in the new version)

346
Do "gene functions" have a "position in the genome"?

Responses: The second part has been deleted as stated above.

376
Introduce abbreviations only once in the main text.

Responses: We have provided the abbreviations section (line 385 in the new version).

[19] is rather outdated.
TabS9 is incomplete.

Responses: The second part has been deleted as stated above.

Powered by Editorial Manager® and ProduXion Manager® from Aries Systems Corporation

To Reviewer #2:

This manuscript describes both the genome assembly of the swamp eel, and some transcriptomics experiments relating to its sex-reversal physiology.

The genome assembly process follows a straightforward short-read shotgun recipe, but is supplemented by in situ hybridization of scaffold probes to arrive at a chromosome-level assembly. This is presented as a novel strategy, which I am uncertain is entirely justified. It is, however, a worthwhile effort. The supposed novelty is entirely related to the pre-selection of co-hybridization probes based on synteny information. In general, I would be very cautious about using comparative evidence to finish a chromosome-level assembly, as any subsequent comparative genomics studies run the risk of circular reasoning. In this case, it appears the comparative evidence is mostly only used as an initial screening, and it does not directly inform the assembly process - but please see my issue 6 below.

Responses: Many thanks for your critical comments and kind suggestions. Yes, the cafs consists of several methods which are not novel. Nevertheless, their combinations, especially synteny and FISHing were adopted, are certainly efficient for chromosome-level assembly. It is indeed feasible, assembling 81.3% of scaffold sequences, and the accuracy is high. Synteny analysis provided an initial screening, are cost-less and take less time for final FISHing assembly process. Final assembly have been confirmed by FISHing, except approximately 8% (~11) of the scaffolds could not be resolved by the conserved synteny prediction due to possible rearrangements in the Monopterus lineage. This issue should be further corrected in future experiments.

Several issues about the assembly part I would ask the authors to address:
1. Is it correct that the assembly is based on multiple discrete scaffolding procedures (SOAPdenovo, l. 456, and SSPACE, l. 459) using the same data? Does this not increase the risk of artifacts?

Responses: The Monopterus genome was assembled by constructing 170bp, 500bp, 800bp, 2kb, 5kb, 10kb, 20kb and 40kb insert size genome library for sequencing respectively. The sequencing data derived from 2kb, 5kb, 10kb and 20kb insert size library was used to assemble scaffolds by SOAPdenovo, and the data derived from 40kb insert size library was used to assemble scaffolds by the SSPACE. The original description was not clear and we have modified the statement in the new version. (line140-148 in the new version)

2.Only ~79% of the expected genome size is captured in contigs (634.7 Mbp of 806 Mbp). Therefore, presumably, these contigs contain abundant collapsed repeats, affecting an analysis of repetitive content (l. 131-138, fig S4) Repeat abundance will likely be underestimated. (line 188-195 in the new version).

Responses: The genome size is 806Mb estimated by K-mer, but only 634.7 Mb was captured in contigs based on the data of second generation sequencing. This most likely results from a lot of repetitive sequences in the genome. The incomplete assemble is the limitation of the second-generation sequencing technology. The repetitive sequences we analyzed are based on the assembled genome. Thus, as you said, repeat sequences would be underestimated. We have discussed the fact in the version.

3.Line 136: please rephrase 'they acted', as no evidence on piRNAs is presented. （line 194 in the new version）

Responses: Several papers have reported that the piRNAs derived from LINE are very closely related with genome stability. We have just discussed the fact that LINEs was greater than that found in other teleost fish (2-5%), which were involved in guardians for genome defense and germline stability via Piwi-interacting small RNAs (piRNAs) [23].

Powered by Editorial Manager® and ProduXion Manager® from Aries Systems Corporation

4.No completeness analysis of the assembly/gene prediction is presented. Please include a BUSCO analysis of the predicted gene set.

Responses: We have added BUSCO (line 167-173 in the new version) analysis to evaluate the completeness of the gene set.

5.Line 142 /fig S5: 'The gene sizes were similar to those of other teleost fish'. In fact, the figure shows that the Monopterus gene set contains many more short genes and CDSs than other teleosts. Exon lengths are comparable. This suggests truncated genes or gene predictions. (line 214 in the new version)

Responses: BUSCO analysis showed that there are some numbers of fragmented and missing BUSCOs, but very low.

6.FISH walking (l. 180-203): Is it correct that no FISH evidence is used to assign 142 scaffolds to chromosomes (l. 191)? How will placing these scaffolds using comparative evidence only affect comparative analyses? (line 263-307 in the new version)

Responses: the data of 142 scaffolds were from comparative genomics analysis, not from FISHing experiments. We have discussed this issue, pointed out the possible error rates and problems. (line 295-298 in the new version)

7.Figure 3B: I do not see the added value of the visualization of gene prediction for this single scaffold. Similarly for figure 4C.

Responses: We have added the start location information of genes on the scaffold 72 in Figure 3B, and added the regions start and end location on the chromosome 10 in figure 4C (figure 4B of the new version).

8.Figure 3A and 4B: The chromosome sizes here add up to 555 Mbp, which is also the size of scaffolds assigned to chromosomes (l. 198) but much less than the expected genome size of 806 Mbp. If these sizes are entirely based on placed scaffolds, why do these illustrations still contain gaps?

Responses: For Figure 3A, the previous scale was really problematic. So we have changed the scale. We mapped the probes based on the ratio of the distance of the probe to centromere to the total length of the chromosome. Thus, we marked the scale with the ratio.
The Figure 4B (Figure 4A in the new version) was confused because of the legend for this figure was not clear. When we packaged the scaffolds into chromosomes, the gap between every two adjacent scaffolds was filled with 50 kb of Ns. Our analysis of the GC content and the gene density were based on this packaged chromosomes, but not the sequences of the outermost scaffold location. Therefore, we have added a new physical map to the Circos plot, which corresponds to the analysis of GC content and gene density.

9.Related: I assume the coloured block in the outer ring of fig 4B are placed scaffolds, and the white spaces gaps. How can these gaps then contain genes, have a GC content, and gene expression (inner rings)?

Responses: Please see the point 8 above.

The second part of the paper addresses sex transition, based on RNA-seq data. I have not reviewed the downstream analyses and conclusions in detail, as I believe the initial RNA-seq data is both insufficient and not analyzed appropriately:
10.There is no description of the RNA-seq raw data. How many reads per sample, etc.?
11.There is no mention of biological replication, therefore the entire analysis appears to be based on one sample per developmental stage - insufficient to draw strong conclusions from.
12.Differential expression analysis is not described in sufficient detail (l. 594-598). These do not appear to be industry-standard statistical analyses. In any case, without replication, how does one test for statistical significance of differential expression?

Powered by Editorial Manager® and ProduXion Manager® from Aries Systems Corporation

	13.RPKM normalization is usually deprecated, unless it can be argued that it is suitable. In this case, comparing the tissues requires major assumptions anyway, as they do not appear to be intrinsically comparable. I have attached density plots of the gene expression values in the three cases. This is after normalization: with successful normalization, the plots would align. In this case, it is far from clear this is even possible. I do not see how in this case, expression in testis can be related to the other samples. My quick scatterplots of gene expression show a similar picture.<br>14.I therefore do not see how these data can be used to reliably infer the patterns shown in fig 5A. (Related, l. 252: these classes/'types' are not differentially expressed themselves, they correspond to genes that are 'differentially expressed' in any one sample compared to the other two). The important/large classes IV and V are probably mostly populated by genes affected by noise/the effects I describe in issue 13.<br>15.Most statements about gene expression or its classification are based on very low expression, which especially in this case (no replication, poor normalization) is not reliable. For instance: fig S9 correlations (or are these log-values?), dhcr24 (l. 265, fig 5B), fgf8b (fig 6B).<br><br>Responses: As for the second part, we have formatted the version as “Data Note”, not as a "Research Article". Following suggestions of Editor Hans Zauner (omit the second part of the paper on sex transition using RNAseq data, only include first part, genome assembly). As in the part two, you suggested to repeat RNAseq data, however the biological replications need gonad samples which can only be obtained in reproductive season (June- Sept), thus this work cannot be performed in several months, although re-analysis the RNAseq data by credible FPKM not previous RPKM normalization show the RNAseq are correct. Nevertheless, final paper in genome assembly for the Data Note is complete and meets standards of the Gigascience.
**Additional Information:**	
**Question**	**Response**
Are you submitting this manuscript to a special series or article collection?	No
**Experimental design and statistics**<br><br>Full details of the experimental design and statistical methods used should be given in the Methods section, as detailed in our Minimum Standards Reporting Checklist. Information essential to interpreting the data presented should be made available in the figure legends.<br><br>Have you included all the information requested in your manuscript?	Yes
**Resources**<br><br>A description of all resources used, including antibodies, cell lines, animals and software tools, with enough information to allow them to be uniquely identified, should be included in the Methods section. Authors are strongly encouraged to cite Research Resource Identifiers (RRIDs) for antibodies, model organisms and tools, where possible.<br><br>Have you included the information	Yes

requested as detailed in our Minimum Standards Reporting Checklist?	
**Availability of data and materials**<br><br>All datasets and code on which the conclusions of the paper rely must be either included in your submission or deposited in publicly available repositories (where available and ethically appropriate), referencing such data using a unique identifier in the references and in the “Availability of Data and Materials” section of your manuscript.<br><br>Have you have met the above requirement as detailed in our Minimum Standards Reporting Checklist?	Yes

Powered by Editorial Manager® and ProduXion Manager® from Aries Systems Corporation

Manuscript Click here to download Manuscript MS-datanote-Zhou.docx

Chromosome-scale assembly of the Monopterus genome

Running title: The Monopterus genome

Xueya Zhao[1], Majing Luo[1], Zhigang Li[1], Pei Zhong[1], Yibin Cheng[1], Fengling Lai[1], Xin Wang[1], Jiumeng Min[2], Mingzhou Bai[2], Yulan Yang[2], Hanhua Cheng[1]*, Rongjia Zhou[1]*

[1]Hubei Key Laboratory of Cell Homeostasis, Laboratory of Molecular and Developmental Genetics, College of Life Sciences, Wuhan University, Wuhan 430072, P. R. China

[2]BGI-Shenzhen, Shenzhen 518083, P. R. China

*Corresponding authors: Professors Rongjia Zhou and Hanhua Cheng, College of Life Sciences, Wuhan University, Wuhan 430072, P. R. China, Fax: 0086-27-68756253, E-mail: rjzhou@whu.edu.cn, hhcheng@whu.edu.cn

Abstract

Background: The teleost fish *Monopterus albus* is emerging as a new model for biological studies due to its natural sex transition and small genome, in addition to its enormous economic and potential medical value. However, no genomic information for the *Monopterus* is currently available.

Findings: Here, we sequenced and de novo assembled whole genome of the *Monopterus*, and report the *de novo* chromosome assembly by FISH walking assisted by conserved synteny (Cafs) for *Monopterus.* Using Cafs, 328 scaffolds were assembled into 12 chromosomes, which cover genomic sequences of 555 Mb, accounting for 81.3% of the sequences assembled in scaffolds (~689 Mb). A total of 18,860 genes were mapped on the chromosomes and showed a non-random distribution along chromosomes.

Conclusions: We report the first reference genome of the *Monopterus* and provided an efficient Cafs strategy for a *de novo* chromosome-level assembly of the *Monopterus* genome，which provides a valuable resource, not only for further studies in genetics, evolution and development, particularly sex determination, but also for breed improvement of the species.

Key words: whole-genome sequencing, genome assembly, chromosomes, fish

Data Description

Background

The freshwater fish *Monopterus albus* taxonomically belongs to the teleost family Synbranchidae of the order Synbranchiformes. This fish is distributed mainly in southern and eastern Asia, in northern Australia and in the southeastern United States [1]. *Monopterus* is an economically important species for fish production because of its high nutritional value (e.g., high polyunsaturated fatty acid omega-6 levels) and potential medical value. The most influential Chinese pharmacy monograph, the Bencao Gangmu, a compendium of materia medica written by the pharmacist Shi-Zhen Li during the Ming Dynasty (AD 1368~AD 1644), recommended *Monopterus* as a natural drug with medicinal virtues to cure several types of diseases, such as facial paralysis, internal haemorrhoid haemorrhage, and other pathogenic conditions described as being influenced by wind and dampness in Traditional Chinese Medicine.

As an emerging model species in development, genetics and evolution [2], *Monopterus* has the attractive feature of undergoing a sex transition from female to intersex to male during its life [3]. This discovery may have considerable theoretical significance in sex determination [4]. *Monopterus* has a small genome size (~800 Mb) and a minimum chromosome number (n = 12) among teleosts, whose chromosome numbers range from 12 to 223 [5]. In addition, all chromosomes of *Monopterus* are telocentric. Given that a third whole-genome duplication occurred in the whole teleost lineage compared to the two genome duplications that occurred in other land vertebrates [6-8], the speciation and sexual differentiation of *Monopterus* may provide new insights into vertebrate evolution. However, the mechanisms of sex determination in the species remain unknown.

Whole-genome sequencing will provide detailed genetic data for studies of genetics, development and evolution and for the genetic manipulation of *Monopterus*. However, no genetic map is currently available for this species. The whole-genome shotgun approach, with

high throughput and low cost, is based on a second-generation sequencing platform that makes the whole-genome *de novo* assembly of a species possible without the need for a physical map. However, the sequence data produced by the second-generation sequencing technologies are highly fragmented due to the short lengths of the reads. A number of methods for increasing the contiguity and accuracy of *de novo* assemblies have recently been developed. The read length generated from sequencing can be improved by a third-generation sequencing platform, such as single-molecule real-time (SMRT) sequencing, with raw reads of a mean length of 15 kb [9], and nanopore single-molecular sequencing, with raw reads of approximately 5-50 kb [10, 11]. However, major drawbacks of these techniques include relatively high error rates and high costs of sequencing. Some strategies for the assembly of a long scaffold have also been developed, for example, BAC/fosmid paired end sequencing, the long-read sequencing (LRseq) [12] approach, contiguity-preserving transposase sequencing (*fragScaff*)[13], and various assembly algorithms [14, 15]. Recently, chromatin interactions, such as Hi-C, have been used to assemble chromosomes, which can produce ultra-long scaffolds; however, a certain amount of error occurs when used for *de novo* assembly[15, 16]. Thus, accurate chromosome-level assembly remains a major challenge.

The most widely used strategy for chromosome-level assembly of the scaffolds generated by the second-generation sequencing is based on a high-density genetic map at chromosome level. Nevertheless, this strategy is feasible only when high-density genetic maps of a species are available. Because there is no genetic map available for *Monopterus,* we have developed an efficient assembly strategy: *de novo* chromosome assembly by FISH walking assisted by conserved synteny (Cafs). Using Cafs technology, which is efficient and cost effective, a precise chromosome-level assembly covering 81.3% of the sequences assembled in scaffolds was produced.

Whole genome sequencing

A whole-genome shotgun strategy and next-generation sequencing technology (Illumina HiSeq 2000 platform) were used to sequence two male *Monopterus*. Genomic DNA was extracted

from eels from the Wuhan area in the Yangtze River basin. To reduce the risk of non-random sequencing, 8 paired-end sequencing libraries with insert sizes of 170 bp, 500 bp, 800 bp, 2 kb, 5 kb, 10 kb, 20 kb, and 40 kb were constructed for the genome. The libraries generated 101.62 GB of sequence data. To reduce sequencing errors in the assembly, sequence reads were filtered to remove low quality reads. After filtering, 78.64 GB (97.6X) of sequence data were retained for the assembly, which ensures a high single-base accuracy (Additional file: Figure S1 and Table S1).

Estimation of genome size

A k-mer was defined as a sequence of k bases in length. The frequency of k-mers in a collection of short, insert-sized reads could be calculated with a 1 bp sliding window. When an optimal amount of data was present, the k-mer frequency followed a Poisson distribution. The k-mer value was used to estimate the genome size, as follows: Genome Size = K_num/Peak_depth, where K_num is the total number of k-mers, and Peak_depth is the expected value of the k-mer depth [17]. The 17-mer distribution obeyed the theoretical Poisson distribution. Finally, we observed that the proportion of heterozygosity in the *Monopterus* genome was small, and estimated that the entire genome comprised 806 Mb, with a GC content of 40.8% (Additional file: Figures S2-S3 and Table S2).

De novo genome assembly

The *Monopterus* genome was *de novo* assembled with the SOAPdenovo software [17] (http://soap.genomics.org.cn). SOAPdenovo employs the de Bruijn graph algorithm to simplify assembly and reduce the computational complexity. Low quality reads were filtered out and potential sequencing errors were removed or corrected with the k-mer frequency methodology. The SOAPdenovo assembly process consisted of three main steps: contig construction, scaffold construction, and gap filling. The sequencing data derived from 2 kb, 5 kb, 10 kb and 20 kb insert size library was used to assemble the scaffolds by SOAPdenovo. The sequencing data derived from 40 kb insert size library were used to build scaffolds with SSPACE (version 1.1) software.

To assess assembly quality and completeness, high quality reads from short-insert-size libraries (75 bp read lengths) were aligned to the assembly with the BWA program [18] (version 0.5.9-r16), with default parameters. Next, SOAPcoverage (version 2.27) was used to calculate sequencing depth. A total of 91.06 % reads could be mapped, and they covered 99.69 % of the assembly, excluding gaps. To further test for possible contigs that might be mis-joined in scaffolds, we analyzed paired-end information. We found that, if contigs were included only when both ends could be uniquely mapped onto the assembly, more than 90.65 % of paired-ends were in the correct orientation and at the expected distance, according to the utilized short-insert-size libraries.

The final assembly comprised 689.5 Mb with contig and scaffold N50 sizes of 22.2 kb and 2.1 Mb, respectively (Table 1). Over 90% of the total sequence was covered by 379 scaffolds; the longest scaffold spanned 11.7 Mb (Table 1). Assembly accuracy was further demonstrated by 99.7% reads mapping to the reference sequence of the genome and the successful mapping of 321 bacterial artificial chromosomes (BACs), sequenced with Sanger sequencing technology (Additional file: Tables S3).

To evaluate the quality of the assembled genome, we conducted BUSCO (Benchmarking Universal Single-Copy Orthologs) analysis [19] using BUSCO v2.0 with vertebrata_odb9 including 2,586 BUSCOs. Using the BUSCO analysis, 96.5% of BUSCOs were completely detected in the assembled genome (2,464: complete and single-copy, 32: complete and duplicated) among 2,586 tested BUSCOs. The number of fragmented and missing BUSCOs was 56 and 34, respectively. Together, the genome of the *Monopterus* assembled is of high quality.

Repeat elements

Transposable elements (TEs) were identified in the genome with combination of homology-based and de novo approaches. The homology-based approach utilized database Repbase [20] (release 19.06), with RepeatMasker (version 4.0.3) and RepeatProteinMask (from the

RepeatMasker package) programs with the default parameters [20]. The de novo approach used two prediction programs, RepeatModeler [21] (version 1.0.7) and LTR-FINDER [22] (version 1.0.5), to build the de novo repeat libraries based on the genome sequences. Next, contaminations and multi-copy genes were removed from the libraries. Then, the RepeatMasker was used a second time to find repeats in these repetitive sequence libraries. Finally, we combined all the results generated by these methods. To improve our comparisons to other teleost fishes, we employed the same procedure and parameters to analyze the *Danio rerio*, *Oryzias latipes*, *Gasterosteus aculeatus*, *Tetraodon nigroviridis*, and *Takifugu rubripes*.

The repetitive element content (Additional file: Figure S4) of the *Monopterus* genome (28%) was much lower than that of the zebrafish (61%) and about the same as medaka (29%) genomes, but higher than that of the threespine stickleback (16%) and pufferfish (8-10%) genomes. In the *Monopterus* genome, the main repetitive transposable elements were the DNA transposons and LINEs. At 8%, the LINEs were the largest category of transposable elements. The percent of LINEs was greater than that found in other teleost fish (2-5%), which were involved in guardians for genome defense and germline stability via Piwi-interacting small RNAs (piRNAs) [23].

Genes and function annotation

We used both homology-based and de novo methods to predict genes in the *Monopterus* genome by scanning the local *Monopterus* genome database, which also included RNA-seq data. For the homology-based prediction, protein sequences from *D. rerio*, *O. latipes*, *G. aculeatus*, *T. nigroviridis*, and *T. rubripes* were downloaded from the Ensembl platform[24] (release 75) and aligned with the *Monopterus* genome with the Tblastn program[25]. Accordingly, homologous genomic sequences were input into the Genewise program[26] to align matching proteins. This procedure allowed us to define gene structures. For de novo prediction, both the Fgenesh[27] and Genscan[28] programs were employed to predict coding genes, with the appropriate parameters. Homology-based and de novo derived gene sets were combined with comprehensive, non-redundant reference gene sets, obtained with the GLEAN platform (http://sourceforge.net/projects/glean-gene/). Genes were corrected by comparisons

with the RNA-seq data; these RNA-seqs were mapped to the *Monopterus* genome with the Tophat program, and the Cufflinks program (http://cufflinks.cbcb.umd.edu/) was used to assemble transcripts. After that, we selected 1000 intact genes, defined as gene set "A", which were supported by the homology-based prediction, and passing a fifth-order Markov model, to verify the ORFs of RNA transcripts based on the Hidden Markov Model (HMM). In the *Monopterus*, a total of 24,056 protein-coding genes were predicted (Additional file: Table S4-S6). The gene sizes were similar to those of other teleost fish (Additional file: Figure S5 and Table S5).

Blastp was used to search for proteins encoded in the *Monopterus* genome by comparing candidate sequences against the SwissProt and TrEMBL databases from UniProt Knowledgebase (UniProtKB) [29]. The annotated motifs and domains in the available databases (ProDom, PRINTS, Pfam, SMART, PANTHER, and PROSITE) were obtained with the InterProScan program [30] (version 4.7). In gene ontology (GO) [31] analyses, gene functions were obtained from the corresponding InterPro entries. Subsets of the GO terms were obtained according to the program of DAVID program (version 6.7) [32]. X-associated genes were annotated based on human GO term list and Z-associated genes were annotated based on chicken GO term list. All genes were also aligned against the KEGG [33] (release 68) protein database. The genes that matched genes in the KEGG database were assumed to be involved in the corresponding signaling pathways. Approximately 80% of the genes could be functionally annotated with homology analysis (Additional file: Figure S6).

***De novo* chromosome assembly by Cafs-strategy**

To assemble chromosomes with accurate sequences from the scaffolds, we developed an efficient assembly strategy without using any genetic map information, Cafs (Figure 1), which is based on chromosome fluorescent *in situ* hybridization (FISH) and the shared synteny between closely related fish species.

We first prepared probes of BACs from sequenced clones and PCR fragment pools (8-15

sequences and covers a total length of 20-30 kb on a scaffold) representing scaffolds for chromosome FISH, and performed synteny analysis of these scaffolds by comparing with the fish species (medaka, sticklebacks, *Tetraodon* and *Monopterus*). Second, from the synteny information of the homologous sequences of these scaffolds in the three fish species，probe combination mapping was used to determine 12 linkage groups, corresponding to 12 chromosomes, each with a molecular landmark (Figure 1A). Briefly, group A and B were first discriminated by two unlinked scaffolds labelled with two different colours. If another scaffold was unlinked to the previous two scaffolds, the third scaffold was identified as a marker of group C. Third, based on the predicted syntenic relationship between closely related fish species, probes for the candidate scaffolds were co-hybridized with the landmarks of the chromosomes, which have been identified. Scaffolds with no predicted location and that were inconsistent with the predicted location were further determined by co-hybridization with 12 landmarks using dual-colour FISH respectively (Figure 1B). For example, scaffold 58 would be grouped into the F group, as it is linked with scaffold 129, which was the landmark of the F group. Fourth, the loose and long pachytene chromosomes were adopted to determine the location and order relationship of the scaffolds through dual- and three-colour FISH. An original marker was used as a walking start (e.g., scaffold 73), and the location of the second scaffold (e.g., scaffold 186) relative to the original marker was identified by dual-colour FISH. The location of a new scaffold was determined by the known scaffold locations using dual- or three-colour FISH (e.g., scaffolds 72, 123, and 4) (Figure 1C). Finally, because all 12 chromosomes are telocentric, the telomeres of the metaphase chromosome were used as landmarks to determine the directions of the mapped scaffolds on the chromosomes (Figure 1D). The relative position of all scaffolds on chromosomes was determined by the measurement of the signals to the centromere (Figure 1E). The distance values were measured by Image-Pro Plus 6.0, and each value was obtained from an average of more than five cells.

Using the Cafs assembly strategy, we conducted large-scale mapping of the scaffolds on each chromosome. Metaphase chromosomes were prepared according to routine protocols from the *Monopterus* kidney tissue [5]. Meiotic pachytene bivalents were prepared from *Monopterus* testis using a previously described method [34]. The FISH was conducted as previously

described [35]. The BAC end sequences were aligned to the genome database by Blat. BACs with two ends aligned to one scaffold, and those ends with sequences with homology to scaffolds greater than 90% were used as probes for FISH. Of the ~747 sequenced clones, 148 BACs could be used as probes for FISH (Table S7). The BACs were confirmed by PCR sequencing from the internal regions of the BACs. 148 BACs and 38 PCR fragment pools (8-15 sequences and covers a total length of 20-30 kb on a scaffold) representing 186 scaffolds (Table S8) were prepared as probes for chromosome FISH.

Before the hybridization experiment, we performed a genome-wide synteny analysis to compare these fish species. We constructed a reference map using the syntenic relationship among the genomes of medaka, stickleback and *Tetraodon* to help map the scaffolds on the *Monopterus* chromosomes. The syntenic blocks between *Monopterus* and other fishes were aligned by Lastz (Blastz) [36] with parameters of T=2 and Y=3400. Furthermore, we used Blat to search for homologous sequences among medaka, sticklebacks, *Tetraodon* and *Monopterus* in order to fill the gap sequences of blocks in the reference map. If two homologous sequences were linked in all three close species, we defined the corresponding scaffolds in *Monopterus* as predicted linked scaffolds.

Under the guidance of the synteny of the homologous sequence of these 186 scaffolds in other three fish species，78 probes combinations of co-hybridization were performed to identify 12 linkage groups, each with a molecular landmark (Figure 2A). We then conducted the walking in a range of 11-22 steps per chromosome (Figure 2B; Additional file: Figure S7). A total of 186 scaffolds were assembled into 12 pachytene chromosomes through step-by-step combination hybridization of the probes using the above-mentioned 148 BACs and 38 PCR fragment pools (Figure 3A). We then determined the orientation of each chromosome by dual-colour FISH on metaphase chromosomes using the telomere as a morphological landmark (Additional file: Figure S8). Of these mapped scaffolds, 92% (99/108) were consistent with the shared synteny between closely related fish species (medaka, sticklebacks and *Tetraodon*). From the synteny analysis, 142 scaffolds were further assembled into 12 chromosomes respectively. Approximately 8% (~11) of the scaffolds could not be resolved by the conserved

synteny prediction due to possible rearrangements in the *Monopterus* lineage. This issue should be further corrected in future experiments.

We finally integrated 328 scaffolds into the reference genome. These mapped scaffolds consisted of 455 Mb determined by FISH and 100 Mb determined by syntenic analysis, which covers genomic sequences of a total length of 555 Mb, accounting for 81.3% of the sequences assembled in scaffolds (689.5 Mb). Based on the assembly, a total of 18,660 protein-coding genes were annotated with location information on the chromosomes (Table 2). For example, there are 87 protein-coding genes on scaffold 72, which was located on chromosome 5 (Figure 3B). These data indicate that a *de novo* chromosome-level assembly of the *Monopterus* genome was produced using the Cafs strategy.

Chromosome-wide gene clustering

To further investigate gene clustering along the chromosomes, we calculated the gene density per chromosome. The average gene density in the genome was 33.6 genes per Mb, with the maximum gene density on chromosome 12, which is the shortest chromosome, and the minimum gene density on chromosome 9 (Table 2). Further sliding window analysis showed that there was also biased distribution of the gene density within the chromosome (Figure 4A). Using a 1-Mb window size and 100-kb step size, the maximum gene density in the genome was detected from nt 22,200,001 to nt 23,200,000 on chromosome 10, which contains 71 genes, in comparison with an average of 33.6 genes per Mb in the genome (Figure 4B).

We then tested the statistical significance of pairing correlations among the gene density, GC content. As these parameters are not normally distributed, we used the non-parametric Spearman correlation test on the ranks of the paired quantities. Correlation analyses were performed with R software (www.r-project.org). The R package ggplot2 was used to draw scatterplots and boxplots. The distribution pattern of the gene density was consistent with the corresponding GC content along the chromosomes (Figure 4A; Additional file: Figure S9). To investigate whether the distribution of the genes along the chromosomes is non-random, we computed the probability of ≥ actual numbers of ridges under a random permutation of the gene

positions following previous method [37]. A ridge was used to describe a chromosome region with high gene density, which is thus defined as at least W consecutive windows, each containing a gene number higher than H. Thus, the ridge is determined by two parameters: cutoff 1 (C_H), gene number per window, and cutoff 2 (C_W), number of consecutive windows. The actual ridge numbers (N) in the genome were calculated under C_H and C_W by sliding window analysis. We used the following calculation parameters to set up a null model: suppose we have a random permutation of X_1, X_2,..., X_i in the range of 1 to S; i, gene number on the chromosome; S, length of the chromosome; and X_1, X_2,..., X_i, gene locations on the chromosome. With the same cutoff values under actual conditions (C_H and C_W), we can obtain a ridge number (n) under the null model. We can compute the frequency (f) when $n \geq N$ by permutation 10,000 times. If f = 0, the p-value < 10^{-4}, or the p-value = f/10000. For all of the cutoff C_H and C_W combinations, we calculated the p-value under different window sizes of 0.2, 0.3, 0.5, 1, 2, and 3 Mb respectively.

Using a combination of the two cutoffs, the numbers of the ridges of each chromosome can be identified. For example, using cutoffs of 40 genes per Mb and 5 consecutive windows, 7 ridges on chromosome 10 were identified (Figure 4B), and 90 ridges were identified in the genome (Figure 4C). The probability of the observed ridges occurring in random permutations of gene positions was very low (p-value < 10^{-4}) (Figure 4C), confirming non-random and clustering distribution of genes along the chromosomes. Probabilities (ridges numbers under a random permutation ≥ ridges numbers in the *Monopterus* genome) for a series of cutoff sets and different window sizes were also calculated and the results showed that there were significant differences in ridges numbers between the *Monopterus* genome and random permutations of gene positions (Figure 4C; Additional file: Figure S10). The ridge numbers of high gene density directly reflect the clustering of genes along the chromosomes. These analyses suggest that the ridge pattern on the chromosomes probably represents a higher-order structure in the genome.

Data availability statement

The genome data from this study have been deposited at DDBJ/EMBL/GenBank under accession number AONE00000000, and the raw transcriptome data have been submitted to

NCBI Gene Expression Omnibus (GEO; http://www.ncbi.nlm.nih.gov/geo/) under accession number GSE43649.

Additional files

Supplemental Figure S1. Sequencing depth distribution of the *Monopterus* genome.

Supplemental Figure S2. Genome size estimation using 17-mer.

Supplemental Figure S3. The GC distribution of the *Monopterus* genome.

Supplemental Figure S4. Divergence distribution of the classified TE elements.

Supplemental Figure S5. Comparisons of predicted coding genes of *Monopterus* with other teleost fishes.

Supplemental Figure S6. The Gene Ontology of the *Monopterus* genes.

Supplemental Figure S7. Localization of each scaffold on chromosomes by FISH-walking strategy.

Supplemental Figure S8. Orientation of each linkage group on metaphase chromosomes.

Supplemental Figure S9. Correlation analysis of gene density with GC content.

Supplemental Figure S10. Statistical tests of numbers of gene density ridges in the genome corresponding to background noise (null model) in different window sizes (0.2, 0.3, 0.5, 1, 2, 3 Mb).

Supplemental Table S1. Statistics of sequencing.

Supplemental Table S2. Statistics of genome from 17-mer.

Supplemental Table S3. Statistics of mapping.

Supplemental Table S4. Statistics of predicted coding genes.

Supplemental Table S5. Comparisons of predicted coding genes of *Monopterus* with other teleost fishes.

Supplemental Table S6. Annotated classification of the *Monopterus* genes.

Supplemental Table S7. Alignments of BAC ends to reference genome.

Supplemental Table S8. Information of FISH probes synthesized by PCR.

List of Abbreviations

BAC: bacterial artificial chromosome; bp: base pair; BUSCO: Benchmarking Universal Single-

Copy Orthologs; Cafs: chromosome assembly by FISH walking assisted by conserved synteny; DAPI :49-6-diamidino-2-phenylindole; FISH: fluorescent *in situ* hybridization; Gb: giga base; GO: gene ontology; H&E: haematoxylin and eosin; Hi-C: high-throughput/resolution chromosome conformation capture; kb: kilo base; KEGG: Kyoto Encyclopedia of Genes and Genomes; LINEs: long interspersed nuclear elements; LRseq: long-read sequencing; Mb: mega base; PAC: plant artificial chromosome; piRNAs: Piwi-interacting small RNAs; SMRT : single-molecule real-time; TEs: Transposable elements.

Acknowledgements

This work was supported by the National Natural Science Foundation of China, National Key Technologies R&D Program and Hubei Province Science and Technology Project.

Author contributions

Conceptualization: Rongjia Zhou.

Funding acquisition: Rongjia Zhou, Hanhua Cheng.

Investigation: Xueya Zhao, Majing Luo, Zhigang Li, Yibin Cheng, Fengling Lai, Xin Wang, Jiumeng Min, Mingzhou Bai, Yulan Yang.

Methodology: Xueya Zhao, Majing Luo, Zhigang Li, Pei Zhong.

Supervision: Rongjia Zhou, Hanhua Cheng.

Validation: Xueya Zhao, Rongjia Zhou.

Writing – original draft: Xueya Zhao, Rongjia Zhou.

Writing – review & editing: Xueya Zhao, Rongjia Zhou.

Ethics statement

Monopterus were obtained from Hubei, China. All animal experiments and methods were performed in accordance with the relevant approved guidelines and regulations, as well as under the approval of the Ethics Committee of Wuhan University.

Competing interests

The authors declare that they have no competing interests

Figure legends

Figure 1 Overview of *de novo* chromosome assembly by FISH walking assisted by conserved synteny. A: Identification of 12 linkage groups by probe combination mapping. FISH probes are hybridized on pachytene chromosomes. Red and green dots indicate scaffold locations. B: Synteny-assisted scaffold mapping. Each candidate BAC (scaffold) is co-hybridized with 12 landmarks by dual-colour FISH respectively. Synteny-supported/non-supported scaffolds are determined by FISH. C: Determination of scaffold order on chromosome by FISH walking. (I) The order of two scaffolds is identified by dual-colour FISH if both of them are on one side of the chromosome. (II) If the scaffolds are in the centre of the chromosome, three-colour FISH is applied to determine their order. (III) The order of some scaffolds (labelled with one colour) could be identified by three signals dual-colour FISH, when their two neighbouring scaffolds (labelled with another colour) have been determined. D: Identification of orientation of linkage groups on metaphase chromosomes. Telomeres and centromeres can be observed on the metaphase chromosomes. E: Localization of scaffolds is determined by calculating the corresponding distances to the centromere.

Figure 2 Chromosome assembly by Cafs. A: FISH images show 12 molecular landmarks corresponding to 12 chromosomes. Green signals indicate the landmarks labelled by digoxigenin and detected with FITC. Each chromosome is determined by a landmark. Chromosomes are stained by DAPI (blue). B: Localization of each scaffold on chromosome 5 by FISH walking strategy. FISH images and corresponding scaffold order from (a') to (m') are shown in the left panels. A three-colour FISH image (g') in the upper right indicates the relative order of scaffolds 4 (yellow, FITC+Cy3), 30 (green, FITC) and 99 (red, Cy3) on chromosome 5. Probes (red dots) and their locations on scaffolds are used to assemble chromosome 5.

Figure 3 Chromosome-scale assembly of the *Monopterus* genome. A: Each chromosome is assembled with scaffolds and their order from telomere (down end) to centromere (up end). The grey and purple cylinders represent the anchored scaffolds. The segments in light blue

between two neighbouring scaffolds indicate gaps. Sticks with a red head anchored on each scaffold indicate the positions of the BACs used as probes. Scale bar, 0-1. B: Scaffold 72 with 87 genes (blue bars) and their location on chromosome 5 is highlighted.

Figure 4 Chromosome-wide gene clustering. A: Circos is used to plot the assembled chromosomes, GC content and gene density. The inner scale is 2 Mb. The strips in the outer circle indicate the scaffolds anchored by FISH. The strips in the second outer circle indicate the scaffolds packaged into chromosomes filled the gaps with 50 kb of Ns. The inner dark grey ridges show the moving GC percentage, and the inner grey ridges show the moving number of the genes at a window size of 1 Mb. B: Distribution of gene clusters (ridges) on chromosome 10. Curves indicate the moving numbers of genes at a window size of 1 Mb (step = 100 kb). The windows with a maximum gene density from nt 22200001 to 23200000 on chromosome 10, which contains 71 genes, are shown in the lower panel. Green boxes highlight ridges in which there are at least 5 consecutive moving windows with a lower limit of 40 genes per window. C: Statistical tests of numbers of gene density ridges in the genome corresponding to background noise (null model). The heat map in the lower panel shows *p*-values in the significance test of observed ridge numbers against the null model (10,000 independent permutations of gene positions). The *x*-axis indicates the cutoff values of numbers of consecutive moving windows, which reflects the extent of the clustering. The *y*-axis indicates the cutoff values of gene numbers within a certain window size (step 100 kb), which reflects the degree of intensity of the clusters. Green lines represent the average gene number in a certain window size. The upper panel highlights a significance test at the condition of two cutoff values, gene density (40/Mb) and consecutive window numbers (5). Red dots represent the number of observed ridges in the genome. Boxplots (black) represent distribution of the ridge numbers in 10,000 independent permutations of gene positions in a random fashion.

References

1. Collins TM, Trexler JC, Nico LG, Rawlings TA. Genetic Diversity in a Morphologically Conservative Invasive Taxon: Multiple Introductions of Swamp Eels to the Southeastern United States. Conserv Biol. 2002; 16:1024-35.
2. Cheng HH, Guo YQ, Yu QX, Zhou RJ. The rice field eel as a model system for vertebrate sexual

development. Cytogenet Genome Res. 2003; 101:274-7.

3. Liu CK. Rudimentary hermaphroditism in the symbranchoid eel, Monopterus javanensis. Sinensia. 1944; 15:1-8.

4. Bullough WS. Hermaphroditism in the lower vertebrates. Nature. 1947; 160:9-11.

5. Yu XJ, Zhou T, Li YC, Li K, Zhou M. Chromosomes of Chinese fresh-water fishes. Beijing Science Press. 1989:1-148.

6. Zhou RJ, Cheng HH, Tiersch TR. Differential genome duplication and fish diversity. Rev Fish Biol Fisher. 2002; 11:331-7.

7. Christoffels A, Koh EG, Chia JM, Brenner S, Aparicio S, Venkatesh B. Fugu genome analysis provides evidence for a whole-genome duplication early during the evolution of ray-finned fishes. Mol Biol Evol. 2004; 21:1146-51.

8. Jaillon O, Aury JM, Brunet F, Petit JL, Stange-Thomann N, Mauceli E, et al. Genome duplication in the teleost fish Tetraodon nigroviridis reveals the early vertebrate proto-karyotype. Nature. 2004; 431:946-57.

9. Huddleston J, Ranade S, Malig M, Antonacci F, Chaisson M, Hon L, et al. Reconstructing complex regions of genomes using long-read sequencing technology. Genome Res. 2014; 24:688-96.

10. Goodwin S, Gurtowski J, Ethe-Sayers S, Deshpande P, Schatz MC, McCombie WR. Oxford Nanopore sequencing, hybrid error correction, and de novo assembly of a eukaryotic genome. Genome Res. 2015; 25:1750-6.

11. Chaisson MJ, Huddleston J, Dennis MY, Sudmant PH, Malig M, Hormozdiari F, et al. Resolving the complexity of the human genome using single-molecule sequencing. Nature. 2015; 517:608-11.

12. Voskoboynik A, Neff NF, Sahoo D, Newman AM, Pushkarev D, Koh W, et al. The genome sequence of the colonial chordate, Botryllus schlosseri. Elife. 2013; 2:e00569.

13. Adey A, Kitzman JO, Burton JN, Daza R, Kumar A, Christiansen L, et al. In vitro, long-range sequence information for de novo genome assembly via transposase contiguity. Genome Res. 2014; 24:2041-9.

14. Kim J, Larkin DM, Cai Q, Asan, Zhang Y, Ge RL, et al. Reference-assisted chromosome assembly. Proc Natl Acad Sci U S A. 2013; 110:1785-90.

15. Putnam NH, O'Connell BL, Stites JC, Rice BJ, Blanchette M, Calef R, et al. Chromosome-scale shotgun assembly using an in vitro method for long-range linkage. Genome Res. 2016; 26:345-50.

16. Burton JN, Adey A, Patwardhan RP, Qiu R, Kitzman JO, Shendure J. Chromosome-scale scaffolding of de novo genome assemblies based on chromatin interactions. Nat Biotechnol. 2013; 31:1119-25.

17. Li RQ, Fan W, Tian G, Zhu HM, He L, Cai J, et al. The sequence and de novo assembly of the giant panda genome. Nature. 2010; 463:311-7.

18. Li H, Durbin R. Fast and accurate short read alignment with Burrows-Wheeler transform. Bioinformatics. 2009; 25:1754-60.

19. Simao FA, Waterhouse RM, Ioannidis P, Kriventseva EV, Zdobnov EM. BUSCO: assessing genome assembly and annotation completeness with single-copy orthologs. Bioinformatics. 2015; 31:3210-2.

20. Jurka J, Kapitonov VV, Pavlicek A, Klonowski P, Kohany O, Walichiewicz J. Repbase Update,

a database of eukaryotic repetitive elements. Cytogenet Genome Res. 2005; 110:462-7.

21. Price AL, Jones NC, Pevzner PA. De novo identification of repeat families in large genomes. Bioinformatics. 2005; 21:i351-i8.

22. Xu Z, Wang H. LTR_FINDER: an efficient tool for the prediction of full-length LTR retrotransposons. Nucleic Acids Res. 2007; 35:W265-8.

23. Reuter M, Berninger P, Chuma S, Shah H, Hosokawa M, Funaya C, et al. Miwi catalysis is required for piRNA amplification-independent LINE1 transposon silencing. Nature. 2011; 480:264-7.

24. Flicek P, Ahmed I, Amode MR, Barrell D, Beal K, Brent S, et al. Ensembl 2013. Nucleic Acids Res. 2013; 41:D48-55.

25. Mount DW. Using the Basic Local Alignment Search Tool (BLAST). CSH Protoc. 2007; 2007:pdb top17.

26. Birney E, Clamp M, Durbin R. GeneWise and genomewise. Genome Res. 2004; 14:988-95.

27. Burge C, Karlin S. Prediction of complete gene structures in human genomic DNA. J Mol Biol. 1997; 268:78-94.

28. Salamov AA, Solovyev VV. Ab initio gene finding in Drosophila genomic DNA. Genome Res. 2000; 10:516-22.

29. Bairoch A, Apweiler R. The SWISS-PROT protein sequence database and its supplement TrEMBL in 2000. Nucleic Acids Res. 2000; 28:45-8.

30. Mulder N, Apweiler R. InterPro and InterProScan: tools for protein sequence classification and comparison. Methods Mol Biol. 2007; 396:59-70.

31. Ashburner M, Ball CA, Blake JA, Botstein D, Butler H, Cherry JM, et al. Gene Ontology: tool for the unification of biology. Nat Genet. 2000; 25:25-9.

32. Huang da W, Sherman BT, Lempicki RA. Systematic and integrative analysis of large gene lists using DAVID bioinformatics resources. Nat Protoc. 2009; 4:44-57.

33. Kanehisa M, Goto S. KEGG: kyoto encyclopedia of genes and genomes. Nucleic Acids Res. 2000; 28:27-30.

34. Yu QX, Fan LC, Cui JX, Ren XH, Li K, Yu XJ. High resolution G-binding and idiogram on pachytene bivalents of rice field eels. Sci China (B). 1994:1090.

35. Henegariu O, Dunai J, Chen XN, Korenberg JR, Ward DC, Greally JM. A triple color FISH technique for mouse chromosome identification. Mamm Genome. 2001; 12:462-5.

36. Schwartz S, Kent WJ, Smit A, Zhang Z, Baertsch R, Hardison RC, et al. Human-mouse alignments with BLASTZ. Genome Res. 2003; 13:103-7.

37. Caron H, van Schaik B, van der Mee M, Baas F, Riggins G, van Sluis P, et al. The human transcriptome map: clustering of highly expressed genes in chromosomal domains. Science. 2001; 291:1289-92.

Table 1. Statistics of the assembly of the *Monopterus* genome.

	Contigs*		Scaffolds	
	**Size (bp)**	**Number**	**Size (bp)**	**Number**
N90	4,762	33,115	368,242	379
N80	8,655	23,414	775,515	254
N70	12,290	17,275	1,109,624	180
N60	16,188	12,785	1,519,751	128
N50	22,239	8,438	2,106,322	87
Longest	159,913	----	11,676,616	----
Total size	634,655,961	----	689,524,511	----
Total number(>=100bp)	----	117,579	----	62,978
Total number (>=2kb)	----	44,314	----	2,360

*The contig size was the final size after filling intra-scaffold gaps. Contigs with lengths shorter than 100bp were not included in the statistics.

 Click here to download Table Table 2.docx

Table 2. Assembly statistics for each chromosome

Chromosome	Chromosome size (Kb)	Scaffolds No.	Gene No.	Gene density (n/10Mb)
**1**	75908.7	33	2264	298
**2**	65103.9	32	2133	328
**3**	51637.3	21	1872	363
**4**	51162.1	30	1791	350
**5**	50080.0	27	1517	303
**6**	48093.1	27	1659	345
**7**	42410.1	29	1500	354
**8**	41999.7	30	1456	347
**9**	41928.7	23	1241	296
**10**	34690.8	30	1262	364
**11**	29285.5	23	1086	371
**12**	22774.4	23	879	386
**Total**	555074.3	328	18660	336

Figure 1

Click here to download Figure Figure 1.tif

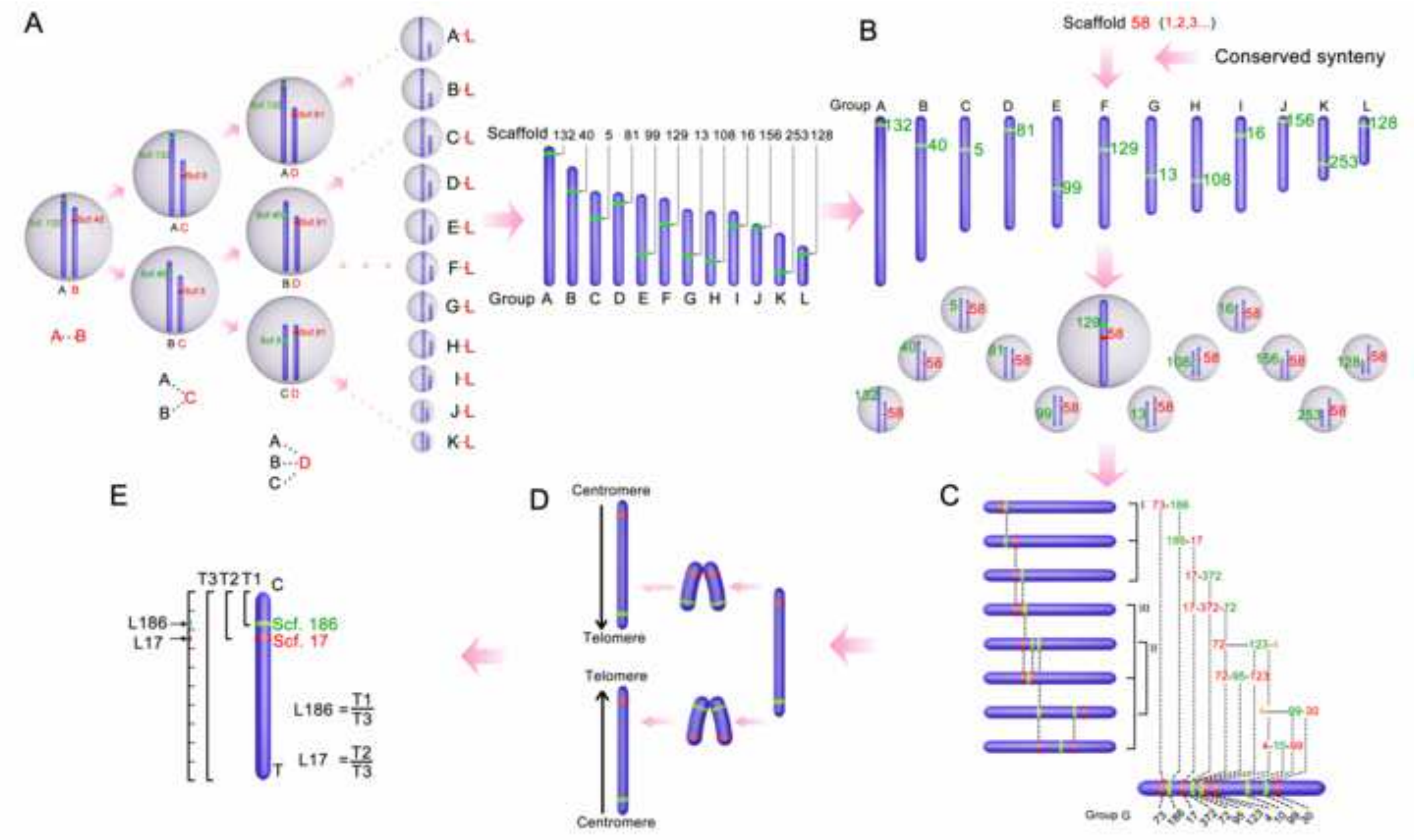

Figure 2

Click here to download Figure Figure 2 .tif

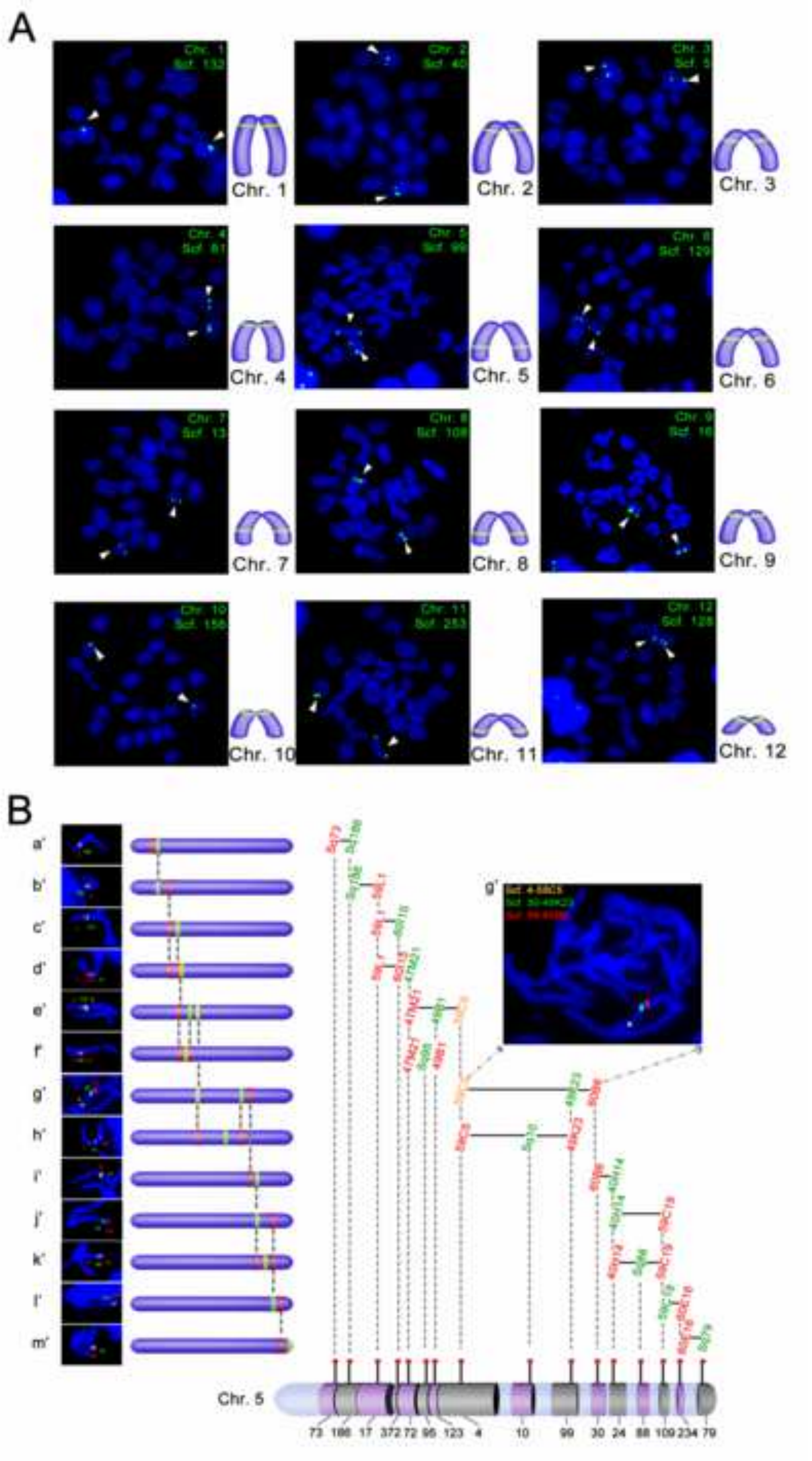

Figure 3

Click here to download Figure Figure 3.tif

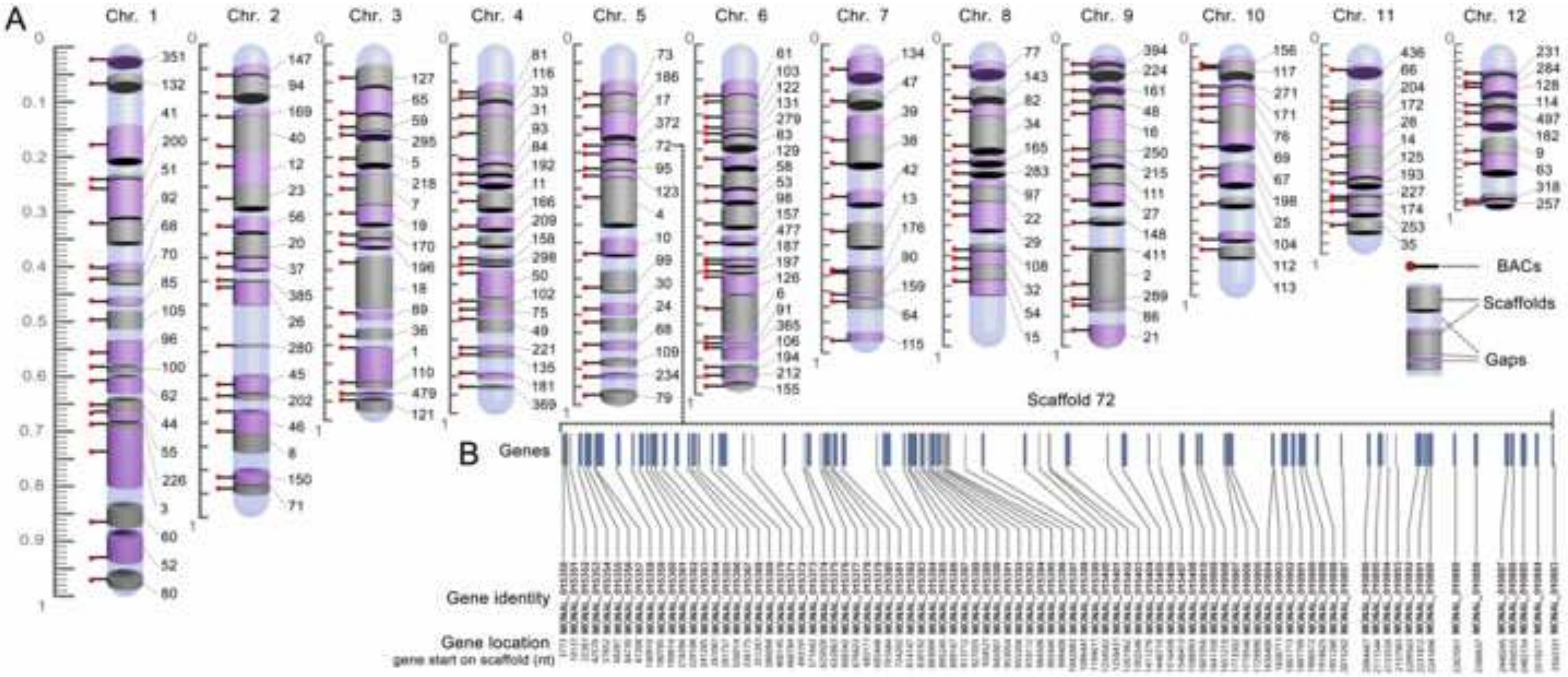

Figure 4

Click here to download Figure Figure 4.tif

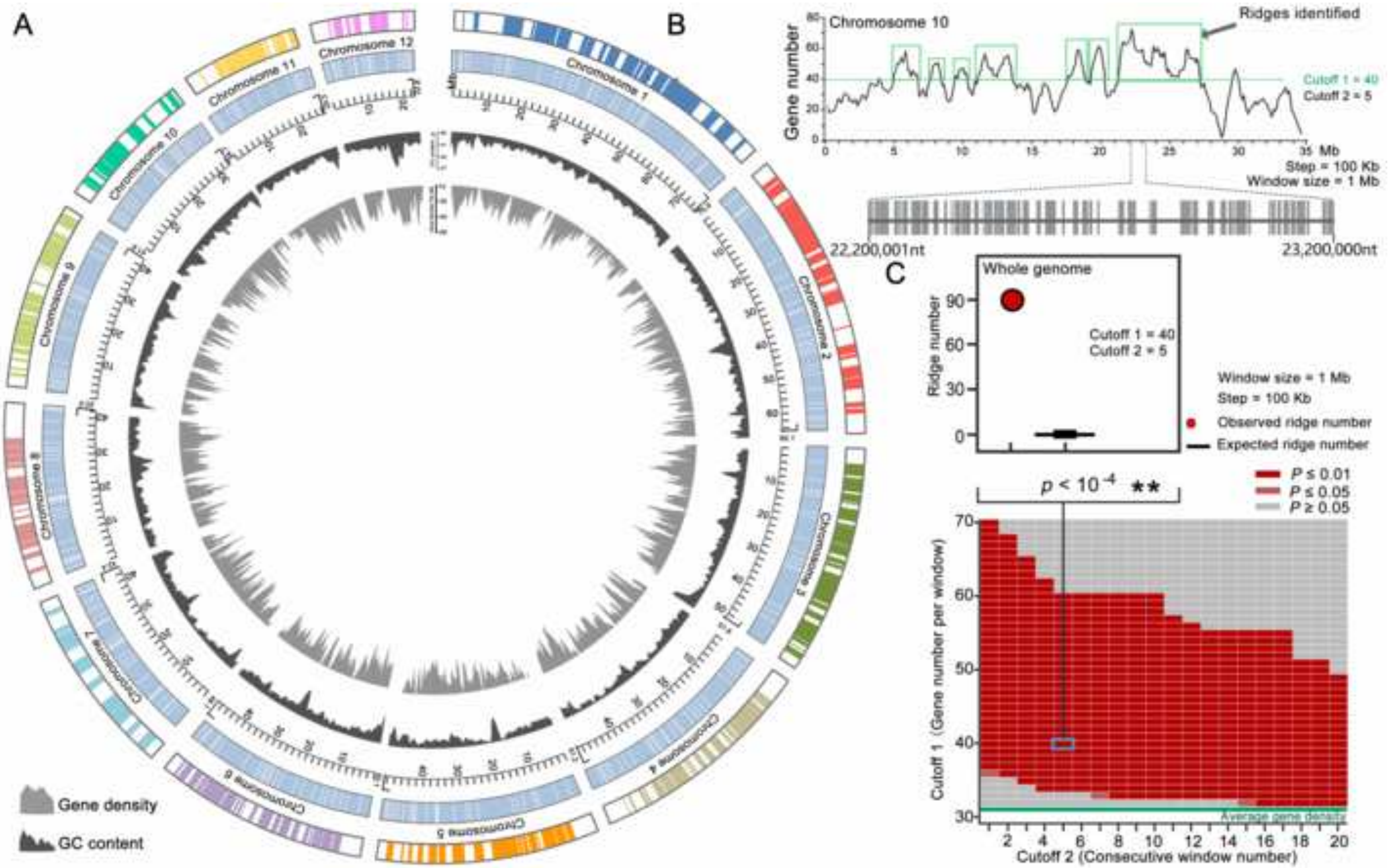

Click here to access/download
Supplementary Material
Additional files-datanote.docx

Personal Cover Click here to download Personal Cover cover letter.docx

Wuhan University

Laboratory of Molecular and Developmental Genetics, College of Life

Since 1893

Wuhan 430072, China
Tel: (0086)27-68756253, E-mail: rjzhou@whu.edu.cn

Editors
GigaScience

January 16, 2018

Dear Editor Hans,

Many thanks for your handling of my manuscript GIGA-D-17-00210 entitled "**Chromosome-scale assembly of the *Monopterus* genome reveals a co-regulation landscape of interconvertible regions of sex during sex transition**" for your consideration for publication in *GigaScience*.

Following your suggests, we have revised and formatted the manuscript as a "Data Note", deleted the second part and focus on description of the genome assembly. The revised manuscript title is "**Chromosome-scale assembly of the Monopterus genome".**

We have revised the manuscript following comments of two reviewers seriously, and provided a file to list **Point-to-point responses to reviewers.**

After revised, we have submitted it in your online system. Would you please check, and let me know if any problem.

The manuscript has not been submitted or is under consideration for publication elsewhere now. All authors agree to submit to your journal.

I look forward to hearing from you. Thank you for your consideration!

With best regards,

Rongjia Zhou, Ph.D.
Luojia Distinguished Professor
Genetics
