# Supplementary material for: Chromosome-scale assembly of the Monopterus genome
Source: Gigascience. 2018 Apr 24;7(5):giy046. doi: 10.1093/gigascience/giy046 (PMC5946948; doi:10.1093/gigascience/giy046)
Supplement: GIGA-D-17-00210_Revision_2.pdf [file giy046_giga-d-17-00210_revision_2.pdf]

GigaScience

Chromosome-scale assembly of the Monopterus genome

--Manuscript Draft--

**Manuscript Number:**	GIGA-D-17-00210R2
**Full Title:**	Chromosome-scale assembly of the Monopterus genome
**Article Type:**	Data Note
**Funding Information:**	National Natural Science Foundation of China (31571280) — Professor Rongjia Zhou<br>National Natural Science Foundation of China (31471182) — Prof. Hanhua Cheng
**Abstract:**	Background: The teleost fish Monopterus albus is emerging as a new model for biological studies due to its natural sex transition and small genome, in addition to its enormous economic and potential medical value. However, no genomic information for the Monopterus is currently available.<br><br>Findings: Here, we sequenced and de novo assembled whole genome of the Monopterus, and report the de novo chromosome assembly by FISH walking assisted by conserved synteny (Cafs) for Monopterus. Using Cafs, 328 scaffolds were assembled into 12 chromosomes, which cover genomic sequences of 555 Mb, accounting for 81.3% of the sequences assembled in scaffolds (~689 Mb). A total of 18,860 genes were mapped on the chromosomes and showed a non-random distribution along chromosomes.<br><br>Conclusions: We report the first reference genome of the Monopterus and provided an efficient Cafs strategy for a de novo chromosome-level assembly of the Monopterus genome，which provides a valuable resource, not only for further studies in genetics, evolution and development, particularly sex determination, but also for breed improvement of the species.
**Corresponding Author:**	Rongjia Zhou, Ph.D.<br>Wuhan University<br>Wuhan, Hubei CHINA
**Corresponding Author Secondary Information:**	
**Corresponding Author's Institution:**	Wuhan University
**Corresponding Author's Secondary Institution:**	
**First Author:**	Xueya Zhao
**First Author Secondary Information:**	
**Order of Authors:**	Xueya Zhao<br>Majing Luo<br>Zhigang Li<br>Pei Zhong<br>Yibin Cheng<br>Fengling Lai<br>Xin Wang<br>Jiumeng Min<br>Mingzhou Bai<br>Yulan Yang

Powered by Editorial Manager® and ProduXion Manager® from Aries Systems Corporation

	Hanhua Cheng
	Rongjia Zhou, Ph.D.
**Order of Authors Secondary Information:**	
**Response to Reviewers:**	To Reviewer #2:<br><br>The authors have improved the manuscript considerably. I think it was wise to omit the transcriptomics study and focus on the genome assembly, which is quite complex already.<br><br>1. Even though the RNA-seq study has been left out, I would like to suggest paying close attention to the implicit assumptions usually present in RNA-seq analysis packages. In this case, comparing between very different samples (my previous issue 12) will be challenging, and switching to a different normalization method ('re-analysis the RNAseq data by credible FPKM … normalization show the RNAseq are correct') probably does not address this problem.<br><br>Response:<br>Thanks for your suggestions. We will get new samples in reproductive season and sequence and analyze more samples using appropriate algorithms with biological replicates.<br><br>Many of the issues I previously raised on the first part have been addressed, but there are a few I would like to revisit:<br><br>2.Only ~79% of the expected genome size is captured in contigs (634.7 Mbp of 806 Mbp). Therefore, presumably, these contigs contain abundant collapsed repeats, affecting an analysis of repetitive content (…) Repeat abundance will likely be underestimated. (line 188-195 in the new version).<br>Responses: The genome size is 806Mb estimated by K-mer, but only 634.7 Mb was captured in contigs based on the data of second generation sequencing. This most likely results from a lot of repetitive sequences in the genome. The incomplete assemble is the limitation of the second-generation sequencing technology. The repetitive sequences we analyzed are based on the assembled genome. Thus, as you said, repeat sequences would be underestimated. We have discussed the fact in the version.<br><br>The underestimation of repeat abundance is not discussed in the new version. In addition, the BUSCO analysis suggests the 634.7 Mbp genome assembly to be very complete (line 167-173). This suggests the rest of the genome to consist of non-coding DNA, possibly containing many repeats. Unless the 806 Mbp k-mer-based estimate is an overestimate?<br><br>Response: (lines195-199 in the new version)<br>Thanks. We have added a paragraph of discussion on page 7. It should be pointed out that only ~79% of the expected genome size was captured in contigs (634.7 Mb of 806 Mb), and the BUSCO analysis showed that the 634.7 Mb genome assembly was complete. These results suggest that the unassembled genome probably consists of non-coding DNA, possibly contains many repeats. Thus, the repeat abundance would likely be underestimated. The third-generation sequencing will provide a more complete assembly. There would be also a few of errors in k-mer-based estimate of genome size, but it is accepted widely.<br><br>3.Line 136: please rephrase 'they acted', as no evidence on piRNAs is presented. （line 194 in the new version）<br>Responses: Several papers have reported that the piRNAs derived from LINE are very closely related with genome stability. We have just discussed the fact that LINEs was greater than that found in other teleost fish (2-5%), which were involved in guardians for genome defense and germline stability via Piwi-interacting small RNAs (piRNAs) [23].<br><br>4. I did not want to dispute the biology here (but see below), but just wanted to point out that this is a speculative point of discussion. The current study presents no

evidence on piRNAs, therefore the phrasing should be adjusted. The current 'LINEs … were involved in … genome defense … via Piwi-interacting small RNAs' (l193-194) suggests the LINEs were actually studied. So I would suggest rephrasing 'were involved' to 'might be involved'.
Regarding the biology - doesn't a high LINE content suggest genome defense (by piRNA) has been ineffective?

Response to 3 and 4: (Lines 192-193 in the new version)
Thanks. You are right, it is a speculative point of discussion, and we have no evidence. A high LINE content suggests that genome defense (by piRNA) has been ineffective. Our study does not involve any piRNA work. Accordingly, we have revised the sentence as “The percent of LINEs was greater than that found in other teleost fish (2-5%), which might be associated with genome instability[23].”

5.Line 142 /fig S5: 'The gene sizes were similar to those of other teleost fish'. In fact, the figure shows that the Monopterus gene set contains many more short genes and CDSs than other teleosts. Exon lengths are comparable. This suggests truncated genes or gene predictions.
Responses: BUSCO analysis showed that there are some numbers of fragmented and missing BUSCOs, but very low.
Indeed, the BUSCO analysis suggests the assembly is complete. However, looking at figure S5, CDS and mRNA lengths are noticeably shorter in Monopterus. This is not reflected in the averages (table S5).

Response: (Lines 219 in the new version) (Figure S5/table S5)
The gene size estimate is just in an average, so we have changed the sentence using “average gene sizes” to “The average gene sizes were similar to…”
The trend of average value in Table S5 is really reflected by the curves in figure S5, but it is not in accordance with the location of the peak of the frequency, but another feature: the true relationship between them is that the smaller the area of the small fragment in the graph, the larger the average value may be. Taking the distribution of mRNA length as an example, since the y-axis represents the percentage, the areas under the curve of different species are equal. The smaller the area corresponding to the shorter segments is, the larger the area corresponding to the longer segments is, and the higher of the ratio of the longer segments is. In another word, although the largest proportion of the Monopterus mRNA are the shorter fragments, a considerable proportion of the longer fragments truly exist which couldn’t be detected in the graph but affect the average value. This phenomenon is more evident for the zebrafish data.

6.FISH walking (l. 180-203): Is it correct that no FISH evidence is used to assign 142 scaffolds to chromosomes (l. 191)? How will placing these scaffolds using comparative evidence only affect comparative analyses? (line 263-307 in the new version)
Responses: the data of 142 scaffolds were from comparative genomics analysis, not from FISHing experiments. We have discussed this issue, pointed out the possible error rates and problems. (line 295-298 in the new version)
The discussion of this issue is minimal (l. 299). The way I read it, the chromosome-scale assembly may contain large structural errors: 8% of FISH-based placements are inconsistent with synteny (l. 294), therefore conserved synteny should be used with a lot of caution. Yet a further 100 Mb of sequence is placed based on synteny only (l. 302). Some scaffolds are apparently left out (l. 297), but the numbers do not add up? 186 + 142 = 328 scaffolds have been placed (l. 301), but of the 142 some (approximately! 11, l. 297) have been left out?

Response: (Lines 301-309 in the new version)
The 142 scaffolds were predicted by synteny analysis. According to the results of the FISH experiment and collinearity analysis, approximately 8% (~11) of the 142 scaffolds could not be resolved by the conserved synteny prediction due to possible rearrangements in the Monopterus lineage. Accurate mapping of the 142 scaffolds remains to be confirmed by the third-generation sequencing. We have added a paragraph of discussion concerning this.

7.Related: I assume the coloured block in the outer ring of fig 4B are placed scaffolds, and the white spaces gaps. How can these gaps then contain genes, have a GC content, and gene expression (inner rings)?

Powered by Editorial Manager® and ProduXion Manager® from Aries Systems Corporation

Responses: Please see the point 8 above.

The outer ring of figure 4A has a different scale than all the inner content, which is confusing as this is a non-standard way of presenting these data. The gaps (white space) in the outer ring are not represented in the inner graphs. At least please note this in the figure legend.

Response: (Figure 4A in the new version)
The outer ring of previous figure 4A is actually same as figure 3A, so we have deleted in the new figure4 for avoiding of confusing with the other rings. For the new figure 4, we have adopted a set of same position for the assembled sequences (gaps were filled with 50 kb of Ns) for sliding window analysis, so the Circos positions are consistent and clear for all rings. We have also added a stricter set of parameters for the association analysis of GC with gene density to highlight the regions with higher correlation between GC content and gene density. Locations of the regions with higher correlation between GC content and gene density ($R > 0.95$, p-value < 0.01) have been added into the Circos using sliding window analysis.

Some comments on the current version:

L99-100: 'high costs of sequencing', is this still the case?

Response: (Lines 99-100 in the old version but deleted in the new version)
We mean a relative high cost of sequencing compared to the second-generation sequencing technologies. But nowadays the price is not a problem for sequencing, so we have deleted the sentences in the revised manuscript.

L103-104: 'assemble chromosomes, which can produce ultra-long scaffolds': I think this should be the other way around (e.g. assembled ultra-long scaffolds can lead to a chromosome-scale assembly)?

Responses: (Lines 102-103 in the new version)
Thank you for your suggestion. The sentence in line 102-103 “have been used to assemble chromosomes, which can produce ultra-long scaffolds” has been replaced by “have been used to assemble ultra-long scaffolds which can lead to a chromosome-scale assembly”.

L119: Does using two individuals lead to assembly problems (due to heterozygosity)? Presumably not, based on the k-mer analyses (figure S2)?

Responses: (Lines 118 in the new version)
The reads produced from the small-size fractionated genomic library to construct contigs by the second-generation sequencing technology were derived from a single male Monopterus, and the data used for k-mer analysis were also derived from this male Monopterus. The heterozygosity revealed from the K-mer analysis reflects the inner heterozygosity of the homologous chromosomes in an individual. For getting a consistent, high quality assembly, we have used two individuals for complementary assembling of longer scaffolds, which is successful on evidence in BGI.

L153/163: These percentages (91.06%/99.7%) appear to refer to the same analysis? I think the latter refers to the 99.69% in line 153, but those are not 'reads mapping'.

Responses: (Lines 161-162 in the new version)
Yes, 99.7% and 99.69% refer to the same analysis. But, 99.69% refers to “coverage”, while 91.06% refers to “reads mapping”. Therefore, we have revised the original sentences in line 161-162 as "Assembly accuracy was further demonstrated by 91.06 % reads mapping (99.69% coverage) to the reference sequences of the genome".

L235: 'closely related species' -> 'distantly related species', they are in different orders.

Responses: (Lines 239 in the new version)
Thank you for your suggestion. We have replaced the sentence “closely related fish species” with “distantly related fish species”.

Powered by Editorial Manager® and ProduXion Manager® from Aries Systems Corporation

	L325: 'The distribution pattern of the gene density was consistent with the corresponding GC content along the chromosomes'. This refers to the correlation between these two measures. However, figure S9 does not show a clear correlation. In fact, I expect the entire positive correlation to be caused by a few outliers (high GC, high gene content, probably on chromosome 12, see figure 4A). The overall trend is not obvious.<br><br>Responses: (Lines 336-343 in the new version)<br>For the new figure 4, we have adopted slide window analysis, so the Circos positions are consistent and clear for all rings. We have added a stricter set of parameters for the association analysis of GC with gene density to highlight the regions with higher correlation between GC content and gene density. Locations of the regions with higher correlation between GC content and gene density (R > 0.95, p-value < 0.01) have been added into the Circos using the slide window analysis. The results show, as you expect, that some regions with very high correlation coefficients (R> 0.95, p-value <0.01) are detected in the genome, for example, four regions on chromosome 12 (Figure 4A).
**Additional Information:**	
**Question**	**Response**
Are you submitting this manuscript to a special series or article collection?	No
**Experimental design and statistics**<br><br>Full details of the experimental design and statistical methods used should be given in the Methods section, as detailed in our Minimum Standards Reporting Checklist. Information essential to interpreting the data presented should be made available in the figure legends.<br><br>Have you included all the information requested in your manuscript?	Yes
**Resources**<br><br>A description of all resources used, including antibodies, cell lines, animals and software tools, with enough information to allow them to be uniquely identified, should be included in the Methods section. Authors are strongly encouraged to cite Research Resource Identifiers (RRIDs) for antibodies, model organisms and tools, where possible.<br><br>Have you included the information requested as detailed in our Minimum Standards Reporting Checklist?	Yes
**Availability of data and materials**<br><br>All datasets and code on which the	Yes

conclusions of the paper rely must be either included in your submission or deposited in publicly available repositories (where available and ethically appropriate), referencing such data using a unique identifier in the references and in the “Availability of Data and Materials” section of your manuscript.<br><br>Have you have met the above requirement as detailed in our Minimum Standards Reporting Checklist?	

Manuscript Click here to download Manuscript Revised MS2.docx

Chromosome-scale assembly of the Monopterus genome

Running title: The Monopterus genome

Xueya Zhao[1], Majing Luo[1], Zhigang Li[1], Pei Zhong[1], Yibin Cheng[1], Fengling Lai[1], Xin Wang[1], Jiumeng Min[2], Mingzhou Bai[2], Yulan Yang[2], Hanhua Cheng[1]*, Rongjia Zhou[1]*

[1]Hubei Key Laboratory of Cell Homeostasis, Laboratory of Molecular and Developmental Genetics, College of Life Sciences, Wuhan University, Wuhan 430072, P. R. China

[2]BGI-Shenzhen, Shenzhen 518083, P. R. China

*Corresponding authors: Professors Rongjia Zhou and Hanhua Cheng, College of Life Sciences, Wuhan University, Wuhan 430072, P. R. China, Fax: 0086-27-68756253, E-mail: rjzhou@whu.edu.cn, hhcheng@whu.edu.cn

Abstract

Background: The teleost fish *Monopterus albus* is emerging as a new model for biological studies due to its natural sex transition and small genome, in addition to its enormous economic and potential medical value. However, no genomic information for the *Monopterus* is currently available.

Findings: Here, we sequenced and de novo assembled whole genome of the *Monopterus*, and report the *de novo* chromosome assembly by FISH walking assisted by conserved synteny (Cafs) for *Monopterus.* Using Cafs, 328 scaffolds were assembled into 12 chromosomes, which cover genomic sequences of 555 Mb, accounting for 81.3% of the sequences assembled in scaffolds (~689 Mb). A total of 18,860 genes were mapped on the chromosomes and showed a non-random distribution along chromosomes.

Conclusions: We report the first reference genome of the *Monopterus* and provided an efficient Cafs strategy for a *de novo* chromosome-level assembly of the *Monopterus* genome，which provides a valuable resource, not only for further studies in genetics, evolution and development, particularly sex determination, but also for breed improvement of the species.

Key words: whole-genome sequencing, genome assembly, chromosomes, fish

Data Description

Background

The freshwater fish *Monopterus albus* taxonomically belongs to the teleost family Synbranchidae of the order Synbranchiformes. This fish is distributed mainly in southern and eastern Asia, in northern Australia and in the southeastern United States [1]. *Monopterus* is an economically important species for fish production because of its high nutritional value (e.g., high polyunsaturated fatty acid omega-6 levels) and potential medical value. The most influential Chinese pharmacy monograph, the Bencao Gangmu, a compendium of materia medica written by the pharmacist Shi-Zhen Li during the Ming Dynasty (AD 1368~AD 1644), recommended *Monopterus* as a natural drug with medicinal virtues to cure several types of diseases, such as facial paralysis, internal haemorrhoid haemorrhage, and other pathogenic conditions described as being influenced by wind and dampness in Traditional Chinese Medicine.

As an emerging model species in development, genetics and evolution [2], *Monopterus* has the attractive feature of undergoing a sex transition from female to intersex to male during its life [3]. This discovery may have considerable theoretical significance in sex determination [4]. *Monopterus* has a small genome size (~800 Mb) and a minimum chromosome number (n = 12) among teleosts, whose chromosome numbers range from 12 to 223 [5]. In addition, all chromosomes of *Monopterus* are telocentric. Given that a third whole-genome duplication occurred in the whole teleost lineage compared to the two genome duplications that occurred in other land vertebrates [6-8], the speciation and sexual differentiation of *Monopterus* may provide new insights into vertebrate evolution. However, the mechanisms of sex determination in the species remain unknown.

Whole-genome sequencing will provide detailed genetic data for studies of genetics, development and evolution and for the genetic manipulation of *Monopterus*. However, no genetic map is currently available for this species. The whole-genome shotgun approach, with

high throughput and low cost, is based on a second-generation sequencing platform that makes the whole-genome *de novo* assembly of a species possible without the need for a physical map. However, the sequence data produced by the second-generation sequencing technologies are highly fragmented due to the short lengths of the reads. A number of methods for increasing the contiguity and accuracy of *de novo* assemblies have recently been developed. The read length generated from sequencing can be improved by a third-generation sequencing platform, such as single-molecule real-time (SMRT) sequencing, with raw reads of a mean length of 15 kb [9], and nanopore single-molecular sequencing, with raw reads of approximately 5-50 kb [10, 11]. Some strategies for the assembly of a long scaffold have also been developed, for example, BAC/fosmid paired end sequencing, the long-read sequencing (LRseq) [12] approach, contiguity-preserving transposase sequencing (*fragScaff*)[13], and various assembly algorithms [14, 15]. Recently, chromatin interactions, such as Hi-C, have been used to assemble ultra-long scaffolds which can lead to a chromosome-scale assembly; however, a certain amount of error occurs when used for *de novo* assembly[15, 16]. Thus, accurate chromosome-level assembly remains a major challenge.

The most widely used strategy for chromosome-level assembly of the scaffolds generated by the second-generation sequencing is based on a high-density genetic map at chromosome level. Nevertheless, this strategy is feasible only when high-density genetic maps of a species are available. Because there is no genetic map available for *Monopterus,* we have developed an efficient assembly strategy: *de novo* chromosome assembly by FISH walking assisted by conserved synteny (Cafs). Using Cafs technology, which is efficient and cost effective, a precise chromosome-level assembly covering 81.3% of the sequences assembled in scaffolds was produced.

Whole genome sequencing

A whole-genome shotgun strategy and second-generation sequencing technology (Illumina HiSeq 2000 platform) were used to sequence two male *Monopterus*. Genomic DNA was extracted from eels from the Wuhan area in the Yangtze River basin. To reduce the risk of non-

random sequencing, 8 paired-end sequencing libraries with insert sizes of 170 bp, 500 bp, 800 bp, 2 kb, 5 kb, 10 kb, 20 kb, and 40 kb were constructed for the genome. The libraries generated 101.62 GB of sequence data. To reduce sequencing errors in the assembly, sequence reads were filtered to remove low quality reads. After filtering, 78.64 GB (97.6X) of sequence data were retained for the assembly, which ensures a high single-base accuracy (Additional file: Figure S1 and Table S1).

Estimation of genome size

A k-mer was defined as a sequence of k bases in length. The frequency of k-mers in a collection of short, insert-sized reads could be calculated with a 1 bp sliding window. When an optimal amount of data was present, the k-mer frequency followed a Poisson distribution. The k-mer value was used to estimate the genome size, as follows: Genome Size = K_num/Peak_depth, where K_num is the total number of k-mers, and Peak_depth is the expected value of the k-mer depth [17]. The 17-mer distribution obeyed the theoretical Poisson distribution. Finally, we observed that the proportion of heterozygosity in the *Monopterus* genome was small, and estimated that the entire genome comprised 806 Mb, with a GC content of 40.8% (Additional file: Figures S2-S3 and Table S2).

***De novo* genome assembly**

The *Monopterus* genome was *de novo* assembled with the SOAPdenovo software [17] (http://soap.genomics.org.cn). SOAPdenovo employs the de Bruijn graph algorithm to simplify assembly and reduce the computational complexity. Low quality reads were filtered out and potential sequencing errors were removed or corrected with the k-mer frequency methodology. The SOAPdenovo assembly process consisted of three main steps: contig construction, scaffold construction, and gap filling. The sequencing data derived from 2 kb, 5 kb, 10 kb and 20 kb insert size library was used to assemble the scaffolds by SOAPdenovo. The sequencing data derived from 40 kb insert size library were used to build scaffolds with SSPACE (version 1.1) software.

To assess assembly quality and completeness, high quality reads from short-insert-size libraries (75 bp read lengths) were aligned to the assembly with the BWA program [18] (version 0.5.9-r16), with default parameters. Next, SOAPcoverage (version 2.27) was used to calculate sequencing depth. A total of 91.06 % reads could be mapped, and they covered 99.69 % of the assembly, excluding gaps. To further test for possible contigs that might be mis-joined in scaffolds, we analyzed paired-end information. We found that, if contigs were included only when both ends could be uniquely mapped onto the assembly, more than 90.65 % of paired-ends were in the correct orientation and at the expected distance, according to the utilized short-insert-size libraries.

The final assembly comprised 689.5 Mb with contig and scaffold N50 sizes of 22.2 kb and 2.1 Mb, respectively (Table 1). Over 90% of the total sequence was covered by 379 scaffolds; the longest scaffold spanned 11.7 Mb (Table 1). Assembly accuracy was further demonstrated by 91.06 % reads mapping (99.69% coverage) to the reference sequences of the genome and the successful mapping of 321 bacterial artificial chromosomes (BACs), sequenced with Sanger sequencing technology (Additional file: Tables S3).

To evaluate the quality of the assembled genome, we conducted BUSCO (Benchmarking Universal Single-Copy Orthologs) analysis [19] using BUSCO v2.0 with vertebrata_odb9 including 2,586 BUSCOs. Using the BUSCO analysis, 96.5% of BUSCOs were completely detected in the assembled genome (2,464: complete and single-copy, 32: complete and duplicated) among 2,586 tested BUSCOs. The number of fragmented and missing BUSCOs was 56 and 34, respectively. Together, the genome of the *Monopterus* assembled is of high quality.

Repeat elements

Transposable elements (TEs) were identified in the genome with combination of homology-based and de novo approaches. The homology-based approach utilized database Repbase [20] (release 19.06), with RepeatMasker (version 4.0.3) and RepeatProteinMask (from the RepeatMasker package) programs with the default parameters [20]. The de novo approach used

two prediction programs, RepeatModeler [21] (version 1.0.7) and LTR-FINDER [22] (version 1.0.5), to build the de novo repeat libraries based on the genome sequences. Next, contaminations and multi-copy genes were removed from the libraries. Then, the RepeatMasker was used again to find repeats in these repetitive sequence libraries. Finally, we combined all the results generated by these methods. To improve our comparisons to other teleost fishes, we employed the same procedure and parameters to analyze the *Danio rerio*, *Oryzias latipes*, *Gasterosteus aculeatus*, *Tetraodon nigroviridis*, and *Takifugu rubripes*.

For the assembled sequence, the repetitive element content (Additional file: Figure S4) of the *Monopterus* genome (28%) was much lower than that of the zebrafish (61%) and about the same as medaka (29%) genomes, but higher than that of the threespine stickleback (16%) and pufferfish (8-10%) genomes. In the *Monopterus* genome, the main repetitive transposable elements were the DNA transposons and LINEs. At 8%, the LINEs were the largest category of transposable elements. The percent of LINEs was greater than that found in other teleost fish (2-5%), which might be associated with genome instability[23].

It should be pointed out that， only ~79% of the expected genome size was captured in contigs (634.7 Mb of 806 Mb), and the BUSCO analysis showed that the 634.7 Mb genome assembly was complete. These data suggest that the unassembled genome probably consists of non-coding DNA, possibly contains many repeats. Thus, the repeat abundance would likely be underestimated. The third-generation sequencing will provide a more complete assembly.

Genes and function annotation

We used both homology-based and de novo methods to predict genes in the *Monopterus* genome by scanning the local *Monopterus* genome database, which also included RNA-seq data. For the homology-based prediction, protein sequences from *D. rerio*, *O. latipes*, *G. aculeatus*, *T. nigroviridis*, and *T. rubripes* were downloaded from the Ensembl platform[24] (release 75) and aligned with the *Monopterus* genome with the Tblastn program[25]. Accordingly, homologous genomic sequences were input into the Genewise program[26] to align matching proteins. This procedure allowed us to define gene structures. For de novo

prediction, both the Fgenesh[27] and Genscan[28] programs were employed to predict coding genes, with the appropriate parameters. Homology-based and de novo derived gene sets were combined with comprehensive, non-redundant reference gene sets, obtained with the GLEAN platform (http://sourceforge.net/projects/glean-gene/). Genes were corrected by comparisons with the RNA-seq data; these RNA-seqs were mapped to the *Monopterus* genome with the Tophat program, and the Cufflinks program (http://cufflinks.cbcb.umd.edu/) was used to assemble transcripts. After that, we selected 1000 intact genes, defined as gene set "A", which were supported by the homology-based prediction, and passing a fifth-order Markov model, to verify the ORFs of RNA transcripts based on the Hidden Markov Model (HMM). In the *Monopterus*, a total of 24,056 protein-coding genes were predicted (Additional file: Table S4-S6). The average gene sizes were similar to those of other teleost fish (Additional file: Figure S5 and Table S5).

Blastp was used to search for proteins encoded in the *Monopterus* genome by comparing candidate sequences against the SwissProt and TrEMBL databases from UniProt Knowledgebase (UniProtKB) [29]. The annotated motifs and domains in the available databases (ProDom, PRINTS, Pfam, SMART, PANTHER, and PROSITE) were obtained with the InterProScan program [30] (version 4.7). In gene ontology (GO) [31] analyses, gene functions were obtained from the corresponding InterPro entries. Subsets of the GO terms were obtained according to the program of DAVID program (version 6.7) [32]. X-associated genes were annotated based on human GO term list and Z-associated genes were annotated based on chicken GO term list. All genes were also aligned against the KEGG [33] (release 68) protein database. The genes that matched genes in the KEGG database were assumed to be involved in the corresponding signaling pathways. Approximately 80% of the genes could be functionally annotated with homology analysis (Additional file: Figure S6).

De novo chromosome assembly by Cafs-strategy

To assemble chromosomes with accurate sequences from the scaffolds, we developed an efficient assembly strategy without using any genetic map information, Cafs (Figure 1), which

is based on chromosome fluorescent *in situ* hybridization (FISH) and the shared synteny between distantly related fish species.

We first prepared probes of BACs from sequenced clones and PCR fragment pools (8-15 sequences and covers a total length of 20-30 kb on a scaffold) representing scaffolds for chromosome FISH, and performed synteny analysis of these scaffolds by comparing with the fish species (medaka, sticklebacks, *Tetraodon* and *Monopterus*). Second, from the synteny information of the homologous sequences of these scaffolds in the three fish species， probe combination mapping was used to determine 12 linkage groups, corresponding to 12 chromosomes, each with a molecular landmark (Figure 1A). Briefly, group A and B were first discriminated by two unlinked scaffolds labelled with two different colours. If another scaffold was unlinked to the previous two scaffolds, the third scaffold was identified as a marker of group C. Third, based on the predicted syntenic relationship between the related fish species, probes for the candidate scaffolds were co-hybridized with the landmarks of the chromosomes, which have been identified. Scaffolds with no predicted location and that were inconsistent with the predicted location were further determined by co-hybridization with 12 landmarks using dual-colour FISH respectively (Figure 1B). For example, scaffold 58 would be grouped into the F group, as it is linked with scaffold 129, which was the landmark of the F group. Fourth, the loose and long pachytene chromosomes were adopted to determine the location and order relationship of the scaffolds through dual- and three-colour FISH. An original marker was used as a walking start (e.g., scaffold 73), and the location of the second scaffold (e.g., scaffold 186) relative to the original marker was identified by dual-colour FISH. The location of a new scaffold was determined by the known scaffold locations using dual- or three-colour FISH (e.g., scaffolds 72, 123, and 4) (Figure 1C). Finally, because all 12 chromosomes are telocentric, the telomeres of the metaphase chromosome were used as landmarks to determine the directions of the mapped scaffolds on the chromosomes (Figure 1D). The relative position of all scaffolds on chromosomes was determined by the measurement of the signals to the centromere (Figure 1E). The distance values were measured by Image-Pro Plus 6.0, and each value was obtained from an average of more than five cells.

Using the Cafs assembly strategy, we conducted large-scale mapping of the scaffolds on each chromosome. Metaphase chromosomes were prepared according to routine protocols from the *Monopterus* kidney tissue [5]. Meiotic pachytene bivalents were prepared from *Monopterus* testis using a previously described method [34]. The FISH was conducted as previously described [35]. The BAC end sequences were aligned to the genome database by Blat. BACs with two ends aligned to one scaffold, and those ends with sequences with homology to scaffolds greater than 90% were used as probes for FISH. Of the ~747 sequenced clones, 148 BACs could be used as probes for FISH (Table S7). The BACs were confirmed by PCR sequencing from the internal regions of the BACs. 148 BACs and 38 PCR fragment pools (8-15 sequences and covers a total length of 20-30 kb on a scaffold) representing 186 scaffolds (Table S8) were prepared as probes for chromosome FISH.

Before the hybridization experiment, we performed a genome-wide synteny analysis to compare these fish species. We constructed a reference map using the syntenic relationship among the genomes of medaka, stickleback and *Tetraodon* to help map the scaffolds on the *Monopterus* chromosomes. The syntenic blocks between *Monopterus* and other fishes were aligned by Lastz (Blastz) [36] with parameters of T=2 and Y=3400. Furthermore, we used Blat to search for homologous sequences among medaka, sticklebacks, *Tetraodon* and *Monopterus* in order to fill the gap sequences of blocks in the reference map. If two homologous sequences were linked in all three close species, we defined the corresponding scaffolds in *Monopterus* as predicted linked scaffolds.

Under the guidance of the synteny of the homologous sequence of these 186 scaffolds in other three fish species, 78 probes combinations of co-hybridization were performed to identify 12 linkage groups, each with a molecular landmark (Figure 2A). We then conducted the walking in a range of 11-22 steps per chromosome (Figure 2B; Additional file: Figure S7). A total of 186 scaffolds were assembled into 12 pachytene chromosomes through step-by-step combination hybridization of the probes using the above-mentioned 148 BACs and 38 PCR fragment pools (Figure 3A). We then determined the orientation of each chromosome by dual-colour FISH on metaphase chromosomes using the telomere as a morphological landmark

(Additional file: Figure S8). Of these mapped scaffolds, 92% (99/108) were consistent with the shared synteny between the related fish species (medaka, sticklebacks and *Tetraodon*). From the synteny analysis, 142 scaffolds were predicted and further assembled into 12 chromosomes respectively. According to the results of the FISH experiment and collinearity analysis, approximately 8% (~11) of the 142 scaffolds could not be resolved by the conserved synteny prediction due to possible rearrangements in the *Monopterus* lineage. Accurate mapping of the 142 scaffolds remains to be confirmed by the third-generation sequencing. The current assembly, particularly the locations of the 186 scaffolds, have great implications for comparative genomics and evolution studies. As the genome of *Monopterus* has the least number of chromosomes in the teleosts, the determination of 12 linkage groups of the *Monopterus* genome is crucial for the studies of large-scale chromosome recombination events (e.g. chromosome fusion or fission) in the teleost evolution.

We finally integrated 328 scaffolds into the reference genome. These mapped scaffolds consisted of 455 Mb determined by FISH and 100 Mb determined by syntenic analysis, which covers genomic sequences of a total length of 555 Mb, accounting for 81.3% of the sequences assembled in scaffolds (689.5 Mb). Based on the assembly, a total of 18,660 protein-coding genes were annotated with location information on the chromosomes (Table 2). For example, there are 87 protein-coding genes on scaffold 72, which was located on chromosome 5 (Figure 3B). These data indicate that a *de novo* chromosome-level assembly of the *Monopterus* genome was produced using the Cafs strategy.

Chromosome-wide gene clustering

To further investigate gene clustering along the chromosomes, we calculated the gene density per chromosome. The average gene density in the genome was 33.6 genes per Mb, with the maximum gene density on chromosome 12, which is the shortest chromosome, and the minimum gene density on chromosome 9 (Table 2). Further sliding window analysis showed that there was also biased distribution of the gene density within the chromosome (Figure 4A). Using a 1-Mb window size and 100-kb step size, the maximum gene density in the genome was detected from nt 22,200,001 to nt 23,200,000 on chromosome 10, which contains 71 genes, in

comparison with an average of 33.6 genes per Mb in the genome (Figure 4B).

We then tested the statistical significance of pairing correlations between gene density and GC content. As these parameters are not normally distributed, we used the non-parametric Spearman correlation test on the ranks of the paired quantities. Correlation analyses were performed with R software (www.r-project.org). The R package ggplot2 was used to draw scatterplots and boxplots. The distribution pattern of the gene density was consistent with the corresponding GC content along the chromosomes (Figure 4A; Additional file: Figure S9A). In order to see whether particular regions contribute to the positive correlation, we used the sliding window analysis to divide the genome into 4 Mb small regions and calculated the correlation coefficient between GC content and gene density in each 4 Mb-region. Using a 3-Mb window size and 100-kb step size, we divided the genomes into 5196 regions with each size of 4 Mb. The analysis showed that there are a large proportion (68.21%) of 4 Mb regions with lower correlations than 0.7 (Additional file: Figure S9B). Some regions with very high correlation coefficients ($R > 0.95$, p-value <0.01) are detected in the genome, for example, four regions on chromosome 12 (Figure 4A).

To investigate whether the distribution of the genes along the chromosomes is non-random, we computed the probability of ≥ actual numbers of ridges under a random permutation of the gene positions following previous method [37]. A ridge was used to describe a chromosome region with high gene density, which is thus defined as at least W consecutive windows, each containing a gene number higher than H. Thus, the ridge is determined by two parameters: cutoff 1 (C_H), gene number per window, and cutoff 2 (C_W), number of consecutive windows. The actual ridge numbers (N) in the genome were calculated under C_H and C_W by sliding window analysis. We used the following calculation parameters to set up a null model: suppose we have a random permutation of $X_1, X_2,..., X_i$ in the range of 1 to S; i, gene number on the chromosome; S, length of the chromosome; and $X_1, X_2,..., X_i$, gene locations on the chromosome. With the same cutoff values under actual conditions (C_H and C_W), we can obtain a ridge number (n) under the null model. We can compute the frequency (f) when $n \geq N$ by permutation 10,000 times. If $f = 0$, the p-value $< 10^{-4}$, or the p-value = f/10000. For all of the

cutoff C_H and C_W combinations, we calculated the *p*-value under different window sizes of 0.2, 0.3, 0.5, 1, 2, and 3 Mb respectively.

Using a combination of the two cutoffs, the numbers of the ridges of each chromosome can be identified. For example, using cutoffs of 40 genes per Mb and 5 consecutive windows, 7 ridges on chromosome 10 were identified (Figure 4B), and 90 ridges were identified in the genome (Figure 4C). The probability of the observed ridges occurring in random permutations of gene positions was very low (p-value < 10^{-4}) (Figure 4C), confirming non-random and clustering distribution of genes along the chromosomes. Probabilities (ridges numbers under a random permutation ≥ ridges numbers in the *Monopterus* genome) for a series of cutoff sets and different window sizes were also calculated and the results showed that there were significant differences in ridges numbers between the *Monopterus* genome and random permutations of gene positions (Figure 4C; Additional file: Figure S10). The ridge numbers of high gene density directly reflect the clustering of genes along the chromosomes. These analyses suggest that the ridge pattern on the chromosomes probably represents a higher-order structure in the genome.

Data availability statement

The genome data from this study have been deposited at DDBJ/EMBL/GenBank under accession number AONE00000000, and the raw transcriptome data have been submitted to NCBI Gene Expression Omnibus (GEO; http://www.ncbi.nlm.nih.gov/geo/) under accession number GSE43649.

Additional files

Supplemental Figure S1. Sequencing depth distribution of the *Monopterus* genome.

Supplemental Figure S2. Genome size estimation using 17-mer.

Supplemental Figure S3. The GC distribution of the *Monopterus* genome.

Supplemental Figure S4. Divergence distribution of the classified TE elements.

Supplemental Figure S5. Comparisons of predicted coding genes of *Monopterus* with other teleost fishes.

Supplemental Figure S6. The Gene Ontology of the *Monopterus* genes.

Supplemental Figure S7. Localization of each scaffold on chromosomes by FISH-walking strategy.

Supplemental Figure S8. Orientation of each linkage group on metaphase chromosomes.

Supplemental Figure S9. Correlation coefficient of GC content with gene density.

Supplemental Figure S10. Statistical tests of numbers of gene density ridges in the genome corresponding to background noise (null model) in different window sizes (0.2, 0.3, 0.5, 1, 2, 3 Mb).

Supplemental Table S1. Statistics of sequencing.

Supplemental Table S2. Statistics of genome from 17-mer.

Supplemental Table S3. Statistics of mapping.

Supplemental Table S4. Statistics of predicted coding genes.

Supplemental Table S5. Comparisons of predicted coding genes of *Monopterus* with other teleost fishes.

Supplemental Table S6. Annotated classification of the *Monopterus* genes.

Supplemental Table S7. Alignments of BAC ends to reference genome.

Supplemental Table S8. Information of FISH probes synthesized by PCR.

List of Abbreviations

BAC: bacterial artificial chromosome; bp: base pair; BUSCO: Benchmarking Universal Single-Copy Orthologs; Cafs: chromosome assembly by FISH walking assisted by conserved synteny; DAPI :49-6-diamidino-2-phenylindole; FISH: fluorescent *in situ* hybridization; Gb: giga base; GO: gene ontology; H&E: haematoxylin and eosin; Hi-C: high-throughput/resolution chromosome conformation capture; kb: kilo base; KEGG: Kyoto Encyclopedia of Genes and Genomes; LINEs: long interspersed nuclear elements; LRseq: long-read sequencing; Mb: mega base; PAC: plant artificial chromosome; piRNAs: Piwi-interacting small RNAs; SMRT : single-molecule real-time; TEs: Transposable elements.

Acknowledgements

This work was supported by the National Natural Science Foundation of China, National Key Technologies R&D Program and Hubei Province Science and Technology Project.

Author contributions

Conceptualization: Rongjia Zhou.

Funding acquisition: Rongjia Zhou, Hanhua Cheng.

Investigation: Xueya Zhao, Majing Luo, Zhigang Li, Yibin Cheng, Fengling Lai, Xin Wang, Jiumeng Min, Mingzhou Bai, Yulan Yang.

Methodology: Xueya Zhao, Majing Luo, Zhigang Li, Pei Zhong.

Supervision: Rongjia Zhou, Hanhua Cheng.

Validation: Xueya Zhao, Rongjia Zhou.

Writing – original draft: Xueya Zhao, Rongjia Zhou.

Writing – review & editing: Xueya Zhao, Rongjia Zhou.

Ethics statement

Monopterus were obtained from Hubei, China. All animal experiments and methods were performed in accordance with the relevant approved guidelines and regulations, as well as under the approval of the Ethics Committee of Wuhan University.

Competing interests

The authors declare that they have no competing interests

Figure legends

Figure 1 Overview of *de novo* chromosome assembly by FISH walking assisted by conserved synteny. A: Identification of 12 linkage groups by probe combination mapping. FISH probes are hybridized on pachytene chromosomes. Red and green dots indicate scaffold locations. B: Synteny-assisted scaffold mapping. Each candidate BAC (scaffold) is co-hybridized with 12 landmarks by dual-colour FISH respectively. Synteny-supported/non-supported scaffolds are determined by FISH. C: Determination of scaffold order on chromosome by FISH walking. (I) The order of two scaffolds is identified by dual-colour FISH if both of them are on one side of the chromosome. (II) If the scaffolds are in the centre of the chromosome, three-colour FISH

is applied to determine their order. (III) The order of some scaffolds (labelled with one colour) could be identified by three signals dual-colour FISH, when their two neighbouring scaffolds (labelled with another colour) have been determined. D: Identification of orientation of linkage groups on metaphase chromosomes. Telomeres and centromeres can be observed on the metaphase chromosomes. E: Localization of scaffolds is determined by calculating the corresponding distances to the centromere.

Figure 2 Chromosome assembly by Cafs. A: FISH images show 12 molecular landmarks corresponding to 12 chromosomes. Green signals indicate the landmarks labelled by digoxigenin and detected with FITC. Each chromosome is determined by a landmark. Chromosomes are stained by DAPI (blue). B: Localization of each scaffold on chromosome 5 by FISH walking strategy. FISH images and corresponding scaffold order from (a') to (m') are shown in the left panels. A three-colour FISH image (g') in the upper right indicates the relative order of scaffolds 4 (yellow, FITC+Cy3), 30 (green, FITC) and 99 (red, Cy3) on chromosome 5. Probes (red dots) and their locations on scaffolds are used to assemble chromosome 5.

Figure 3 Chromosome-scale assembly of the *Monopterus* genome. A: Each chromosome is assembled with scaffolds and their order from telomere (down end) to centromere (up end). The grey and purple cylinders represent the anchored scaffolds. The segments in light blue between two neighbouring scaffolds indicate gaps. Sticks with a red head anchored on each scaffold indicate the positions of the BACs used as probes. Scale bar, 0-1. B: Scaffold 72 with 87 genes (blue bars) and their location on chromosome 5 is highlighted.

Figure 4 Chromosome-wide gene clustering. A: Circos is used to plot the assembled chromosomes, GC content and gene density. The inner scale is 2 Mb. The strips in the outer circle indicate the scaffolds packaged into chromosomes filled the gaps with 50 kb of Ns. The inner dark grey ridges show the moving GC percentage, and the inner grey ridges show the moving number of the genes at a window size of 1 Mb. The green sticks indicate the location of the regions with high correlation between GC content with gene density ($R > 0.95$, p-value < 0.01). B: Distribution of gene clusters (ridges) on chromosome 10. Curves indicate the

moving numbers of genes at a window size of 1 Mb (step = 100 kb). The windows with a maximum gene density from nt 22200001 to 23200000 on chromosome 10, which contains 71 genes, are shown in the lower panel. Green boxes highlight ridges in which there are at least 5 consecutive moving windows with a lower limit of 40 genes per window. C: Statistical tests of numbers of gene density ridges in the genome corresponding to background noise (null model). The heat map in the lower panel shows *p*-values in the significance test of observed ridge numbers against the null model (10,000 independent permutations of gene positions). The *x*-axis indicates the cutoff values of numbers of consecutive moving windows, which reflects the extent of the clustering. The *y*-axis indicates the cutoff values of gene numbers within a certain window size (step 100 kb), which reflects the degree of intensity of the clusters. Green lines represent the average gene number in a certain window size. The upper panel highlights a significance test at the condition of two cutoff values, gene density (40/Mb) and consecutive window numbers (5). Red dots represent the number of observed ridges in the genome. Boxplots (black) represent distribution of the ridge numbers in 10,000 independent permutations of gene positions in a random fashion.

References

1. Collins TM, Trexler JC, Nico LG, Rawlings TA. Genetic Diversity in a Morphologically Conservative Invasive Taxon: Multiple Introductions of Swamp Eels to the Southeastern United States. Conserv Biol. 2002; 16:1024-35.
2. Cheng HH, Guo YQ, Yu QX, Zhou RJ. The rice field eel as a model system for vertebrate sexual development. Cytogenet Genome Res. 2003; 101:274-7.
3. Liu CK. Rudimentary hermaphroditism in the symbranchoid eel, Monopterus javanensis. Sinensia. 1944; 15:1-8.
4. Bullough WS. Hermaphroditism in the lower vertebrates. Nature. 1947; 160:9-11.
5. Yu XJ, Zhou T, Li YC, Li K, Zhou M. Chromosomes of Chinese fresh-water fishes. Beijing Science Press. 1989:1-148.
6. Zhou RJ, Cheng HH, Tiersch TR. Differential genome duplication and fish diversity. Rev Fish Biol Fisher. 2002; 11:331-7.
7. Christoffels A, Koh EG, Chia JM, Brenner S, Aparicio S, Venkatesh B. Fugu genome analysis provides evidence for a whole-genome duplication early during the evolution of ray-finned fishes. Mol Biol Evol. 2004; 21:1146-51.
8. Jaillon O, Aury JM, Brunet F, Petit JL, Stange-Thomann N, Mauceli E, et al. Genome duplication in the teleost fish Tetraodon nigroviridis reveals the early vertebrate proto-karyotype. Nature. 2004; 431:946-57.
9. Huddleston J, Ranade S, Malig M, Antonacci F, Chaisson M, Hon L, et al. Reconstructing

complex regions of genomes using long-read sequencing technology. Genome Res. 2014; 24:688-96.

10. Goodwin S, Gurtowski J, Ethe-Sayers S, Deshpande P, Schatz MC, McCombie WR. Oxford Nanopore sequencing, hybrid error correction, and de novo assembly of a eukaryotic genome. Genome Res. 2015; 25:1750-6.

11. Chaisson MJ, Huddleston J, Dennis MY, Sudmant PH, Malig M, Hormozdiari F, et al. Resolving the complexity of the human genome using single-molecule sequencing. Nature. 2015; 517:608-11.

12. Voskoboynik A, Neff NF, Sahoo D, Newman AM, Pushkarev D, Koh W, et al. The genome sequence of the colonial chordate, Botryllus schlosseri. Elife. 2013; 2:e00569.

13. Adey A, Kitzman JO, Burton JN, Daza R, Kumar A, Christiansen L, et al. In vitro, long-range sequence information for de novo genome assembly via transposase contiguity. Genome Res. 2014; 24:2041-9.

14. Kim J, Larkin DM, Cai Q, Asan, Zhang Y, Ge RL, et al. Reference-assisted chromosome assembly. Proc Natl Acad Sci U S A. 2013; 110:1785-90.

15. Putnam NH, O'Connell BL, Stites JC, Rice BJ, Blanchette M, Calef R, et al. Chromosome-scale shotgun assembly using an in vitro method for long-range linkage. Genome Res. 2016; 26:345-50.

16. Burton JN, Adey A, Patwardhan RP, Qiu R, Kitzman JO, Shendure J. Chromosome-scale scaffolding of de novo genome assemblies based on chromatin interactions. Nat Biotechnol. 2013; 31:1119-25.

17. Li RQ, Fan W, Tian G, Zhu HM, He L, Cai J, et al. The sequence and de novo assembly of the giant panda genome. Nature. 2010; 463:311-7.

18. Li H, Durbin R. Fast and accurate short read alignment with Burrows-Wheeler transform. Bioinformatics. 2009; 25:1754-60.

19. Simao FA, Waterhouse RM, Ioannidis P, Kriventseva EV, Zdobnov EM. BUSCO: assessing genome assembly and annotation completeness with single-copy orthologs. Bioinformatics. 2015; 31:3210-2.

20. Jurka J, Kapitonov VV, Pavlicek A, Klonowski P, Kohany O, Walichiewicz J. Repbase Update, a database of eukaryotic repetitive elements. Cytogenet Genome Res. 2005; 110:462-7.

21. Price AL, Jones NC, Pevzner PA. De novo identification of repeat families in large genomes. Bioinformatics. 2005; 21:i351-i8.

22. Xu Z, Wang H. LTR_FINDER: an efficient tool for the prediction of full-length LTR retrotransposons. Nucleic Acids Res. 2007; 35:W265-8.

23. Reuter M, Berninger P, Chuma S, Shah H, Hosokawa M, Funaya C, et al. Miwi catalysis is required for piRNA amplification-independent LINE1 transposon silencing. Nature. 2011; 480:264-7.

24. Flicek P, Ahmed I, Amode MR, Barrell D, Beal K, Brent S, et al. Ensembl 2013. Nucleic Acids Res. 2013; 41:D48-55.

25. Mount DW. Using the Basic Local Alignment Search Tool (BLAST). CSH Protoc. 2007; 2007:pdb top17.

26. Birney E, Clamp M, Durbin R. GeneWise and genomewise. Genome Res. 2004; 14:988-95.

27. Burge C, Karlin S. Prediction of complete gene structures in human genomic DNA. J Mol Biol. 1997; 268:78-94.

28. Salamov AA, Solovyev VV. Ab initio gene finding in Drosophila genomic DNA. Genome Res. 2000; 10:516-22.
29. Bairoch A, Apweiler R. The SWISS-PROT protein sequence database and its supplement TrEMBL in 2000. Nucleic Acids Res. 2000; 28:45-8.
30. Mulder N, Apweiler R. InterPro and InterProScan: tools for protein sequence classification and comparison. Methods Mol Biol. 2007; 396:59-70.
31. Ashburner M, Ball CA, Blake JA, Botstein D, Butler H, Cherry JM, et al. Gene Ontology: tool for the unification of biology. Nat Genet. 2000; 25:25-9.
32. Huang da W, Sherman BT, Lempicki RA. Systematic and integrative analysis of large gene lists using DAVID bioinformatics resources. Nat Protoc. 2009; 4:44-57.
33. Kanehisa M, Goto S. KEGG: kyoto encyclopedia of genes and genomes. Nucleic Acids Res. 2000; 28:27-30.
34. Yu QX, Fan LC, Cui JX, Ren XH, Li K, Yu XJ. High resolution G-binding and idiogram on pachytene bivalents of rice field eels. Sci China (B). 1994:1090.
35. Henegariu O, Dunai J, Chen XN, Korenberg JR, Ward DC, Greally JM. A triple color FISH technique for mouse chromosome identification. Mamm Genome. 2001; 12:462-5.
36. Schwartz S, Kent WJ, Smit A, Zhang Z, Baertsch R, Hardison RC, et al. Human-mouse alignments with BLASTZ. Genome Res. 2003; 13:103-7.
37. Caron H, van Schaik B, van der Mee M, Baas F, Riggins G, van Sluis P, et al. The human transcriptome map: clustering of highly expressed genes in chromosomal domains. Science. 2001; 291:1289-92.

Table 1. Statistics of the assembly of the *Monopterus* genome.

	**Contigs***		**Scaffolds**	
	**Size (bp)**	**Number**	**Size (bp)**	**Number**
N90	4,762	33,115	368,242	379
N80	8,655	23,414	775,515	254
N70	12,290	17,275	1,109,624	180
N60	16,188	12,785	1,519,751	128
N50	22,239	8,438	2,106,322	87
Longest	159,913	----	11,676,616	----
Total size	634,655,961	----	689,524,511	----
Total number(>=100bp)	----	117,579	----	62,978
Total number (>=2kb)	----	44,314	----	2,360

*The contig size was the final size after filling intra-scaffold gaps. Contigs with lengths shorter than 100bp were not included in the statistics.

Table 2. Assembly statistics for each chromosome

Chromosome	Chromosome size (Kb)	Scaffolds No.	Gene No.	Gene density (n/10Mb)
**1**	75908.7	33	2264	298
**2**	65103.9	32	2133	328
**3**	51637.3	21	1872	363
**4**	51162.1	30	1791	350
**5**	50080.0	27	1517	303
**6**	48093.1	27	1659	345
**7**	42410.1	29	1500	354
**8**	41999.7	30	1456	347
**9**	41928.7	23	1241	296
**10**	34690.8	30	1262	364
**11**	29285.5	23	1086	371
**12**	22774.4	23	879	386
**Total**	555074.3	328	18660	336

Figure 1

Click here to download Figure Figure 1.tif

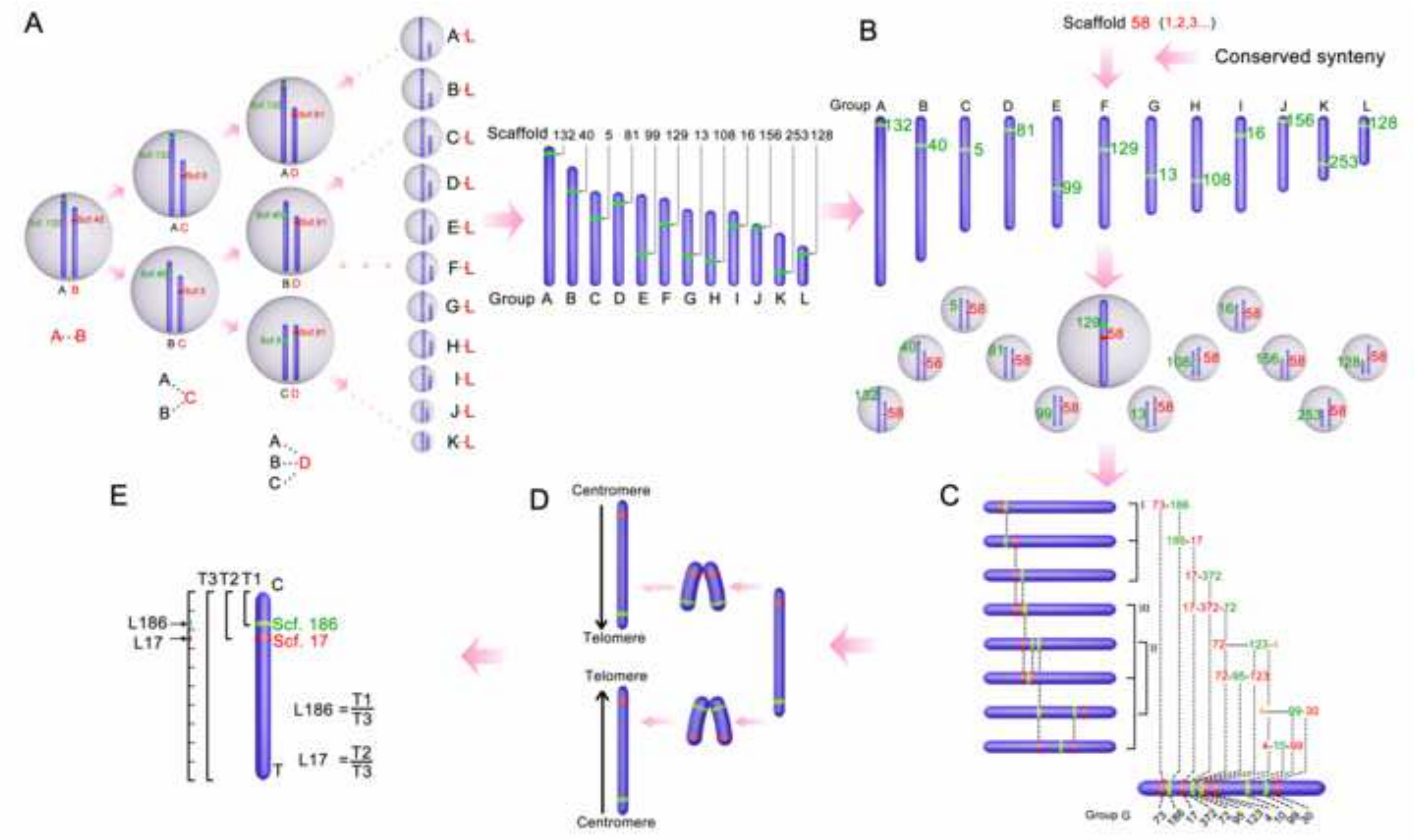

Figure 2

Click here to download Figure Figure 2 .tif

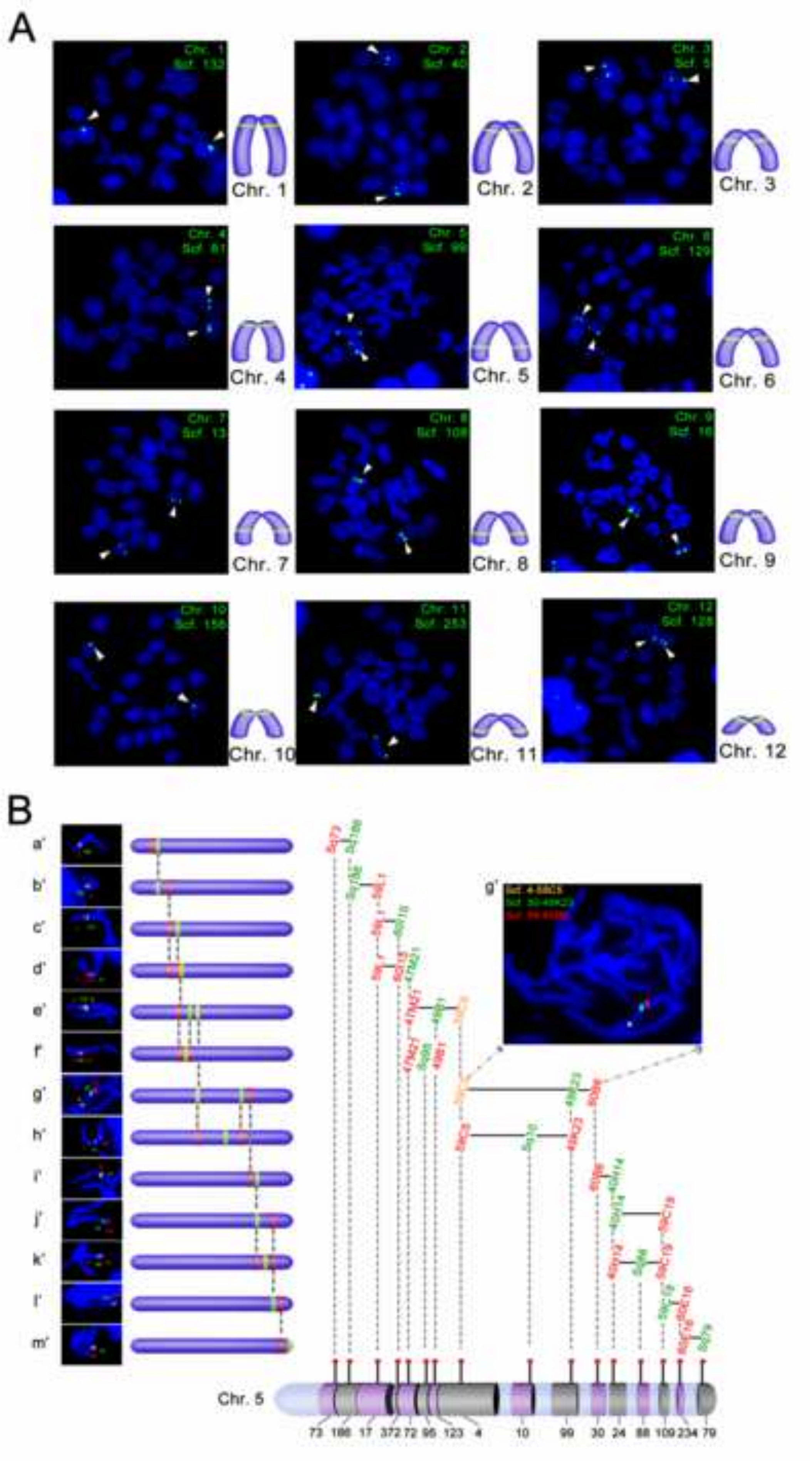

Figure 3

Click here to download Figure Figure 3.tif

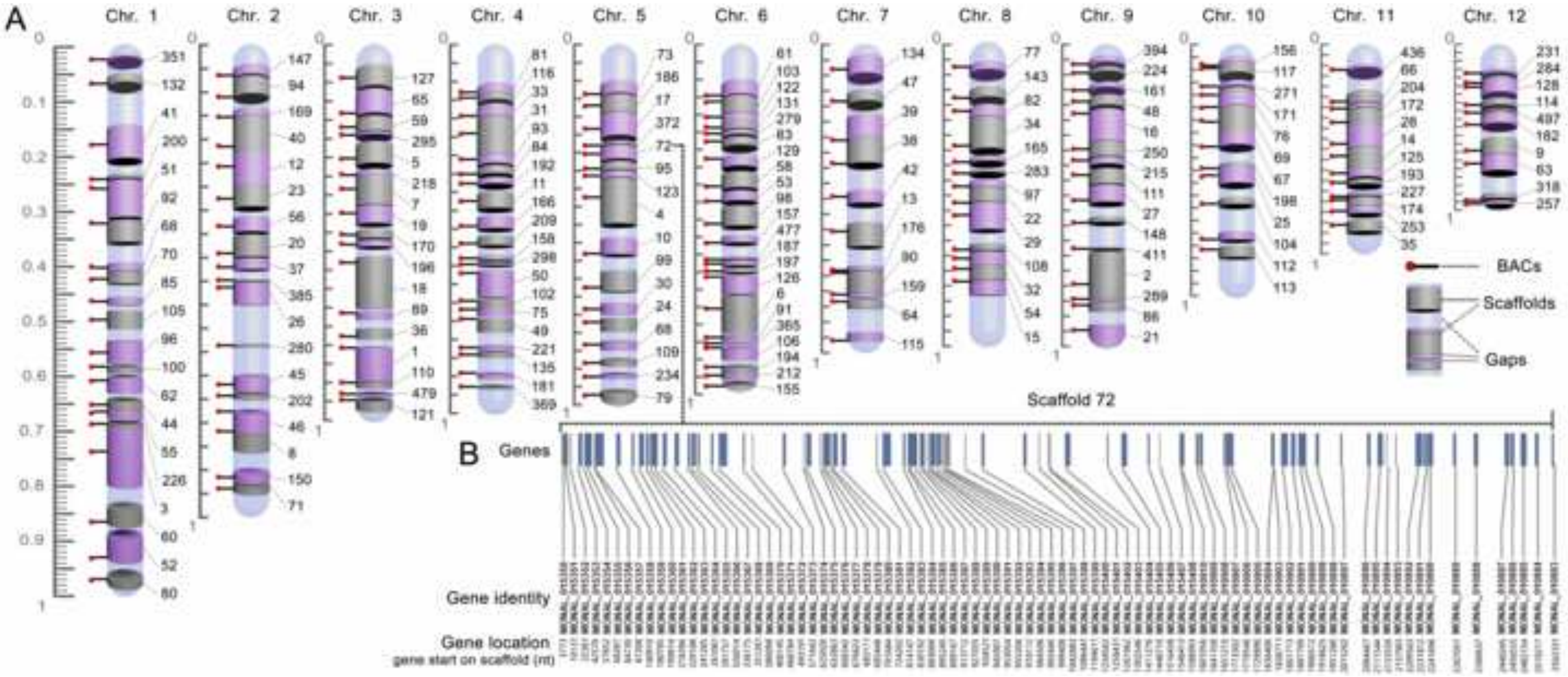

Figure 4

Click here to download Figure Figure 4.tif

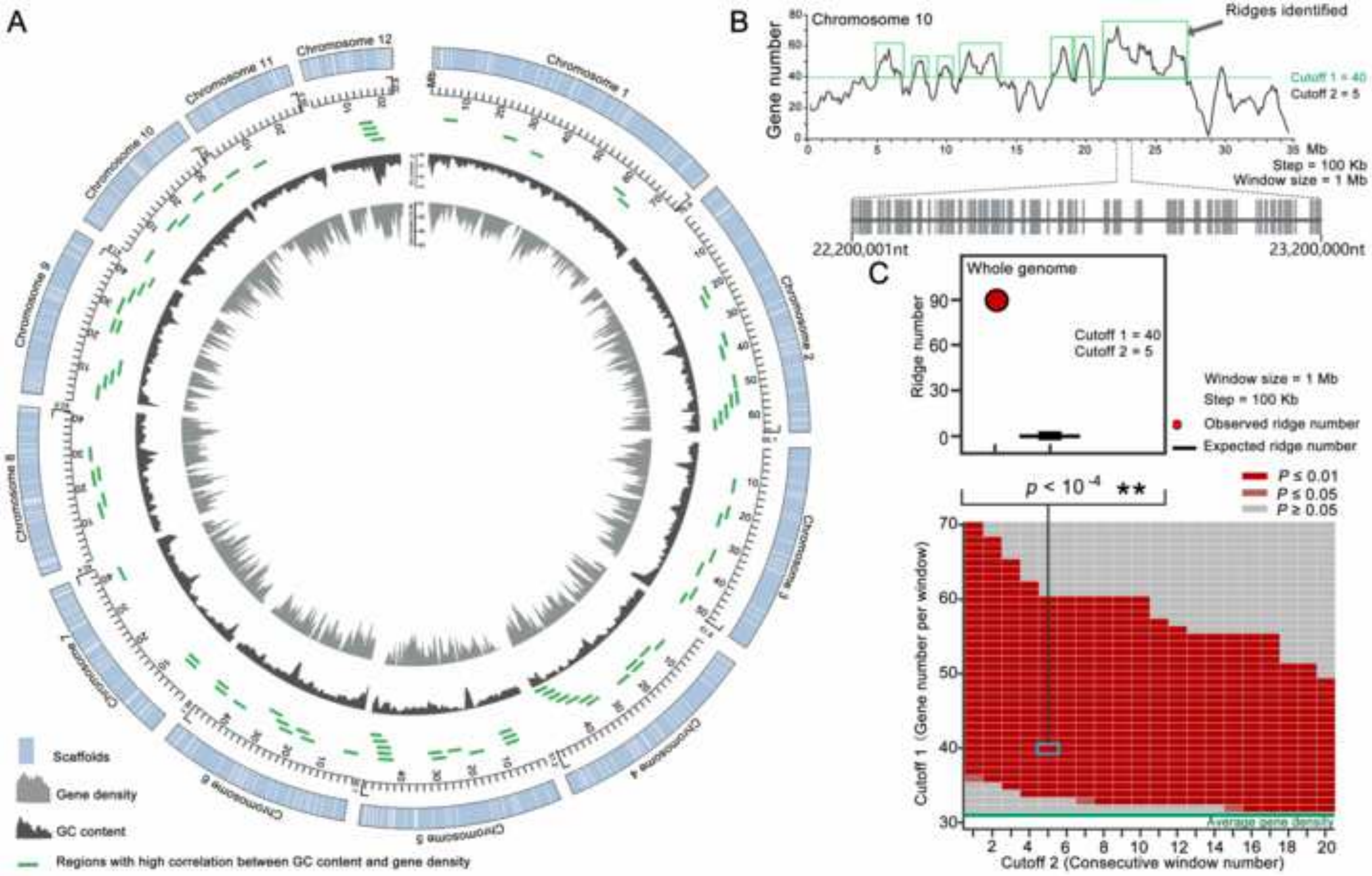

Click here to access/download
Supplementary Material
Additional files-revised.docx

Since 1893

Wuhan University

Laboratory of Molecular and Developmental Genetics, College of Life

Wuhan 430072, China

Tel: (0086)27-68756253, E-mail: rjzhou@whu.edu.cn

Editors
GigaScience

March 6, 2018

Dear Editor Hans,

Many thanks for your handling of my manuscript GIGA-D-17-00210R1 entitled "**Chromosome-scale assembly of the *Monopterus* genome**" for your consideration for publication in *GigaScience*.

Following both your suggests and the reviewer's comments, we have revised the manuscript. We have paid pay particular attention to the points regarding scaffold placements, and provided a new, consistent and clear figure 4 with position information. The current manuscript has been also corrected in standard English.

We have revised the manuscript following comments of reviewer #2 seriously, and provided a file to list **Point-to-point responses to the reviewer.**

After revised, we have submitted it in your online system. Would you please check, and let me know if any problem.

The manuscript has not been submitted or is under consideration for publication elsewhere now. All authors agree to submit to your journal.

I look forward to hearing from you. Thank you for your consideration!

With best regards,

Rongjia Zhou, Ph.D.
Luojia Distinguished Professor
Genetics
