# Supplementary material for: Chromosome-scale assembly of the Monopterus genome
Source: Gigascience. 2018 Apr 24;7(5):giy046. doi: 10.1093/gigascience/giy046 (PMC5946948; doi:10.1093/gigascience/giy046)
Supplement: GIGA-D-17-00210_Revision_3.pdf [file giy046_giga-d-17-00210_revision_3.pdf]

GigaScience
Chromosome-scale assembly of the Monopterus genome
--Manuscript Draft--

**Manuscript Number:**	GIGA-D-17-00210R3
**Full Title:**	Chromosome-scale assembly of the Monopterus genome
**Article Type:**	Data Note
**Funding Information:**	National Natural Science Foundation of China (31571280) — Professor Rongjia Zhou<br>National Natural Science Foundation of China (31471182) — Prof. Hanhua Cheng
**Abstract:**	Background: The teleost fish Monopterus albus is emerging as a new model for biological studies due to its natural sex transition and small genome, in addition to its enormous economic and potential medical value. However, no genomic information for the Monopterus is currently available.<br><br>Findings: Here, we sequenced and de novo assembled whole genome of the Monopterus, and report the de novo chromosome assembly by FISH walking assisted by conserved synteny (Cafs) for Monopterus. Using Cafs, 328 scaffolds were assembled into 12 chromosomes, which cover genomic sequences of 555 Mb, accounting for 81.3% of the sequences assembled in scaffolds (~689 Mb). A total of 18,660 genes were mapped on the chromosomes and showed a non-random distribution along chromosomes.<br><br>Conclusions: We report the first reference genome of the Monopterus and provided an efficient Cafs strategy for a de novo chromosome-level assembly of the Monopterus genome，which provides a valuable resource, not only for further studies in genetics, evolution and development, particularly sex determination, but also for breed improvement of the species.
**Corresponding Author:**	Rongjia Zhou, Ph.D.<br>Wuhan University<br>Wuhan, Hubei CHINA
**Corresponding Author Secondary Information:**	
**Corresponding Author's Institution:**	Wuhan University
**Corresponding Author's Secondary Institution:**	
**First Author:**	Xueya Zhao
**First Author Secondary Information:**	
**Order of Authors:**	Xueya Zhao<br>Majing Luo<br>Zhigang Li<br>Pei Zhong<br>Yibin Cheng<br>Fengling Lai<br>Xin Wang<br>Jiumeng Min<br>Mingzhou Bai<br>Yulan Yang

Powered by Editorial Manager® and ProduXion Manager® from Aries Systems Corporation

	Hanhua Cheng
	Rongjia Zhou, Ph.D.
**Order of Authors Secondary Information:**	
**Response to Reviewers:**	Point-to-point responses to Reviewer:<br><br>Reviewer #2:<br>I thank the authors for improving the manuscript's clarity to the point where it is in principle publishable. Please find below a few final suggestions for minor additional clarifications.<br><br>On my issue of using multiple eels for sequencing, assembly and k-mer analysis: The answer provided is satisfactory, however this is not yet clear from the manuscript. The line (118) referred to just mentions 'eels', when the answer precisely specifies how many animals were used, why and for which analyses.<br><br>Response: (lines 116-120 and lines 131-134 in the new version)<br>Thanks for your suggestion. We have added the description for the usage of multiple eels and explanation for k-mer in lines 116-120 and lines 131-134 respectively as follows:<br>"To reduce the risk of non-random sequencing, 8 paired-end sequencing libraries with insert sizes of 170 bp, 500 bp, 800 bp, 2 kb, 5 kb, 10 kb, 20 kb and 40 kb were constructed. Of them, the library with 40 kb inserts was from second individual for assembling of long scaffolds. These libraries generated 101.62 GB of sequence data."<br>"The data used for k-mer analysis were derived from the male which was used to construct the genome sequencing libraries. The heterozygosity revealed from the k-mer analysis reflects the inner heterozygosity in an individual."<br><br>On my issue of placing 142 scaffolds using synteny only: this needs very careful phrasing. I think the point is that 8% of scaffolds could not be placed based on FISH, and that therefore perhaps 8% of 142 (therefore approximately 11) scaffolds placed by synteny only can be expected to be incorrectly placed. 'Can be expected' is quite different from 'could not be resolved' as stated in the answer. In the manuscript, I suggest rephrasing line 301 to something like 'Based on the results of the FISH experiments and collinearity analyses, we can expect approximately 8% placement errors amongst these 142 scaffolds.'<br><br>Response: (line 300-301 in the new version)<br>Thanks for your suggestion. We have replaced "According to the results of the FISH experiment and collinearity analysis, approximately 8% (~11) of the 142 scaffolds could not be resolved by the conserved synteny prediction due to possible rearrangements in the Monopterus lineage." with "Based on the results of the FISH experiments and collinearity analyses, we can expect approximately 8% placement errors amongst these 142 scaffolds."<br><br>Several bioinformatics tools could be properly cited, e.g. SSPACE (L146), Blat (L272), Cufflinks (L214), Circos (L471).<br><br>Response: SSPACE (line 147), Blat (line 271), Cufflinks (line 214), Circos (line 341) in the new version.<br>Thanks for your suggestion. We have added the corresponding references in the version.<br>——SSPACE (version 1.1)-reference 17<br>——Blat (version blat_34)-reference 37<br>——Cufflinks (version cufflinks-2.2.1)-reference 29<br>——Circos (version 0.69)-reference 39<br>In addition, we have added the description of Circos in line 341 of the article, "In this analysis, the Circos is used to plot the assembled chromosomes, GC content and gene density."<br><br><br>Line 98: Reference 11 is probably not appropriate for nanopore sequencing?

	Response: (line 95 in the new version)<br>Thanks. We have deleted the reference 11, where the reference 10 is appropriate and enough for nanopore sequencing information.<br><br><br>Line 108: 'the second-generation sequencing' -> second-generation sequencing. Same for L199, L304.<br><br>Response: (line 90, 105, 114, 387, 199, 302 in the new version)<br>Thanks. We have deleted "the" in these sentences, and the same as the "third-generation sequencing".<br><br>Line 184: 'to analyze the Danio rerio …' -> to analyze the Danio rerio … genomes<br><br>Response: (line 184-185 in the new version)<br>Thanks. We have replaced "to analyze the Danio rerio…" with "to analyze the Danio rerio, Oryzias latipes, Gasterosteus aculeatus, Tetraodon nigroviridis, and Takifugu rubripes genomes".<br><br>Line 198: contains -> containing<br><br>Response: (line 198 in the new version)<br>Thanks. We have replaced "contains …" with "containing …".<br><br><br>Line 228: 'the program of DAVID program'?<br><br>Response: (line 228 in the new version)<br>Thanks. We have replaced "the program of DAVID program" with "the program DAVID".<br><br>Line 229: 'on human/chicken GO term list' -> on a/the human/chicken GO term list<br><br>Response: (line 229-230 in the new version)<br>Thanks. We have corrected the sentence.<br><br>Line 241: The details of PCR fragments distract from the general description of the method. These details are again provided in line 276, where they are more appropriate.<br><br>Response: (lines 241-243, lines 275-277 in the new version)<br>Thanks. We have deleted the description in lines 241-243, and only retain the contents in lines 275-277.<br><br>Line 244: The parentheses are superfluous.<br><br>Response: (line 243 in the new version)<br>Thanks. We have deleted the parentheses.<br><br>Line 277: covers -> covering<br><br>Response: (line 276 in the new version)<br>Thanks. We have replaced "covers" with "covering".<br><br><br>Line 300: '142 scaffolds were…' -> 'An additional 142 scaffolds were…'<br><br>Response: (line 299 in the new version)<br>Thanks. We have replaced "142 scaffolds" with "an additional 142 scaffolds".<br><br><br>Line 346: 'probability of ≥ actual numbers', I think this could be clearer if written out in full.

	Response: (line 344-346 in the new version)<br>Thanks. We have replaced “we computed the probability of ≥ actual numbers of ridges under a random permutation of the gene positions following previous method” with a complete sentence “we computed the probability of the events (ridge numbers under a random permutation of the gene positions ≥ actual numbers of ridges) following a method previously described”.<br><br>Line 347: 'following previous method' -> following a method previously described<br><br>Response: (line 346 in the new version)<br>Thanks. We have corrected this sentence.<br><br>Line 473: 'into chromosomes filled the gaps' -> into chromosomes, with each gap (see figure 3) replaced with 50 kb of Ns<br><br>Response: (line 491 in the new version)<br>Thanks. We have replaced “into chromosomes filled the gaps” with “into chromosomes, with each gap (see figure 3) replaced with 1 kb of Ns”. We also corrected the size of the gaps with 1 kb, as we have rechecked data and found the mistake in the previous writing.
**Additional Information:**	
**Question**	**Response**
Are you submitting this manuscript to a special series or article collection?	No
**Experimental design and statistics**<br><br>Full details of the experimental design and statistical methods used should be given in the Methods section, as detailed in our Minimum Standards Reporting Checklist. Information essential to interpreting the data presented should be made available in the figure legends.<br><br>Have you included all the information requested in your manuscript?	Yes
**Resources**<br><br>A description of all resources used, including antibodies, cell lines, animals and software tools, with enough information to allow them to be uniquely identified, should be included in the Methods section. Authors are strongly encouraged to cite Research Resource Identifiers (RRIDs) for antibodies, model organisms and tools, where possible.<br><br>Have you included the information requested as detailed in our Minimum Standards Reporting Checklist?	Yes

Powered by Editorial Manager® and ProduXion Manager® from Aries Systems Corporation

**Availability of data and materials**<br><br>All datasets and code on which the conclusions of the paper rely must be either included in your submission or deposited in publicly available repositories (where available and ethically appropriate), referencing such data using a unique identifier in the references and in the “Availability of Data and Materials” section of your manuscript.<br><br>Have you have met the above requirement as detailed in our Minimum Standards Reporting Checklist?	Yes

Powered by Editorial Manager® and ProduXion Manager® from Aries Systems Corporation

Manuscript Click here to download Manuscript Monopterus16_4.docx

Chromosome-scale assembly of the *Monopterus* genome

Xueya Zhao[1], Majing Luo[1], Zhigang Li[1], Pei Zhong[1], Yibin Cheng[1], Fengling Lai[1], Xin Wang[1], Jiumeng Min[2], Mingzhou Bai[2], Yulan Yang[2], Hanhua Cheng[1]*, Rongjia Zhou[1]*

[1]Hubei Key Laboratory of Cell Homeostasis, Laboratory of Molecular and Developmental Genetics, College of Life Sciences, Wuhan University, Wuhan 430072, P. R. China

[2]BGI Genomics, BGI-Shenzhen, Shenzhen 518083, P. R. China

*Corresponding authors: Professors Rongjia Zhou and Hanhua Cheng, College of Life Sciences, Wuhan University, Wuhan 430072, P. R. China, Fax: 0086-27-68756253, E-mail: rjzhou@whu.edu.cn, hhcheng@whu.edu.cn

Abstract

Background: The teleost fish *Monopterus albus* is emerging as a new model for biological studies due to its natural sex transition and small genome, in addition to its enormous economic and potential medical value. However, no genomic information for the *Monopterus* is currently available.

Findings: Here, we sequenced and de novo assembled the genome of *Monopterus albus* and report the *de novo* chromosome assembly by FISH walking assisted by conserved synteny (Cafs). Using Cafs, 328 scaffolds were assembled into 12 chromosomes, which cover genomic sequences of 555 Mb, accounting for 81.3% of the sequences assembled in scaffolds (~689 Mb). A total of 18,660 genes were mapped on the chromosomes and showed a non-random distribution along chromosomes.

Conclusions: We report the first reference genome of the *Monopterus* and provided an efficient Cafs strategy for a *de novo* chromosome-level assembly of the *Monopterus* genome，which provides a valuable resource, not only for further studies in genetics, evolution and development, particularly sex determination, but also for breed improvement of the species.

Key words: whole-genome sequencing, genome assembly, chromosomes, fish

Data Description

Background

The freshwater fish *Monopterus albus* taxonomically belongs to the teleost family Synbranchidae of the order Synbranchiformes. This fish is distributed mainly in southern and eastern Asia, in northern Australia and in the southeastern United States [1]. *Monopterus* is an economically important species because of its high nutritional value (e.g., high polyunsaturated fatty acid omega-6 levels) and potential medical value. The most influential Chinese pharmacy monograph, the Bencao Gangmu, a compendium of materia medica written by the pharmacist Shi-Zhen Li during the Ming Dynasty (AD 1368~AD 1644), recommended *Monopterus* as a natural drug with medicinal virtues.

As an emerging model species in development, genetics and evolution [2], *Monopterus* has the attractive feature of undergoing a sex transition from female to intersex to male during its life [3]. This discovery may have considerable theoretical significance in sex determination [4]. *Monopterus* has a small genome size (~800 Mb) and a minimum chromosome number (n = 12) among teleosts, whose chromosome numbers range from 12 to 223 [5]. In addition, all chromosomes of *Monopterus* are telocentric. Given that a third whole-genome duplication occurred in the whole teleost lineage compared to the two genome duplications that occurred in other land vertebrates [6-8], the speciation and sexual differentiation of *Monopterus* may provide new insights into vertebrate evolution. However, the mechanisms of sex determination in the species remain unknown.

Whole-genome sequencing will provide detailed genetic data for studies of genetics, development and evolution and for the genetic manipulation of *Monopterus*. However, no genetic map is currently available for this species. The whole-genome shotgun approach, with high throughput and low cost, is based on a second-generation sequencing platform that makes the whole-genome *de novo* assembly of a species possible without the need for a physical map. However, the sequence data produced by second-generation sequencing technologies are highly fragmented due to the short lengths of the reads. A number of methods for increasing the

contiguity and accuracy of *de novo* assemblies have recently been developed. The read length generated from sequencing can be improved by a third-generation sequencing platform, such as single-molecule real-time (SMRT) sequencing, with raw reads of a mean length of 15 kb [9], and nanopore single-molecular sequencing, with raw reads of approximately 5-50 kb[10]. Some strategies for the assembly of a long scaffold have also been developed, for example, BAC/fosmid paired end sequencing, the long-read sequencing (LRseq) [11] approach, contiguity-preserving transposase sequencing (*fragScaff*)[12], and various assembly algorithms [13, 14]. Recently, chromatin interactions, such as Hi-C, have been used to assemble ultra-long scaffolds which can lead to a chromosome-scale assembly; however, a certain amount of error occurs when used for *de novo* assembly[14, 15]. Thus, accurate chromosome-level assembly remains a major challenge.

The most widely used strategy for chromosome-level assembly of the scaffolds generated by second-generation sequencing is based on a high-density genetic map at chromosome level. Nevertheless, this strategy is feasible only when high-density genetic maps of a species are available. Because there is no genetic map available for *Monopterus*, we have developed an efficient assembly strategy: *de novo* chromosome assembly by FISH walking assisted by conserved synteny (Cafs). Using Cafs technology, which is efficient and cost effective, a precise chromosome-level assembly covering 81.3% of the sequences assembled in scaffolds was produced.

Whole genome sequencing

A whole-genome shotgun strategy and second-generation sequencing technology (Illumina HiSeq 2000 platform) were used to sequence two male *Monopterus*. Genomic DNA was extracted from eels from the Wuhan area in the Yangtze River basin. To reduce the risk of non-random sequencing, 8 paired-end sequencing libraries with insert sizes of 170 bp, 500 bp, 800 bp, 2 kb, 5 kb, 10 kb, 20 kb and 40 kb were constructed. Of them, the library with 40 kb inserts was from second individual for assembling of long scaffolds. These libraries generated 101.62 GB of sequence data. To reduce sequencing errors in the assembly, sequence reads were filtered

to remove low quality reads. After filtering, 78.64 GB (97.6X) of sequence data were retained for the assembly, which ensures a high single-base accuracy (Additional file: Figure S1 and Table S1).

Estimation of genome size

A k-mer was defined as a sequence of k bases in length. The frequency of k-mers in a collection of short, insert-sized reads could be calculated with a 1 bp sliding window. When an optimal amount of data was present, the k-mer frequency followed a Poisson distribution. The k-mer value was used to estimate the genome size, as follows: Genome Size = K_num/Peak_depth, where K_num is the total number of k-mers, and Peak_depth is the expected value of the k-mer depth [16]. The 17-mer distribution obeyed the theoretical Poisson distribution. The data used for k-mer analysis were derived from the male which was used to construct the genome sequencing libraries. The heterozygosity revealed from the k-mer analysis reflects the inner heterozygosity in an individual. Finally, we observed that the proportion of heterozygosity in the *Monopterus* genome was small, and estimated that the entire genome comprised 806 Mb, with a GC content of 40.8% (Additional file: Figures S2-S3 and Table S2).

***De novo* genome assembly**

The *Monopterus* genome was *de novo* assembled with the SOAPdenovo software [16] (SOAP, RRID:SCR_000689). SOAPdenovo employs the de Bruijn graph algorithm to simplify assembly and reduce the computational complexity. Low quality reads were filtered out and potential sequencing errors were removed or corrected with the k-mer frequency methodology. The SOAPdenovo assembly process consisted of three main steps: contig construction, scaffold construction, and gap filling. The sequencing data derived from the libraries with insert size of 2 kb, 5 kb, 10 kb and 20 kb was used to assemble the scaffolds by SOAPdenovo. The sequencing data derived from the library with 40 kb insert were used to build scaffolds with SSPACE version 1.1 software (SPACE, RRID:SCR_005056) [17].

To assess assembly quality and completeness, high quality reads from short-insert-size libraries

(75 bp read lengths) were aligned to the assembly with the BWA (BWA, RRID:SCR_010910) program [18] (version 0.5.9-r16), with default parameters. Next, SOAPcoverage (version 2.27) was used to calculate sequencing depth. A total of 91.06 % reads could be mapped, and they covered 99.69 % of the assembly, excluding gaps. To further test for possible contigs that might be mis-joined in scaffolds, we analyzed paired-end information. We found that, if contigs were included only when both ends could be uniquely mapped onto the assembly, 90.66 % of paired-ends were in the correct orientation and at the expected distance, according to the utilized short-insert-size libraries (Additional file: Tables S3).

The final assembly comprised 689.5 Mb with contig and scaffold N50 sizes of 22.2 kb and 2.1 Mb, respectively (Table 1). Over 90% of the total sequence was covered by 379 scaffolds; the longest scaffold spanned 11.7 Mb (Table 1). Assembly accuracy was further demonstrated by 91.06 % reads mapping (99.69% coverage) to the reference sequences of the genome and the successful mapping of 321 bacterial artificial chromosomes (BACs), sequenced with Sanger sequencing technology.

To evaluate the quality of the assembled genome, we conducted Benchmarking Universal Single-Copy Orthologs (BUSCO, RRID:SCR_015008) analysis [19] using BUSCO v2.0 with vertebrata_odb9 including 2,586 BUSCOs. Using the BUSCO analysis, 96.5% of BUSCOs were completely detected in the assembled genome (2,464: complete and single-copy, 32: complete and duplicated) among 2,586 tested BUSCOs. The number of fragmented and missing BUSCOs was 56 and 34, respectively. Together, the genome of the *Monopterus* assembled is of high quality.

Repeat elements

Transposable elements (TEs) were identified in the genome with combination of homology-based and de novo approaches. The homology-based approach utilized database Repbase [20] (release 19.06), with RepeatMasker (RepeatMasker, RRID:SCR_012954), version 4.0.3, and RepeatProteinMask (from the RepeatMasker package) programs with the default parameters [20]. The de novo approach used two prediction programs, RepeatModeler (RepeatModeler,

RRID:SCR_015027) [21], version 1.0.7, and LTR-FINDER [22] (version 1.0.5), to build the de novo repeat libraries based on the genome sequences. Next, contaminations and multi-copy genes were removed from the libraries. Then, the RepeatMasker was used again to find repeats in these repetitive sequence libraries. Finally, we combined all the results generated by these methods. To improve our comparisons to other teleost fishes, we employed the same procedure and parameters to analyze the *Danio rerio, Oryzias latipes*, *Gasterosteus aculeatus*, *Tetraodon nigroviridis,* and *Takifugu rubripes* genomes.

For the assembled sequence, the repetitive element content of the *Monopterus* genome (28%) was much lower than that of the zebrafish (61%) and about the same as medaka (29%) genomes, but higher than that of the threespine stickleback (16%) and pufferfish (8-10%) genomes (Additional file: Figure S4). In the *Monopterus* genome, the main repetitive transposable elements were the DNA transposons and LINEs. At 8%, the LINEs were the largest category of transposable elements. The percent of LINEs was greater than that found in other teleost fish (2-5%), which might be associated with genome instability[23].

It should be pointed out that， only ~79% of the expected genome size was captured in contigs (634.7 Mb of 806 Mb), and the BUSCO analysis showed that the 634.7 Mb genome assembly was complete. These data suggest that the unassembled genome probably consists of non-coding DNA, possibly containing many repeats. Thus, the repeat abundance would likely be underestimated. Third-generation sequencing will provide a more complete assembly.

Genes and function annotation

We used both homology-based and de novo methods to predict genes in the *Monopterus* genome by scanning the local *Monopterus* genome database, which also included RNA-seq data. For the homology-based prediction, protein sequences from *D. rerio*, *O. latipes*, *G. aculeatus*, *T. nigroviridis*, and *T. rubripes* were downloaded from the Ensemble platform[24] (release 75) and aligned with the *Monopterus* genome with the Tblastn program[25]. Accordingly, homologous genomic sequences were input into the Genewise program[26] to align matching proteins. This procedure allowed us to define gene structures. For de novo

prediction, both the Fgenesh[27] and Genscan[28] programs were employed to predict coding genes, with the appropriate parameters. Homology-based and de novo derived gene sets were combined with comprehensive, non-redundant reference gene sets, obtained with the GLEAN platform (http://sourceforge.net/projects/glean-gene/). Genes were corrected by comparisons with the RNA-seq data; these RNA-seqs were mapped to the *Monopterus* genome with the Tophat program, and the Cufflinks (Cufflinks, RRID:SCR_014597) program (version cufflinks-2.2.1) [29] was used to assemble transcripts. After that, we selected 1000 intact genes, defined as gene set “A”, which were supported by the homology-based prediction, and passing a fifth-order Markov model, to verify the ORFs of RNA transcripts based on the Hidden Markov Model (HMM). In the *Monopterus*, a total of 24,056 protein-coding genes were predicted (Additional file: Table S4-S6). The average gene sizes were similar to those of other teleost fish (Additional file: Figure S5 and Table S5).

Blastp was used to search for proteins encoded in the *Monopterus* genome by comparing candidate sequences against the SwissProt and TrEMBL databases from UniProt Knowledgebase (UniProtKB) [30]. The annotated motifs and domains in the available databases (ProDom, PRINTS, Pfam, SMART, PANTHER, and PROSITE) were obtained with the InterProScan (InterProScan, RRID:SCR_005829) program [31] (version 4.7). In gene ontology (GO) [32] analyses, gene functions were obtained from the corresponding InterPro entries. Subsets of the GO terms were obtained according to the program DAVID (version 6.7) [33]. X-associated genes were annotated based on the GO term list in human and Z-associated genes were annotated based on the GO term list in chicken. All genes were also aligned against the KEGG [34] (release 68) protein database. The genes that matched genes in the KEGG database were assumed to be involved in the corresponding signaling pathways. Approximately 80% of the genes could be functionally annotated with homology analysis (Additional file: Figure S6).

De novo chromosome assembly by Cafs-strategy

To assemble chromosomes with accurate sequences from the scaffolds, we developed an

efficient assembly strategy without using any genetic map information, Cafs (Figure 1), which is based on chromosome fluorescent *in situ* hybridization (FISH) and the shared synteny between distantly related fish species.

We first prepared probes of BACs from sequenced clones and PCR fragment pools representing scaffolds for chromosome FISH, and performed synteny analysis of these scaffolds by comparing with the fish species medaka, sticklebacks, *Tetraodon* and *Monopterus*. Second, from the synteny information of the homologous sequences of these scaffolds in the three fish species， probe combination mapping was used to determine 12 linkage groups, corresponding to 12 chromosomes, each with a molecular landmark (Figure 1A). Briefly, group A and B were first discriminated by two unlinked scaffolds labelled with two different colours. If another scaffold was unlinked to the previous two scaffolds, the third scaffold was identified as a marker of group C. Third, based on the predicted syntenic relationship between the related fish species, probes for the candidate scaffolds were co-hybridized with the landmarks of the chromosomes, which have been identified. Scaffolds with no predicted location and that were inconsistent with the predicted location were further determined by co-hybridization with 12 landmarks using dual-colour FISH respectively (Figure 1B). For example, scaffold 58 would be grouped into the F group, as it is linked with scaffold 129, which was the landmark of the F group. Fourth, the loose and long pachytene chromosomes were adopted to determine the location and order relationship of the scaffolds through dual- and three-colour FISH. An original marker was used as a walking start (e.g., scaffold 73), and the location of the second scaffold (e.g., scaffold 186) relative to the original marker was identified by dual-colour FISH. The location of a new scaffold was determined by the known scaffold locations using dual- or three-colour FISH (e.g., scaffolds 72, 123, and 4) (Figure 1C). Finally, because all 12 chromosomes are telocentric, the telomeres of the metaphase chromosome were used as landmarks to determine the directions of the mapped scaffolds on the chromosomes (Figure 1D). The relative position of all scaffolds on chromosomes was determined by the measurement of the signals to the centromere (Figure 1E). The distance values were measured by Image-Pro Plus 6.0, and each value was obtained from an average of more than five cells.

Using the Cafs assembly strategy, we conducted large-scale mapping of the scaffolds on each chromosome. Metaphase chromosomes were prepared according to routine protocols from the *Monopterus* kidney tissue [5]. Meiotic pachytene bivalents were prepared from *Monopterus* testis using a previously described method [35]. The FISH was conducted as previously described [36]. The BAC end sequences were aligned to the genome database by Blat (version blat_34)[37]. BACs with two ends aligned to one scaffold, and those ends with sequences with homology to scaffolds greater than 90% were used as probes for FISH. Of the ~747 sequenced clones, 148 BACs could be used as probes for FISH (Table S7). The BACs were confirmed by PCR sequencing from the internal regions of the BACs. 148 BACs and 38 pools of PCR fragments (8-15 sequences covering a total length of 20-30 kb on a scaffold) representing 186 scaffolds (Table S8) were prepared as probes for chromosome FISH.

Before the hybridization experiment, we performed a genome-wide synteny analysis to compare these fish species. We constructed a reference map using the syntenic relationship among the genomes of medaka, stickleback and *Tetraodon* to help map the scaffolds on the *Monopterus* chromosomes. The syntenic blocks between *Monopterus* and other fishes were aligned by Lastz (Blastz) [38] with parameters of T=2 and Y=3400. Furthermore, we used Blat to search for homologous sequences among medaka, sticklebacks, *Tetraodon* and *Monopterus* in order to fill the gap sequences of blocks in the reference map. If two homologous sequences were linked in all three species, we defined the corresponding scaffolds in *Monopterus* as predicted linked scaffolds.

Under the guidance of the synteny of the homologous sequence of these 186 scaffolds in other three fish species, 78 probes combinations of co-hybridization were performed to identify 12 linkage groups, each with a molecular landmark (Figure 2A). We then conducted the walking in a range of 11-22 steps per chromosome (Figure 2B; Additional file: Figure S7). A total of 186 scaffolds were assembled into 12 pachytene chromosomes through step-by-step combination hybridization of the probes using the above-mentioned 148 BACs and 38 PCR fragment pools (Figure 3A). We then determined the orientation of each chromosome by dual-colour FISH on metaphase chromosomes using the telomere as a morphological landmark

(Additional file: Figure S8). Of these mapped scaffolds, 92% (99/108) were consistent with the shared synteny between the related fish species (medaka, sticklebacks and *Tetraodon*). From the synteny analysis, an additional 142 scaffolds were predicted and further assembled into 12 chromosomes respectively. Based on the results of the FISH experiments and collinearity analyses, we can expect approximately 8% placement errors amongst these 142 scaffolds. Accurate mapping of the 142 scaffolds remains to be confirmed by third-generation sequencing. The current assembly, particularly the locations of the 186 scaffolds, have great implications for comparative genomics and evolution studies. As the genome of *Monopterus* has the least number of chromosomes in the teleosts, the determination of 12 linkage groups of the *Monopterus* genome is crucial for the studies of large-scale chromosome recombination events (e.g. chromosome fusion or fission) in the teleost evolution.

We finally integrated 328 scaffolds into the reference genome. These mapped scaffolds consisted of 455 Mb determined by FISH and 100 Mb determined by syntenic analysis, which covers genomic sequences of a total length of 555 Mb, accounting for 81.3% of the sequences assembled in scaffolds (689.5 Mb). Based on the assembly, a total of 18,660 protein-coding genes were annotated with location information on the chromosomes (Table 2). For example, there are 87 protein-coding genes on scaffold 72, which was located on chromosome 5 (Figure 3B). These data indicate that a *de novo* chromosome-level assembly of the *Monopterus* genome was produced using the Cafs strategy.

Chromosome-wide gene clustering

To further investigate gene clustering along the chromosomes, we calculated the gene density per chromosome. The average gene density in the genome was 33.6 genes per Mb, with the maximum gene density on chromosome 12, which is the shortest chromosome, and the minimum gene density on chromosome 9 (Table 2). Further sliding window analysis showed that there was also biased distribution of the gene density within the chromosome (Figure 4A). Using a 1-Mb window size and 100-kb step size, the maximum gene density in the genome was detected from nt 22,200,001 to nt 23,200,000 on chromosome 10, which contains 71 genes, in comparison with an average of 33.6 genes per Mb in the genome (Figure 4B).

We then tested the statistical significance of pairing correlations between gene density and GC content. As these parameters are not normally distributed, we used the non-parametric Spearman correlation test on the ranks of the paired quantities. Correlation analyses were performed with R software (www.r-project.org). The R package ggplot2 was used to draw scatterplots and boxplots. The distribution pattern of the gene density was consistent with the corresponding GC content along the chromosomes (Figure 4A; Additional file: Figure S9A). In order to see whether particular regions contribute to the positive correlation, we used the sliding window analysis to divide the genome into 4 Mb small regions and calculated the correlation coefficient between GC content and gene density in each 4 Mb-region. Using a 3-Mb window size and 100-kb step size, we divided the genomes into 5196 regions with each size of 4 Mb. The analysis showed that there are a large proportion (68.21%) of 4 Mb regions with lower correlations than 0.7 (Additional file: Figure S9B). Some regions with very high correlation coefficients ($R > 0.95$, p-value <0.01) are detected in the genome, for example, four regions on chromosome 12 (Figure 4A). In this analysis, the Circos (version 0.69)[39] is used to plot the assembled chromosomes, GC content and gene density.

To investigate whether the distribution of the genes along the chromosomes is non-random, we computed the probability of the events (ridge numbers under a random permutation of the gene positions ≥ actual numbers of ridges) following a method previously described [40]. A ridge was used to describe a chromosome region with high gene density, which is thus defined as at least W consecutive windows, each containing a gene number higher than H. Thus, the ridge is determined by two parameters: cutoff 1 (C_H), gene number per window, and cutoff 2 (C_W), number of consecutive windows. The actual ridge numbers (N) in the genome were calculated under C_H and C_W by sliding window analysis. We used the following calculation parameters to set up a null model: suppose we have a random permutation of $X_1, X_2, ..., X_i$ in the range of 1 to S; i, gene number on the chromosome; S, length of the chromosome; and $X_1, X_2, ..., X_i$, gene locations on the chromosome. With the same cutoff values under actual conditions (C_H and C_W), we can obtain a ridge number (n) under the null model. We can compute the frequency (f) when $n \geq N$ by permutation 10,000 times. If $f = 0$, the p-value $< 10^{-4}$, or the p-value $= f/10000$. For

all of the cutoff C_H and C_W combinations, we calculated the *p*-value under different window sizes of 0.2, 0.3, 0.5, 1, 2, and 3 Mb respectively.

Using a combination of the two cutoffs, the numbers of the ridges of each chromosome can be identified. For example, using cutoffs of 40 genes per Mb and 5 consecutive windows, 7 ridges on chromosome 10 were identified (Figure 4B), and 90 ridges were identified in the genome (Figure 4C). The probability of the observed ridges occurring in random permutations of gene positions was very low (p-value < 10^{-4}) (Figure 4C), confirming non-random and clustering distribution of genes along the chromosomes. Probabilities (ridges numbers under a random permutation ≥ ridges numbers in the *Monopterus* genome) for a series of cutoff sets and different window sizes were also calculated and the results showed that there were significant differences in ridges numbers between the *Monopterus* genome and random permutations of gene positions (Figure 4C; Additional file: Figure S10). The ridge numbers of high gene density directly reflect the clustering of genes along the chromosomes. These analyses suggest that the ridge pattern on the chromosomes probably represents a higher-order structure in the genome.

Re-use potential

In summary, here we report the first genome to be sequenced and assembled in the order Synbranchiformes in fresh-water fishes. Because *Monopterus* is not only an economically important freshwater fish in aquacultural production, but also an increasingly known model species for biological studies, the assembled genome will provide valuable information for (i) genetic improvement of economical traits by hunting key genes/QTLs in the species, (ii) understanding molecular mechanisms underlying sex reversal, (iii) genome evolution studies by comparative genomics among *Monopterus*, other fishes and the species in land, (iv) speciation and ecological conservation researches by dissecting aerial respiration ability of the species, which is a crucial feature in the origin of the tetrapods from the sea up to the land. We also expect that the genome data will be used by other researchers for functional genomics, for example, gene knockout using genes provided here through CRISPR/Cas9 approach, and also making transgenic fish using the genes and assembly represented in this study. In addition, the assembled genome provides a reference for further complete and accurate assembly/annotation

as gaps/assembly errors exist in the version of assembly using second-generation sequencing technology.

Data availability

The genome data from this study have been deposited at DDBJ/EMBL/GenBank under accession number AONE00000000, and the raw transcriptome data have been submitted to NCBI Gene Expression Omnibus (GEO) under accession number GSE43649. Datasets further supporting the manuscript, including BUSCO results, annotations and perl scripts, are available in the *GigaScience* database, GigaDB[41].

Additional files

Supplemental Figure S1. Sequencing depth distribution of the *Monopterus* genome.

Supplemental Figure S2. Genome size estimation using 17-mer.

Supplemental Figure S3. The GC distribution of the *Monopterus* genome.

Supplemental Figure S4. Divergence distribution of the classified TE elements.

Supplemental Figure S5. Comparisons of predicted coding genes of *Monopterus* with other teleost fishes.

Supplemental Figure S6. The Gene Ontology of the *Monopterus* genes.

Supplemental Figure S7. Localization of each scaffold on chromosomes by FISH-walking strategy.

Supplemental Figure S8. Orientation of each linkage group on metaphase chromosomes.

Supplemental Figure S9. Correlation coefficient of GC content with gene density.

Supplemental Figure S10. Statistical tests of numbers of gene density ridges in the genome corresponding to background noise (null model) in different window sizes (0.2, 0.3, 0.5, 1, 2, 3 Mb).

Supplemental Table S1. Statistics of sequencing.

Supplemental Table S2. Statistics of genome from 17-mer.

Supplemental Table S3. Statistics of mapping.

Supplemental Table S4. Statistics of predicted coding genes.

Supplemental Table S5. Comparisons of predicted coding genes of *Monopterus* with other

teleost fishes.

Supplemental Table S6. Annotated classification of the *Monopterus* genes.

Supplemental Table S7. Alignments of BAC ends to reference genome.

Supplemental Table S8. Information of FISH probes synthesized by PCR.

List of Abbreviations

BAC: bacterial artificial chromosome; bp: base pair; BUSCO: Benchmarking Universal Single-Copy Orthologs; Cafs: chromosome assembly by FISH walking assisted by conserved synteny; DAPI :49-6-diamidino-2-phenylindole; FISH: fluorescent *in situ* hybridization; Gb: giga base; GO: gene ontology; H&E: haematoxylin and eosin; Hi-C: high-throughput/resolution chromosome conformation capture; kb: kilo base; KEGG: Kyoto Encyclopedia of Genes and Genomes; LINEs: long interspersed nuclear elements; LRseq: long-read sequencing; Mb: mega base; PAC: plant artificial chromosome; piRNAs: Piwi-interacting small RNAs; SMRT : single-molecule real-time; TEs: Transposable elements; CRISPR: clustered regularly interspaced short palindromic repeats; Cas9: CRISPR associated protein 9

Acknowledgements

This work was supported by the National Natural Science Foundation of China, National Key Technologies R&D Program and Hubei Province Science and Technology Project.

Authors' contributions

Conceptualization: Rongjia Zhou.

Funding acquisition: Rongjia Zhou, Hanhua Cheng.

Investigation: Xueya Zhao, Majing Luo, Zhigang Li, Yibin Cheng, Fengling Lai, Xin Wang, Jiumeng Min, Mingzhou Bai, Yulan Yang.

Methodology: Xueya Zhao, Majing Luo, Zhigang Li, Pei Zhong.

Supervision: Rongjia Zhou, Hanhua Cheng.

Validation: Xueya Zhao, Rongjia Zhou.

Writing – original draft: Xueya Zhao, Rongjia Zhou.

Writing – review & editing: Xueya Zhao, Rongjia Zhou.

Ethics statement

Monopterus were obtained from Hubei, China. All animal experiments and methods were performed in accordance with the relevant approved guidelines and regulations, as well as under the approval of the Ethics Committee of Wuhan University.

Competing interests

The authors declare that they have no competing interests

Figure legends

Figure 1 Overview of *de novo* chromosome assembly by FISH walking assisted by conserved synteny. A: Identification of 12 linkage groups by probe combination mapping. FISH probes are hybridized on pachytene chromosomes. Red and green dots indicate scaffold locations. B: Synteny-assisted scaffold mapping. Each candidate BAC (scaffold) is co-hybridized with 12 landmarks by dual-colour FISH respectively. Synteny-supported/non-supported scaffolds are determined by FISH. C: Determination of scaffold order on chromosome by FISH walking. (I) The order of two scaffolds is identified by dual-colour FISH if both of them are on one side of the chromosome. (II) If the scaffolds are in the centre of the chromosome, three-colour FISH is applied to determine their order. (III) The order of some scaffolds (labelled with one colour) could be identified by three signals dual-colour FISH, when their two neighbouring scaffolds (labelled with another colour) have been determined. D: Identification of orientation of linkage groups on metaphase chromosomes. Telomeres and centromeres can be observed on the metaphase chromosomes. E: Localization of scaffolds is determined by calculating the corresponding distances to the centromere.

Figure 2 Chromosome assembly by Cafs. A: FISH images show 12 molecular landmarks corresponding to 12 chromosomes. Green signals indicate the landmarks labelled by digoxigenin and detected with FITC. Each chromosome is determined by a landmark. Chromosomes are stained by DAPI (blue). B: Localization of each scaffold on chromosome 5

by FISH walking strategy. FISH images and corresponding scaffold order from (a') to (m') are shown in the left panels. A three-colour FISH image (g') in the upper right indicates the relative order of scaffolds 4 (yellow, FITC+Cy3), 30 (green, FITC) and 99 (red, Cy3) on chromosome 5. Probes (red dots) and their locations on scaffolds are used to assemble chromosome 5.

Figure 3 Chromosome-scale assembly of the *Monopterus* genome. A: Each chromosome is assembled with scaffolds and their order from telomere (down end) to centromere (up end). The grey and purple cylinders represent the anchored scaffolds. The segments in light blue between two neighbouring scaffolds indicate gaps. Sticks with a red head anchored on each scaffold indicate the positions of the BACs used as probes. Scale bar, 0-1. B: Scaffold 72 with 87 genes (blue bars) and their location on chromosome 5 is highlighted.

Figure 4 Chromosome-wide gene clustering. A: Circos shows the assembled chromosomes, GC content and gene density. The inner scale is 2 Mb. The strips in the outer circle indicate the scaffolds packaged into chromosomes, with each gap (see figure 3) replaced with 1 kb of Ns. The inner dark grey ridges show the moving GC percentage, and the inner grey ridges show the moving number of the genes at a window size of 1 Mb. The green sticks indicate the location of the regions with high correlation between GC content with gene density ($R > 0.95$, p-value < 0.01). B: Distribution of gene clusters (ridges) on chromosome 10. Curves indicate the moving numbers of genes at a window size of 1 Mb (step = 100 kb). The windows with a maximum gene density from nt 22200001 to 23200000 on chromosome 10, which contains 71 genes, are shown in the lower panel. Green boxes highlight ridges in which there are at least 5 consecutive moving windows with a lower limit of 40 genes per window. C: Statistical tests of numbers of gene density ridges in the genome corresponding to background noise (null model). The heat map in the lower panel shows p-values in the significance test of observed ridge numbers against the null model (10,000 independent permutations of gene positions). The x-axis indicates the cutoff values of numbers of consecutive moving windows, which reflects the extent of the clustering. The y-axis indicates the cutoff values of gene numbers within a certain window size (step 100 kb), which reflects the degree of intensity of the clusters. Green lines represent the average gene number in a certain window size. The upper panel highlights a

significance test at the condition of two cutoff values, gene density (40/Mb) and consecutive window numbers (5). Red dots represent the number of observed ridges in the genome. Boxplots (black) represent distribution of the ridge numbers in 10,000 independent permutations of gene positions in a random fashion.

References

1. Collins TM, Trexler JC, Nico LG, Rawlings TA. Genetic Diversity in a Morphologically Conservative Invasive Taxon: Multiple Introductions of Swamp Eels to the Southeastern United States. Conserv Biol. 2002; 16:1024-35.
2. Cheng HH, Guo YQ, Yu QX, Zhou RJ. The rice field eel as a model system for vertebrate sexual development. Cytogenet Genome Res. 2003; 101:274-7.
3. Liu CK. Rudimentary hermaphroditism in the symbranchoid eel, Monopterus javanensis. Sinensia. 1944; 15:1-8.
4. Bullough WS. Hermaphroditism in the lower vertebrates. Nature. 1947; 160:9-11.
5. Yu XJ, Zhou T, Li YC, Li K, Zhou M. Chromosomes of Chinese fresh-water fishes. Beijing Science Press. 1989:1-148.
6. Zhou RJ, Cheng HH, Tiersch TR. Differential genome duplication and fish diversity. Reviews in Fish Biology and Fisheries. 2002; 11:331-7.
7. Christoffels A, Koh EG, Chia JM, Brenner S, Aparicio S, Venkatesh B. Fugu genome analysis provides evidence for a whole-genome duplication early during the evolution of ray-finned fishes. Mol Biol Evol. 2004; 21:1146-51.
8. Jaillon O, Aury JM, Brunet F, Petit JL, Stange-Thomann N, Mauceli E, et al. Genome duplication in the teleost fish Tetraodon nigroviridis reveals the early vertebrate proto-karyotype. Nature. 2004; 431:946-57.
9. Huddleston J, Ranade S, Malig M, Antonacci F, Chaisson M, Hon L, et al. Reconstructing complex regions of genomes using long-read sequencing technology. Genome Res. 2014; 24:688-96.
10. Goodwin S, Gurtowski J, Ethe-Sayers S, Deshpande P, Schatz MC, McCombie WR. Oxford Nanopore sequencing, hybrid error correction, and de novo assembly of a eukaryotic genome. Genome Res. 2015; 25:1750-6.
11. Voskoboynik A, Neff NF, Sahoo D, Newman AM, Pushkarev D, Koh W, et al. The genome sequence of the colonial chordate, Botryllus schlosseri. Elife. 2013; 2:e00569.
12. Adey A, Kitzman JO, Burton JN, Daza R, Kumar A, Christiansen L, et al. In vitro, long-range sequence information for de novo genome assembly via transposase contiguity. Genome Res. 2014; 24:2041-9.
13. Kim J, Larkin DM, Cai Q, Asan, Zhang Y, Ge RL, et al. Reference-assisted chromosome assembly. Proc Natl Acad Sci U S A. 2013; 110:1785-90.
14. Putnam NH, O'Connell BL, Stites JC, Rice BJ, Blanchette M, Calef R, et al. Chromosome-scale shotgun assembly using an in vitro method for long-range linkage. Genome Res. 2016; 26:345-50.
15. Burton JN, Adey A, Patwardhan RP, Qiu R, Kitzman JO, Shendure J. Chromosome-scale

scaffolding of de novo genome assemblies based on chromatin interactions. Nat Biotechnol. 2013; 31:1119-25.
16. Li RQ, Fan W, Tian G, Zhu HM, He L, Cai J, et al. The sequence and de novo assembly of the giant panda genome. Nature. 2010; 463:311-7.
17. Boetzer M, Henkel CV, Jansen HJ, Butler D, Pirovano W. Scaffolding pre-assembled contigs using SSPACE. Bioinformatics. 2011; 27:578-9.
18. Li H, Durbin R. Fast and accurate short read alignment with Burrows-Wheeler transform. Bioinformatics. 2009; 25:1754-60.
19. Simao FA, Waterhouse RM, Ioannidis P, Kriventseva EV, Zdobnov EM. BUSCO: assessing genome assembly and annotation completeness with single-copy orthologs. Bioinformatics. 2015; 31:3210-2.
20. Jurka J, Kapitonov VV, Pavlicek A, Klonowski P, Kohany O, Walichiewicz J. Repbase Update, a database of eukaryotic repetitive elements. Cytogenet Genome Res. 2005; 110:462-7.
21. Price AL, Jones NC, Pevzner PA. De novo identification of repeat families in large genomes. Bioinformatics. 2005; 21:i351-i8.
22. Xu Z, Wang H. LTR_FINDER: an efficient tool for the prediction of full-length LTR retrotransposons. Nucleic Acids Res. 2007; 35:W265-8.
23. Reuter M, Berninger P, Chuma S, Shah H, Hosokawa M, Funaya C, et al. Miwi catalysis is required for piRNA amplification-independent LINE1 transposon silencing. Nature. 2011; 480:264-7.
24. Flicek P, Ahmed I, Amode MR, Barrell D, Beal K, Brent S, et al. Ensembl 2013. Nucleic Acids Res. 2013; 41:D48-55.
25. Mount DW. Using the Basic Local Alignment Search Tool (BLAST). CSH Protoc. 2007; 2007:pdb top17.
26. Birney E, Clamp M, Durbin R. GeneWise and genomewise. Genome Res. 2004; 14:988-95.
27. Burge C, Karlin S. Prediction of complete gene structures in human genomic DNA. J Mol Biol. 1997; 268:78-94.
28. Salamov AA, Solovyev VV. Ab initio gene finding in Drosophila genomic DNA. Genome Res. 2000; 10:516-22.
29. Trapnell C, Roberts A, Goff L, Pertea G, Kim D, Kelley DR, et al. Differential gene and transcript expression analysis of RNA-seq experiments with TopHat and Cufflinks. Nat Protoc. 2012; 7:562-78.
30. Bairoch A, Apweiler R. The SWISS-PROT protein sequence database and its supplement TrEMBL in 2000. Nucleic Acids Res. 2000; 28:45-8.
31. Mulder N, Apweiler R. InterPro and InterProScan: tools for protein sequence classification and comparison. Methods Mol Biol. 2007; 396:59-70.
32. Ashburner M, Ball CA, Blake JA, Botstein D, Butler H, Cherry JM, et al. Gene Ontology: tool for the unification of biology. Nat Genet. 2000; 25:25-9.
33. Huang da W, Sherman BT, Lempicki RA. Systematic and integrative analysis of large gene lists using DAVID bioinformatics resources. Nat Protoc. 2009; 4:44-57.
34. Kanehisa M, Goto S. KEGG: kyoto encyclopedia of genes and genomes. Nucleic Acids Res. 2000; 28:27-30.
35. Yu QX, Fan LC, Cui JX, Ren XH, Li K, Yu XJ. High resolution G-binding and idiogram on pachytene bivalents of rice field eels. Sci China (B). 1994:1090.

36. Henegariu O, Dunai J, Chen XN, Korenberg JR, Ward DC, Greally JM. A triple color FISH technique for mouse chromosome identification. Mamm Genome. 2001; 12:462-5.
37. Kent WJ. BLAT--the BLAST-like alignment tool. Genome Res. 2002; 12:656-64.
38. Schwartz S, Kent WJ, Smit A, Zhang Z, Baertsch R, Hardison RC, et al. Human-mouse alignments with BLASTZ. Genome Res. 2003; 13:103-7.
39. Krzywinski M, Schein J, Birol I, Connors J, Gascoyne R, Horsman D, et al. Circos: an information aesthetic for comparative genomics. Genome Res. 2009; 19:1639-45.
40. Caron H, van Schaik B, van der Mee M, Baas F, Riggins G, van Sluis P, et al. The human transcriptome map: clustering of highly expressed genes in chromosomal domains. Science. 2001; 291:1289-92.
41. Zhao XY, Luo MJ, Li ZG, Zhong P, Cheng YB, Lai FL, et al. The Monopterus genome. GigaScience database 2018. http://dx.doi.org/10.5524/100435

Table 1 Click here to download Table Table 1.docx

Table 1. Statistics of the assembly of the *Monopterus* genome.

	Contigs*		Scaffolds	
	**Size (bp)**	**Number**	**Size (bp)**	**Number**
N90	4,762	33,115	368,242	379
N80	8,655	23,414	775,515	254
N70	12,290	17,275	1,109,624	180
N60	16,188	12,785	1,519,751	128
N50	22,239	8,438	2,106,322	87
Longest	159,913	----	11,676,616	----
Total size	634,655,961	----	689,524,511	----
Total number(>=100bp)	----	117,579	----	62,978
Total number (>=2kb)	----	44,314	----	2,360

*The contig size was the final size after filling intra-scaffold gaps. Contigs with lengths shorter than 100bp were not included in the statistics.

Table 2 Click here to download Table Table 2.docx

Table 2. Assembly statistics for each chromosome

Chromosome	Chromosome size (Kb)	Scaffolds No.	Gene No.	Gene density (n/10Mb)
**1**	75908.7	33	2264	298
**2**	65103.9	32	2133	328
**3**	51637.3	21	1872	363
**4**	51162.1	30	1791	350
**5**	50080.0	27	1517	303
**6**	48093.1	27	1659	345
**7**	42410.1	29	1500	354
**8**	41999.7	30	1456	347
**9**	41928.7	23	1241	296
**10**	34690.8	30	1262	364
**11**	29285.5	23	1086	371
**12**	22774.4	23	879	386
**Total**	555074.3	328	18660	336

Figure 1

Click here to download Figure Figure 1.tif

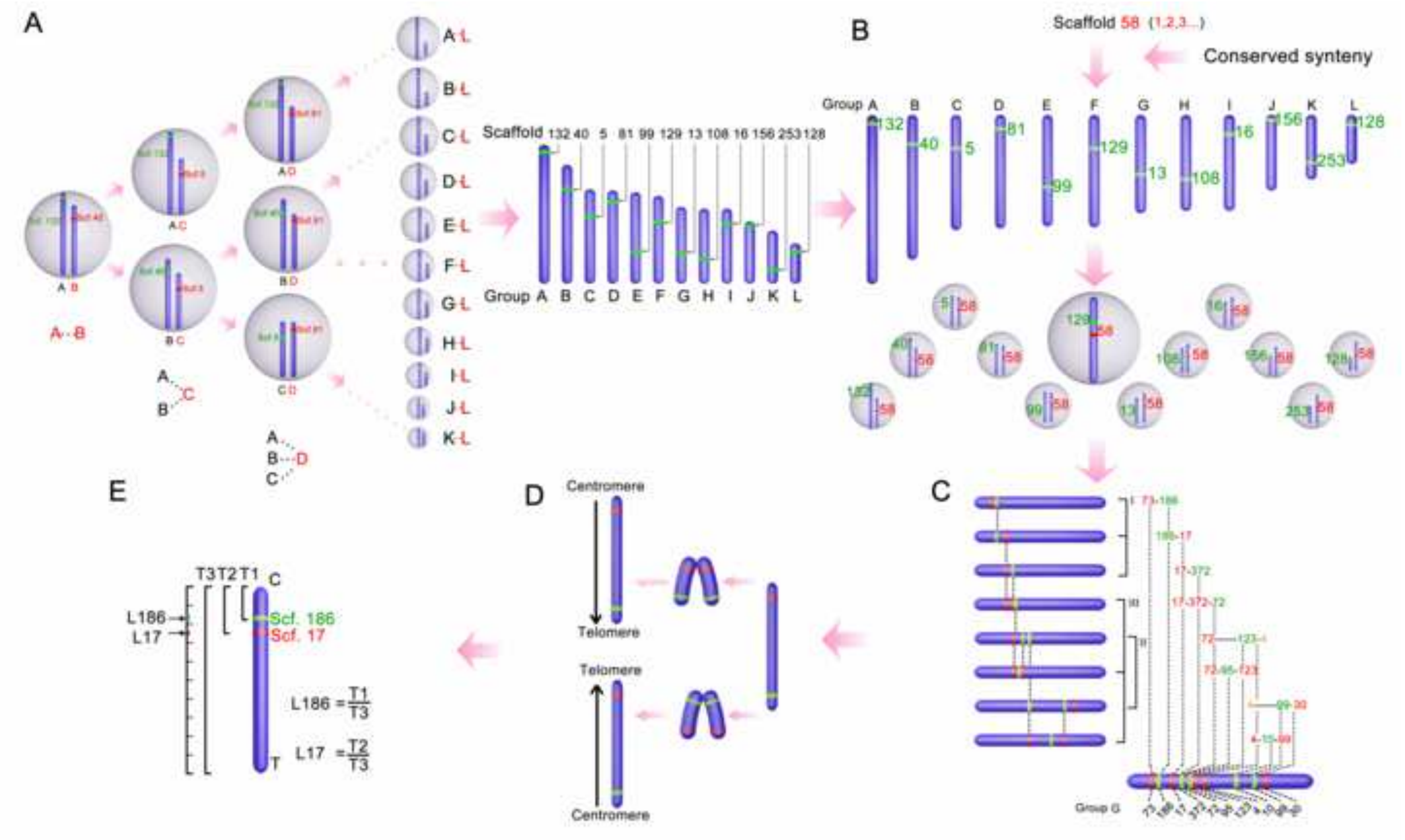

Figure 2

Click here to download Figure Figure 2 .tif

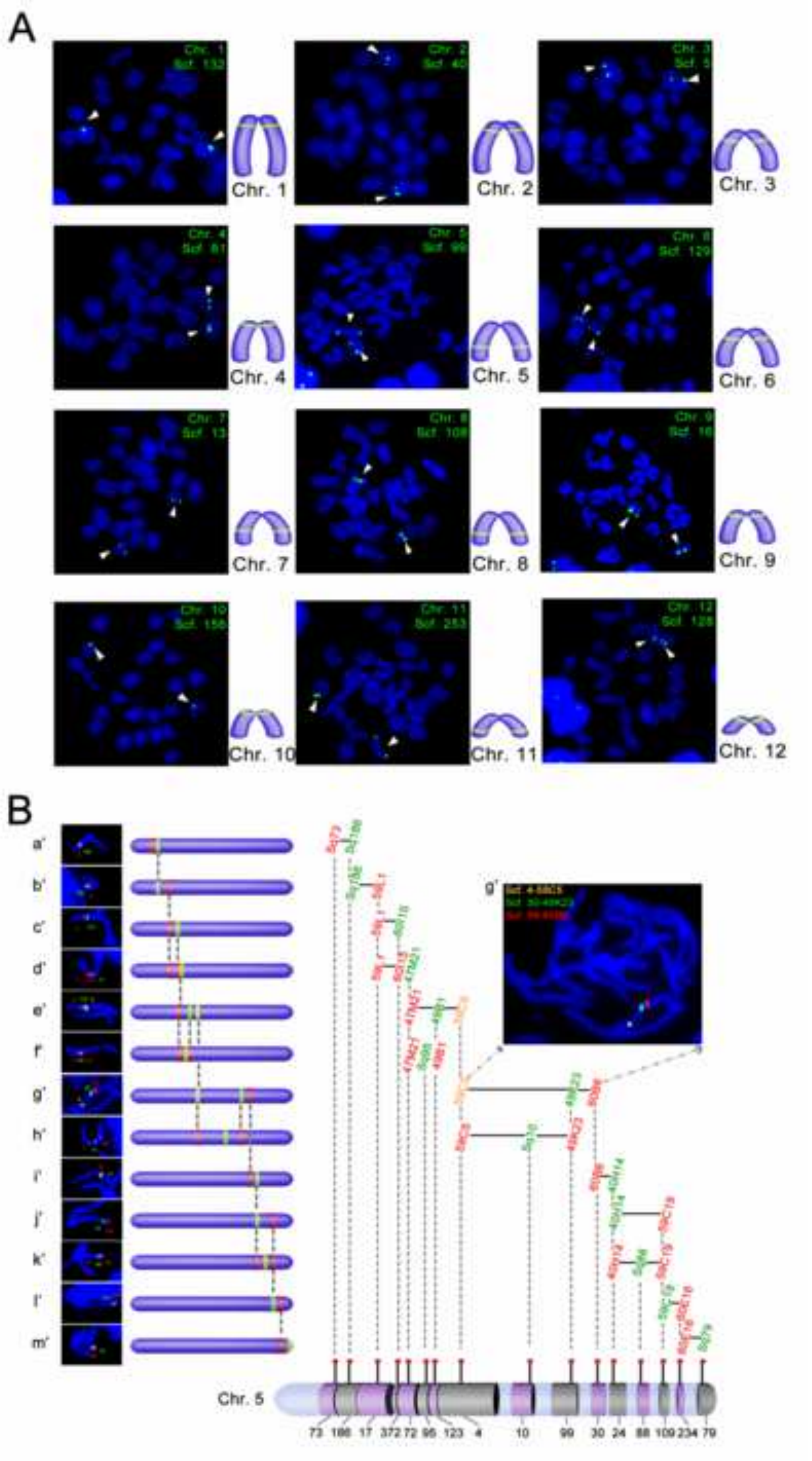

Figure 3

Click here to download Figure Figure 3.tif

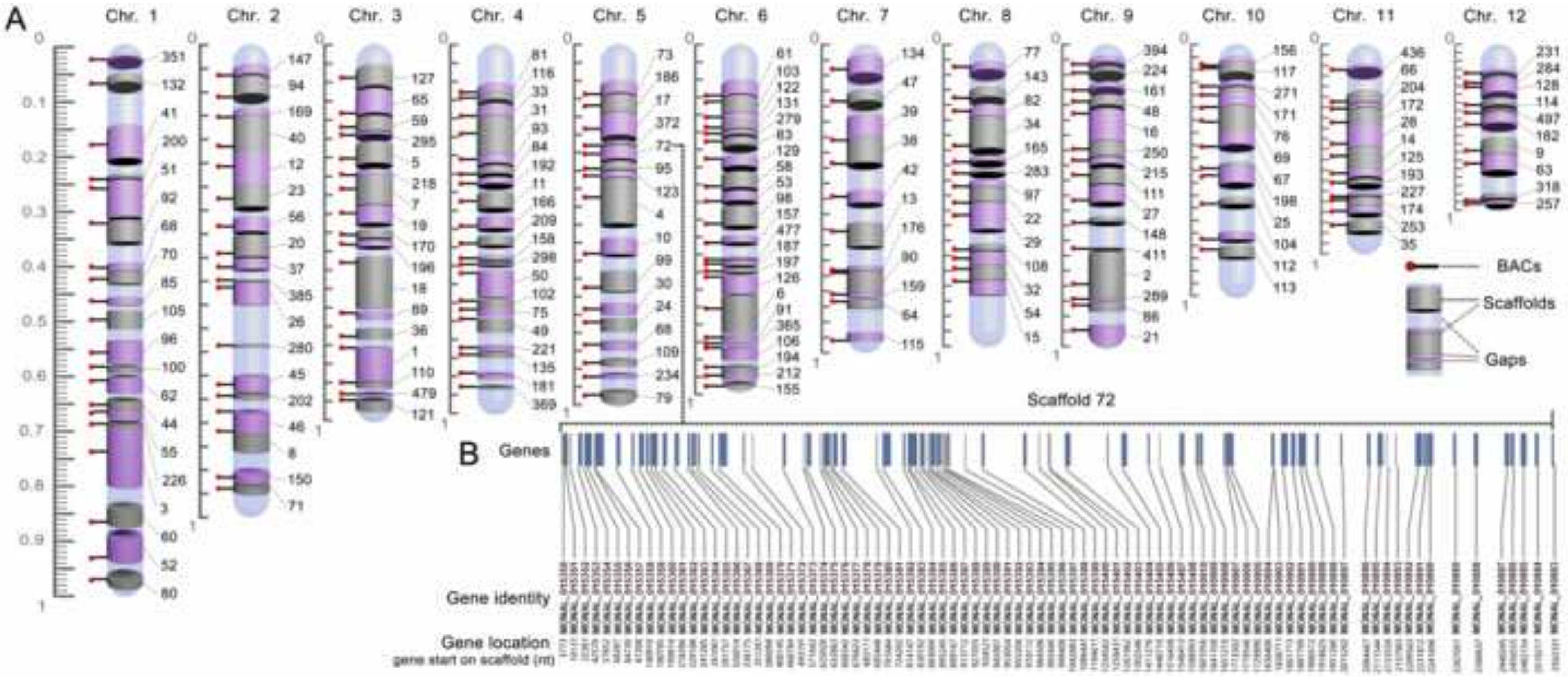

Figure 4

Click here to download Figure Figure 4.tif

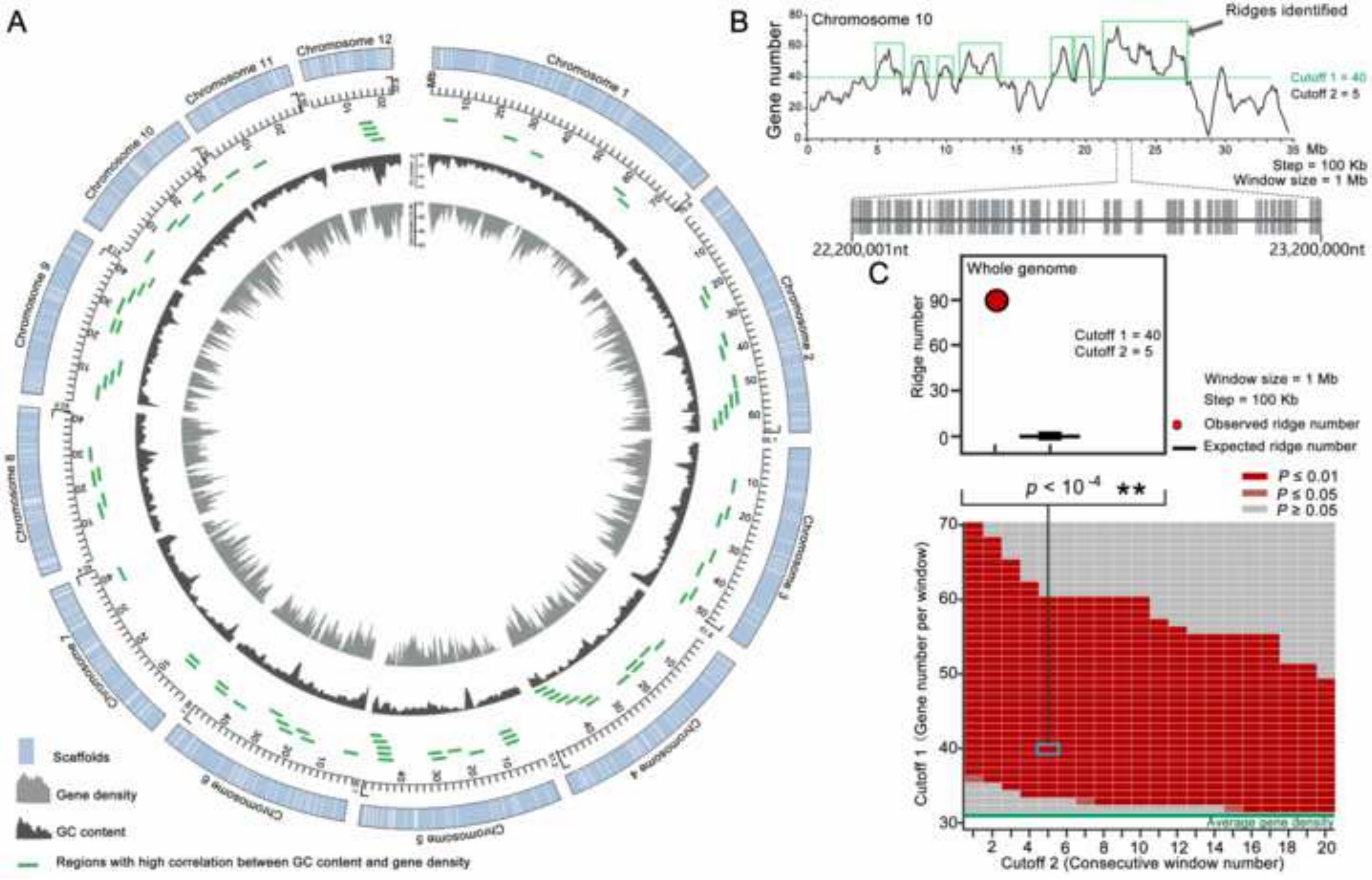

Click here to access/download
Supplementary Material
Additional files-revised.pdf

Click here to access/download
Supplementary Material
Additional files-revised.docx
