# Supplementary material for: Chromosome-scale assembly of the Monopterus genome
Source: Gigascience. 2018 Apr 24;7(5):giy046. doi: 10.1093/gigascience/giy046 (PMC5946948; doi:10.1093/gigascience/giy046)
Supplement: Reviewer_2_(Original_Submission)_Attachment-swamp_eel_plots.pdf [file giy046_reviewer_2_(original_submission)_attachment-swamp_eel_plots.pdf]

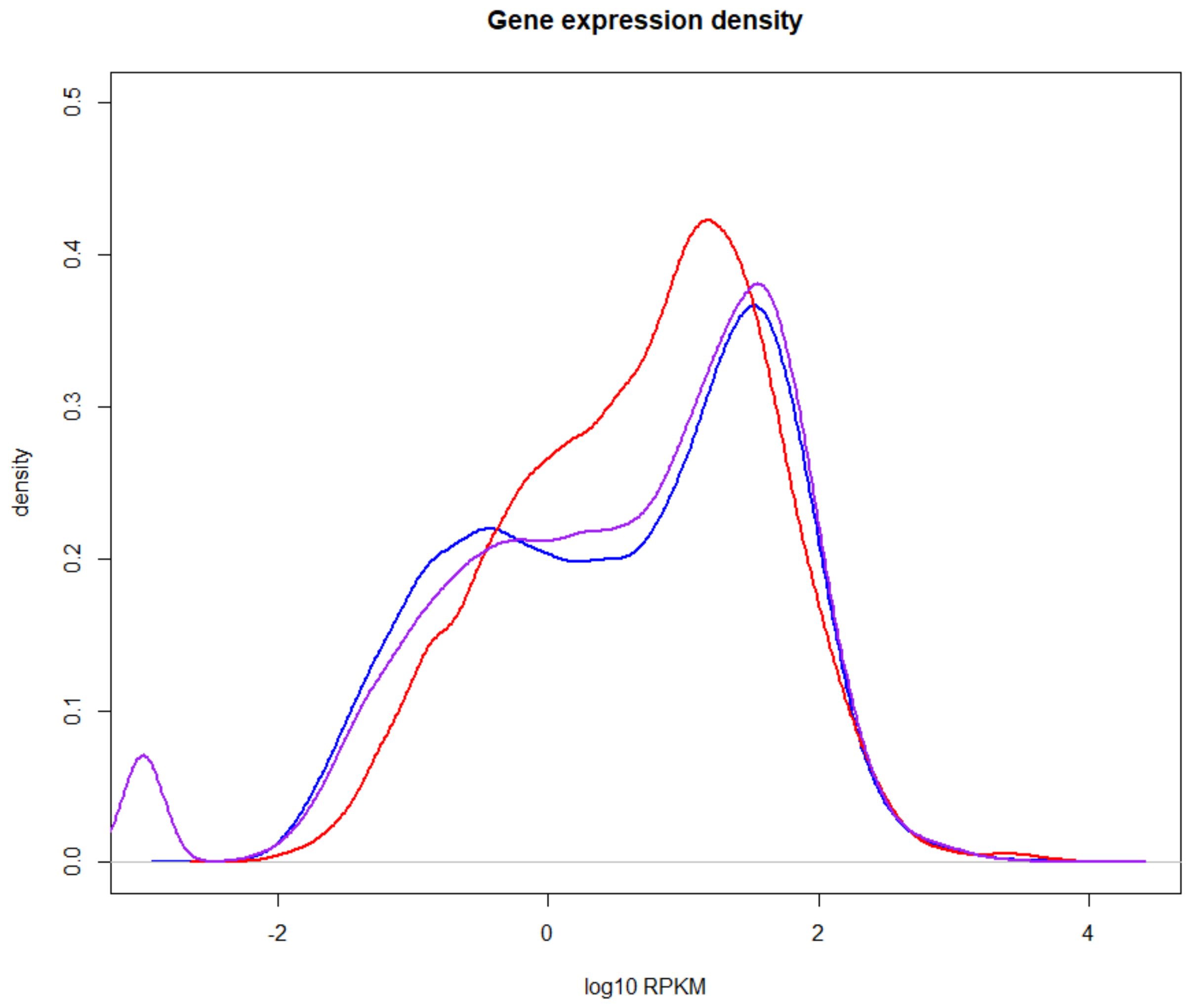

Displayed are the densities of gene expression values. After successful normalization for comparable samples, these plots should overlap as much as possible. Here, they do not and probably cannot.

Blue: ovary; purple: ovotestis; red: testis

Alternatively, scatterplots of the gene expression values also suggest a direct comparison of the testis sample to the other two is not straightforward. For calling a gene up- or down-regulated, the implicit assumption is that the majority of gene expression values do not change significantly. In other words, the trend between two samples should be a (straight) line, with deviations indicating differential expression. Such a trend cannot be established between testis and the other samples, and therefore neither can meaningful differential expression.

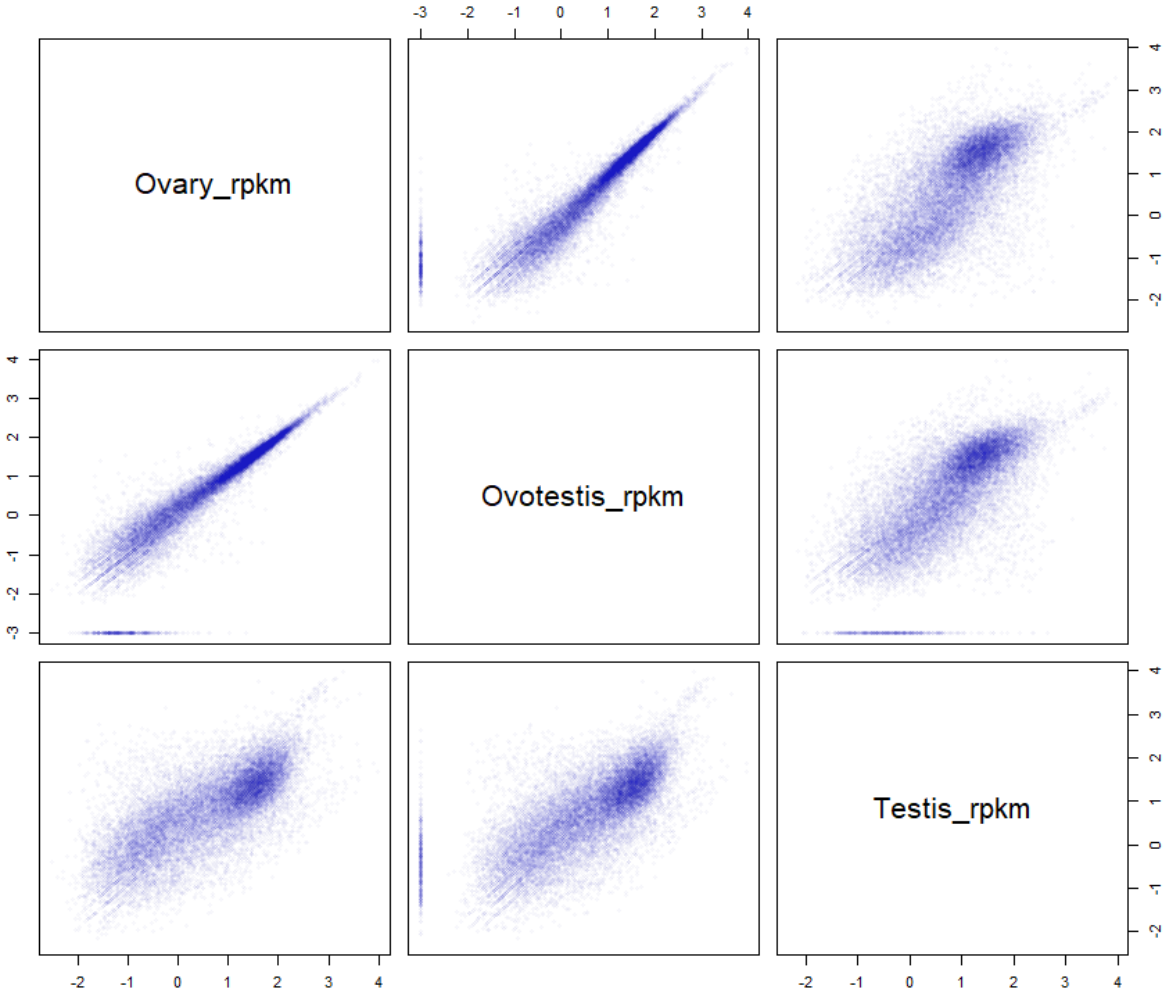

Ovary_rpkm
Ovotestis_rpkm
Testis_rpkm
