# Supplementary material for: Chromosome-scale assembly of the Monopterus genome
Source: Gigascience. 2018 Apr 24;7(5):giy046. doi: 10.1093/gigascience/giy046 (PMC5946948; doi:10.1093/gigascience/giy046)
Supplement: Additional Files [file giy046_supp.zip › Additional files-revised.docx]

**Chromosome-scale assembly of the *Monopterus* genome**

Supporting information

Running title: The Monopterus genome

Xueya Zhao^1^, Majing Luo^1^, Zhigang Li^1^, Pei Zhong^1^, Yibin Cheng^1^, Fengling Lai^1^, Xin Wang^1^, Jiumeng Min^2^, Mingzhou Bai^2^, Yulan Yang^2^, Hanhua Cheng^1^*, Rongjia Zhou^1^*

^1^Hubei Key Laboratory of Cell Homeostasis, Laboratory of Molecular and Developmental Genetics, College of Life Sciences, Wuhan University, Wuhan 430072, P. R. China

^2^BGI Genomics, BGI-Shenzhen, Shenzhen 518083, P. R. China

*Corresponding authors: Professors Rongjia Zhou and Hanhua Cheng, College of Life Sciences, Wuhan University, Wuhan 430072, P. R. China, Fax: 0086-27-68756253, E-mail: rjzhou@whu.edu.cn, hhcheng@whu.edu.cn

Incl:

**Supplemental 10 figures**

**Supplemental 8 tables**

**Supplemental figures**


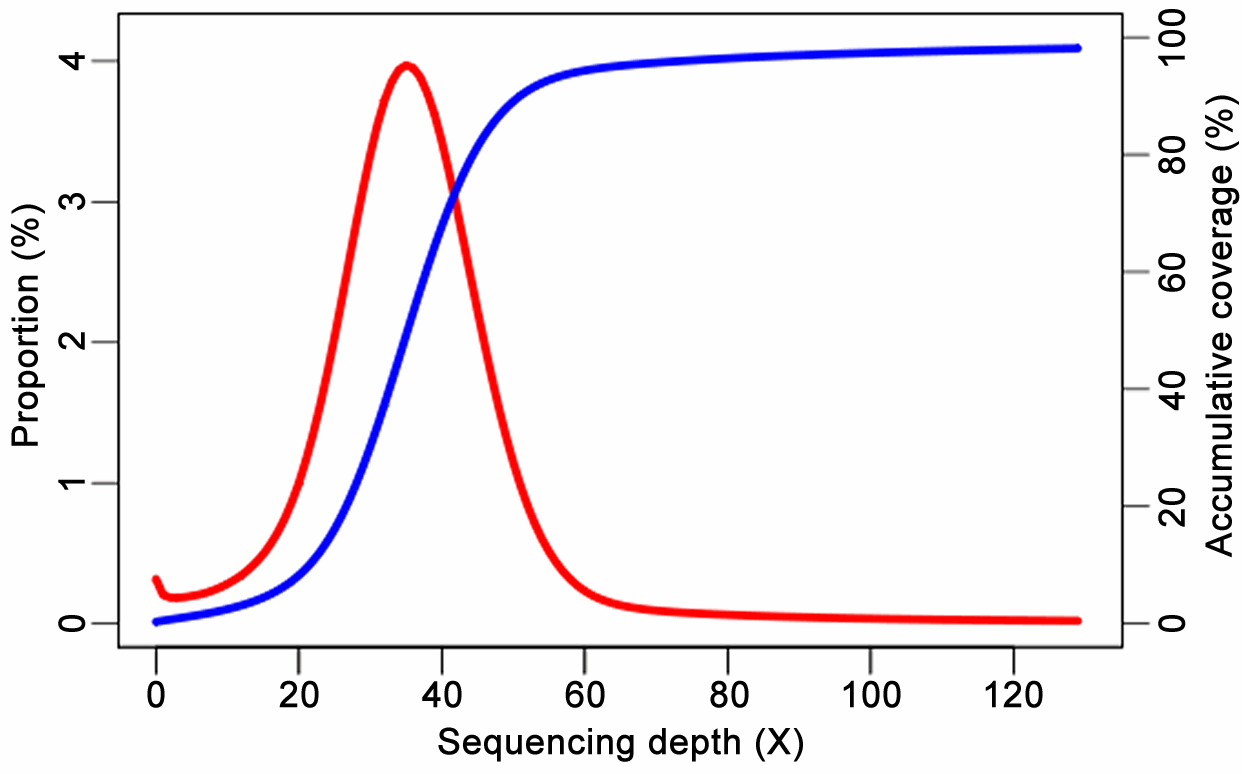


**Figure S1. Sequencing depth distribution of the *Monopterus* genome.** All high quality of reads were aligned onto the assembly and sequencing depth at each position was calculated. The blue curve was accumulative coverage distribution and the red curve represents sequencing depth distribution. We found that the average sequencing depth was 35 and approximately 95.56% of genomic regions in genome were covered by at least 15 reads.


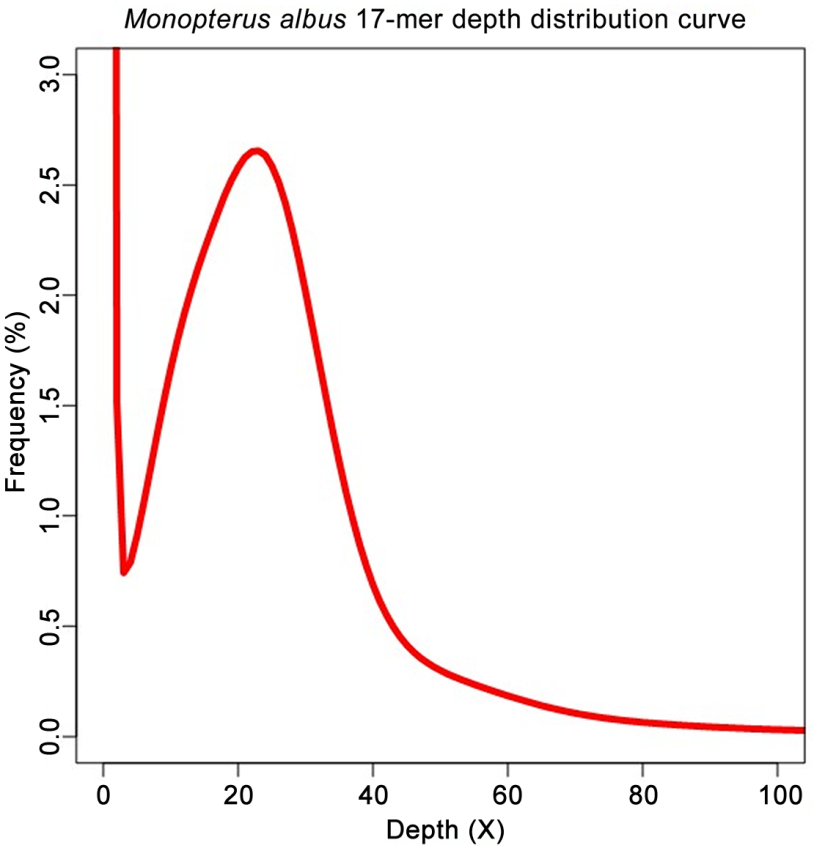


**Figure S2. Genome size estimation using 17-mer.** The x-axis represented sequence depth (X), the y-axis was the proportion, which represented the frequency at that depth divided by the total frequencies at all depths.


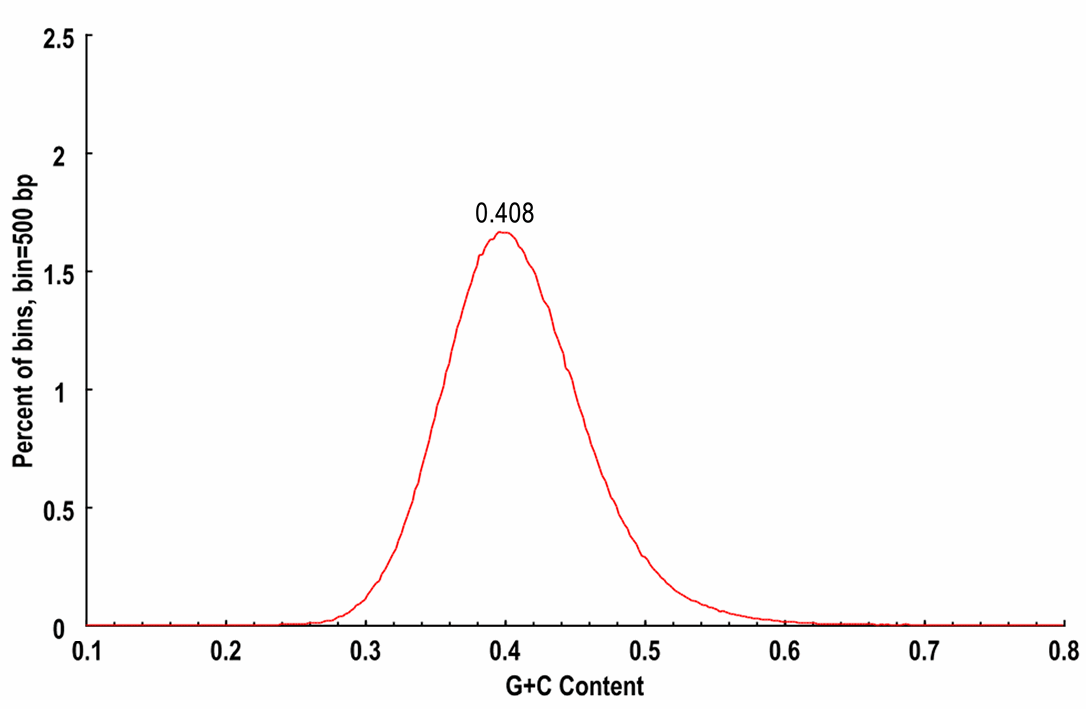


**Figure S3. The GC distribution of the *Monopterus* genome**. GC content distributions for relative species genome. The x-axis is GC content and the y-axis is the proportion of the bins number divided by the total windows. We used 500bp bins (with 250bp overlap) sliding along the genome.


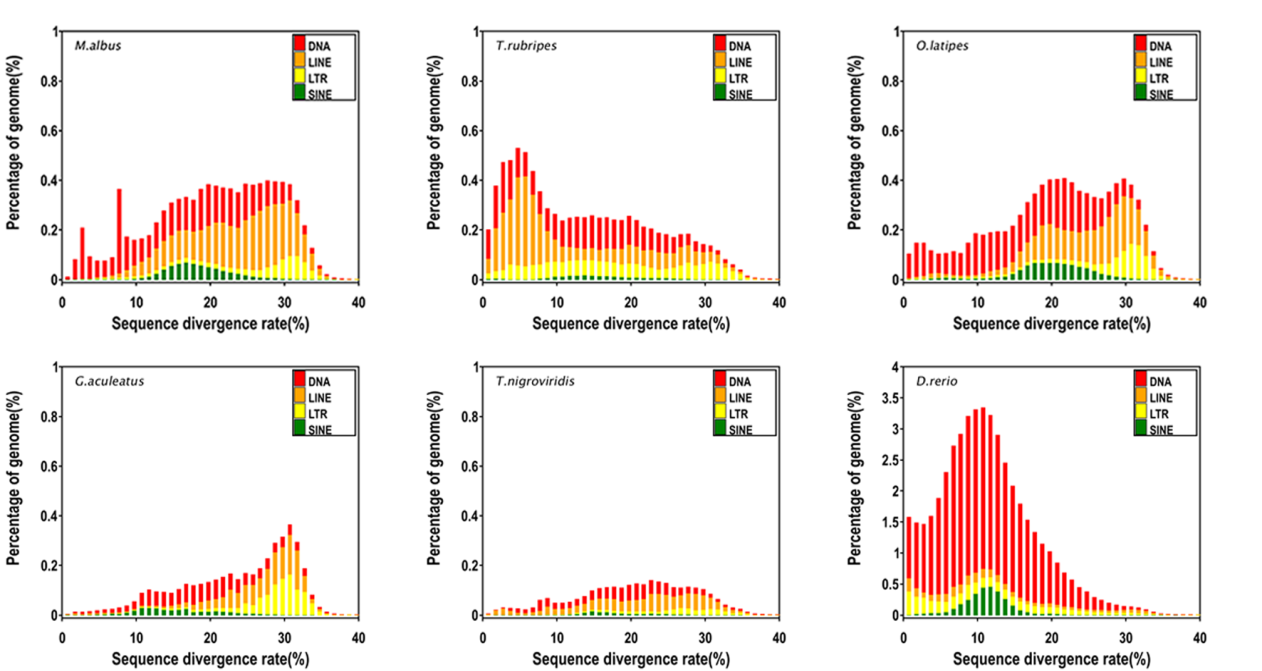


**Figure S4. Divergence distribution of the classified TE elements.** The x-axis represented the sequence divergence rate and the y-axis showed the percentage of genomes. The divergence rate was calculated between the identified TE elements in the genome and the consensus in the TE libraries used Repbase.

**
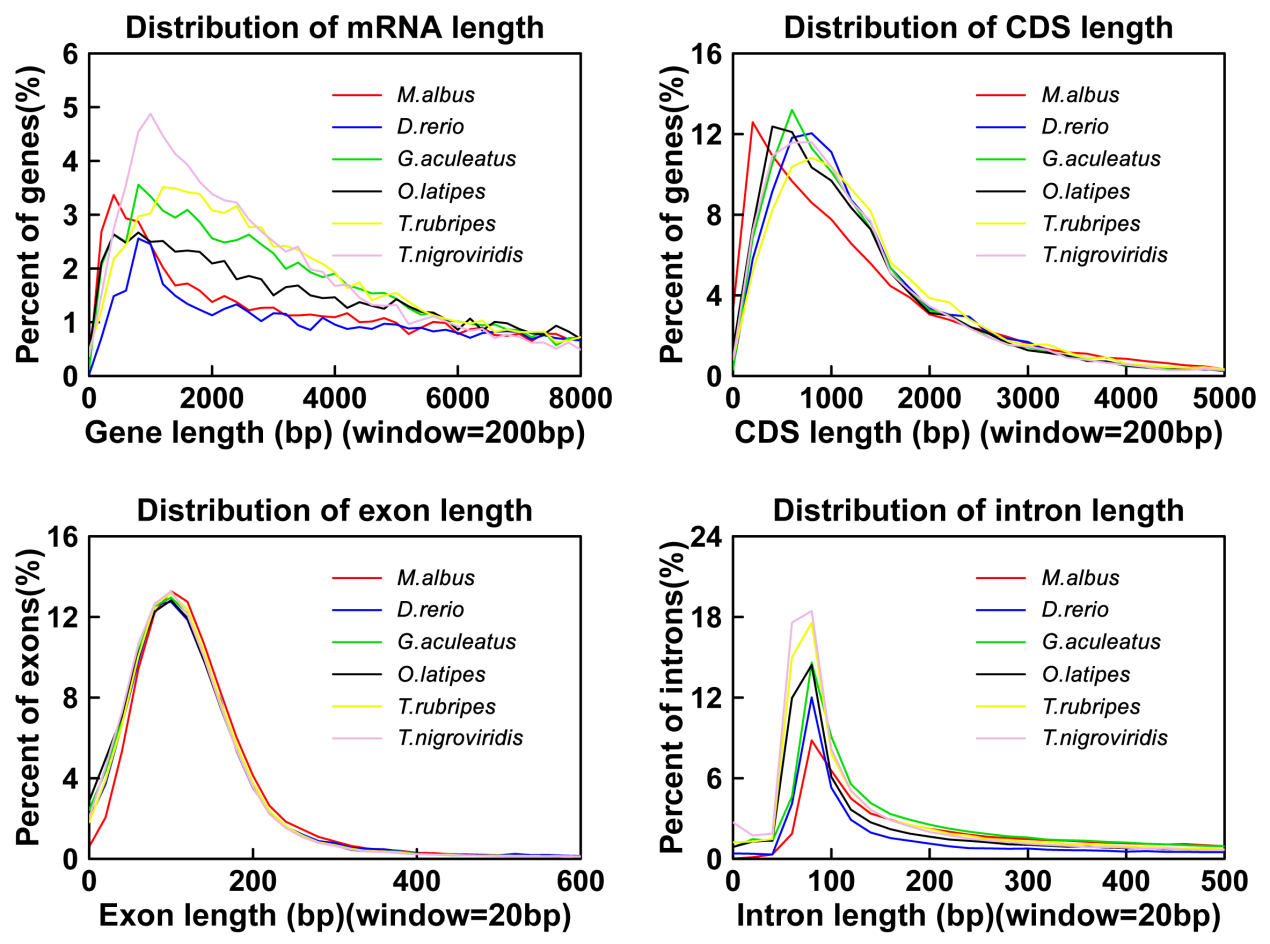
**

**Figure S5. Comparisons of predicted coding genes of *Monopterus* with other teleost fishes.** No obvious unexpected differences were observed among them. *M. albus*, *D. rerio,* *G. aculeatus, O. latipes, T. rubripes* and *T. nigroviridis* are abbreviations for *Monopterus albus*, *Danio rerio, Gasterosteus aculeatus, Oryzias latipes,* *Takifugu rubripes* and *Tetraodon nigroviridis* respectively.`

**
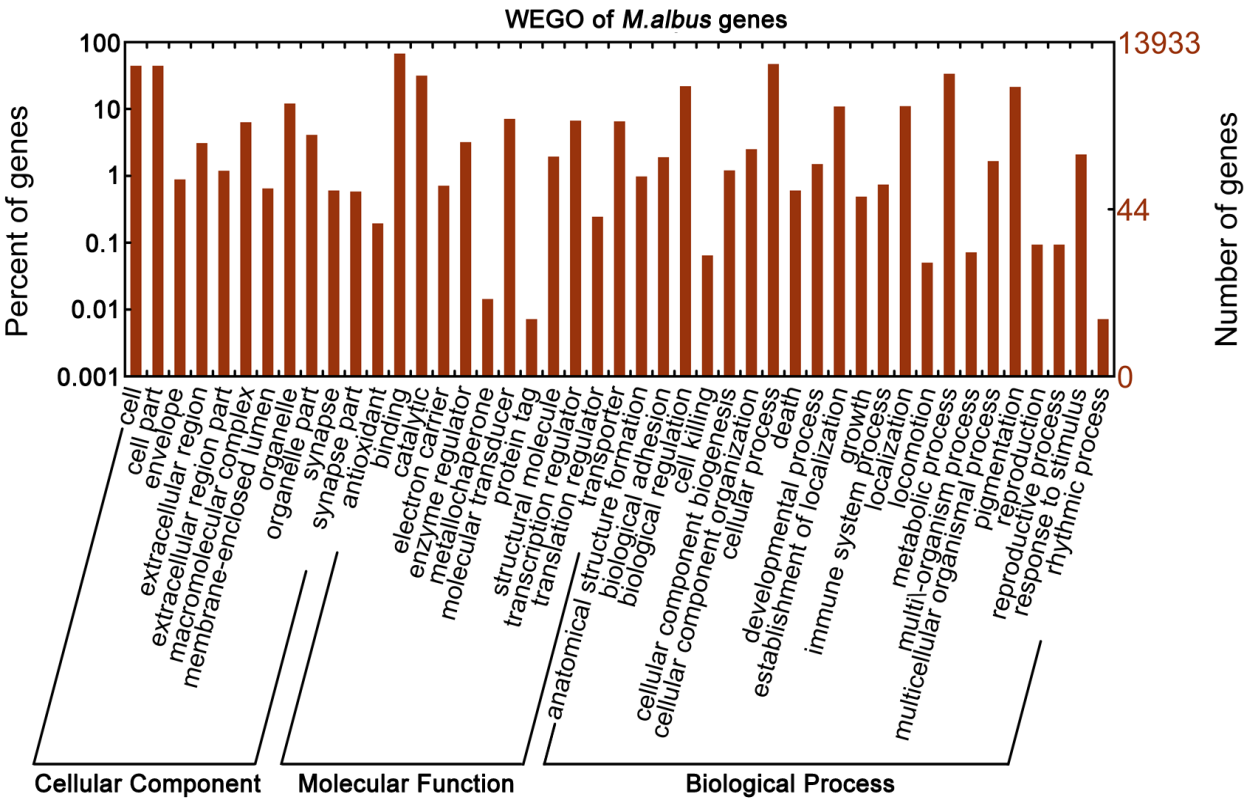
**

**Figure S6. The Gene Ontology of the *Monopterus* genes.** X-axis represented the catalogs of cellular component, molecular function and biological process. Y-axis on left side represented percentage of genes in each catalog and number of genes on the right side.

**

**

**

**

**Figure S7. Localization of each scaffold on chromosomes by FISH-walking strategy.** (A) FISH images and corresponding scaffolds order on chromosomes 1, 2, 3, 4 and 6. (B) FISH images and corresponding scaffolds order on chromosomes 7, 8, 9, 10, 11 and 12. Localization of each scaffold on chromosome was determined according to the FISH images following the alphabet order. Each number presents the scaffold ID. Dotted lines indicate scaffolds determined in previous steps, and solid lines represent scaffolds determined in this step.


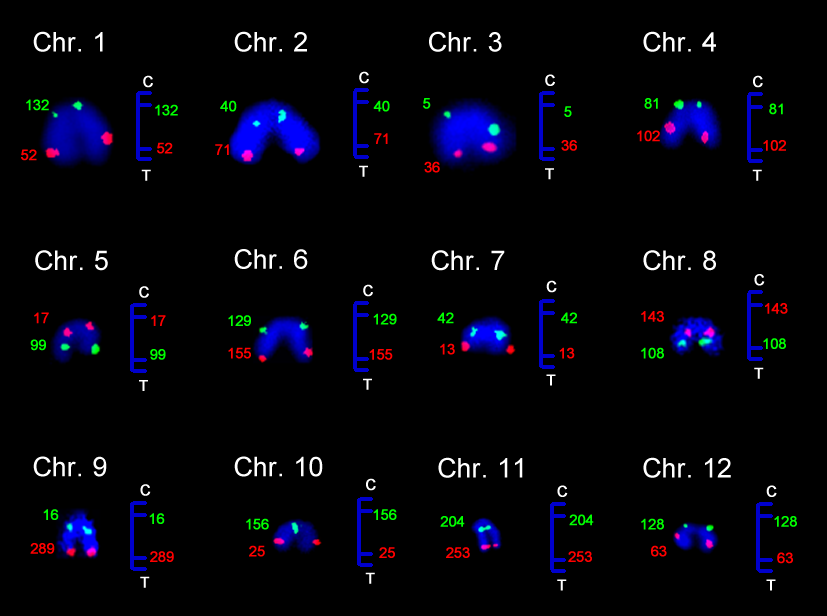


**Figure S8. Orientation of each linkage group on metaphase chromosomes.** Two scaffolds at each end of the chromosome were mapped by FISH and marked with red and green fluorescence respectively. 12 chromosomes derived from 12 cells in mitotic metaphase. Numbers indicates the scaffolds ID. “C”, the centromere of the chromosome; “T”, the telomere of the chromosome.


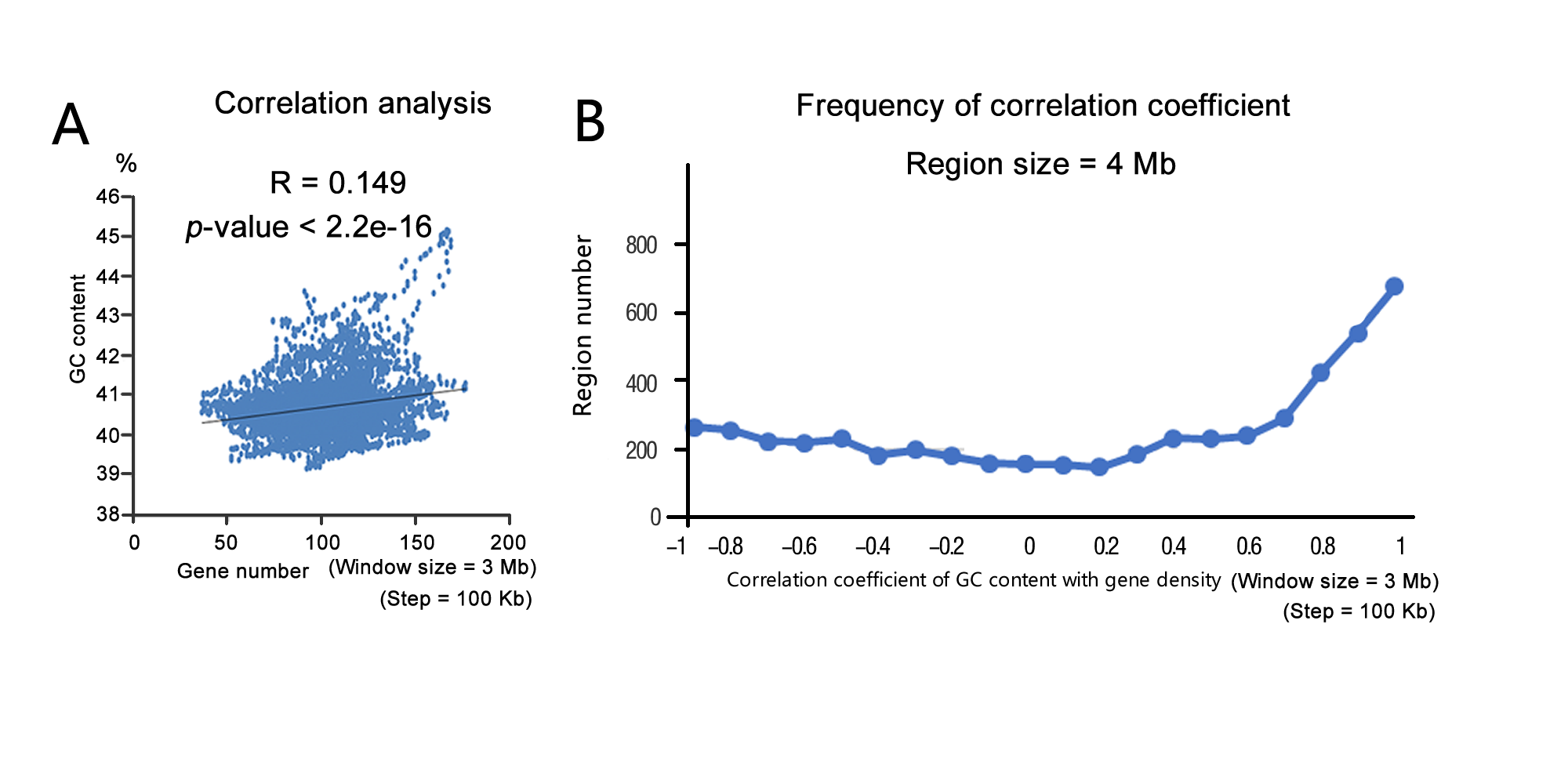


**Figure S9.** **Correlation coefficient of GC content with gene density.** A: Spearman correlation test shows a significantly positive correlation between GC content and gene density. *x*-axis indicates the gene numbers per window of 3 Mb. *y*-axis indicates the GC content per window of 3 Mb. B: Frequency of correlation coefficient shows that there are a large number of 4 Mb regions with lower correlation than 0.7. *x*-axis indicates the region numbers with size of 4 Mb. *y*-axis indicates the correlation coefficient values.

**

**

**Figure S10. Statistical tests of numbers of gene density ridges in the genome corresponding to background noise (null model) in different window sizes (0.2, 0.3, 0.5, 1, 2, 3 Mb).** The heat maps show *p*-values in the significance test of observed ridge numbers against the null model (10,000 independent permutations of gene positions). The *x*-axis indicates the cutoff values of numbers of consecutive moving windows, which reflects the extent of the clustering. The *y*-axis indicates the cutoff values of gene numbers within a certain window size (step 100 kb), which reflects the degree of intensity of the clusters. Green lines represent the average gene number in a certain window size.

**Supplemental tables**

**Table** **S1. Statistics of sequencing.**

| Pair-end libraries | Insert size(bp) | AveraTge reads  length (bp) | Total data (Gb) | Sequence depth (X) | Physical  depth (X) |
| --- | --- | --- | --- | --- | --- |
| Solexa reads | 170 | 98 | 19.82 | 24.59 | 21.32 |
|  | 500 | 98 | 12.85 | 15.94 | 40.67 |
|  | 800 | 98 | 6.54 | 8.11 | 33.14 |
|  | 2K | 49 | 14.45 | 17.93 | 365.88 |
|  | 5K | 49 | 15.56 | 19.31 | 985.17 |
|  | 10K | 49 | 2.82 | 3.50 | 357.64 |
|  | 20K | 49 | 3.91 | 4.85 | 990.66 |
|  | 40K | 49 | 2.69 | 3.34 | 1364.98 |
| Total | ---- | ---- | 78.64 | 97.57 | 2794.48 |

*The *Monopterus* genome size was estimated as 806 Mb

**Table** **S2. Statistics of genome from 17-mer.**

| K | K-mer_num | Peak_depth | Genome size (bp) | Used bases (bp) | X |
| --- | --- | --- | --- | --- | --- |
| 17 | 18,527,714,516 | 23 | 805,552,805 | 22,142,878,324 | 27.49 |

**Table** **S3. Statistics of mapping.**

| **Genome size (Mb)** | **Effective size (Mb)** | **Total reads (M)** | **Map reads (M)** | **Paired end map (M)** | **PE (%)** | **Coverage**  **(M)** | **Coverage**  **(%)** |
| --- | --- | --- | --- | --- | --- | --- | --- |
| 690 | 635 | 389 | 354 | 321 | 90.66 | 633 | 99.69 |

Effective size (Mb): the size of genome without gaps.

Total reads: the number of total reads.

PE (%): Paired end map/ Map reads.

**Table** **S4. Statistics of predicted coding genes.**

| Gene set | | Number |
| --- | --- | --- |
| *De novo* | FGENESH | 39,139 |
|  | GENSCAN | 27,920 |
| Homolog | *D. rerio* | 19,438 |
|  | *G. aculeatus* | 20,071 |
|  | *O. latipes* | 20,084 |
|  | *T. rubripes* | 18,398 |
|  | *T. nigroviridis* | 18,742 |
| GLEAN | | 21,992 |
| RNA-Seq | | 24,056 |

**Table** **S5. Comparisons of predicted coding genes of *Monopterus* with other teleost fishes.**

| Name | Number | Average transcript length (bp) | Average CDS length (bp) | Average exon per gene | Average exon length (bp) | Average intron length (bp) |
| --- | --- | --- | --- | --- | --- | --- |
| *M. albus* | 24,056 | 13473.52 | 1684.06 | 9.50 | 177.27 | 1387.04 |
| *T. rubripes* | 18,507 | 7497.44 | 1694.35 | 11.11 | 152.52 | 574.06 |
| *T.nigroviridis* | 19,583 | 6065.82 | 1517.44 | 10.53 | 144.15 | 477.44 |
| *O. latipes* | 19,671 | 12136.68 | 1517.33 | 10.27 | 147.80 | 1145.94 |
| *G. aculeatus* | 20,772 | 8455.87 | 1549.23 | 10.40 | 148.90 | 734.36 |
| *D. rerio* | 26,046 | 24122.71 | 1593.16 | 9.29 | 171.51 | 2717.92 |

**Table** **S6. Annotated classification of the *Monopterus* genes.**

|  |  | Number | Percent (%) |
| --- | --- | --- | --- |
| Total | | 24,056 | 100.00 |
|  | InterPro | 16,309 | 67.80 |
|  | GO | 13,933 | 57.92 |
| Anonotated | KEGG | 14,766 | 61.38 |
|  | Swissprot | 18,085 | 75.18 |
|  | TrEMBL | 19,230 | 79.94 |
| Unannotated | | 4,709 | 19.58 |

**Table S7. Alignments of BAC ends to reference genome.**

| Scaffold_ID | Scaffold size (bp) | BAC end ID | Start_end  （on scaffold) | BAC end ID | Start_end  (on scaffold) | Insert size (bp) |
| --- | --- | --- | --- | --- | --- | --- |
| scaffold2 | 8873700 | 60K3-PIBFP-1-F_E05.seq(-) | 2603868_2602920 | 60K3-PIBRP-1-R_F05.seq(+) | 2492037_2492554 | 111831 |
| scaffold3 | 11676166 | 49G9-PIBFP-1-F_A06.seq(+) | 5063689_5064440 | 49G9-PIBFP-1-R_B06.seq(-) | 5165842_5165011 | 102153 |
| scaffold4 | 8771891 | 59C5-PIBRP-1-F_E10.seq(-) | 3502358_3501411 | 59C5-PIBRP-1-R_F10.seq(+) | 3394427_3395184 | 107931 |
| scaffold5 | 4089012 | 59K4-PIBRP-1-F_E07.seq(-) | 2782018_2781231 | 59K4-PIBRP-1-R_F07.seq(+) | 2677780_2678675 | 104238 |
| scaffold6 | 7362726 | 49G23-PIBFP-1-F_A10.seq(-) | 4739737_4738857 | 49G23-PIBFP-1-R_B10.seq(+) | 4629351_4629856 | 110386 |
| scaffold7 | 5747303 | 60I10-PIBFP-1-F_G07.seq(+) | 2391662_2392602 | 60I10-PIBRP-1-R_H07.seq(-) | 2501108_2500262 | 109446 |
| scaffold9 | 3226598 | 59D8-PIBFP-1-F_E04.seq(+) | 1864946_1865848 | 59D8-PIBFP-1-R_F04.seq(-) | 1970488_1969544 | 105542 |
| scaffold11 | 3114667 | 60I11-PIBFP-1-F_G09.seq(+) | 1442545_1443460 | 60I11-PIBRP-1-R_H09.seq(-) | 1545921_1545126 | 103376 |
| scaffold12 | 6447076 | 60L8-PIBFP-1-F_E03.seq(-) | 2602072_2601166 | 60L8-PIBRP-1-R_F03.seq(+) | 2501530_2502305 | 100542 |
| scaffold13 | 4588008 | 49C3-PIBFP-1F_E06.seq(-) | 1364742_1363809 | 49C3-PIBFP-1R_F06.seq(+) | 1268231_1268660 | 96511 |
| scaffold14 | 4294272 | 47H18-PIBFP-1-F_A12.seq(-) | 1197160_1196350 | 47H18-PIBFP-1-R_B12.seq(+) | 1107027_1107818 | 90133 |
| scaffold16 | 6086815 | 59A18-PIBFP-1-F_G12.seq(+) | 5942973_5943882 | 59A18-PIBRP-1-R_H12.seq(-) | 6075594_6074799 | 132621 |
| scaffold17 | 4474239 | 59L1-PIBFP-1-F_E01.seq(+) | 1686876_1687822 | 59L1-PIBFP-1-R_F01.seq(-) | 1785693_1784830 | 98817 |
| scaffold18 | 10146043 | 47O2-PIBFP-1-F_A03.seq(+) | 9057544_9058328 | 47O2-PIBRP-1-R_B03.seq(-) | 9159513_9158593 | 101969 |
| scaffold19 | 3416764 | 59N9-PIBFP-1-F_C05.seq(-) | 2155965_2155089 | 59N9-PIBFP-1-R_D05.seq(+) | 2056480_2056945 | 99485 |
| scaffold20 | 4175638 | 60C19-PIBFP-1-F_E02.seq(-) | 3497974_3497029 | 60C19-PIBFP-1-R_F02.seq(+) | 3379075_3379978 | 118899 |
| scaffold21 | 4120493 | 59E17-PIBFP-1-F_C10.seq(+) | 3087193_3088132 | 59E17-PIBRP-1-R_D10.seq(-) | 3201394_3200547 | 114201 |
| scaffold23 | 4207233 | 10C4-PIBFP-1-F_E08.seq(+) | 2343655_2344243 | 10C4-PIBFP-1-R_F08.seq(-) | 2409653_2408762 | 65998 |
| scaffold24 | 2734378 | 40H14-PIBFP-1-F_A04.seq(+) | 1005262_1006184 | 40H14-PIBRP-1-R_B04.seq(-) | 1098549_1097598 | 93287 |
| scaffold25 | 3106006 | 47M23-PIBRP-1-F_C09.seq(-) | 1933865_1932996 | 47M23-PIBRP-1-R_D09.seq(+) | 1811897_1812723 | 121968 |
| scaffold26 | 4322107 | 59C9-PIBRP-1-F_E06.seq(+) | 3388550_3389496 | 59C9-PIBRP-1-R_F06.seq(-) | 3500399_3499772 | 111849 |
| scaffold27 | 3247232 | 59D4-PIBFP-1-F_E08.seq(-) | 690342_689459 | 59D4-PIBFP-1-R_F08.seq(+) | 580406_581142 | 109936 |
| scaffold28 | 4311598 | 49F2-PIBFP-1-F_C04.seq(-) | 3275721_3274767 | 49F2-PIBRP-1-R_D04.seq(+) | 3169565_3170347 | 106156 |
| scaffold29 | 5206414 | 59J4-PIBFP-1-F_G07.seq(+) | 2012751_2013645 | 59J4-PIBFP-1-R_H07.seq(-) | 2129388_2128564 | 116637 |
| scaffold30 | 2219514 | 60B6-PIBFP-1-F_G12.seq(+) | 917059_917917 | 60B6-PIBRP-1-R_H12.seq(-) | 1026934_1026087 | 109875 |
| scaffold31 | 7233435 | 49M19-PIBFP-1-F_C01.seq(+) | 4314616_4315539 | 49M19-PIBFP-1-R_D01.seq(-) | 4423745_4422908 | 109129 |
| scaffold32 | 2547755 | 47G18-PIBRP-1-F_A12.seq(+) | 994017_994901 | 47G18-PIBRP-1-R_B12.seq(-) | 1046390_1045539 | 52373 |
| scaffold33 | 2178718 | 49O5-PIBFP-1F_A09.seq(-) | 379104_378191 | 49O5-PIBFP-1R_B09.seq(+) | 356709_357425 | 22395 |
| scaffold34 | 6032531 | 47A22-PIBRP-1-F_G08.seq(-) | 5053738_5052789 | 47A22-PIBRP-1-R_H08.seq(+) | 4968022_4968372 | 85716 |
| scaffold36 | 2106322 | 10C1-PIBFP-1-F_E02.seq(+) | 1654704_1655672 | 10C1-PIBFP-1-R_F02.seq(-) | 1699163_1698231 | 44459 |
| scaffold37 | 2091753 | 10M3-PIBFP-1-F_C05.seq(+) | 1774050_1774806 | 10M3-PIBFP-1-R_D05.seq(-) | 1828426_1827518 | 54376 |
| scaffold38 | 4842900 | 60K14-PIBFP-1-F_E03.seq(-) | 4579887_4579114 | 60K14-PIBFP-1-R_F03.seq(+) | 4463460_4464082 | 116427 |
| scaffold40 | 6890715 | 59G3-PIBRP-1-F_A06.seq(-) | 1309643_1308772 | 59G3-PIBRP-1-R_B06.seq(+) | 1197005_1197738 | 112638 |
| scaffold41 | 6455291 | 40A20-PIBFP-1-F_G04.seq(+) | 3403980_3404884 | 40A20-PIBRP-1-R_H04.seq(-) | 3506990_3506029 | 103010 |
| scaffold42 | 2432183 | 59E24-PIBFP-1-F_C12.seq(+) | 900635_901472 | 59E24-PIBRP-1-R_D12.seq(-) | 993879_993087 | 93244 |
| scaffold44 | 1937076 | 59J5-PIBFP-1-F_G09.seq(-) | 1015883_1014987 | 59J5-PIBFP-1-R_H09.seq(+) | 903672_904262 | 112211 |
| scaffold45 | 3009193 | 10E9-PIB-FP_C06.seq(-) | 1126279_1125366 | 10E9-PIB-RP_D06.seq(+) | 1087759_1088466 | 38520 |
| scaffold46 | 3749709 | 59N1-PIBFP-1-F_C01.seq(+) | 38878_39780 | 59N1-PIBFP-1-R_D01.seq(-) | 134956_134285 | 96078 |
| scaffold50 | 5224853 | 59J24-PIBRP1-F_G11.seq(-) | 521626_520775 | 59J24-PIBRP1-R_H11.seq(+) | 458828_459668 | 62798 |
| scaffold51 | 7210046 | 59D1-PIBFP-1-F_E02.seq(-) | 5722791_5722014 | 59D1-PIBFP-1-R_F02.seq(+) | 5594766_5595657 | 128025 |
| scaffold52 | 4557624 | 49M23-PIBFP-1-F_C09.seq(+) | 4340268_4341171 | 49M23-PIBFP-1-R_D09.seq(-) | 4442247_4441590 | 101979 |
| scaffold54 | 2755434 | 60P20-PIBRP-1-F_A03.seq(-) | 2309159_2308208 | 60P20-PIBRP-1-R_B03.seq(+) | 2202989_2203863 | 106170 |
| scaffold55 | 1799210 | 59P15-PIBRP1F_A05.seq(+) | 451231_452156 | 59P15-PIBRP1R_B05.seq(-) | 573838_572998 | 122607 |
| scaffold56 | 2862434 | 59E12-PIBRP-1-F_C12.seq(+) | 358704_359630 | 59E12-PIBRP-1-R_D12.seq(-) | 484437_483570 | 125733 |
| scaffold59 | 2628122 | 10A6-PIBFP-1-F_G12.seq(-) | 1283224_1282631 | 10A6-PIBFP-1-R_H12.seq(+) | 1222432_1223287 | 60792 |
| scaffold60 | 3273427 | 60D6-PIBFP-1-F_E12.seq(-) | 392550_391691 | 60D6-PIBRP-1-R_F12.seq(+) | 285524_286283 | 107026 |
| scaffold61 | 2717057 | 40G18-PIBFP-1-F_A12.seq(+) | 2330175_2331096 | 40G18-PIBRP-1-R_B12.seq(-) | 2420155_2419205 | 89980 |
| scaffold62 | 3130620 | 49G10-PIBFP-1-F_A08.seq(-) | 921297_920348 | 49G10-PIBFP-1-R_B08.seq(+) | 815318_816181 | 105979 |
| scaffold63 | 3058335 | 60D17-PIBRP-1-F_E10.seq(+) | 1162764_1163708 | 60D17-PIBRP-1-R_F10.seq(-) | 1267467_1266582 | 104703 |
| scaffold64 | 1719917 | 60J1-PIBFP-1-F_G01.seq(-) | 340563_339663 | 60J1-PIBRP-1-R_H01.seq(+) | 230860_231663 | 109703 |
| scaffold66 | 2151455 | 49M13-PIBFP1-F_C01.seq(-) | 1309881_1308939 | 49M13-PIBFP1-R_D01.seq(+) | 1222716_1223559 | 87165 |
| scaffold68 | 1627818 | 40O20-PIBFP-1-F_A03.seq(-) | 882645_881698 | 40O20-PIBRP-1-R_B03.seq(+) | 784035_784809 | 98610 |
| scaffold69 | 4807879 | 60F13-PIBRP-1-F_C02.seq(+) | 2441869_2442818 | 60F13-PIBRP-1-R_D02.seq(-) | 2547925_2547326 | 106056 |
| scaffold70 | 2250409 | 40B24-PIBRP1-F_G12.seq(-) | 1398889_1397967 | 40B24-PIBRP1-R_H12.seq(+) | 1305086_1305948 | 93803 |
| scaffold71 | 1600240 | 59E18-PIBFP-1-F_C12.seq(-) | 1177001_1176123 | 59E18-PIBRP-1-R_D12.seq(+) | 1076981_1077798 | 100020 |
| scaffold72 | 2567831 | 47M21-PIBRP-1-F_C05.seq(-) | 1279202_1278252 | 47M21-PIBRP-1-R_D05.seq(+) | 1187607_1188385 | 91595 |
| scaffold80 | 1488826 | 40A18-PIBFP-1-F_G12.seq(+) | 372125_373036 | 40A18-PIBRP-1-R_H12.seq(-) | 473162_472213 | 101037 |
| scaffold81 | 1740688 | 59J19-PIBRP1-F_G01.seq(+) | 1187261_1187883 | 59J19-PIBRP1-R_H01.seq(-) | 1297263_1296765 | 110002 |
| scaffold82 | 2201039 | 47K19-PIBRP-1-F_E01.seq(-) | 507905_507153 | 47K19-PIBRP-1-R_F01.seq(+) | 429283_430044 | 78622 |
| scaffold83 | 1471024 | 5909-PIBRP-1-F_A05.seq(+) | 289912_290854 | 5909-PIBRP-1-R_B05.seq(-) | 381223_380337 | 91311 |
| scaffold85 | 1460651 | 47E20-PIBRP-1-F_C04.seq(+) | 808623_809573 | 47E20-PIBRP-1-R_D04.seq(-) | 887675_886995 | 79052 |
| scaffold86 | 1869716 | 59E1-PIBRP-1-F_C02.seq(-) | 1228554_1227605 | 59E1-PIBRP-1-R_D02.seq(+) | 1110836_1111662 | 117718 |
| scaffold89 | 1662256 | 60H13-PIBRP-1-F_A02.seq(-) | 355641_354886 | 60H13-PIBRP-1-R_B02.seq(+) | 235722_236356 | 119919 |
| scaffold90 | 1429129 | 60C5-PIBFP-1-F_E10.seq(-) | 239259_238437 | 60C5-PIBRP-1-R_F10.seq(+) | 134597_135356 | 104662 |
| scaffold92 | 4208067 | 49M3-PIBFP-1F_C05.seq(-) | 3832190_3831274 | 49M3-PIBFP-1R_D05.seq(+) | 3723853_3724789 | 108337 |
| scaffold93 | 1801319 | 59L13-PIBRP1F_E01.seq(-) | 648480_647608 | 59L13-PIBRP1R_F01.seq(+) | 531240_532058 | 117240 |
| scaffold94 | 3287358 | 49M15-PIBFP1-F_C05.seq(+) | 2864_3574 | 49M15-PIBFP1-R_D05.seq(-) | 108972_108040 | 106108 |
| scaffold96 | 4483444 | 40B19-PIBRP1-F_G02.seq(+) | 2421480_2422437 | 40B19-PIBRP1-R_H02.seq(-) | 2541278_2540533 | 119798 |
| scaffold97 | 1401509 | 49G18-PIBFP1-F_A12.seq(-) | 899550_898656 | 49G18-PIBFP1-R_B12.seq(+) | 798443_799267 | 101107 |
| scaffold98 | 3814451 | 60A7-PIBFP-1-F_G02.seq(-) | 617758_616826 | 60A7-PIBRP-1-R_H02.seq(+) | 513336_514177 | 104422 |
| scaffold99 | 4035179 | 49K23-PIBFP-1-F_E09.seq(-) | 3275808_3274868 | 49K23-PIBFP-1-R_F09.seq(+) | 3177090_3177949 | 98718 |
| scaffold100 | 1335476 | 40P13-PIBRP1-F_A01.seq(-) | 1216566_1215638 | 40P13-PIBRP1-R_B01.seq(+) | 1107945_1108784 | 108621 |
| scaffold102 | 1772076 | 59L22-PIBRP1-F_E07.seq(+) | 475151_476070 | 59L22-PIBRP1-R_F07.seq(-) | 586732_585877 | 111581 |
| scaffold103 | 3201532 | 60P22-PIBRP-1-F_A07.seq(-) | 2773968_2773077 | 60P22-PIBRP-1-R_B07.seq(+) | 2679766_2680513 | 94202 |
| scaffold105 | 3645435 | 49M20-PIBFP-1-F_C03.seq(-) | 1929697_1928748 | 49M20-PIBFP-1-R_D03.seq(+) | 1826971_1827442 | 102726 |
| scaffold108 | 1292301 | 59J21-PIBRP1-F_G05.seq(-) | 265971_265354 | 59J21-PIBRP1-R_H05.seq(+) | 142937_143761 | 123034 |
| scaffold109 | 1265270 | 59C19-PIBFP-1-F_E02.seq(+) | 730344_731293 | 59C19-PIBRP-1-R_F02.seq(-) | 823316_822410 | 92972 |
| scaffold110 | 1264126 | 47B13-PIBFP-1-F_G02.seq(+) | 393049_393926 | 47B13-PIBFP-1-R_H02.seq(-) | 446559_446119 | 53510 |
| scaffold111 | 2847258 | 47M15-PIBRP-1-F_C05.seq(-) | 2062904_2062235 | 47M15-PIBRP-1-R_D05.seq(+) | 2015636_2016498 | 47268 |
| scaffold113 | 2130100 | 40J13-PIBFP-1-F_G01.seq(-) | 1693561_1692670 | 40J13-PIBRP-1-R_H01.seq(+) | 1577257_1578023 | 116304 |
| scaffold114 | 1257335 | 60I9-PIBFP-1-F_G05.seq(-) | 505673_504727 | 60I9-PIBRP-1-R_H05.seq(+) | 386151_387021 | 119522 |
| scaffold115 | 1718868 | 60P7-PIBFP-1-F_A01.seq(+) | 223420_224361 | 60P7-PIBRP-1-R_B01.seq(-) | 324794_323990 | 101374 |
| scaffold116 | 1298005 | 60J16-PIBRP-1-F_G07.seq(+) | 382579_383528 | 60J16-PIBRP-1-R_H07.seq(-) | 471292_470543 | 88713 |
| scaffold117 | 1836875 | 59B15-PIBRP1F_G06.seq(-) | 1627066_1626192 | 59B15-PIBRP1R_H06.seq(+) | 1497600_1498405 | 129466 |
| scaffold122 | 1519751 | 60M8-PIBFP-1-F_C03.seq(-) | 213922_213082 | 60M8-PIBRP-1-R_D03.seq(+) | 102656_103490 | 111266 |
| scaffold123 | 1193958 | 49B1-PIBFP-1-F_G02.seq(-) | 667706_666758 | 49B1-PIBRP-1-R_H02.seq(+) | 628117_629025 | 39589 |
| scaffold125 | 1508297 | 49N3-PIBFP-1-F_C05.seq(-) | 295326_294553 | 49N3-PIBRP-1-R_D05.seq(+) | 180865_181729 | 114461 |
| scaffold126 | 3583832 | 59B2-PIBFP-1-F_G04.seq(+) | 251170_252119 | 59B2-PIBFP-1-R_H04.seq(-) | 359295_358927 | 108125 |
| scaffold128 | 2370831 | 60I21-PIBFP-1-F_G05.seq(-) | 1790807_1790021 | 60I21-PIBFP-1-R_H05.seq(+) | 1694886_1695752 | 95921 |
| scaffold129 | 2048959 | 60G7-PIBFP-1-F_A02.seq(+) | 207617_208223 | 60G7-PIBRP-1-R_B02.seq(-) | 327236_326386 | 119619 |
| scaffold131 | 1438011 | 60G6-PIBFP-1-F_A12.seq(+) | 587072_587589 | 60G6-PIBRP-1-R_B12.seq(-) | 689308_688409 | 102236 |
| scaffold132 | 1659295 | 59G5-PIBRP-1-F_A10.seq(-) | 1336739_1335791 | 59G5-PIBRP-1-R_B10.seq(+) | 1225972_1226878 | 110767 |
| scaffold143 | 1945595 | 60D5-PIBFP-1-F_E10.seq(+) | 1321032_1321960 | 60D5-PIBRP-1-R_F10.seq(-) | 1412865_1412108 | 91833 |
| scaffold147 | 1838780 | 60K7-PIBFP-1-F_E01.seq(-) | 403833_403201 | 60K7-PIBRP-1-R_F01.seq(+) | 295428_296267 | 108405 |
| scaffold148 | 963564 | 47E18-PIBRP-1-F_C12.seq(-) | 147416_146536 | 47E18-PIBRP-1-R_D12.seq(+) | 105522_106344 | 41894 |
| scaffold150 | 1603186 | 47I14-PIBRP-1-F_G03.seq(-) | 1105596_1104790 | 47I14-PIBRP-1-R_H03.seq(+) | 1064341_1065191 | 41255 |
| scaffold155 | 944781 | 59G23-PIBFP-1-F_A10.seq(-) | 621068_620172 | 59G23-PIBRP-1-R_B10.seq(+) | 513921_514770 | 107147 |
| scaffold156 | 976802 | 59D2-PIBFP-1-F_E04.seq(-) | 249142_248193 | 59D2-PIBFP-1-R_F04.seq(+) | 204905_205414 | 44237 |
| scaffold157 | 1325698 | 60A17-PIBFP-1-F_G10.seq(-) | 1238997_1238166 | 60A17-PIBFP-1-R_H10.seq(+) | 1151296_1151978 | 87701 |
| scaffold159 | 928226 | 49L9-PIBFP-1-F_G05.seq(-) | 255495_254545 | 49L9-PIBFP-1-R_H05.seq(+) | 213606_214511 | 41889 |
| scaffold161 | 919015 | 59N20-PIBRP1-F_C03.seq(-) | 731800_730883 | 59N20-PIBRP1-R_D03.seq(+) | 701748_702626 | 30052 |
| scaffold165 | 903223 | 49E4-PIBFP-1F_C08.seq(-) | 621495_620595 | 49E4-PIBFP-1R_D08.seq(+) | 505892_506760 | 115603 |
| scaffold166 | 2351422 | 10O2-PIBFP-1-F_A03.seq(-) | 465497_464557 | 10O2-PIBFP-1-R_B03.seq(+) | 422073_422986 | 43424 |
| scaffold169 | 1506375 | 60G20-PIBFP-1-F_A04.seq(+) | 1251898_1252495 | 60G20-PIBFP-1-R_B04.seq(-) | 1298402_1297980 | 46504 |
| scaffold170 | 1573008 | 10G4-PIBFP-1-F_A08.seq(-) | 775851_774899 | 10G4-PIBFP-1-R_B08.seq(+) | 721648_722532 | 54203 |
| scaffold171 | 1082927 | 49P7-PIBFP-1-F_A01.seq(-) | 458730_457781 | 49P7-PIBFP-1-R_B01.seq(+) | 431984_432673 | 26746 |
| scaffold172 | 3048293 | 59B13-PIBRP1F_G02.seq(+) | 1005193_1006063 | 59B13-PIBRP1R_H02.seq(-) | 1122136_1121335 | 116943 |
| scaffold174 | 1614732 | 10O11-PIB-FP_A09.seq(+) | 1493479_1494379 | 10O11-PIB-RP_B09.seq(-) | 1567295_1566415 | 73816 |
| scaffold176 | 857811 | 47K21-PIBRP-1-F_E05.seq(-) | 735000_734212 | 47K21-PIBRP-1-R_F05.seq(+) | 702524_703434 | 32476 |
| scaffold182 | 1637902 | 59P6-PIBFP-1-F_A11.seq(-) | 1223999_1223420 | 59P6-PIBFP-1-R_B11.seq(+) | 1117176_1117981 | 106823 |
| scaffold187 | 954809 | 40L2-PIBFP-1-F_E03.seq(-) | 451384_450531 | 40L2-PIBRP-1-R_F03.seq(+) | 356805_357707 | 94579 |
| scaffold192 | 1171570 | 47J1-PIBFP-1-F_G01.seq(-) | 990911_990434 | 47J1-PIBRP-1-R_H01.seq(+) | 933852_934303 | 57059 |
| scaffold193 | 813494 | 47P18-PIBFP-1-F_A11.seq(-) | 642271_641431 | 47P18-PIBFP-1-R_B11.seq(+) | 600399_601195 | 41872 |
| scaffold194 | 1271777 | 60O11-PIBFP-1-F_A09.seq(-) | 147698_146838 | 60O11-PIBRP-1-R_B09.seq(+) | 49826_50662 | 97872 |
| scaffold196 | 1196508 | 49I1-PIBFP-1F_G01.seq(-) | 571650_570763 | 49I1-PIBFP-1R_H01.seq(+) | 462097_462870 | 109553 |
| scaffold197 | 811274 | 59L14-PIBRP1F_E03.seq(+) | 299007_299920 | 59L14-PIBRP1R_F03.seq(-) | 395779_395057 | 96772 |
| scaffold198 | 846835 | 49M1-PIBFP-1F_C01.seq(+) | 153978_154883 | 49M1-PIBFP-1R_D01.seq(-) | 253508_252658 | 99530 |
| scaffold200 | 777631 | 49E11-PIBFP-1-F_C10.seq(+) | 658587_659135 | 49E11-PIBFP-1-R_D10.seq(-) | 755132_754308 | 96545 |
| scaffold202 | 1094111 | 60P6-PIBFP-1-F_A11.seq(-) | 669154_668544 | 60P6-PIBRP-1-R_B11.seq(+) | 582055_582870 | 87099 |
| scaffold204 | 759221 | 49P10-PIBFP-1-F_A07.seq(-) | 234263_233442 | 49P10-PIBFP-1-R_B07.seq(+) | 122907_123671 | 111356 |
| scaffold209 | 1777546 | 10C5-PIBFP-1-F_E10.seq(+) | 1432350_1433284 | 10C5-PIBFP-1-R_F10.seq(-) | 1467440_1466539 | 35090 |
| scaffold212 | 1313664 | 49K4-PIBFP-1F_E07.seq(+) | 533432_534344 | 49K4-PIBFP-1R_F07.seq(-) | 658406_657502 | 124974 |
| scaffold215 | 1242188 | 5908-PIBRP-1-F_A03.seq(-) | 1142681_1141868 | 5908-PIBRP-1-R_B03.seq(+) | 1071116_1072020 | 71565 |
| scaffold218 | 1025475 | 60E7-PIBFP-1-F_C02.seq(-) | 399030_398096 | 60E7-PIBRP-1-R_D02.seq(+) | 284654_285547 | 114376 |
| scaffold224 | 688637 | 47N15-PIBFP-1-F_C05.seq(+) | 23711_24627 | 47N15-PIBFP-1-R_D05.seq(-) | 76210_75357 | 52499 |
| scaffold226 | 682300 | 49K2-PIBFP-1F_E03.seq(-) | 317722_316828 | 49K2-PIBFP-1R_F03.seq(+) | 210665_211521 | 107057 |
| scaffold227 | 679439 | 59M1-PIBRP-1-F_C01.seq(+) | 398636_399588 | 59M1-PIBRP-1-R_D01.seq(-) | 498203_497305 | 99567 |
| scaffold231 | 726173 | 60D7-PIBFP-1-F_E02.seq(+) | 123953_124902 | 60D7-PIBRP-1-R_F02.seq(-) | 183103_182194 | 59150 |
| scaffold234 | 758216 | 60E16-PIBFP-1-F_C08.seq(-) | 698037_697200 | 60E16-PIBFP-1-R_D08.seq(+) | 610871_611530 | 87166 |
| scaffold250 | 2818770 | 49A7-PIBFP-1-F_G02.seq(+) | 853586_854534 | 49A7-PIBFP-1-R_H02.seq(-) | 957477_956841 | 103891 |
| scaffold253 | 1364888 | 49P8-PIBFP-1-F_A03.seq(-) | 728560_727613 | 49P8-PIBFP-1-R_B03.seq(+) | 603349_603967 | 125211 |
| scaffold257 | 567122 | 47D20-PIBFP-1-F_E04.seq(+) | 61796_62767 | 47D20-PIBFP-1-R_F04.seq(-) | 100068_99568 | 38272 |
| scaffold271 | 896877 | 47F21-PIBFP-1-F_C06.seq(-) | 620542_619610 | 47F21-PIBFP-1-R_D06.seq(+) | 539810_540707 | 80732 |
| scaffold279 | 777631 | 47L17-PIBFP-1-F_E09.seq(-) | 129403_128548 | 47L17-PIBFP-1-R_F09.seq(+) | 101327_102160 | 28076 |
| scaffold280 | 515715 | 59O19-PIBFP-1-F_A01.seq(-) | 253191_252236 | 59O19-PIBRP-1-R_B01.seq(+) | 139953_140717 | 113238 |
| scaffold283 | 511759 | 49M24-PIBFP-1-F_C11.seq(-) | 299860_298995 | 49M24-PIBFP-1-R_D11.seq(+) | 190486_191303 | 109374 |
| scaffold289 | 501926 | 59P13-PIBRP1F_A01.seq(-) | 313042_312256 | 59P13-PIBRP1R_B01.seq(+) | 215141_215965 | 97901 |
| scaffold295 | 476693 | 59I24-PIBFP-1-F_G11.seq(+) | 355635_356511 | 59I24-PIBRP-1-R_H11.seq(-) | 449905_449065 | 94270 |
| scaffold318 | 950566 | 59O22-PIBFP-1-F_A07.seq(-) | 171780_170843 | 59O22-PIBRP-1-R_B07.seq(+) | 67725_68622 | 104055 |
| scaffold351 | 400300 | 59F23-PIBRP1-F_C10.seq(+) | 128416_129298 | 59F23-PIBRP1-R_D10.seq(-) | 255527_254686 | 127111 |
| scaffold365 | 321214 | 49O20-PIBFP-1-F_A03.seq(+) | 188745_189370 | 49O20-PIBFP-1-R_B03.seq(-) | 289080_288205 | 100335 |
| scaffold372 | 560011 | 60I15-PIBFP-1-F_G05.seq(+) | 170336_171134 | 60I15-PIBFP-1-R_H05.seq(-) | 265566_264819 | 95230 |
| scaffold411 | 315327 | 40D19-PIBRP1-F_E02.seq(-) | 170911_169959 | 40D19-PIBRP1-R_F02.seq(+) | 44367_45151 | 126544 |
| scaffold436 | 533849 | 10M7-PIB-FP_C01.seq(-) | 122196_121303 | 10M7-PIB-RP_D01.seq(+) | 49386_50188 | 72810 |
| scaffold477 | 311592 | 60I1-PIBFP-1-F_G01.seq(+) | 26337_27162 | 60I1-PIBRP-1-R_H01.seq(-) | 131271_130491 | 104934 |
| scaffold479 | 307236 | 59B6-PIBFP-1-F_G12.seq(+) | 121856_122628 | 59B6-PIBFP-1-R_H12.seq(-) | 230715_229934 | 108859 |
| scaffold497 | 210723 | 60M19-PIBFP-1-F_C01.seq(+) | 12400_12832 | 60M19-PIBFP-1-R_D01.seq(-) | 105575_104673 | 93175 |

**Table S8.** **Information of FISH probes synthesized by PCR.**

| Scaffold_ID | Probe name | Start  (on Scaffold) | End  (on Scaffold) | Probe length (bp) |
| --- | --- | --- | --- | --- |
| Scaffold1 | Sq1 | 13788 | 38838 | 25050 |
| Scaffold8 | Sq8 | 7397 | 44317 | 36920 |
| Scaffold10 | Sq10 | 222416 | 253358 | 30942 |
| Scaffold15 | Sq15 | 16596 | 41524 | 24928 |
| Scaffold22 | Sq22 | 20730 | 49241 | 28511 |
| Scaffold35 | Sq35 | 1572872 | 1599579 | 26707 |
| Scaffold39 | Sq39 | 35362 | 56948 | 21586 |
| Scaffold47 | Sq47 | 595074 | 629376 | 34302 |
| Scaffold48 | Sq48 | 374869 | 408753 | 33884 |
| Scaffold49 | Sq49 | 2421817 | 2456049 | 34232 |
| Scaffold53 | Sq53 | 186911 | 214888 | 27977 |
| Scaffold58 | Sq58 | 2453931 | 2487313 | 33382 |
| Scaffold65 | Sq65 | 3981255 | 4015277 | 34022 |
| Scaffold67 | Sq67 | 151624 | 178918 | 27294 |
| Scaffold73 | Sq73 | 1900080 | 1927426 | 27346 |
| Scaffold75 | Sq75 | 386503 | 424799 | 38296 |
| Scaffold76 | Sq76 | 365348 | 391589 | 26241 |
| Scaffold77 | Sq77 | 99469 | 126733 | 27264 |
| Scaffold79 | Sq79 | 852498 | 879688 | 27190 |
| Scaffold84 | Sq84 | 151133 | 178129 | 26996 |
| Scaffold88 | Sq88 | 717213 | 746654 | 29441 |
| Scaffold91 | Sq91 | 502385 | 529871 | 27486 |
| Scaffold95 | Sq95 | 990706 | 1017785 | 27079 |
| Scaffold104 | Sq104 | 509840 | 536840 | 27000 |
| Scaffold106 | Sq106 | 2256192 | 2282836 | 26644 |
| Scaffold112 | Sq112 | 425874 | 452841 | 26967 |
| Scaffold121 | Sq121 | 66953 | 93805 | 26852 |
| Scaffold127 | Sq127 | 1622094 | 1648585 | 26491 |
| Scaffold134 | Sq134 | 1713447 | 1739869 | 26422 |
| Scaffold135 | Sq135 | 631840 | 669145 | 37305 |
| Scaffold158 | Sq158 | 317942 | 354570 | 36628 |
| Scaffold181 | Sq181 | 41750 | 75750 | 34000 |
| Scaffold186 | Sq186 | 1552142 | 1579263 | 27121 |
| Scaffold221 | Sq221 | 442834 | 479405 | 36571 |
| Scaffold284 | Sq284 | 323108 | 350147 | 27039 |
| Scaffold298 | Sq298 | 331617 | 366124 | 34507 |
| Scaffold369 | Sq369 | 224392 | 261298 | 36906 |
| Scaffold394 | Sq394 | 566295 | 593137 | 26842 |

The orientation of the scaffolds is from 5’-end to 3’-end. Each probe contains 8-15 PCR fragments with length of 1.5-2.5Kb, which align on scaffold with a total length of 20-30 Kb.
