# Supplementary material for: Chromosome-scale assembly of the Monopterus genome
Source: Gigascience. 2018 Apr 24;7(5):giy046. doi: 10.1093/gigascience/giy046 (PMC5946948; doi:10.1093/gigascience/giy046)
Supplement: Response_to_Reviewer_Comments_Original_Submission.pdf [file giy046_response_to_reviewer_comments_original_submission.pdf]

Author's Response To Reviewer Comments

Close

Point-to-point responses to reviewers

To Reviewer 1:

The manuscript contains valuable and interesting data/results, but the presentation lacks clarity and soundness in many places as well as does not meet scientific standards in a few cases.

Responses: Many thanks for your critical comments. We have revised the whole manuscript following your suggestions and scientific standards. We have also formatted the version as “Data Note”, not as a "Research Article". Following suggestions of Editor Hans Zauner (omit the second part of the paper on sex transition using RNAseq data, only include first part, genome assembly). As in the part two, the reviewer 2 suggested to repeat RNAseq data, however the biological replications need gonad samples which can only be obtained in reproductive season (June- Sept), this work cannot be performed in several months. Nevertheless, final paper in genome assembly for the Data Note is complete and meets standards of the Gigascience.

MAJOR COMPULSORY REVISIONS

GENERAL

The selection of cutoffs 1/2 is not clear and seems to be highly subjective. Essential data (eg. map positions of ridges and IRSEs) is missing. Terminology centrally to the manuscript is not clear (eg. "interconvertible", "interconvertible region", "interconvertible domain" and "interconvertible manner"). lines 47/372/418 Clear overstatements: "we discovered a co-regulation mechanism", "found a co-regulation mechanism" and "showed a co-regulation mechanism". 306-310 The features (GWPs) in this correlation analysis are mutually (inversely) dependent. Moreover it is not clear whether the given p-value has been corrected for multiple testing. 314-319 The paragraph is conceptually/semantically not clear to me. What means "distribution pattern can be described in interconvertible regions", "IRSEs explain" and "emerge in an alternating, mutually interconvertible manner along the chromosome"? Fig8D and the reference therein to Fig6 are unclear. 321-331 It is not clear (neither from the Methods) how IRSEs are being defined and mapped. Therefore the presented data are not traceable. What means "suggesting an obvious association" in the Results. The IDSE definition and the concept as well as Fig9 are also not clear hand should have in no way been placed in the Results.

Responses: The second part has been deleted as stated above.

MINOR ESSENTIAL COMMENTS

34/67

No data are presented to support "enormous/high" medical value of the fish. The reference to a medieval writing indicates at best a "potential" medical value.

Responses: Thanks, we have modified them as “potential medical value”. (line 36/69 in the new version)

44
The terminology "types of genes" is misleading.
48/113 "IRSE" vs. "IDSE"?

Responses: The second part has been deleted as stated above.

91
"NGS" means not necessarily "short length of the reads". This refers only to 2nd generation technologies.

Responses: “NGS” has been replaced by “the second generation sequencing technologies”. (line 91 in the new version)

107
What are "complete genetic maps"?

Responses: "complete genetic maps" has been replaced by“high-density genetic map”. (line 109/110 in the new version)

110
Wording "chromosome-level assembly with 81.3% coverage of the sequenced genome" is not scientifically sound.
Responses: The sentence has been re-written as: “chromosome-level assembly covering 81.3% of the sequences assembled in scaffolds”. (line 114 in the new version)

113
"reasonably explains" is not scientifically sound.

Responses: The section has been deleted.

122
How "78.6 GB sequence dataset with a sequencing depth of 97-fold" was calculated?

Responses: We have added the sequence data description including the related methods (line 128 in the new version).
The genome size was estimated from k-mer analysis (806 Mb). The sequencing depth can be calculated from the total number of sequencing bases (78.6 GB) and the genome size (806 Mb) as 78.6 GB/806 Mb=97.

128

Not "mapping to the genome" but mapping to the reference sequence of the genome.

Responses: Thanks, we have corrected it as “mapping to the reference sequence of the genome”. (line 163 in the new version)

135
"DNAs"?

Responses: DNA transposons, TEs are assigned according to their mechanism of transposition, which can be described as either copy and paste (retrotransposons) or cut and paste (DNA transposons). Retrotransposons are commonly grouped into three main orders: LTRs, SINEs and LINEs. DNAs refer to the DNA transposons. We have replaced the “DNAs” with “DNA transposons”. (line 191 in the new version)

136
"acted in concert"?

Responses: We have corrected it as “which is involved in guardians for genome defense and germline stability via piRNAs”. (line 193 in the new version)

140
"the database"?

Responses: the local Monopterus genome database, we have modified it. (line 199 in the new version)

156-176
What means "group", "efficient combinations", "landmarks or the sequences", "order of another scaffold", "each scaffold"?

Responses: We have modified them as:
“linkage group” refers to the sequence sets of a chromosome.
“efficient combinations” replaced by “probes combinations” (line 241 in the new version)
“landmarks or the sequences” replaced by “each with a molecular landmark” (line 243 in the new version) “The order of another scaffold” replaced by “The location of a new scaffold” (line 255 in the new version)
“each scaffold” replaced by “all scaffolds on chromosomes”. (line 259 in the new version)

180
Rephrase "A scaffold was mapped on each chromosome by FISH, which was used as a landmark" and describe clearer how the landmark scaffolds were selected.

Responses: We have modified it and added a paragraph to describe the landmark scaffolds.
Line 236-245:"We first prepared probes of BACs from sequenced clones and PCR fragment

pools (8-15 sequences and covers a total length of 20-30 kb on a scaffold) representing scaffolds for chromosome FISH, and performed synteny analysis of these scaffolds by comparing with the fish species (medaka, sticklebacks, Tetraodon and Monopterus). Second, from the synteny information of the homologous sequences of these scaffolds in the three fish species, probe combination mapping was used to determine 12 linkage groups, corresponding to 12 chromosomes, each with a molecular landmark (Figure 1A). Briefly, group A and B were first discriminated by two unlinked scaffolds labelled with two different colours. If another scaffold was unlinked to the previous two scaffolds, the third scaffold was identified as a marker of group C. "

183-194

Where do the numbers 186/108142 come from and how they relate to each other? The sentence "142 of the scaffolds were further assembled into 12 chromosomes" is misleading. What means "issue should be further corrected"?

Responses: We have re-written the sentences clearly in the section "De novo chromosome assembly by Cafs-strategy."

186 refers to the number of the scaffolds mapped by FISH;

These 186 scaffolds can be divided into two categories according to the results of the synteny analysis:

108 scaffolds whose homologous sequences had the consistent synteny in at least two of the three closely related fish species, while the rest 76 scaffolds didn't show consistent synteny.

For the 108 scaffolds whose location could be predicted by the synteny analysis, 99 of which were confirmed by FISH, while the rest 9 whose FISH results were inconsistent with their prediction, which may be the result of the rearrangement of the genome in the genome evolution.

Thus, the accuracy of the synteny prediction is 99/108=92%.

142 refer to the number of the scaffolds mapped by synteny prediction only, without FISH confirmation. FISH work remains to be further performed in the future. (line 285-298 in the new version)

196/199

" We finally integrated 328 scaffolds into 12 chromosomes" is lab jargon. You assembled 328 scaffolds into reference sequences for 12 chromosomes. The same refers to "sequenced genome of 683 Mb with 97.6X". You sequenced a genome and assembled a reference sequence of 683 Mb that is (most likely - please provide the overall length of assembled reads eg. as supplement) 97.6x covered by aligned reads.

Responses: We have re-described the sentences as,"We finally assembled 328 scaffolds into reference sequences for 12 chromosomes",

"accounting for 81.3% of the sequenced genome of 683 Mb with 97.6X" was replaced by "accounting for 81.3% of the sequences assembled in scaffolds (689.5 Mb)".

The overall length of assembled reads was shown in table 1.

(line 300/303 in the new version)

203/207

What mean "efficient" and "most attractive"?

Responses: We have deleted the words and modified. (line 207 in the new version)

213-217

What means "deep transcriptome sequencing"? It must be explained at least in Methods. What means "distribution patterns of genes on chromosomes in accordance with their expression profiles"? The statement "significant positive correlation between the gene density and expression" should be better explained/supported in the main text. The terms "gene density" and "expression" are not introduced Figures show "Gene number". The reference to Fig4B is not helpful. R values should be given together with the p-value in the main text. For any p-values the respective test should be indicated.

Responses: The second part has been deleted as stated above.

227

The statement "distribution pattern of the gene density was consistent with the corresponding GC content" is not supported by data.

Responses: In the previous version, FigS9B showed the spearman correlation test for GC content and gene density, and the figure has been changed into Fig.S9 in the new version. (line 324 in the new version)

234-239

Define "consecutive window numbers" unambiguously. Not "distribution pattern of the ridges" but numbers of ridges have been defined. What means "significantly low", "(ridge numbers in random ≥ observations", "highly significant difference in ridges with high gene density"?

Responses: We have revised them as:

“consecutive window numbers” means “the numbers of consecutive moving windows in which each gene numbers have a lower limit of cutoff 1”. We have added the detailed description in brackets.

“distribution pattern of the ridges” is replaced by “the numbers of the ridges”.

“significantly low” is replaced by “very low”.

“ridge numbers in random ≥ observations” is replaced by “ridges numbers under a random permutation ≥ ridges numbers in the Monopterus genome”.

“showed a highly significant difference in ridges with high gene density along chromosomes in comparison with random permutations of gene positions” is replaced by “the results showed that there were significant differences in ridges numbers between the Monopterus genome and random permutations of gene positions”. (line 341-345 in the new version)

245

The statement "These analyses suggest that the ridge pattern on the chromosomes probably represents a higher-order structure in the genome" belongs to Discussion not to Results.

Responses: We have put the sentence in the last section. (line 351 in the new version)

250/739
Clarify the meaning of "key gene types during gonad transition" and "gene regulation pathways in gonad transition".
255-259
What means "clearly"? Please provide gene numbers/fractions for each type as suppl table. What means "the GO terms in the biological process category were over-represented in all types"?
281-283
What means "high degree" when comparing types V and II as well as "clustered on chromosomes in a large-scale"?
286-287
What means "each type of gene" and "continuous degree of gene clustering"?
292-296
How the p-values were obtained (corrected for multiple testing) and how they relate to "*/**" in Fig7B. 336 What means "complete genome information" - a complete and error free as possible reference sequence?
337-342
Please provide a justification/reference for the statement.
343
What means "the other 22 species"?
345
Please provide data for "efficient and cost effective".
348 The statement "81.3% coverage of the sequenced genome is most likely an overestimation.
Responses: The part and discussion sections have been deleted as the data note format.
353
Provide data for "improve the understanding of the mechanisms of genome". 355-356 What means "favoured genes" and "QTLs of economic importance".
372
Clear overstatement: "found a co-regulation mechanism". 375-377
Clarify what means "cluster" in this context and whether in your concept an IDSE is formed by a subset of genes of a "ridge" 383 What means "IRSE-o should occupy a different topological domain from the IRSE-t".

394
Provide data for "IDSEs are closely associated with sex determination".

Responses: The second part has been deleted as stated above.

428
(line 411 in the new version) To my understanding no "animal experiments" have been

performed.

Responses: Yes, we have performed animal experiments, kidney tissues were dissected, which has been added in the line 265 in the new version.

585
the statement "three tissues (ovary, ovotestis, and testis)" is not corre ct. How many biological replicates were identified? 596-597
Clarify whether "RPKM<0.1" refers to one or both analyzed states. What means "FDR (false discovery rate) < 0.05 (p-value<0.01)"?
741-745 The statement "Differential expression is identified by fold change of RPKM (≥2)" contradicts those in Methods. What means "p-value = 0.041 (DAVID, version 6.7). Fisher's exact test (FDR = 0.05) was used to assess the enrichment"?
752-764
The figure as such does not show "Clustering patterns". What are "typical genes" and "intensity of clusters"? "and at least" should read as "in at least"?
766-778 Phrasing "consecutive gene clusters of co-regulated genes" is misleading and should read as "clusters of consecutive co-regulated genes". Significance is not explained (test, thresholds, multiple test correction). 786 What means "alternating sine curves"?
793
Are the p-values corrected for multiple testing?
803-810 The legend does not sufficiently explain what is shown in the different panels.

Responses: The second part has been deleted as stated above.

Tab 1
What "*" refers to? What means "final contig length after filling intra-scaffold gaps"? Does the overall scaffold length include Ns?

Responses: We have modified the description of Table 1. The final contig size are the size after filling gaps. "contig length" is replaced by "contig size".

Tab2
"Chromosome length" is incorrect/misleading.

Responses: "Chromosome length" is replaced by "Chromosome size".

Fig5B
Cyp51 in the left panel is shaded according to log2(FC)=2 (ie. FC=4) but the right panel shows a FC<3.
FigS12
What means "gene numbers in each catalog"?
FigS13 The title does not cover type IX.

Responses: The second part has been deleted as stated above.

DISCRETIONARY COMMENTS

39

"Cafs" is not clear here.

Responses: "Cafs" was the abbreviation of “de novo chromosome assembly by FISH walking assisted by conserved synteny”. The detailed strategy of Cafs were in the section “De novo chromosome assembly by Cafs-strategy”. (line 39 in the new version)

87-90

Mixing "genetic" and "physical" maps is confusing for a non-geneticist reader.

Responses: Yes, our statement is not clear and accurate. Both genetic map and physical map can assist chromosome assembly, but they are different. We have modified them by using the word in different sentences. (line 89-92 in the new version)

98

"BAC/fosmid paired end sequencing from large-insert libraries" is a tautology.

Responses: Yes, we have described it as “BAC/fosmid paired end sequencing”. (line 101 in the new version)

106

"map on chromosomes" map of chromosomes?

Responses: We have modified it. (line 109 in the new version)

128

Not "Sanger sequence technology" but "Sanger sequencing technology".

Responses: Thanks, we have modified it. (line 164 in the new version)

146

Replace "Chromosome-level assembly strategy without a genetic map" by "Chromosome assembly by FISH walking assisted by conserved synteny - strategy outline", outline the strategy in this section and provide the details for the actual project in the next section.

Responses: Thanks, we have modified it, and provide the details for the actual project in the section following the Data Note format. (line 230 in the new version)

346

Do "gene functions" have a "position in the genome"?

Responses: The second part has been deleted as stated above.

376
Introduce abbreviations only once in the main text.

Responses: We have provided the abbreviations section (line 385 in the new version).

[19] is rather outdated.
TabS9 is incomplete.

Responses: The second part has been deleted as stated above.

To Reviewer #2:

This manuscript describes both the genome assembly of the swamp eel, and some transcriptomics experiments relating to its sex-reversal physiology.

The genome assembly process follows a straightforward short-read shotgun recipe, but is supplemented by in situ hybridization of scaffold probes to arrive at a chromosome-level assembly. This is presented as a novel strategy, which I am uncertain is entirely justified. It is, however, a worthwhile effort. The supposed novelty is entirely related to the pre-selection of co-hybridization probes based on synteny information. In general, I would be very cautious about using comparative evidence to finish a chromosome-level assembly, as any subsequent comparative genomics studies run the risk of circular reasoning. In this case, it appears the comparative evidence is mostly only used as an initial screening, and it does not directly inform the assembly process - but please see my issue 6 below.

Responses: Many thanks for your critical comments and kind suggestions. Yes, the cafs consists of several methods which are not novel. Nevertheless, their combinations, especially synteny and FISHing were adopted, are certainly efficient for chromosome-level assembly. It is indeed feasible, assembling 81.3% of scaffold sequences, and the accuracy is high. Synteny analysis provided an initial screening, are cost-less and take less time for final FISHing assembly process. Final assembly have been confirmed by FISHing, except approximately 8% (~11) of the scaffolds could not be resolved by the conserved synteny prediction due to possible rearrangements in the Monopterus lineage. This issue should be further corrected in future experiments.

Several issues about the assembly part I would ask the authors to address:
1. Is it correct that the assembly is based on multiple discrete scaffolding procedures (SOAPdenovo, l. 456, and SSPACE, l. 459) using the same data? Does this not increase the risk of artifacts?

Responses: The Monopterus genome was assembled by constructing 170bp, 500bp, 800bp, 2kb, 5kb, 10kb, 20kb and 40kb insert size genome library for sequencing respectively. The sequencing data derived from 2kb, 5kb, 10kb and 20kb insert size library was used to assemble scaffolds by SOAPdenovo, and the data derived from 40kb insert size library was used to assemble scaffolds by the SSPACE. The original description was not clear and we have modified the statement in the new version. (line140-148 in the new version)

2.Only ~79% of the expected genome size is captured in contigs (634.7 Mbp of 806 Mbp). Therefore, presumably, these contigs contain abundant collapsed repeats, affecting an analysis of repetitive content (l. 131-138, fig S4) Repeat abundance will likely be underestimated. (line 188-195 in the new version).

Responses: The genome size is 806Mb estimated by K-mer, but only 634.7 Mb was captured in contigs based on the data of second generation sequencing. This most likely results from a lot of repetitive sequences in the genome. The incomplete assemble is the limitation of the second-generation sequencing technology. The repetitive sequences we analyzed are based on the assembled genome. Thus, as you said, repeat sequences would be underestimated. We have discussed the fact in the version.

3.Line 136: please rephrase 'they acted', as no evidence on piRNAs is presented. （line 194 in the new version）

Responses: Several papers have reported that the piRNAs derived from LINE are very closely related with genome stability. We have just discussed the fact that LINEs was greater than that found in other teleost fish (2-5%), which were involved in guardians for genome defense and germline stability via Piwi-interacting small RNAs (piRNAs) [23].

4.No completeness analysis of the assembly/gene prediction is presented. Please include a BUSCO analysis of the predicted gene set.

Responses: We have added BUSCO (line 167-173 in the new version) analysis to evaluate the completeness of the gene set.

5.Line 142 /fig S5: 'The gene sizes were similar to those of other teleost fish'. In fact, the figure shows that the Monopterus gene set contains many more short genes and CDSs than other teleosts. Exon lengths are comparable. This suggests truncated genes or gene predictions. (line 214 in the new version)

Responses: BUSCO analysis showed that there are some numbers of fragmented and missing BUSCOs, but very low.

6.FISH walking (l. 180-203): Is it correct that no FISH evidence is used to assign 142 scaffolds to chromosomes (l. 191)? How will placing these scaffolds using comparative evidence only affect comparative analyses? (line 263-307 in the new version)

Responses: the data of 142 scaffolds were from comparative genomics analysis, not from FISHing experiments. We have discussed this issue, pointed out the possible error rates and problems. (line 295-298 in the new version)

7.Figure 3B: I do not see the added value of the visualization of gene prediction for this single scaffold. Similarly for figure 4C.

Responses: We have added the start location information of genes on the scaffold 72 in Figure 3B, and added the regions start and end location on the chromosome 10 in figure 4C (figure 4B of the new version).

8.Figure 3A and 4B: The chromosome sizes here add up to 555 Mbp, which is also the size of scaffolds assigned to chromosomes (l. 198) but much less than the expected genome size of 806 Mbp. If these sizes are entirely based on placed scaffolds, why do these illustrations still contain gaps?

Responses: For Figure 3A, the previous scale was really problematic. So we have changed the scale. We mapped the probes based on the ratio of the distance of the probe to centromere to the total length of the chromosome. Thus, we marked the scale with the ratio.
The Figure 4B (Figure 4A in the new version) was confused because of the legend for this figure was not clear. When we packaged the scaffolds into chromosomes, the gap between every two adjacent scaffolds was filled with 50 kb of Ns. Our analysis of the GC content and the gene density were based on this packaged chromosomes, but not the sequences of the outermost scaffold location. Therefore, we have added a new physical map to the Circos plot, which corresponds to the analysis of GC content and gene density.

9.Related: I assume the coloured block in the outer ring of fig 4B are placed scaffolds, and the white spaces gaps. How can these gaps then contain genes, have a GC content, and gene expression (inner rings)?

Responses: Please see the point 8 above.

The second part of the paper addresses sex transition, based on RNA-seq data. I have not reviewed the downstream analyses and conclusions in detail, as I believe the initial RNA-seq data is both insufficient and not analyzed appropriately:
10. There is no description of the RNA-seq raw data. How many reads per sample, etc.?
11. There is no mention of biological replication, therefore the entire analysis appears to be based on one sample per developmental stage - insufficient to draw strong conclusions from.
12. Differential expression analysis is not described in sufficient detail (l. 594-598). These do not appear to be industry-standard statistical analyses. In any case, without replication, how does one test for statistical significance of differential expression?
13. RPKM normalization is usually deprecated, unless it can be argued that it is suitable. In this case, comparing the tissues requires major assumptions anyway, as they do not appear to be intrinsically comparable. I have attached density plots of the gene expression values in the three cases. This is after normalization: with successful normalization, the plots would align. In this case, it is far from clear this is even possible. I do not see how in this case, expression in testis

can be related to the other samples. My quick scatterplots of gene expression show a similar picture.
14. I therefore do not see how these data can be used to reliably infer the patterns shown in fig 5A. (Related, l. 252: these classes/'types' are not differentially expressed themselves, they correspond to genes that are 'differentially expressed' in any one sample compared to the other two). The important/large classes IV and V are probably mostly populated by genes affected by noise/the effects I describe in issue 13.
15. Most statements about gene expression or its classification are based on very low expression, which especially in this case (no replication, poor normalization) is not reliable. For instance: fig S9 correlations (or are these log-values?), dhcr24 (l. 265, fig 5B), fgf8b (fig 6B).

Responses: As for the second part, we have formatted the version as “Data Note”, not as a "Research Article". Following suggestions of Editor Hans Zauner (omit the second part of the paper on sex transition using RNAseq data, only include first part, genome assembly). As in the part two, you suggested to repeat RNAseq data, however the biological replications need gonad samples which can only be obtained in reproductive season (June- Sept), thus this work cannot be performed in several months, although re-analysis the RNAseq data by credible FPKM not previous RPKM normalization show the RNAseq are correct. Nevertheless, final paper in genome assembly for the Data Note is complete and meets standards of the Gigascience.

Close
