# Supplementary material for: Chromosome-scale assembly of the Monopterus genome
Source: Gigascience. 2018 Apr 24;7(5):giy046. doi: 10.1093/gigascience/giy046 (PMC5946948; doi:10.1093/gigascience/giy046)
Supplement: Response_to_Reviewer_Comments_Revision_1.pdf [file giy046_response_to_reviewer_comments_revision_1.pdf]

Author's Response To Reviewer Comments

Close

To Reviewer #2:

The authors have improved the manuscript considerably. I think it was wise to omit the transcriptomics study and focus on the genome assembly, which is quite complex already.

1. Even though the RNA-seq study has been left out, I would like to suggest paying close attention to the implicit assumptions usually present in RNA-seq analysis packages. In this case, comparing between very different samples (my previous issue 12) will be challenging, and switching to a different normalization method ('re-analysis the RNAseq data by credible FPKM … normalization show the RNAseq are correct') probably does not address this problem.

Response:
Thanks for your suggestions. We will get new samples in reproductive season and sequence and analyze more samples using appropriate algorithms with biological replicates.

Many of the issues I previously raised on the first part have been addressed, but there are a few I would like to revisit:

2.Only ~79% of the expected genome size is captured in contigs (634.7 Mbp of 806 Mbp). Therefore, presumably, these contigs contain abundant collapsed repeats, affecting an analysis of repetitive content (…) Repeat abundance will likely be underestimated. (line 188-195 in the new version).
Responses: The genome size is 806Mb estimated by K-mer, but only 634.7 Mb was captured in contigs based on the data of second generation sequencing. This most likely results from a lot of repetitive sequences in the genome. The incomplete assemble is the limitation of the second-generation sequencing technology. The repetitive sequences we analyzed are based on the assembled genome. Thus, as you said, repeat sequences would be underestimated. We have discussed the fact in the version.

The underestimation of repeat abundance is not discussed in the new version. In addition, the BUSCO analysis suggests the 634.7 Mbp genome assembly to be very complete (line 167-173). This suggests the rest of the genome to consist of non-coding DNA, possibly containing many repeats. Unless the 806 Mbp k-mer-based estimate is an overestimate?

Response: (lines195-199 in the new version)
Thanks. We have added a paragraph of discussion on page 7. It should be pointed out that only ~79% of the expected genome size was captured in contigs (634.7 Mb of 806 Mb), and the BUSCO analysis showed that the 634.7 Mb genome assembly was complete. These results suggest that the unassembled genome probably consists of non-coding DNA, possibly contains many repeats. Thus, the repeat abundance would likely be underestimated. The third-generation sequencing will provide a more complete assembly. There would be also a few of errors in k-mer-based estimate of genome size, but it is accepted widely.

3.Line 136: please rephrase 'they acted', as no evidence on piRNAs is presented.
（line 194 in the new version）
Responses: Several papers have reported that the piRNAs derived from LINE are very closely related with genome stability. We have just discussed the fact that LINEs was greater than that found in other teleost fish (2-5%), which were involved in guardians for genome defense and germline stability via Piwi-interacting small RNAs (piRNAs) [23].

4. I did not want to dispute the biology here (but see below), but just wanted to point out that this is a speculative point of discussion. The current study presents no evidence on piRNAs, therefore the phrasing should be adjusted. The current 'LINEs … were involved in … genome defense … via Piwi-interacting small RNAs' (l193-194) suggests the LINEs were actually studied. So I would suggest rephrasing 'were involved' to 'might be involved'.
Regarding the biology - doesn't a high LINE content suggest genome defense (by piRNA) has been ineffective?

Response to 3 and 4: (Lines 192-193 in the new version)
Thanks. You are right, it is a speculative point of discussion, and we have no evidence. A high LINE content suggests that genome defense (by piRNA) has been ineffective. Our study does not involve any piRNA work. Accordingly, we have revised the sentence as “The percent of LINEs was greater than that found in other teleost fish (2-5%), which might be associated with genome instability[23].”

5.Line 142 /fig S5: 'The gene sizes were similar to those of other teleost fish'. In fact, the figure shows that the Monopterus gene set contains many more short genes and CDSs than other teleosts. Exon lengths are comparable. This suggests truncated genes or gene predictions.
Responses: BUSCO analysis showed that there are some numbers of fragmented and missing BUSCOs, but very low.
Indeed, the BUSCO analysis suggests the assembly is complete. However, looking at figure S5, CDS and mRNA lengths are noticeably shorter in Monopterus. This is not reflected in the averages (table S5).

Response: (Lines 219 in the new version) (Figure S5/table S5)
The gene size estimate is just in an average, so we have changed the sentence using “average gene sizes” to “The average gene sizes were similar to…”
The trend of average value in Table S5 is really reflected by the curves in figure S5, but it is not in accordance with the location of the peak of the frequency, but another feature: the true relationship between them is that the smaller the area of the small fragment in the graph, the larger the average value may be. Taking the distribution of mRNA length as an example, since the y-axis represents the percentage, the areas under the curve of different species are equal. The smaller the area corresponding to the shorter segments is, the larger the area corresponding to the longer segments is, and the higher of the ratio of the longer segments is. In another word, although the largest proportion of the Monopterus mRNA are the shorter fragments, a considerable proportion of the longer fragments truly exist which couldn’t be detected in the graph but affect the average value. This phenomenon is more evident for the zebrafish data.

6.FISH walking (l. 180-203): Is it correct that no FISH evidence is used to assign 142 scaffolds to chromosomes (l. 191)? How will placing these scaffolds using comparative evidence only affect comparative analyses? (line 263-307 in the new version)
Responses: the data of 142 scaffolds were from comparative genomics analysis, not from FISHing experiments. We have discussed this issue, pointed out the possible error rates and problems. (line 295-298 in the new version)
The discussion of this issue is minimal (l. 299). The way I read it, the chromosome-scale assembly may contain large structural errors: 8% of FISH-based placements are inconsistent with synteny (l. 294), therefore conserved synteny should be used with a lot of caution. Yet a further 100 Mb of sequence is placed based on synteny only (l. 302). Some scaffolds are apparently left out (l. 297), but the numbers do not add up? 186 + 142 = 328 scaffolds have been placed (l. 301), but of the 142 some (approximately! 11, l. 297) have been left out?

Response: (Lines 301-309 in the new version)
The 142 scaffolds were predicted by synteny analysis. According to the results of the FISH experiment and collinearity analysis, approximately 8% (~11) of the 142 scaffolds could not be resolved by the conserved synteny prediction due to possible rearrangements in the Monopterus lineage. Accurate mapping of the 142 scaffolds remains to be confirmed by the third-generation sequencing. We have added a paragraph of discussion concerning this.

7.Related: I assume the coloured block in the outer ring of fig 4B are placed scaffolds, and the white spaces gaps. How can these gaps then contain genes, have a GC content, and gene expression (inner rings)?
Responses: Please see the point 8 above.

The outer ring of figure 4A has a different scale than all the inner content, which is confusing as this is a non-standard way of presenting these data. The gaps (white space) in the outer ring are not represented in the inner graphs. At least please note this in the figure legend.

Response: (Figure 4A in the new version)
The outer ring of previous figure 4A is actually same as figure 3A, so we have deleted in the new figure4 for avoiding of confusing with the other rings. For the new figure 4, we have adopted a set of same position for the assembled sequences (gaps were filled with 50 kb of Ns) for sliding window analysis, so the Circos positions are consistent and clear for all rings. We have also added a stricter set of parameters for the association analysis of GC with gene density to highlight the regions with higher correlation between GC content and gene density. Locations of the regions with higher correlation between GC content and gene density ($R > 0.95$, p-value < 0.01) have been added into the Circos using sliding window analysis.

Some comments on the current version:

L99-100: 'high costs of sequencing', is this still the case?

Response: (Lines 99-100 in the old version but deleted in the new version)
We mean a relative high cost of sequencing compared to the second-generation sequencing

technologies. But nowadays the price is not a problem for sequencing, so we have deleted the sentences in the revised manuscript.

L103-104: 'assemble chromosomes, which can produce ultra-long scaffolds': I think this should be the other way around (e.g. assembled ultra-long scaffolds can lead to a chromosome-scale assembly)?

Responses: (Lines 102-103 in the new version)
Thank you for your suggestion. The sentence in line 102-103 “have been used to assemble chromosomes, which can produce ultra-long scaffolds” has been replaced by “have been used to assemble ultra-long scaffolds which can lead to a chromosome-scale assembly”.

L119: Does using two individuals lead to assembly problems (due to heterozygosity)? Presumably not, based on the k-mer analyses (figure S2)?

Responses: (Lines 118 in the new version)
The reads produced from the small-size fractionated genomic library to construct contigs by the second-generation sequencing technology were derived from a single male Monopterus, and the data used for k-mer analysis were also derived from this male Monopterus. The heterozygosity revealed from the K-mer analysis reflects the inner heterozygosity of the homologous chromosomes in an individual. For getting a consistent, high quality assembly, we have used two individuals for complementary assembling of longer scaffolds, which is successful on evidence in BGI.

L153/163: These percentages (91.06%/99.7%) appear to refer to the same analysis? I think the latter refers to the 99.69% in line 153, but those are not 'reads mapping'.

Responses: (Lines 161-162 in the new version)
Yes, 99.7% and 99.69% refer to the same analysis. But, 99.69% refers to “coverage”, while 91.06% refers to “reads mapping”. Therefore, we have revised the original sentences in line 161-162 as "Assembly accuracy was further demonstrated by 91.06 % reads mapping (99.69% coverage) to the reference sequences of the genome".

L235: 'closely related species' -> 'distantly related species', they are in different orders.

Responses: (Lines 239 in the new version)
Thank you for your suggestion. We have replaced the sentence “closely related fish species” with “distantly related fish species”.

L325: 'The distribution pattern of the gene density was consistent with the corresponding GC content along the chromosomes'. This refers to the correlation between these two measures. However, figure S9 does not show a clear correlation. In fact, I expect the entire positive correlation to be caused by a few outliers (high GC, high gene content, probably on chromosome 12, see figure 4A). The overall trend is not obvious.

Responses: (Lines 336-343 in the new version)
For the new figure 4, we have adopted slide window analysis, so the Circos positions are consistent and clear for all rings. We have added a stricter set of parameters for the association analysis of GC with gene density to highlight the regions with higher correlation between GC content and gene density. Locations of the regions with higher correlation between GC content and gene density ($R > 0.95$, p-value < 0.01) have been added into the Circos using the slide window analysis. The results show, as you expect, that some regions with very high correlation coefficients ($R > 0.95$, p-value <0.01) are detected in the genome, for example, four regions on chromosome 12 (Figure 4A).

Close
