# Supplementary material for: Chromosome-scale assembly of the Monopterus genome
Source: Gigascience. 2018 Apr 24;7(5):giy046. doi: 10.1093/gigascience/giy046 (PMC5946948; doi:10.1093/gigascience/giy046)
Supplement: Response_to_Reviewer_Comments_Revision_2.pdf [file giy046_response_to_reviewer_comments_revision_2.pdf]

# Author's Response To Reviewer Comments

Close

Point-to-point responses to Reviewer:

Reviewer #2:
I thank the authors for improving the manuscript's clarity to the point where it is in principle publishable. Please find below a few final suggestions for minor additional clarifications.

On my issue of using multiple eels for sequencing, assembly and k-mer analysis: The answer provided is satisfactory, however this is not yet clear from the manuscript. The line (118) referred to just mentions 'eels', when the answer precisely specifies how many animals were used, why and for which analyses.

Response: (lines 116-120 and lines 131-134 in the new version)
Thanks for your suggestion. We have added the description for the usage of multiple eels and explanation for k-mer in lines 116-120 and lines 131-134 respectively as follows:
"To reduce the risk of non-random sequencing, 8 paired-end sequencing libraries with insert sizes of 170 bp, 500 bp, 800 bp, 2 kb, 5 kb, 10 kb, 20 kb and 40 kb were constructed. Of them, the library with 40 kb inserts was from second individual for assembling of long scaffolds. These libraries generated 101.62 GB of sequence data."
"The data used for k-mer analysis were derived from the male which was used to construct the genome sequencing libraries. The heterozygosity revealed from the k-mer analysis reflects the inner heterozygosity in an individual."

On my issue of placing 142 scaffolds using synteny only: this needs very careful phrasing. I think the point is that 8% of scaffolds could not be placed based on FISH, and that therefore perhaps 8% of 142 (therefore approximately 11) scaffolds placed by synteny only can be expected to be incorrectly placed. 'Can be expected' is quite different from 'could not be resolved' as stated in the answer. In the manuscript, I suggest rephrasing line 301 to something like 'Based on the results of the FISH experiments and collinearity analyses, we can expect approximately 8% placement errors amongst these 142 scaffolds.'

Response: (line 300-301 in the new version)
Thanks for your suggestion. We have replaced "According to the results of the FISH experiment and collinearity analysis, approximately 8% (~11) of the 142 scaffolds could not be resolved by the conserved synteny prediction due to possible rearrangements in the Monopterus lineage." with "Based on the results of the FISH experiments and collinearity analyses, we can expect approximately 8% placement errors amongst these 142 scaffolds."

Several bioinformatics tools could be properly cited, e.g. SSPACE (L146), Blat (L272), Cufflinks (L214), Circos (L471).

Response: SSPACE (line 147), Blat (line 271), Cufflinks (line 214), Circos (line 341) in the new version.

Thanks for your suggestion. We have added the corresponding references in the version.
——SSPACE (version 1.1)-reference 17
——Blat (version blat_34)-reference 37
——Cufflinks (version cufflinks-2.2.1)-reference 29
——Circos (version 0.69)-reference 39
In addition, we have added the description of Circos in line 341 of the article, “In this analysis, the Circos is used to plot the assembled chromosomes, GC content and gene density.”

Line 98: Reference 11 is probably not appropriate for nanopore sequencing?

Response: (line 95 in the new version)
Thanks. We have deleted the reference 11, where the reference 10 is appropriate and enough for nanopore sequencing information.

Line 108: 'the second-generation sequencing' -> second-generation sequencing. Same for L199, L304.

Response: (line 90, 105, 114, 387, 199, 302 in the new version)
Thanks. We have deleted “the” in these sentences, and the same as the “third-generation sequencing”.

Line 184: 'to analyze the Danio rerio …' -> to analyze the Danio rerio … genomes

Response: (line 184-185 in the new version)
Thanks. We have replaced “to analyze the Danio rerio…” with “to analyze the Danio rerio, Oryzias latipes, Gasterosteus aculeatus, Tetraodon nigroviridis, and Takifugu rubripes genomes”.

Line 198: contains -> containing

Response: (line 198 in the new version)
Thanks. We have replaced “contains …” with “containing …”.

Line 228: 'the program of DAVID program'?

Response: (line 228 in the new version)
Thanks. We have replaced “the program of DAVID program” with “the program DAVID”.

Line 229: 'on human/chicken GO term list' -> on a/the human/chicken GO term list

Response: (line 229-230 in the new version)
Thanks. We have corrected the sentence.

Line 241: The details of PCR fragments distract from the general description of the method. These details are again provided in line 276, where they are more appropriate.

Response: (lines 241-243, lines 275-277 in the new version)
Thanks. We have deleted the description in lines 241-243, and only retain the contents in lines 275-277.

Line 244: The parentheses are superfluous.

Response: (line 243 in the new version)
Thanks. We have deleted the parentheses.

Line 277: covers -> covering

Response: (line 276 in the new version)
Thanks. We have replaced "covers" with "covering".

Line 300: '142 scaffolds were…' -> 'An additional 142 scaffolds were…'

Response: (line 299 in the new version)
Thanks. We have replaced "142 scaffolds" with "an additional 142 scaffolds".

Line 346: 'probability of ≥ actual numbers', I think this could be clearer if written out in full.

Response: (line 344-346 in the new version)
Thanks. We have replaced "we computed the probability of ≥ actual numbers of ridges under a random permutation of the gene positions following previous method" with a complete sentence "we computed the probability of the events (ridge numbers under a random permutation of the gene positions ≥ actual numbers of ridges) following a method previously described".

Line 347: 'following previous method' -> following a method previously described

Response: (line 346 in the new version)
Thanks. We have corrected this sentence.

Line 473: 'into chromosomes filled the gaps' -> into chromosomes, with each gap (see figure 3) replaced with 50 kb of Ns

Response: (line 491 in the new version)
Thanks. We have replaced "into chromosomes filled the gaps" with "into chromosomes, with each gap (see figure 3) replaced with 1 kb of Ns". We also corrected the size of the gaps with 1 kb, as we have rechecked data and found the mistake in the previous writing.

Close
