# Supplementary material for: Chromosome-scale assembly of the Monopterus genome
Source: Gigascience. 2018 Apr 24;7(5):giy046. doi: 10.1093/gigascience/giy046 (PMC5946948; doi:10.1093/gigascience/giy046)
Supplement: Reviewer_1_Report_(Original_Submission) -- Matthias Platzer — 10/1/2017 Reviewed [file giy046_reviewer_1_report_(original_submission).pdf]

Reviewer Report

Title: Chromosome-scale assembly of the Monopterus genome

Version: Original Submission **Date:** 10/1/2017

Reviewer name: Matthias Platzer

Reviewer Comments to Author:

The manuscript contains valuable and interesting data/results, but the presentation lacks clarity and soundness in many places as well as does not meet scientific standards in a few cases.

MAJOR COMPULSORY REVISIONS

GENERAL
The selection of cutoffs 1/2 is not clear and seems to be highly subjective. Essential data (eg. map positions of ridges and IRSEs) is missing. Terminology centrally to the manuscript is not clear (eg. "interconvertible", "interconvertible region", "interconvertible domain" and "interconvertible manner").

lines 47/372/418
Clear overstatements: "we discovered a co-regulation mechanism", "found a co-regulation mechanism" and "showed a co-regulation mechanism".

306-310
The features (GWPs) in this correlation analysis are mutually (inversely) dependent. Moreover it is not clear whether the given p-value has been corrected for multiple testing.

314-319
The paragraph is conceptually/semantically not clear to me. What means "distribution pattern can be described in interconvertible regions", "IRSEs explain" and "emerge in an alternating, mutually interconvertible manner along the chromosome"? Fig8D and the reference therein to Fig6 are unclear.

321-331
It is not clear (neither from the Methods) how IRSEs are being defined and mapped. Therefore the presented data are not traceable. What means "suggesting an obvious association" in the Results. The IDSE definition and the concept as well as Fig9 are also not clear hand should have in no way been placed in the Results.

MINOR ESSENTIAL COMMENTS

34/67
No data are presented to support "enormous/high" medical value of the fish. The reference to a medieval writing indicates at best a "potential" medical value.

44
The terminology "types of genes" is misleading.

48/113
"IRSE" vs. "IDSE"?

91
"NGS" means not necessarily "short length of the reads". This refers only to 2nd generation technologies.

107
What are "complete genetic maps"?

110
Wording "chromosome-level assembly with 81.3% coverage of the sequenced genome" is not scientifically sound.

113
"reasonably explains" is not scientifically sound.

122
How "78.6 GB sequence dataset with a sequencing depth of 97-fold" was calculated?

128
Not "mapping to the genome" but mapping to the reference sequence of the genome.

135
"DNAs"?

136
"acted in concert"?

140
"the database"?

156-176
What means "group", "efficient combinations", "landmarks or the sequences", "order of another scaffold", "each scaffold"?

180
Rephrase "A scaffold was mapped on each chromosome by FISH, which was used as a landmark" and describe clearer how the landmark scaffolds were selected.

183-194
Where do the numbers 186/108142 come from and how they relate to each other? The sentence "142 of the scaffolds were further assembled into 12 chromosomes" is misleading. What means "issue should be further corrected"?

196/199
" We finally integrated 328 scaffolds into 12 chromosomes" is lab jargon. You assembled 328 scaffolds into reference sequences for 12 chromosomes. The same refers to "sequenced genome of 683 Mb with 97.6X". You sequenced a genome and assembled a reference sequence of 683 Mb that is (most likely - please provide the overall length of assembled reads eg. as supplement) 97.6x covered by aligned reads.

203/207
What mean "efficient" and "most attractive"?

213-217
What means "deep transcriptome sequencing"? It must be explained at least in Methods. What means "distribution patterns of genes on chromosomes in accordance with their expression profiles"? The statement "significant positive correlation between the gene density and expression" should be better

explained/supported in the main text. The terms "gene density" and "expression" are not introduced Figures show "Gene number". The reference to Fig4B is not helpful. R values should be given together with the p-value in the main text. For any p-values the respective test should be indicated.

227
The statement "distribution pattern of the gene density was consistent with the corresponding GC content" is not supported by data.

234-239
Define "consecutive window numbers" unambiguously. Not "distribution pattern of the ridges" but numbers of ridges have been defined. What means "significantly low", "(ridge numbers in random ≥ observations", "highly significant difference in ridges with high gene density"?

245
The statement "These analyses suggest that the ridge pattern on the chromosomes probably represents a higher-order structure in the genome" belongs to Discussion not to Results.

250/739
Clarify the meaning of "key gene types during gonad transition" and "gene regulation pathways in gonad transition".

255-259
What means "clearly"? Please provide gene numbers/fractions for each type as suppl table. What means "the GO terms in the biological process category were over-represented in all types"?

281-283
What means "high degree" when comparing types V and II as well as "clustered on chromosomes in a large-scale"?

286-287
What means "each type of gene" and "continuous degree of gene clustering"?

292-296
How the p-values were obtained (corrected for multiple testing) and how they relate to "*/**" in Fig7B.

336
What means "complete genome information" - a complete and error free as possible reference sequence?

337-342
Please provide a justification/reference for the statement.

343
What means "the other 22 species"?

345
Please provide data for "efficient and cost effective".

348
The statement "81.3% coverage of the sequenced genome is most likely an overestimation.

353
Provide data for "improve the understanding of the mechanisms of genome".

355-356

What means "favoured genes" and "QTLs of economic importance".

372
Clear overstatement: "found a co-regulation mechanism".

375-377
Clarify what means "cluster" in this context and whether in your concept an IDSE is formed by a subset of genes of a "ridge"

383
What means "IRSE-o should occupy a different topological domain from the IRSE-t".

394
Provide data for "IDSEs are closely associated with sex determination".

428
To my understanding no "animal experiments" have been performed.

585
the statement "three tissues (ovary, ovotestis, and testis)" is not corre ct. How many biological replicates were identified?

596-597
Clarify whether "RPKM<0.1" refers to one or both analyzed states. What means "FDR (false discovery rate) < 0.05 (p-value<0.01)"?

741-745
The statement "Differential expression is identified by fold change of RPKM (≥2)" contradicts those in Methods. What means "p-value = 0.041 (DAVID, version 6.7). Fisher's exact test (FDR = 0.05) was used to assess the enrichment"?

752-764
The figure as such does not show "Clustering patterns". What are "typical genes" and "intensity of clusters"? "and at least" should read as "in at least"?

766-778
Phrasing "consecutive gene clusters of co-regulated genes" is misleading and should read as "clusters of consecutive co-regulated genes". Significance is not explained (test, thresholds, multiple test correction).

786
What means "alternating sine curves"?

793
Are the p-values corrected for multiple testing?

803-810
The legend does not sufficiently explain what is shown in the different panels.

Tab 1
What "*" refers to? What means "final contig length after filling intra-scaffold gaps"? Does the overall scaffold length includes Ns?

Tab2
"Chromosome length" is incorrect/misleading.

Fig5B
Cyp51 in the left panel is shaded according to log2(FC)=2 (ie. FC=4) but the right panel shows a FC<3.

FigS12
What means "gene numbers in each catalog"?

FigS13
The title does not cover type IX.

DISCRETIONARY COMMENTS

39
"Cafs" is not clear here.

87-90
Mixing "genetic" and "physical" maps is confusing for an non-geneticist reader.

98
"BAC/fosmid paired end sequencing from large-insert libraries" is a tautology.

106
"map on chromosomes" map of chromosomes?

128
Not "Sanger sequence technology" but "Sanger sequencing technology".

146
Replace "Chromosome-level assembly strategy without a genetic map" by "Chromosome assembly by FISH walking assisted by conserved synteny - strategy outline", outline the strategy in this section and provide the details for the actual project in the next section.

346
Do "gene functions" have a "position in the genome"?

376
Introduce abbreviations only once in the main text.

[19] is rather outdated.

TabS9
is incomplete.

Methods

Are the methods appropriate to the aims of the study, are they well described, and are necessary controls included? Yes

Conclusions

Are the conclusions adequately supported by the data shown? Yes

Reporting Standards

Does the manuscript adhere to the journal's guidelines on minimum standards of reporting? Yes

Choose an item.

Statistics

Are you able to assess all statistics in the manuscript, including the appropriateness of statistical tests used? Yes, and I have assessed the statistics in my report.

Quality of Written English

Please indicate the quality of language in the manuscript: Needs some language corrections before being published

Declaration of Competing Interests

Please complete a declaration of competing interests, considering the following questions:

- Have you in the past five years received reimbursements, fees, funding, or salary from an organisation that may in any way gain or lose financially from the publication of this manuscript, either now or in the future?
- Do you hold any stocks or shares in an organisation that may in any way gain or lose financially from the publication of this manuscript, either now or in the future?
- Do you hold or are you currently applying for any patents relating to the content of the manuscript?
- Have you received reimbursements, fees, funding, or salary from an organization that holds or has applied for patents relating to the content of the manuscript?
- Do you have any other financial competing interests?
- Do you have any non-financial competing interests in relation to this paper?

If you can answer no to all of the above, write 'I declare that I have no competing interests' below. If your reply is yes to any, please give details below.

no competing interersts

I agree to the open peer review policy of the journal. I understand that my name will be included on my report to the authors and, if the manuscript is accepted for publication, my named report including any attachments I upload will be posted on the website along with the authors' responses. I agree for my report to be made available under an Open Access Creative Commons CC-BY license

(http://creativecommons.org/licenses/by/4.0/). I understand that any comments which I do not wish to be included in my named report can be included as confidential comments to the editors, which will not be published.

I agree to the open peer review policy of the journal

To further support our reviewers, we have joined with Publons, where you can gain additional credit to further highlight your hard work (see: https://publons.com/journal/530/gigascience). On publication of this paper, your review will be automatically added to Publons, you can then choose whether or not to claim your Publons credit. I understand this statement.

Yes Choose an item.
