# Supplementary material for: Chromosome-scale assembly of the Monopterus genome
Source: Gigascience. 2018 Apr 24;7(5):giy046. doi: 10.1093/gigascience/giy046 (PMC5946948; doi:10.1093/gigascience/giy046)
Supplement: Reviewer_2_Report_(Original_Submission) -- Christiaan Henkel — 11/20/2017 Reviewed [file giy046_reviewer_2_report_(original_submission).pdf]

Reviewer Report

Title: Chromosome-scale assembly of the Monopterus genome

Version: Original Submission **Date:** 11/20/2017

Reviewer name: Christiaan Henkel

Reviewer Comments to Author:

This manuscript describes both the genome assembly of the swamp eel, and some transcriptomics experiments relating to its sex-reversal physiology.

The genome assembly process follows a straightforward short-read shotgun recipe, but is supplemented by in situ hybridization of scaffold probes to arrive at a chromosome-level assembly. This is presented as a novel strategy, which I am uncertain is entirely justified. It is, however, a worthwhile effort. The supposed novelty is entirely related to the pre-selection of co-hybridization probes based on synteny information. In general, I would be very cautious about using comparative evidence to finish a chromosome-level assembly, as any subsequent comparative genomics studies run the risk of circular reasoning. In this case, it appears the comparative evidence is mostly only used as an initial screening, and it does not directly inform the assembly process - but please see my issue 6 below.

Several issues about the assembly part I would ask the authors to address:

1. Is it correct that the assembly is based on multiple discrete scaffolding procedures (SOAPdenovo, l. 456, and SSPACE, l. 459) using the same data? Does this not increase the risk of artifacts?
2. Only ~79% of the expected genome size is captured in contigs (634.7 Mbp of 806 Mbp). Therefore, presumably, these contigs contain abundant collapsed repeats, affecting an analysis of repetitive content (l. 131-138, fig S4). Repeat abundance will likely be underestimated.
3. Line 136: please rephrase 'they acted', as no evidence on piRNAs is presented.
4. No completeness analysis of the assembly/gene prediction is presented. Please include a BUSCO analysis of the predicted gene set.
5. Line 142/fig S5: 'The gene sizes were similar to those of other teleost fish'. In fact, the figure shows that the Monopterus gene set contains many more short genes and CDSs than other teleosts. Exon lengths are comparable. This suggests truncated genes or gene predictions.
6. FISH walking (l. 180-203): Is it correct that no FISH evidence is used to assign 142 scaffolds to chromosomes (l. 191)? How will placing these scaffolds using comparative evidence only affect comparative analyses?
7. Figure 3B: I do not see the added value of the visualization of gene prediction for this single scaffold. Similarly for figure 4C.
8. Figure 3A and 4B: The chromosome sizes here add up to 555 Mbp, which is also the size of scaffolds assigned to chromosomes (l. 198) but much less than the expected genome size of 806 Mbp. If these sizes are entirely based on placed scaffolds, why do these illustrations still contain gaps?
9. Related: I assume the coloured block in the outer ring of fig 4B are placed scaffolds, and the white spaces gaps. How can these gaps then contain genes, have a GC content, and gene expression (inner rings)?

The second part of the paper addresses sex transition, based on RNA-seq data. I have not reviewed the downstream analyses and conclusions in detail, as I believe the initial RNA-seq data is both insufficient and not analyzed appropriately:

10. There is no description of the RNA-seq raw data. How many reads per sample, etc.?
11. There is no mention of biological replication, therefore the entire analysis appears to be based on one sample per developmental stage - insufficient to draw strong conclusions from.

12. Differential expression analysis is not described in sufficient detail (l. 594-598). These do not appear to be industry-standard statistical analyses. In any case, without replication, how does one test for statistical significance of differential expression?

13. RPKM normalization is usually deprecated, unless it can be argued that it is suitable. In this case, comparing the tissues requires major assumptions anyway, as they do not appear to be intrinsically comparable. I have attached density plots of the gene expression values in the three cases. This is after normalization: with successful normalization, the plots would align. In this case, it is far from clear this is even possible. I do not see how in this case, expression in testis can be related to the other samples. My quick scatterplots of gene expression show a similar picture.

14. I therefore do not see how these data can be used to reliably infer the patterns shown in fig 5A. (Related, l. 252: these classes/'types' are not differentially expressed themselves, they correspond to genes that are 'differentially expressed' in any one sample compared to the other two). The important/large classes IV and V are probably mostly populated by genes affected by noise/the effects I describe in issue 13.

15. Most statements about gene expression or its classification are based on very low expression, which especially in this case (no replication, poor normalization) is not reliable. For instance: fig S9 correlations (or are these log-values?), dhcr24 (l. 265, fig 5B), fgf8b (fig 6B).

Methods

Are the methods appropriate to the aims of the study, are they well described, and are necessary controls included? No

Conclusions

Are the conclusions adequately supported by the data shown? No

Reporting Standards

Does the manuscript adhere to the journal's guidelines on [minimum standards of reporting?]() Yes

Choose an item.

Statistics

Are you able to assess all statistics in the manuscript, including the appropriateness of statistical tests used? Yes, and I have assessed the statistics in my report.

Quality of Written English

Please indicate the quality of language in the manuscript: Needs some language corrections before being published

Declaration of Competing Interests

Please complete a declaration of competing interests, considering the following questions:

- Have you in the past five years received reimbursements, fees, funding, or salary from an organisation that may in any way gain or lose financially from the publication of this manuscript, either now or in the future?

- Do you hold any stocks or shares in an organisation that may in any way gain or lose financially from the publication of this manuscript, either now or in the future?
- Do you hold or are you currently applying for any patents relating to the content of the manuscript?
- Have you received reimbursements, fees, funding, or salary from an organization that holds or has applied for patents relating to the content of the manuscript?
- Do you have any other financial competing interests?
- Do you have any non-financial competing interests in relation to this paper?

If you can answer no to all of the above, write 'I declare that I have no competing interests' below. If your reply is yes to any, please give details below.

I declare that I have no competing interests

I agree to the open peer review policy of the journal. I understand that my name will be included on my report to the authors and, if the manuscript is accepted for publication, my named report including any attachments I upload will be posted on the website along with the authors' responses. I agree for my report to be made available under an Open Access Creative Commons CC-BY license (http://creativecommons.org/licenses/by/4.0/). I understand that any comments which I do not wish to be included in my named report can be included as confidential comments to the editors, which will not be published.

I agree to the open peer review policy of the journal

To further support our reviewers, we have joined with Publons, where you can gain additional credit to further highlight your hard work (see: https://publons.com/journal/530/gigascience). On publication of this paper, your review will be automatically added to Publons, you can then choose whether or not to claim your Publons credit. I understand this statement.

Yes Choose an item.
