# Supplementary material for: Chromosome-scale assembly of the Monopterus genome
Source: Gigascience. 2018 Apr 24;7(5):giy046. doi: 10.1093/gigascience/giy046 (PMC5946948; doi:10.1093/gigascience/giy046)
Supplement: Reviewer_2_Report_(Revision_1) -- Christiaan Henkel — 2/5/2018 Reviewed [file giy046_reviewer_2_report_(revision_1).pdf]

Reviewer Report

Title: Chromosome-scale assembly of the Monopterus genome

Version: Revision 1 **Date:** 2/5/2018

Reviewer name: Christiaan Henkel

Reviewer Comments to Author:

The authors have improved the manuscript considerably. I think it was wise to omit the transcriptomics study and focus on the genome assembly, which is quite complex already.Even though the RNA-seq study has been left out, I would like to suggest paying close attention to the implicit assumptions usually present in RNA-seq analysis packages. In this case, comparing between very different samples (my previous issue 12) will be challenging, and switching to a different normalization method ('re-analysis the RNAseq data by credible FPKM ... normalization show the RNAseq are correct') probably does not address this problem.Many of the issues I previously raised on the first part have been addressed, but there are a few I would like to revisit:2.Only ~79% of the expected genome size is captured in contigs (634.7 Mbp of 806Mbp). Therefore, presumably, these contigs contain abundant collapsed repeats,affecting an analysis of repetitive content (...) Repeat abundance willlikely be underestimated. (line 188-195 in the new version).Responses: The genome size is 806Mb estimated by K-mer, but only 634.7 Mb wascaptured in contigs based on the data of second generation sequencing. This mostlikely results from a lot of repetitive sequences in the genome. The incompleteassemble is the limitation of the second-generation sequencing technology. Therepetitive sequences we analyzed are based on the assembled genome. Thus, as yousaid, repeat sequences would be underestimated. We have discussed the fact in theversion.The underestimation of repeat abundance is not discussed in the new version. In addition, the BUSCO analysis suggests the 634.7 Mbp genome assembly to be very complete (line 167-173). This suggests the rest of the genome to consist of non-coding DNA, possibly containing many repeats. Unless the 806 Mbp k-mer-based estimate is an overestimate?3.Line 136: please rephrase 'they acted', as no evidence on piRNAs is presented.（line 194 in the new version）Responses: Several papers have reported that the piRNAs derived from LINE are veryclosely related with genome stability. We have just discussed the fact that LINEs wasgreater than that found in other teleost fish (2-5%), which were involved in guardiansfor genome defense and germline stability via Piwi-interacting small RNAs (piRNAs)[23].I did not want to dispute the biology here (but see below), but just wanted to point out that this is a speculative point of discussion. The current study presents no evidence on piRNAs, therefore the phrasing should be adjusted. The current 'LINEs ... were involved in ... genome defense ... via Piwi-interacting small RNAs' (l193-194) suggests the LINEs were actually studied. So I would suggest rephrasing 'were involved' to 'might be involved'.Regarding the biology - doesn't a high LINE content suggest genome defense (by piRNA) has been ineffective?5.Line 142 /fig S5: 'The gene sizes were similar to those of other teleost fish'. In fact,the figure shows that the Monopterus gene set contains many more short genes andCDSs than other teleosts. Exon lengths are comparable. This suggests truncatedgenes or gene predictions. (line 214 in the new version)Responses: BUSCO analysis showed that there are some numbers of fragmented andmissing BUSCOs, but very low.Indeed, the BUSCO analysis suggests the assembly is complete. However, looking at figure S5, CDS and mRNA lengths are noticeably shorter in Monopterus. This is not reflected in the averages (table S5).6.FISH walking (l. 180-203): Is it correct that no FISH evidence is used to assign 142scaffolds to chromosomes (l. 191)? How will placing these scaffolds using comparativeevidence only affect comparative analyses? (line 263-307 in the new version)Responses: the data of 142 scaffolds were from comparative genomics analysis, notfrom FISHing experiments. We have discussed this issue, pointed out the possibleerror rates and problems. (line 295-298 in the new version)The discussion of this issue is minimal (l. 299). The way I read it, the chromosome-scale assembly may contain large structural errors: 8% of FISH-based placements are inconsistent with synteny (l. 294), therefore conserved synteny should be used with a lot of caution. Yet a further 100 Mb of sequence is placed based on synteny only (l. 302). Some scaffolds are apparently left out (l. 297), but the numbers do not add up? 186 + 142 = 328 scaffolds have been placed (l. 301), but of the 142 some (approximately! 11, l. 297) have

been left out?9.Related: I assume the coloured block in the outer ring of fig 4B are placed scaffolds,and the white spaces gaps. How can these gaps then contain genes, have a GCcontent, and gene expression (inner rings)?Responses: Please see the point 8 above.The outer ring of figure 4A has a different scale than all the inner content, which is confusing as this is a non-standard way of presenting these data. The gaps (white space) in the outer ring are not represented in the inner graphs. At least please note this in the figure legend.Some comments on the current version:L99-100: 'high costs of sequencing', is this still the case? L103-104: 'assemble chromosomes, which can produce ultra-long scaffolds': I think this should be the other way around (e.g. assembled ultra-long scaffolds can lead to a chromosome-scale assembly)?L119: Does using two individuals lead to assembly problems (due to heterozygosity)? Presumably not, based on the k-mer analyses (figure S2)?L153/163: These percentages (91.06%/99.7%) appear to refer to the same analysis? I think the latter refers to the 99.69% in line 153, but those are not 'reads mapping'. L235: 'closely related species' -> 'distantly related species', they are in different orders.L325: 'The distribution pattern of the gene density was consistent with the corresponding GC content along the chromosomes'. This refers to the correlation between these two measures. However, figure S9 does not show a clear correlation. In fact, I expect the entire positive correlation to be caused by a few outliers (high GC, high gene content, probably on chromosome 12, see figure 4A). The overall trend is not obvious.

Level of Interest

Please indicate how interesting you found the manuscript: An article whose findings are important to those with closely related research interests

Quality of Written English

Please indicate the quality of language in the manuscript: Needs some language corrections before being published

Declaration of Competing Interests

Please complete a declaration of competing interests, considering the following questions:

- Have you in the past five years received reimbursements, fees, funding, or salary from an organisation that may in any way gain or lose financially from the publication of this manuscript, either now or in the future?
- Do you hold any stocks or shares in an organisation that may in any way gain or lose financially from the publication of this manuscript, either now or in the future?
- Do you hold or are you currently applying for any patents relating to the content of the manuscript?
- Have you received reimbursements, fees, funding, or salary from an organization that holds or has applied for patents relating to the content of the manuscript?
- Do you have any other financial competing interests?
- Do you have any non-financial competing interests in relation to this paper?

If you can answer no to all of the above, write 'I declare that I have no competing interests' below. If your reply is yes to any, please give details below.

I declare that I have no competing interests

I agree to the open peer review policy of the journal. I understand that my name will be included on my report to the authors and, if the manuscript is accepted for publication, my named report including any attachments I upload will be posted on the website along with the authors' responses. I agree for my report to be made available under an Open Access Creative Commons CC-BY license (http://creativecommons.org/licenses/by/4.0/). I understand that any comments which I do not wish to be included in my named report can be included as confidential comments to the editors, which will not be published.

I agree to the open peer review policy of the journal

To further support our reviewers, we have joined with Publons, where you can gain additional credit to further highlight your hard work (see: https://publons.com/journal/530/gigascience). On publication of this paper, your review will be automatically added to Publons, you can then choose whether or not to claim your Publons credit. I understand this statement.

Yes
