# Supplementary material for: Chromosome-scale assembly of the Monopterus genome
Source: Gigascience. 2018 Apr 24;7(5):giy046. doi: 10.1093/gigascience/giy046 (PMC5946948; doi:10.1093/gigascience/giy046)
Supplement: Reviewer_2_Report_(Revision_2) -- Christiaan Henkel — 3/21/2018 Reviewed [file giy046_reviewer_2_report_(revision_2).pdf]

**Reviewer Report**

**Title: Chromosome-scale assembly of the Monopterus genome**

**Version: Revision 2** **Date:** 3/21/2018

**Reviewer name: Christiaan Henkel**

**Reviewer Comments to Author:**

I thank the authors for improving the manuscript's clarity to the point where it is in principle publishable. Please find below a few final suggestions for minor additional clarifications.On my issue of using multiple eels for sequencing, assembly and k-mer analysis: The answer provided is satisfactory, however this is not yet clear from the manuscript. The line (118) referred to just mentions 'eels', when the answer precisely specifies how many animals were used, why and for which analyses.On my issue of placing 142 scaffolds using synteny only: this needs very careful phrasing. I think the point is that 8% of scaffolds could not be placed based on FISH, and that therefore perhaps 8% of 142 (therefore approximately 11) scaffolds placed by synteny only can be expected to be incorrectly placed. 'Can be expected' is quite different from 'could not be resolved' as stated in the answer. In the manuscript, I suggest rephrasing line 301 to something like 'Based on the results of the FISH experiments and collinearity analyses, we can expect approximately 8% placement errors amongst these 142 scaffolds.'Several bioinformatics tools could be properly cited, e.g. SSPACE (L146), Blat (L272), Cufflinks (L214), Circos (L471).Line 98: Reference 11 is probably not appropriate for nanopore sequencing?Line 108: 'the second-generation sequencing' -> second-generation sequencing. Same for L199, L304.Line 184: 'to analyze the Danio rerio ...' -> to analyze the Danio rerio ... genomesLine 198: contains -> containingLine 228: 'the program of DAVID program'?Line 229: 'on human/chicken GO term list' -> on a/the human/chicken GO term listLine 241: The details of PCR fragments distract from the general description of the method. These details are again provided in line 276, where they are more appropriate.Line 244: The parentheses are superfluous.Line 277: covers -> coveringLine 300: '142 scaffolds were...' -> 'An additional 142 scaffolds were...'Line 346: 'probability of ≥ actual numbers', I think this could be clearer if written out in full.Line 357: 'following previous method' -> following a method previously describedLine 473: 'into chromosomes filled the gaps' -> into chromosomes, with each gap (see figure 3) replaced with 50 kb of Ns

**Level of Interest**

Please indicate how interesting you found the manuscript: An article whose findings are important to those with closely related research interests

**Quality of Written English**

Please indicate the quality of language in the manuscript: Needs some language corrections before being published

**Declaration of Competing Interests**

Please complete a declaration of competing interests, considering the following questions:

- Have you in the past five years received reimbursements, fees, funding, or salary from an organisation that may in any way gain or lose financially from the publication of this manuscript, either now or in the future?
- Do you hold any stocks or shares in an organisation that may in any way gain or lose financially from the publication of this manuscript, either now or in the future?
- Do you hold or are you currently applying for any patents relating to the content of the manuscript?
- Have you received reimbursements, fees, funding, or salary from an organization that holds or has applied for patents relating to the content of the manuscript?
- Do you have any other financial competing interests?
- Do you have any non-financial competing interests in relation to this paper?

If you can answer no to all of the above, write 'I declare that I have no competing interests' below. If your reply is yes to any, please give details below.

I declare that I have no competing interests

I agree to the open peer review policy of the journal. I understand that my name will be included on my report to the authors and, if the manuscript is accepted for publication, my named report including any attachments I upload will be posted on the website along with the authors' responses. I agree for my report to be made available under an Open Access Creative Commons CC-BY license (http://creativecommons.org/licenses/by/4.0/). I understand that any comments which I do not wish to be included in my named report can be included as confidential comments to the editors, which will not be published.

I agree to the open peer review policy of the journal

To further support our reviewers, we have joined with Publons, where you can gain additional credit to further highlight your hard work (see: https://publons.com/journal/530/gigascience). On publication of this paper, your review will be automatically added to Publons, you can then choose whether or not to claim your Publons credit. I understand this statement.

Yes
